# IPNA clinical practice recommendations for the diagnosis and management of children with IgA nephropathy and IgA vasculitis nephritis

**DOI:** 10.1007/s00467-024-06502-6

**Published:** 2024-09-27

**Authors:** Marina Vivarelli, Susan Samuel, Rosanna Coppo, Jonathan Barratt, Melvin Bonilla-Felix, Dieter Haffner, Keisha Gibson, Mark Haas, Maher Ahmed Abdel-Hafez, Marta Adragna, Paul Brogan, Siah Kim, Isaac Liu, Zhi-Hong Liu, Mukta Mantan, Yuko Shima, Masaki Shimuzu, Qian Shen, Hernan Trimarchi, Deirdre Hahn, Elisabeth Hodson, Ken Pfister, Areefa Alladin, Olivia Boyer, Koichi Nakanishi, the International Pediatric Nephrology Association

**Affiliations:** 1 Laboratory of Nephrology, Bambino Gesù Children’s Hospital, IRCCS, Piazza S. Onofrio 4 00165, Rome, Italy; 2 Section of Nephrology, Department of Pediatrics, University of Calgary, Calgary, Canada; 3 Fondazione Ricerca Molinette, Regina Margherita Hospital, Turin, Italy; 4 University of Leicester, Leicester, England UK; 5 Department of Pediatrics, University of Puerto Rico—Medical Sciences Campus, San Juan, , Puerto Rico; 6 Department of Pediatric Kidney, Liver and Metabolic Diseases, Hannover Medical School, Hannover, Germany; 7 University of North Carolina at Chapel Hill, Chapel Hill, NC USA; 8 Cedars-Sinai Medical Center, Los Angeles, CA USA; 9 Tanta University, Tanta, Egypt; 10 Hospital de Pediatría Prof. Dr. Juan P. Garrahan, Buenos Aires, Argentina; 11 University College London Great Ormond Street Institute of Child Health, London, England UK; 12 Children’s Hospital at Westmead, Westmead, Australia; 13 Duke-NUS Medical School and YLLSOM, National University of Singapore, Singapore, Singapore; 14 Nanjing University School of Medicine, Nanjing, China; 15 Maulana Azad Medical College, University of Delhi, Delhi, India; 16 Wakayama Medical University, Wakayama, Japan; 17 Department of Pediatrics and Developmental Biology, Graduate School of Medical and Dental Sciences, Tokyo Medical and Dental University, Bunkyo, Japan; 18 Children’s Hospital of Fudan University, Shanghai, China; 19 Hospital Britanico de Buenos Aires, Buenos Aires, Argentina; 20 Cochrane Kidney and Transplant, Westmead, Australia; 21 Department of Pediatrics, University of Calgary, Calgary, Canada; 22 University of Guyana, Georgetown, Guyana; 23 Pediatric Nephrology, MARHEA Reference Center, Imagine Institute, Paris Cité University, Necker Children’s Hospital, APHP, Paris, France; 24 Department of Child Health and Welfare (Pediatrics), Graduate School of Medicine, University of Ryukyus, Nishihara, Okinawa Japan

**Keywords:** IgA nephropathy, IgA vasculitis nephritis, Children, Treatment, Guideline

## Abstract

IgA nephropathy and IgA vasculitis with nephritis, albeit rare, represent two relatively frequent glomerular conditions in childhood. Compared to adults, pediatric IgA nephropathy has a more acute presentation, most frequently with synpharyngitic macrohematuria and histologically with more intense inflammation and less intense chronic damage. Management of these conditions is controversial and supported by little high-quality evidence. The paucity of evidence is due to the disease heterogeneity, its inter-ethnic variability, and the difficulty of extrapolating data from adult studies due to the peculiarities of the condition in children. IgA vasculitis with nephritis is a kidney manifestation of a systemic disorder, typical of the pediatric age, in which both the diagnosis of kidney involvement and its management are poorly defined, and an interdisciplinary approach is crucial. Both conditions can have a profound and long-lasting impact on kidney function and the global health of affected children. The International Pediatric Nephrology Association has therefore convened a diverse international group of experts from different disciplines to provide guidance on the recommended management of these conditions in children and to establish common definitions and define priorities for future high-quality, evidence-based collaborative studies for the benefit of children.

**Supplementary Information:**

The online version contains supplementary material available at 10.1007/s00467-024-06502-6.

## Introduction

Immunoglobulin A nephropathy (IgAN) is the most common primary glomerulonephritis worldwide [1–3], caused by the deposition of IgA1-IgG immune complexes in the glomeruli, triggering a cascade of inflammatory events that lead to kidney damage [4]. Diagnosis of IgAN is made through a kidney biopsy, characterized by dominant or co-dominant IgA deposits, the hallmark of IgAN [2, 4]. IgA nephropathy can present throughout the life course but is most common in the second and third decades of life [3]. However, if present in children, the median age of presentation is 10.9 years (range 2.5–19.6 years) [5].

The incidence of IgAN in children varies according to geography, population, urine screening, and kidney biopsy practices. A precise incidence is difficult to determine since mass urine screening is not routinely available in most countries, and kidney biopsies are not performed for all suspected cases [3, 6]. The estimated annual IgAN incidence in pediatric patients was 0.2 per 100,000 in those under the age of 15 or 17 years, depending on country-specific published reports in Europe [7, 8]. Countries that do not have mass urine screening programs have an incidence rate for IgAN ranging from 0.1 to 2 cases/year per 100,000 population, while countries with mass urine screening have much higher incidence rates ranging from 4.5 to 9.9 cases/year per 100,000 children under 15 years of age [3, 6, 8]. It commonly presents with blood in the urine (hematuria), with or without proteinuria, and variable degrees of kidney dysfunction. The degree of proteinuria and kidney dysfunction vary at presentation and during the disease course [9]. Rarely, IgAN presents as a rapidly progressive glomerulonephritis, which is more common in adolescents and adults compared to younger children [10].

IgAN is a leading cause of chronic kidney disease (CKD) and kidney failure [2, 11]. In adults, 30–40% of patients with IgAN will develop progressive kidney failure requiring kidney replacement therapy (dialysis or transplantation) after 20–30 years of follow-up [3, 10]. Pooled cohorts of adults aged 29.2–49.2 years from the Oxford Classification of IgAN study, Oxford derivation, the North American, and the European Validation study comparing the prediction of kidney outcomes in IgAN (VALIGA) found 5-year and 10-year risks of 50% eGFR loss or kidney failure of 11.2% and 26.8%, respectively [12]. On the other hand, in a population of 281 children who were detected through school urine screening programs after 1990 and appropriately treated, a 15-year kidney survival rate of 98.8% (95% CI 97.7–100) has been reported [13]. More recently, the National Registry of Rare Kidney Diseases (RaDaR) study showed kidney survival rates in IgAN patients in the UK after 20–30 years of follow-up to be in the range of 50% for children and 25% for adults [14]. This wide range of outcomes depends on many variables, among which ethnicity plays a central role [15, 16].

As recently confirmed by a large study performed on a Chinese population, IgAN differs significantly in its presentation between children and adults. Although this large study needs to be validated in patients of different ethnicities and a longer follow-up, it confirms that while adults are mostly diagnosed following asymptomatic urinary screening or symptomatic proteinuria, children typically present with intra-infectious gross hematuria, leading to early diagnosis by kidney biopsy [17]. The lesions on the biopsy typically are more intensely inflammatory in children, with less chronic changes compared to adults with IgAN [17]. Crucially, the authors of this study suggest that these distinctive features have implications on the optimal management of children with IgAN, in whom a more aggressive anti-inflammatory approach, primarily with glucocorticoids, has a more beneficial effect in terms of long-term outcome in children than in adults [17].

IgA vasculitis (IgAV) is an autoimmune disorder characterized by inflammation of small blood vessels caused by perivascular deposition of IgA and activation of neutrophils [18]. The annual incidence is estimated at 3 to 27 pediatric cases per 100,000 children. It is a systemic disease involving the skin, gut, joints, and kidneys, and represents the most common small vessel vasculitis in children [18, 19]. Kidney involvement is reported in 20–54% of children with IgAV and is referred to as IgA vasculitis with nephritis (IgAVN); however, risk factors for kidney involvement are not well established [19]. In the presence of established IgAV, the diagnosis of IgAVN is usually based on clinical presentation and is heralded by the onset of hematuria, proteinuria, hypertension, and/or decreased kidney function. Compared to adults, in whom IgAV with nephritis is extremely rare and frequently unfavorable in outcome [20], with a severity that increases in parallel with age [20], in children, IgAV with nephritis tends to be more benign and, in some occasions, self-remitting, though also in children severity increases with age [20]. A kidney biopsy is usually reserved for those with severe kidney involvement (nephrotic-range proteinuria, decreased kidney function) [21]. Additionally, IgAVN may progress to kidney failure in 1–15% of cases [18]. Overall, IgAN and IgAVN share many similarities, both in kidney histology and in underlying pathogenesis. However, IgAVN affects younger children with a mean age of 6 years [18]. Systemic features of IgAVN may require an individualized therapeutic approach compared to IgAN. Data supporting the use of glucocorticoids in patients with IgAN and IgAVN across the spectrum of severity is limited, and the effectiveness of immunosuppressive treatments to prevent the progression of CKD or kidney failure is debated.

Recently, Kidney Disease Improving Global Outcomes (KDIGO) established a comprehensive evidence-based guideline for the treatment of patients with glomerular diseases which included patients with IgAN and IgAVN and was mainly focused on adults [2]. However, the KDIGO guideline was created without broad-based representation and input from the pediatric nephrology community. Therefore, the International Pediatric Nephrology Association (IPNA) deemed that creating clinical practice recommendations (CPRs) for the diagnosis and management of children with IgAN and IgAVN is important to inform best care and decrease practice variation for these patients. Recommendations for future research are given which are aimed at closing the knowledge gap in children with IgAN and IgAVN.

## Methods

### Overview of the guideline project

The IPNA Best Practice and Standards Committee has adopted the use of the RIGHT (*R*eporting *I*tems for practice *G*uidelines in *H*eal*T*hcare) Statement for Practice Guidelines for the creation of CPRs [22]. A core leadership group (27 members including 18 pediatric nephrologists, 2 pediatric rheumatologists, 2 adult nephrologists, 1 kidney pathologist, 2 research trainees), an external expert group, and a voting panel were assembled. The core group conducted the systematic literature search and prepared the evidence review tables under the supervision of a pediatric nephrologist with expertise in epidemiology and two patient representatives. The expertise and responsibilities of the core group members are provided in Supplementary Table S1. The external expert panel consisted of 24 experts (22 pediatric nephrologists representing all IPNA regional societies, one pediatric rheumatologist, and one adult nephrologist). The core group provided a draft manuscript to the patient representatives, who reviewed it and provided feedback from relevant patient and family associations. The voting panel of representatives, selected from each IPNA Regional Society as experts in IgAN and IgAVN in children, was composed of 16 pediatric nephrologists and 2 pediatric rheumatologists (who voted on IgAVN only). Voting group representatives provided their level of agreement using electronic surveys which posted the question as per the Delphi method, and a 5-point scale with the options “strongly disagree,” “disagree,” “neither agree/disagree,” “agree,” and “strongly agree” was used. For topics that did not achieve a 70% level of consensus, the recommendations were then edited by the core group and voted on again until a consensus level of > 70% was achieved.

### Developing clinical questions for the guideline

We developed the questions to be answered in the guideline using the PICO format (*P*atient or *P*opulation, *I*ntervention, *C*omparator, *O*utcome) and the following definitions: *Population*, children and youth (< 18 years) with IgAN or IgAVN; *Intervention and Comparators*, treatment compared with no treatment, other treatment, or placebo; *Outcomes*, changes in clinical biomarkers (urine protein, serum creatinine, estimated glomerular filtration rate (eGFR), kidney replacement therapy, kidney failure, interventions for treatment to induce remission or delay in progression of kidney disease).

### Literature search

We searched the PubMed database for relevant articles published by August 8, 2022. We retained all systematic reviews of randomized controlled trials (RCTs) on the treatment of IgAN and IgAVN in children, prospective uncontrolled trials, observational studies, biopsy classification and validation studies, and registry studies on the diagnosis and management of children with IgAN and IgAVN and restricted our search to human studies. Non-English language abstracts were also considered and translated into English whenever possible. Risk ratios (RR) with 95% confidence intervals (CI) were cited from two Cochrane systematic reviews evaluating RCTs of interventions for adult-onset IgAN and childhood IgAVN [23]. The literature search was also updated with relevant articles as they became available during manuscript preparation up to May 1, 2024. The publications used and a summary of the articles are provided in Supplementary Tables S2 and S3 for IgAN and Supplementary Tables S7 and S8 for IgAVN, while the search strategy is detailed in Supplementary Table S9.

### Grading system

**Fig. 1:**
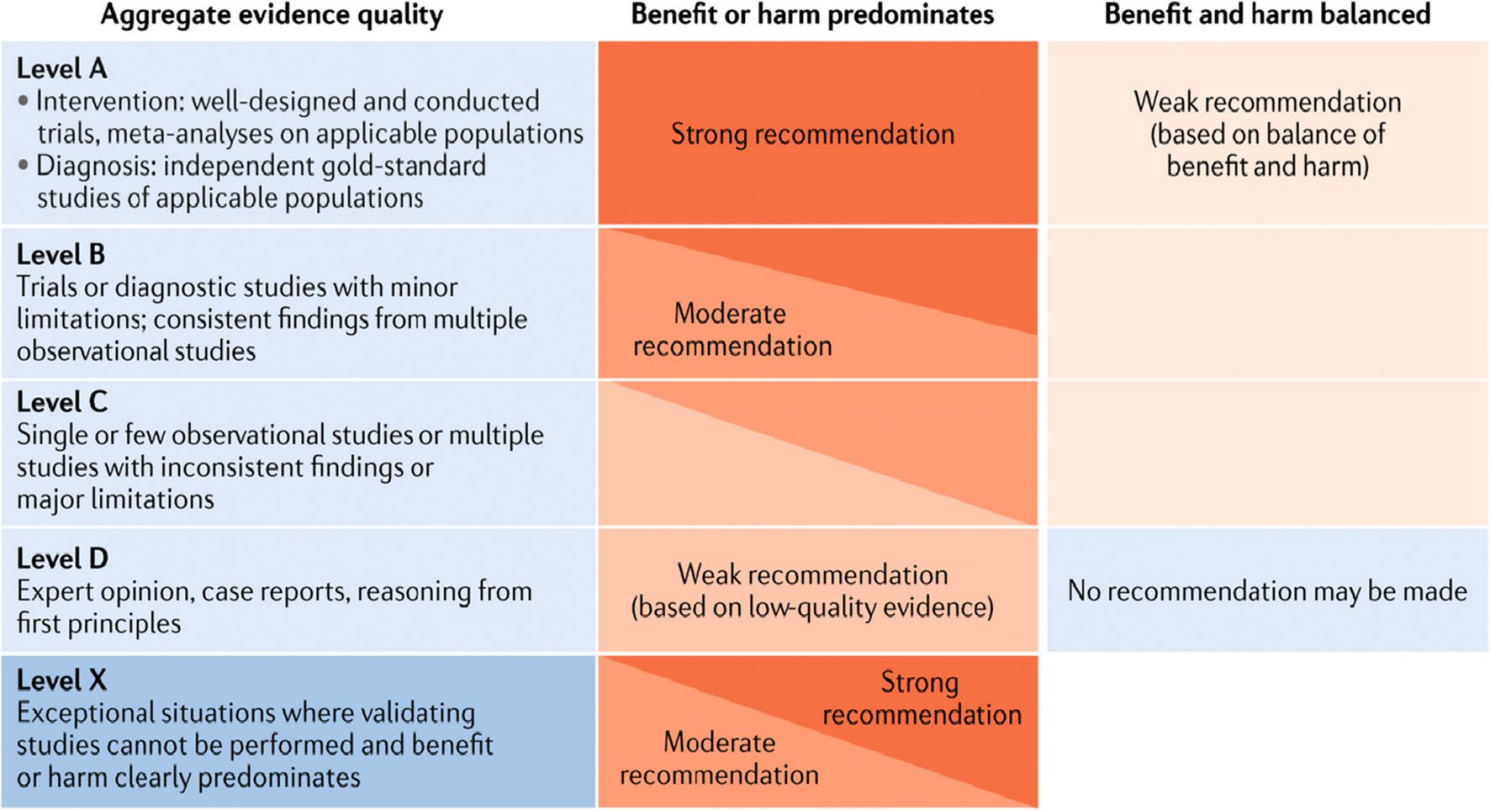
Matrix for grading of evidence and assigning strength of recommendations currently used by the American Academy of Pediatrics [22]

The American Academy of Pediatrics grading system was adopted by the IPNA Best Practice and Standards Committee [24] (Fig. 1). Each evidence statement was graded as high (A), moderate (B), low (C), very low (D), or not applicable (X) according to the system. Grade X refers to clinical situations where appropriate studies cannot be performed because benefit or harm clearly predominates; we also used it to grade clear contra-indications and safety boundaries. The strength of recommendations was also graded as strong, moderate, or weak.

### Clinical practice recommendations for IgA nephropathy (IgAN)

Recommended definitions are provided in Table 1 and in the text as appropriate.

**Table 1:** Common definitions for IgA nephropathy (IgA) and IgA vasculitis nephritis (IgAVN)

Definitions	Descriptor
IgA nephropathy (IgAN)	Dominant or co-dominant glomerular (mesangial or mesangial plus capillary wall) IgA staining on kidney biopsy and the exclusion of differential diagnoses on clinical or pathological grounds
Relapse IgAN	Reappearance of urinary protein creatinine ratio (UPCR—based on first-morning void, preferably) > 0.5 mg/mg (50 mg/mmol) or proteinuria > 500 mg/day in 24-h collection based on at least two urine samples collected 1–2 weeks apart in patients who achieved remission. Hematuria and/or reduced eGFR (< 90 mL/min/1.73 m^2^) or declining eGFR after exclusion of other causes of kidney injury can also be present ** This definition leaves a gray zone between 0.2 and 0.5 g/g (20–50 mg/mmol) or 200–500 mg/day of proteinuria. In a patient who previously achieved remission with proteinuria within this range despite optimal RASB during follow-up of IgAN, we recommend repeating proteinuria assessment three to four times within 4–8 weeks to ascertain the value of proteinuria. The prognostic significance of this level of proteinuria is uncertain, and while it may warrant optimizing conservative therapy, perhaps, it does not warrant the use of immunosuppression
Remission IgAN	Resolution of proteinuria (UPCR < 0.2 mg/mg or 20 mg/mmol) or proteinuria < 100 mg/m^2^ per day or < 0.2 g/day in 24-h collection) based on at least two urine samples collected at least 1 month apart in the presence of normal (≥ 90 mL/min/1.73 m^2^) or stable eGFR. Complete remission includes, in addition to these features, the resolution of hematuria, defined as a negative dipstick for blood and/or < 5 RBC/high-power microscopic field
Synpharyngitic hematuria	Macroscopic hematuria (i.e., red or brown urine with RBCs noted on microscopy) occurring during the course of an upper respiratory infection
Hematuria	RBCs ≥ 5/hpf, urine dipstick ≥ 1 + blood on at least two separate samples, and/or presence of dysmorphic RBCs (acanthocytes), and/or RBC casts in a fresh spot urine sample
Persistent microscopic hematuria	RBCs ≥ 5/hpf, urine dipstick ≥ 1 + blood on at least two urine samples collected at least one month apart
Orthostatic proteinuria	An elevated urine protein excretion while in the upright position only, with normal urine protein excretion in a supine position
Nephrotic-range proteinuria	UPCR ≥ 2 mg/mg (200 mg/mmol) in spot urine, or proteinuria ≥ 1000 mg/m^2^/day in a 24-h urine sample corresponding to 3 + (300–1000 mg/ dL) or 4 + (≥ 1000 mg/dL) by urine dipstick
Nephrotic syndrome	Nephrotic-range proteinuria and either hypoalbuminemia (serum albumin < 30 g/L) or edema when serum albumin is not available
IgA vasculitis (IgAV)	A systemic vasculitis with IgA-dominant immune deposits affecting small vessels (predominantly capillaries, venules, or arterioles)
IgA vasculitis with nephritis (IgAVN)	Glomerulonephritis characterized by dominant or co-dominant IgA glomerular deposition, defined by kidney involvement in a patient with IgAV, morphologically indistinguishable from IgAN
Remission of IgAVN	Resolution of proteinuria (UPCR < 0.2 mg/mg or 20 mg/mmol) or proteinuria < 100 mg/m^2^ per day or < 0.2 g/day in 24-h collection) based on at least two urine samples collected at least 1 month apart in the presence of normal (≥ 90 mL/min/1.73 m^2^) or stable eGFR. Complete remission includes, in addition to these features, the resolution of hematuria, defined as a negative dipstick for blood and/or < 5 RBC/high-power microscopic field
Relapse of IgAVN	Recurrence of hematuria (gross hematuria or ≥ 2 + in dipstick or 5 RBCs/hpf) and/or proteinuria (UPCR ≥ 0.2 mg/mg or 20 mg/mmol) on first-morning void in at least two urine samples and/or reduced kidney function (eGFR < 90 mL/min/1.73 m^2^ or > 25% reduction from baseline) in a patient who has achieved a complete remission for at least 1 month Notes: Remission and relapse refer to clinical markers and not pathologic proof of the presence or absence of disease activity **This definition leaves a gray zone between 20 and 50 mg/mmol or 0.2–0.5 g/g or 200–500 mg/day of proteinuria
Glucocorticoid toxicity (*consideration for change in treatment strategy)	New or worsening obesity/overweight, sustained hypertension, hyperglycemia, behavioral/psychiatric disorders, sleep disruption, impaired statural growth (height velocity < 25th percentile and/or height < 3rd percentile) in a child with normal growth before start of steroid treatment, Cushingoid features, striae rubrae/distensae, glaucoma, ocular cataract, bone pain, avascular necrosis
Recurrence of IgAN	Transplant kidney biopsy showing glomerular IgA deposits in a patient whose native disease was IgAN
Recurrence of IgAVN	Transplant kidney biopsy showing glomerular IgA deposits in a patient whose native disease was IgAVN
Rapidly progressive glomerulonephritis	< 50% of normal kidney function or rapid loss of renal function accompanied by crescentic lesions and necrosis in the kidney biopsy

*RASB*, renin-angiotensin system blockade; *eGFR*, estimated glomerular filtration rate; *RBC*, red blood cells; *HPF*, high-power field; *IgAN*, immunoglobulin A nephropathy; *IgAVN*, immunoglobulin A vasculitis nephritis; *UPCR*, urine protein creatinine ratio

### Initial assessment, diagnosis, and indications for kidney biopsy

- Recommendation 1a
  - We recommend obtaining a careful patient history for kidney and systemic manifestations and performing a detailed physical examination and blood and urine tests as per Table 2 in children suspected of having IgAN (grade *A*, *strong recommendation*).

**Table 2:** Initial clinical and laboratory workup and follow-up for children with IgA nephropathy. Modified from IPNA clinical practice recommendations for children with SRNS (Trautmann 2020)

Investigations	Initial workup	Follow-up monitoring
**Clinical evaluation**		
Patient history		
• Including rash, episode of gross hematuria, physical activity, fever episodes, pain, abdominal discomfort, swelling, fatigue, school attendance, etc	✓	Every 3 months
• Search for secondary causes of IgAN, such as HIV infection, hepatitis, inflammatory bowel disease, autoimmune disease, liver cirrhosis, and infection-related GN	✓	As appropriate
• Check for tuberculosis in endemic areas before starting immunosuppressant drugs	✓	As appropriate
Physical examination		
• Search for signs of edema	✓	Every 3 months
• Steroid toxicity (Cushing syndrome, cataracts, glucose intolerance, growth retardation, osteoporosis, hypertension, infection)	✓	Every 3 months for those receiving steroid therapy
• Extrarenal features, such as rashes, oral ulcers, joint swelling	✓	As appropriate
• Pubertal status: Tanner stage, testicular volume in boys (in patients aged > 10 years)	✓	Every 12 months
• Vital parameters: blood pressure	✓	Every 3 months; yearly 24-h ambulatory BP monitoring in patients with hypertension, if feasible
Anthropometry		
• Growth chart: height/length, weight	✓	Every 6 months
• Calculation of BMI and annual height velocity	✓	
**Biochemistry**		
Urine		
• First-morning urine protein–creatinine ratio or 24-h proteinuria excretion	✓	Every 3 months or more frequently
• Urine sediment for erythrocyte count and morphology	✓	
Blood		
• Complete blood count (CBC)	✓	Every 6 months (more frequently in CKD stage 4–5)
• Creatinine, BUN, or urea	✓	
• Electrolytes, serum albumin, total protein	✓	
C-reactive protein	✓	As required (clinical decision)
Estimated GFR	✓	Every 3 months (more frequently in CKD stage 4–5)
ALP, PTH, 25(OH) vitamin D	✓	Every 12 months (more frequently in patients with CKD stages 3–5)
Lipid profile (LDL- and HDL-cholesterol, triglycerides)	✓	Every 12 months or as appropriate
Baseline coagulation tests (prothrombin time (INR), aPTT, fibrinogen, ATIII), detailed thrombophilic screening in patients with reported previous thrombotic events, central venous lines, persistent nephrotic-range proteinuria, and/or increased familial history for thrombotic events	✓	At diagnosis and then as appropriate
Thyroid function (T3, FT4, TSH)	For patients with nephrotic-range proteinuria	As appropriate especially in patients with persistent nephrotic-range proteinuria
Immunoglobulin A levels, C3 circulating levels	✓	At diagnosis
Immunoglobulin G levels	✓	In case of recurrent infections
Antinuclear antibodies, dsDNA, ENA, ANCA	✓	As appropriate
HBs-Ag, anti-HCV-IgG, syphilis, HIV tests, TB tests	✓	Before steroid and as appropriate
**Imaging**		
Renal ultrasound: renal echogenicity and size of kidneys	✓	At presentation (mandatory before renal biopsy)
Ultrasound of abdomen and pleural space (ascites, effusions, thrombosis)	For patients with nephrotic-range proteinuria	As appropriate especially in patients with persistent nephrotic-range proteinuria
Cardiac ultrasound (left ventricular mass, effusions)	✓	Every 12 months in hypertensive patients or in case of severe edema
Chest X-ray	✓	If indicated
**Histopathology**		
Renal biopsy	✓	At diagnosis, and subsequently if indicated: in case of unexplained drop in eGFR, unexplained increase in proteinuria
**Dietary assessment**		
Dietician review and advice by a dietician regarding salt, potassium, caloric, and protein intake	✓	Every 3 months (more frequently in malnourished patients, and patients with CKD stages 4–5)

*GN*, glomerulonephritis; *BUN*, blood urea nitrogen; *eGFR*, estimated glomerular filtration rate; *CKD*, chronic kidney disease; *HIV*, human immunodeficiency virus; *IgAVN*, IgA vasculitis nephritis; *dsDNA*, double-stranded DNA; *ENA*, extractible nuclear antigen; *ANCA*, anti-neutrophil cytoplasm antigen; *HBs-Ag*, hepatitis B antigen; *anti-HCV-IgG*, anti-hepatitis C immunoglobulin G; *TB*, tuberculosis

#### Evidence and rationale

IgAN can present with a range of clinical features, including asymptomatic microscopic hematuria (with varying degrees of proteinuria, with or without progressive kidney disease), intra-infectious (usually accompanying upper respiratory tract infection with fever, so-called “synpharyngitic”) macroscopic hematuria, rapidly progressive glomerulonephritis, nephrotic syndrome, and acute kidney injury (AKI) [4, 10]. IgAN is a condition that is characterized by the presence of dominant or co-dominant IgA staining in glomeruli. However, similar findings can also be observed in the context of other systemic illnesses, such as IgAV. These illnesses may present with symptoms such as rashes, oral ulcers, hemoptysis, abdominal pain, and joint swelling [2, 4]. KDIGO 2021 Clinical Practice Guidelines first recommend excluding IgAV and IgAN secondary to liver diseases, viral illnesses, and inflammatory bowel disease, as well as systemic autoimmune diseases and IgA-dominant infection-related (post-infectious) glomerulonephritis (GN) before diagnosing primary IgAN [2].

In patients with IgAN, decisions for management are mainly based on the amount of proteinuria, blood pressure levels, and eGFR. Urinalysis should document hematuria and quantify albuminuria and/or proteinuria (see Table 1 for definitions). For children, 24-h urine collection is difficult to perform. Thus, clinical practice recommendations from IPNA and KDIGO 2021 recommend assessment of (ideally) first-morning urinary protein/creatinine ratio (UPCR) instead of 24-h protein excretion in children with suspected glomerular diseases [2, 25]. An important differential in children presenting with proteinuria is orthostatic proteinuria which can be excluded through the collection of first-morning void urine samples to quantify proteinuria. First-morning void urine is recommended for all children with proteinuria to exclude orthostatic proteinuria. The eGFR should be calculated using the Schwartz formula and its modifications for children [26, 27].

- Recommendation 1b
  - A kidney biopsy is required for the diagnosis of primary IgAN (grade X, strong recommendation).

- Recommendation 1c
  - We recommend considering the possibility of primary IgAN, to be confirmed by kidney biopsy, in the presence of hematuria (gross and/or microscopic) with proteinuria (urinary protein/creatinine ratio (UPCR) ≥ 0.2 mg/mg or 20 mg/mmol) persisting over 2–3 weeks in at least two measurements on clear urine 1–2 weeks apart in the absence of lower urinary tract etiologies, or features of systemic disease, with a normal serum C3 level (grade X, strong recommendation).

- Recommendation 1d
  - We suggest considering the possibility of primary IgAN, to be confirmed by kidney biopsy, in the case of persistent (> 2–3 weeks) or recurrent (> 2–3 times) gross hematuria occurring during an upper respiratory infection (as opposed to 2–3 weeks following the infection) (grade C, moderate recommendation).

#### Evidence and rationale

Often, the diagnosis of IgAN is suspected when children have abnormal urinary findings, especially in the school urinary screening program that is performed regularly in a few countries like Japan, South Korea, and Taiwan. An epidemiological survey based on urinary screening of children from Japan (*n* = 374,846) identified 37 children with IgAN with a mean age of 10.7 years [8]. Twenty-eight (75.7%) were biopsied due to an abnormal school urine screen while the remainder presented with gross hematuria; none presented with nephrotic syndrome or acute kidney injury (AKI) [8]. In a Japanese cohort of 258 children with IgAN, 62% presented with microscopic hematuria with or without asymptomatic proteinuria, 26% had macroscopic hematuria, and only 12% presented with acute nephritic syndrome or nephrotic syndrome [28]. On the other hand, in countries without a urinary screening program, most children with IgAN are identified after an episode of gross hematuria, most frequently concomitant with an infectious episode (classically an upper respiratory tract infection) [29]. In a study by the Southwest Pediatric Nephrology group from the USA (*n* = 218), 79% of children had gross hematuria at diagnosis, and 51% had proteinuria of 2 + or more on urine dipstick testing [29]. Also, a large series from Spain showed IgAN in 11.6% of 939 pediatric kidney biopsies, and macroscopic hematuria was the presenting symptom in 50.5% of these children [30].

Moreover, with the increasing availability of clinical trials and prospective registries for this condition, both requiring a histological diagnosis, a kidney biopsy is an absolute requirement for confirming a diagnosis in patients with suspected IgAN [2]. In patients with a positive family history of hematuria and CKD, or in patients with a diagnosis of “IgAN” with no response to adequate treatment, the possibility of collagen type IV mutations needs to be kept in mind, and performing genetic testing or α5(IV) chain staining on a skin/kidney biopsy specimen to explore this diagnosis is important [31–33].

- Recommendation 1e
  - We recommend performing a kidney biopsy promptly in children with persistent (> 2–3 weeks) or recurrent hematuria and nephrotic-range proteinuria (UPCR > 2 mg/mg or 200 mg/mmol) and/or reduced eGFR (< 90 mL/min/1.73 m2) (grade X, strong recommendation).
  - We recommend performing a kidney biopsy in children with persistent (> 2–3 weeks) or recurrent hematuria and UPCR > 0.5 mg/mg (50 mg/mmol) in at least two measurements on clear urine 1–2 weeks apart (grade X, moderate recommendation).
  - We suggest performing a kidney biopsy in children with persistent (> 2–3 weeks) or recurrent hematuria and UPCR between 0.2 and 0.5 mg/mg (20–50 mg/mmol) in at least three measurements on clear urine 1–2 weeks apart (grade D, weak recommendation).

- Recommendation 1f
  - We recommend considering the diseases listed in Table 3 as the major differential diagnoses of IgA nephropathy in children (grade X, strong recommendation).

**Table 3:** Major differential diagnosis of IgA nephropathy in children

Condition	Clinical presentation	Diagnosis
Thin basement membrane disease	Persistent glomerular hematuria (microscopic ± gross), minimal proteinuria	Kidney biopsy (EM) shows diffusely thin glomerular basement membranes for patient age
Alport syndrome (autosomal recessive or X-linked)	Recurrent gross hematuria, persistent microscopic hematuria with or without proteinuria and/or sensorineural hearing loss and/or ocular abnormalities (i.e., lenticonus), most often in boys	Genetic testing (mutations in *COL4A5*, *COL4A3*, or *COL4A4* genes), skin biopsy for the absence of collagen alpha-5(IV) chain in the epidermal basement membrane (X-linked), kidney biopsy (electron microscopy) shows lamellated GBM
Post-infectious glomerulonephritis	Microscopic ± gross hematuria starting 1–3 weeks after an upper respiratory infection, usually with proteinuria, often hypertension, reduced kidney function, edema	Low serum C3 levels, elevated anti-streptococcal titer (ASOT), renal biopsy shows diffuse glomerulonephritis, C3 ± IgG deposits, subepithelial “humps” by electron microscopy
Lupus nephritis	Photosensitive rash, joint pains, hematuria, proteinuria, normal or reduced kidney function, ± hypertension	ANA, anti-dsDNA positive, low serum C3 and C4 levels, renal biopsy with varying histologic findings but “full house” (IgG, IgA, IgM, C1q, C3) immune deposits
IgA vasculitis nephritis	Purpuric rash predominantly over lower limbs and trunk, ± abdominal and joint pain, hematuria often with proteinuria, normal or reduced kidney function	Clinical criteria of EULAR/PRES, normal serum C3, biopsy of skin lesions with leukocytoclastic vasculitis + IgA in dermal vessels, indistinguishable from IgA nephropathy on kidney biopsy
ANCA-associated vasculitis	Decreased kidney function, often rapidly progressive with active urine sediment and variable proteinuria, may be features of systemic vasculitis	Serology positive for anti-MPO or anti-PR3, normal serum C3, renal biopsy shows necrotizing/crescentic GN with few or no immune deposits by IF and EM

*EM*, electron microscopy; *ANA*, antinuclear antibody; *EULAR/PRES*, European League Anti-Rheumatic Disease/Pediatric Rheumatology European Society

##### Explanatory note on kidney biopsy

In order to make a diagnosis of primary IgAN, a kidney biopsy that is processed for, at minimum, light microscopy and immunofluorescence (IF)/immunohistochemistry (IHC) for immunoglobulins (IgG, IgA, IgM) is required. Additional staining for C3, C1q, and C4 (all complement components); fibrin; and kappa and lambda light chains is recommended as per individual center practices. A diagnosis of primary IgAN requires dominant or co-dominant IgA glomerular staining among immunoglobulins, and the exclusion of differential diagnoses for clinical (IgAVN, secondary IgAN) and/or pathologic (e.g., IgA-dominant post-infectious GN) reasons (Supplementary Table S4). Electron microscopy is not required for diagnosis but is helpful in excluding hereditary basement membrane abnormalities and IgA-dominant post-infectious GN that is beyond the acute phase.

#### Evidence and rationale

**Fig. 2:**
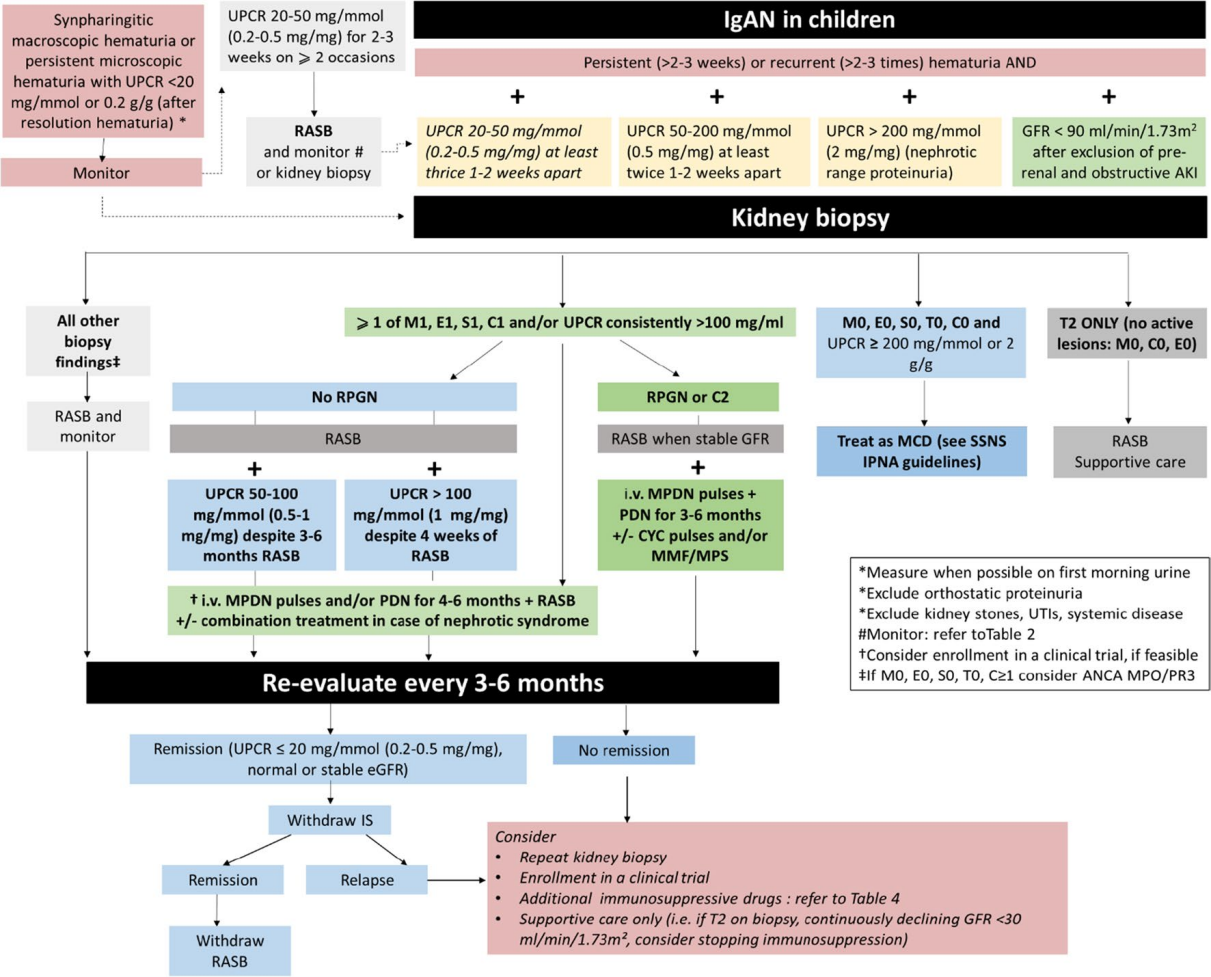
Management algorithm for IgA nephropathy

After the exclusion of lower urinary tract etiologies (i.e., hypercalciuria, hypocitraturia, kidney stones, urinary tract infections), the main differential diagnoses to exclude in children with isolated, recurrent, or persistent hematuria are listed in Table 3. While some of these conditions can be distinguished from IgAN based on clinical and serologic findings, a kidney biopsy is needed to establish a definite diagnosis of IgAN and is often important for guiding therapy and assessing prognosis. A possible exception is X-linked Alport syndrome where a skin biopsy (with immunofluorescence studies for alpha-5 chains of type IV collagen) may often suffice, and all forms of Alport syndrome where a genetic study of *COL4* mutations can provide a definitive diagnosis, especially if there is an appropriate family history [32, 33]. A recent history of potential post-infectious nephritis should also be sought. Figure 2 shows a flowchart for the diagnosis and management of IgAN for use in clinical practice.

The use of immunosuppressive drugs, including glucocorticoids, before a kidney biopsy is not recommended unless a rapidly declining GFR suggesting rapidly progressive glomerulonephritis (see Recommendation 4b) is present, in which case it must be performed as soon as possible. The use of renin-angiotensin system blockers (RASB) to reduce proteinuria may be concomitant but should not delay performing a kidney biopsy. There is no single laboratory test or group of tests specific for IgAN. For assessment of kidney pathology, the KDIGO guidelines for adequacy of specimen and processing should be followed [2]. While the site of IgA deposits is best delineated by electron microscopy, a diagnosis of IgAN can be made based on light microscopy and IF/IHC findings alone.

Biopsies should be routinely classified according to the Oxford MEST-C classification (mesangial [M] and endocapillary [E] hypercellularity, segmental glomerulosclerosis [S], interstitial fibrosis/tubular atrophy [T], and crescents [C]). Classification categories are provided in Supplementary Table S5 [34]. While many studies have validated the use of this classification in adults, fewer studies in pediatric populations validate its use in this group with more variable results, likely related to smaller sample sizes and fewer patients reaching study endpoints (Supplementary Table S6) [35–39]. While mesangial, endocapillary, and extracapillary hypercellularity are common biopsy features in childhood IgAN, tubular atrophy/interstitial fibrosis, which is strongly associated with the development of kidney failure, is less frequent than in adults [40].

One study evaluating the causes of microscopic and gross hematuria in children from Thailand showed that among 342 children with microscopic hematuria, no cause was found in 276 children, and of the remaining 66, 16% had hypercalciuria while post-infectious (infection-related) GN was diagnosed in 1% [41]. Of those presenting with gross hematuria (*n* = 228), no cause was found in 86 patients while 22% had hypercalciuria and 15.7% were diagnosed with IgAN on biopsy [41]. In a retrospective review of 100 patients referred for gross hematuria (during 1992–1999), complete data was reported for 82 children; 13 (15.9%) were diagnosed with IgAN while 6 (7.3%) had thin basement membrane disease among those with glomerular hematuria (*n* = 24) [42]. A recent study identified 31% of patients with IgAN and thin basement membrane pathogenic variants in *COL4A3/COL4A4/COL4A5* genes [43, 44]. Among those with non-glomerular hematuria (*n* = 56), 9 (16.1%) children had hypercalciuria [43]. A recent study observing 28 children with IgAN longitudinally supports the importance of kidney biopsy even in patients presenting with low levels of proteinuria [45].

Regarding children with recurrent macrohematuria and IgAN, evidence in children confirms the findings from adults that this clinical feature is accompanied by a better prognosis compared to patients with a single episode of isolated macrohematuria and to patients without hematuria [46]. However, persistent intense macrohematuria can occasionally, in IgAN patients as in other settings, be accompanied by AKI due to tubular necrosis. This is reported mainly in adult older patients, and a single pediatric report showed spontaneous resolution [47].

### Clinical and laboratory workup, frequency of follow-up

- Recommendation 2a
  - We recommend long-term follow-up in children with IgAN, including those achieving complete remission, as they can relapse after many years (grade X, strong recommendation).

- Recommendation 2b
  - We recommend adjusting follow-up intervals based on the severity of clinical symptoms, histopathological features, the treatment regimen, and the response to treatment (Table 2) (grade X, strong recommendation).

- Recommendation 2c
  - We suggest re-evaluating the etiology of the disease to exclude secondary IgAN for children with persistent proteinuria (UPCR ≥ 0.2 mg/mg or 20 mg/mmol) after 3–6 months of supportive treatment (grade X, moderate recommendation).

- Recommendation 2d
  - We recommend lifelong ongoing yearly monitoring of blood pressure and urinalysis for patients with a history of pediatric IgAN (grade X, strong recommendation).

- Recommendation 2e
  - We recommend, upon diagnosis, offering the patient and his/her family psychological and social support, to be optimized on a case-by-case basis.

#### Evidence and rationale

There is no robust evidence for the follow-up protocols of IgAN to improve kidney prognosis. Risk factors for disease progression include ongoing high levels of proteinuria, ongoing hematuria, hypertension during follow-up, decreased kidney function at the time of biopsy, and the histopathological grading [48]. Hypertension was a strong risk factor for IgAN progression. More severe histologic lesions are often found together with hypertension, higher amount of protein in the urine, and decreased eGFR at the end of follow-up.

Urinary findings can dynamically reflect the severity of disease in both adults and children with IgAN. Sustained proteinuria of 1 g/day was strongly associated with the progression of chronic kidney disease and with the development of kidney failure in IgAN in patients aged 15.6–73.5 years [48, 49]. Each gram per day increase above 1 is associated with a 10 to 25 times more rapid decrease in kidney function [49]. Remission of proteinuria might imply a reduced risk of progression to kidney failure, and proteinuria reduction early in treatment was associated with a lower risk for poor outcome, defined as the composite of time to the first occurrence of doubling of serum creatinine, kidney failure, or death [50, 51]. Based on more recent data from the RaDaR study, even lower levels of proteinuria (0.5–1 g/day in adults) appear to be associated with an increased risk of kidney disease progression [14]. In terms of hematuria, the lack of a validated and reproducible assessment modality has hindered studies on its prognostic value. Some single-center data suggest that remission of hematuria is associated with better kidney survival in IgAN [48, 52]. Microscopic hematuria may be an important risk factor for IgAN progression, as suggested by several studies recently reviewed and highlighted [53, 54]. However, recently, isolated recurrent macrohematuria has been shown to be a benign prognostic factor [46]. Regular urinalysis, documenting the presence and amount of proteinuria and hematuria, should be considered routine care for children with IgAN.

The amount of proteinuria, eGFR, blood pressure, pathological findings, and treatment regimen are used as a guideline for setting the regular follow-up interval. Close follow-up is required to detect eGFR decline among patients with IgAN at high risk of progression. Changes in kidney function should be monitored carefully when RASB are initially administered, and infectious risk should be monitored in patients receiving glucocorticoid or other immunosuppressive agents.

One study evaluating 96 pediatric IgAN patients with focal mesangial proliferation who were not treated showed that 57 (59.4%) achieved spontaneous remission of proteinuria and hematuria within a mean time of 5.9 years [55]. Of the 57 patients with spontaneous remission, ten (17.5%) recurred with urinary abnormalities. Maintenance of remission was observed in 79.9% and 67.9% at 5 and 10 years, respectively, after first remission. Severe clinical signs usually develop after 5 to 15 years [56]. Approximately 70% of patients who had childhood-onset IgAN had kidney symptoms even after approximately 20 years of follow-up evaluation [56]. A retrospective cohort study of 1012 patients (mean age of 32 ± 12.0 years) with IgAN at a single center in Japan showed that about 50% of patients progressed to kidney failure within 30 years despite treatment [57]. IgAN was falsely believed to be benign until one study in the 1990s reported that about 40% of IgAN patients progressed to kidney failure within 20 years [58, 59]. In a study of 241 Japanese children, it was reported that 5% of the patients had developed CKD by 5 years from the onset of the disease, 6% by 10 years, and 11% by 15 years [60]. Therefore, long-term follow-up is recommended in children with IgAN, even in cases with mild kidney dysfunction and mild proteinuria or in cases of complete remission, as urinary abnormalities may reappear and kidney failure may progress [2]. This has recently been confirmed by large-scale epidemiological studies evaluating adult risk of CKD in individuals presenting with different forms of kidney impairment, including glomerular diseases, in childhood [61, 62]. For this same reason, adequate psychological and social support is of paramount importance.

Some authors classify patients into low-risk groups and moderate-to-high-risk groups based on proteinuria, eGFR, and blood pressure, and suggest the follow-up interval should be every 6–12 months for at least 10 years for the low-risk group [63, 64]. The adult International IgAN Prediction Tool from the International IgA Nephropathy Network was recently updated for children, exploring an international cohort of 1060 children and concluding that this tool can also be used to assess the risk of progression in pediatric forms of IgAN [64].

### Treatment and management of biopsy-proven IgAN

- Recommendation 3a
  - In patients with UPCR ≥ 0.2 mg/mg (20 mg/mmol), we recommend optimizing supportive care by (1) providing dietary counseling to moderate salt intake below 3–5 g/day (level X, strong recommendation) and (2) prescribing treatment with RASB either as ACE-inhibitor (ACEi) or angiotensin receptor blocker (ARB) at maximally tolerated doses aiming for UPCR < 0.2 mg/mg (20 mg/mmol) (grade C, moderate recommendation).
  - In children with CKD stage 2 or higher, we recommend targeting 24-h mean arterial BP ≤ 50th percentile or targeting ≤ 75th percentile for age, sex, and height by ambulatory BP monitoring in those presenting with proteinuria (UPCR > 0.5 mg/mg or 50 mg/mmol) or no proteinuria, respectively (grade B, moderate recommendation).
  - We also recommend regular aerobic exercise, targeting the ideal body weight, and no smoking or vaping (grade X, strong recommendation).
  - We recommend counseling children and parents regarding the increased risk of progression of kidney disease associated with being overweight and obese, including nutritional counseling, if available (grade X, moderate recommendation).
  - We do not recommend the introduction of any specific treatment in children with no risk for progression, e.g., patients with isolated microscopic hematuria or gross hematuria completely resolved without any persistent urinary abnormality (grade B, moderate recommendation).
  - We recommend regular re-evaluation since the risk may change during follow-up (grade X, strong recommendation).

#### Evidence and rationale

*RASB (renin-angiotensin system blockers)*: The target 24-h mean arterial pressure (measured by ambulatory blood pressure monitoring) for children with IgAN, as for any child with hypertensive CKD, is ≤ 75th percentile or ≤ 50th percentile for age, sex, and height in the presence of proteinuria (UPCR > 0.5 mg/mg or 50 mg/mmol) or in the absence of proteinuria, respectively [2, 65]. The KDIGO 2021 guidelines strongly recommend RASB (ACEi or ARB) as initial therapy for adults with IgAN and proteinuria > 0.5 g/day, based on moderate-grade evidence [2]. However, there is insufficient data currently to recommend that children be managed in the same way as adults with IgAN, and the threshold level of proteinuria to be considered for initiating RASB inhibition has not been proven by RCTs [2, 66].

#### Pediatric data

Coppo et al. conducted an RCT on the effects of ACEi (benazepril) over 35 months in 66 pediatric and young adult IgAN patients (9–35 years old) with baseline proteinuria of 1–3.5 g/day/1.73 m^2^ and creatinine clearance > 50 mL/min/1.73 m^2^ [67]. In the placebo group, creatinine clearance decreased by more than 30% in 14.7% of patients, compared with 3.1% in the benazepril group. Partial or complete remission of proteinuria was detected in 40.6% and 12.5%, respectively, of patients on benazepril versus 8.8% and 0% on placebo. These results supported the benefits of ACEi in young patients with proteinuria > 1 g/day/1.73 m^2^.

Nakanishi et al. conducted a prospective, single-arm study administering lisinopril without comparators in 40 children with IgAN showing focal mesangial proliferation [68]. After 2 years of treatment, proteinuria levels were significantly lower than baseline. Adverse events of dizziness occurred in 12.5% of children and resolved with dose reduction, never needing treatment discontinuation. These data indicated that lisinopril was efficacious and safe in the treatment of children with mild IgAN.

Shima et al. conducted an RCT on children with IgAN and focal mesangial proliferation treated with lisinopril alone or with lisinopril and losartan in combination for 2 years [69]. At the end of the study, the two groups showed similar frequency of complete remission of proteinuria (89.0% and 89.3%). The incidence of dizziness was higher in the combination group, but no differences in other adverse events were reported. Furthermore, there was no significant difference in mesangial proliferation and improvement of crescent formation, suggesting the recommendation of monotherapy with lisinopril in children with mild IgAN.

There are few studies on the long-term outcome of pediatric IgAN treated with RASB. Higa et al. retrospectively evaluated 32 pediatric IgAN patients with proteinuria of 0.5 g/day/1.73 m^2^ or less, treated with RAS inhibitors, and found a 100% kidney survival at 15 years [70].

Based on the above evidence, we recommend the use of RASB in children with IgAN. Considering that any level of persistent proteinuria is a risk factor for progression to kidney failure in patients with IgAN [71, 72], all patients presenting with persistent proteinuria are eligible for RASB administration. Normal urinary protein excretion level is UPCR < 0.2 mg/mg (20 mg/mmol); therefore, RAS inhibitors are recommended in patients showing persistent UPCR ≥ 0.2 mg/mg (20 mg/mmol) [2]. However, no pediatric RCT is available to support the value of this threshold of proteinuria to initiate the treatment.

If a decrease in blood pressure, a decrease in eGFR, or an increase in serum potassium is observed after starting treatment with a RASB, dose reduction or discontinuation should be considered. Administration of RASB is absolutely contraindicated in women who are pregnant or may become pregnant because ACEi/ARB fetopathy (severe amniotic fluid deprivation, lung hypoplasia, kidney failure, and limb contracture) can occur with the use of RASB [2].

The dose, duration, and side effects of these drugs in children with IgAN are based on clinical trials and observational studies summarized in Supplementary Tables S2 and S3. However, there is a lack of evidence regarding the timing and criteria for initiating this treatment. Moreover, most studies used a combination of RASB and other drugs; hence, the evaluation of the effects of RASB alone is often difficult. The identification of the criteria to decide during follow-up that RASB alone is not sufficient and other drugs must be considered has not been supported by ad hoc designed clinical trials. In addition, there are a minority of pediatric cases in which RASB alone would not be suggested at the time of diagnosis, as detailed in other sections.

If UPCR < 0.5 mg/mg (50 mg/mmol) is maintained with RASB monotherapy, continuation of treatment for 2 years is considered acceptable [70]. On the other hand, if UPCR ≥ 1.0 mg/mg (100 mg/mmol) is observed after 3 to 6 months of RASB monotherapy, a change in treatment strategy to immunosuppressive therapy should be considered.

##### Diet, body weight, and exercise

Weight reduction in a group of obese adults with IgAN resulted in a decrease in proteinuria, with a significant correlation between weight loss and the degree of decrease in proteinuria [73]. A single-center, prospective RCT in Thailand in 26 overweight adult patients with IgAN with proteinuria showed that a 6-month low-calorie diet resulted in a significant decrease in proteinuria accompanied by a reduction in weight, fat content, and leptin levels [74]. An evaluation of the 10-year kidney survival in 43 adult patients with IgAN showed that a BMI ≥ 25 kg/m^2^ was associated with a ≥ 1.5-fold increase in serum creatinine [75]. Although there are no specific studies on children, knowing the deleterious effects of obesity on the cardiovascular system and kidney function, it is recommended that children with IgAN and their parents should be encouraged to maintain a healthy lifestyle, prevent overweight, and decrease weight if already overweight or obese with a moderate sodium intake (max 3–5 g/day), avoidance of protein powder or formulations, avoidance of smoking, and practicing regular aerobic exercise as key importance in this patient population [2, 76].

- Recommendation 3b
  - We do not recommend the routine use of glucocorticoids in children with IgAN (grade X, strong recommendation).
  - We suggest considering treatment with glucocorticoids in selected settings (refer to evidence text below) (grade C, moderate recommendation).

#### Evidence and rationale

*Glucocorticoids*: Based on the results of randomized controlled trials in adults, the KDIGO Glomerulonephritis 2021 Guideline committee suggested that if proteinuria > 1 g/day persisted unchanged after 3–6 months of RASB, treatment with glucocorticoids should be considered for 6 months in both children and adults [77–80]. Protocols reviewed included either intravenous pulses of methylprednisolone (3 times, 1 g each) on months 1, 3, and 5 and oral prednisone (0.5 mg/kg on alternate days), or oral prednisone 0.8–1 mg/kg/day for 2 months, tapering in 6 months [78, 80].

#### Adult data

Subsequent trials in adults provided caution with respect to the use of glucocorticoids. The STOP-IgAN trial failed to demonstrate superior efficacy of immunosuppressive therapy (monotherapy with methylprednisolone pulses for 6 months in patients with eGFR > 60 mL/min/1.73 m^2^; oral prednisone and cyclophosphamide followed by azathioprine in patients with lower eGFR) versus supportive care for 3 years [81]. The lack of long-term benefits was confirmed over a 10-year follow-up [82]. The Therapeutic Evaluation of Steroids in IgA Nephropathy Global (TESTING) RCT compared oral methylprednisolone (0.6–0.8 mg/kg/day weaning and discontinued after 6–9 months) versus placebo [80]. Serious adverse events (mostly infections, including two deaths) in patients receiving glucocorticoids led to discontinuation of recruitment after 2 years, leading to the inability to draw conclusions (Table 4). Therefore, the KDIGO 2021 Glomerulonephritis Guideline committee suggested to use glucocorticoids for 6 months in IgAN patients with persistent proteinuria > 1 g/day despite maximal supportive care (including low sodium diet and maximally tolerated doses of RASB for 3–6 months) only after careful evaluation of the risk–benefit profile of glucocorticoid toxicity, and preferably to enroll the patient in a clinical trial [2].

**Table 4:** Dose, monitoring, and adverse effects of main agents used to treat IgAVN

Therapeutic agent Dose	Monitoring	Adverse effects
**Oral glucocorticoids:** Prednisolone or prednisone (PDN) 1–2 mg/kg/day (maximum dose 60 mg) for 4 weeks then alternate-day dosing tapered over 3–6 months IV glucocorticoids pulses: methylprednisolone, three doses given daily for 3 days at induction, 500 mg/m^2^ per dose (maximum dose 1000 mg). May be repeated	Quarterly: blood pressure, height, weight Yearly: ophthalmological examination	Obesity/weight gain, hypertension, diabetes mellitus, behavioral/psychiatric disorders, sleep disruption, growth failure, cushingoid features, striae rubrae/distensae, glaucoma, cataract, bone pain, and avascular necrosis
**Mizoribine:** Purine antimetabolite mainly available in Japan 4 mg/kg/day (maximum dose 150 mg)	Initially monthly, later quarterly: CBC Uric acid	Hyperuricemia Fertile females must be warned of the need to avoid unplanned pregnancy (mizoribine can cause fetal malformations)
**Mycophenolate mofetil (MMF)/ mycophenolic sodium (MPS**): **MMF:** 1200 mg/m^2^ per day in two divided doses every 12 h (usually start at 600 mg/m^2^ in two doses for the first week to improve tolerance) **MPS:** 360 mg corresponds to 500 mg of MMF Therapeutic drug monitoring using a limited sampling strategy: The most effective MPA AUC is above 50 mg × h/L	Quarterly: CBC LFTs	Abdominal pain, diarrhea, weight loss (may be improved by the use of MPS) Leukopenia, anemia, and abnormal LFTs Verrucae Alopecia (infrequent) Fertile females must be warned of the need to avoid unplanned pregnancy (MMF/MPS can cause fetal malformations)
**Calcineurin inhibitors:** **Cyclosporine** Start: 3–5 mg/kg per day (maximum dose 250 mg) in two divided doses Target: C_0_ 60–100 ng/mL *or* C_2_ 300 two 550 ng/mL (aiming for the lowest possible dose to maintain remission) **Tacrolimus** Start: 0.1–0.2 mg/kg per day (maximum dose 10 mg) in two divided doses Target: C_0_ level between 3 and 7 ng/mL (aiming for the lowest possible dose to maintain remission)	Quarterly: Blood pressure CBC, creatinine, eGFR, K^+^ LFTs, lipids Uric acid (CsA) Mg^+^ (TAC) Fasting glucose (TAC) Drug levels Consider discontinuation or a kidney biopsy after 2–3 years to avoid/detect toxicity	Acute and chronic nephrotoxicity Hypertension Seizures, tremor, posterior reversible encephalopathy syndrome (PRES) Hirsutism (CsA), gum hyperplasia (CsA), diabetes mellitus (TAC) TAC drug levels can increase in case of intense diarrhea Consider risk of toxicity due to drug interactions (e.g., macrolide antibiotics, certain anti-epileptic agents, and grapefruit juice increase drug levels)
**Cyclophosphamide:** IV pulses of 500 mg/1.73 m^2^ every 2 weeks for a maximum of six doses No more than two courses (max TCD 168 mg/kg)	CBC and urine culture every 14 days during therapy	Leukopenia, severe infections Alopecia, nail discoloration Seizures Infertility GI upset (abdominal pain, diarrhea) Hemorrhagic cystitis Jaundice Fertile individuals must be warned of the need to avoid unplanned pregnancy (CYC can cause fetal malformation)

*CBC*, complete blood count; *LFT*, liver function tests; *CsA*, cyclosporine; *TAC*, tacrolimus; *eGFR*, estimated glomerular filtration rate; *K*^+^, potassium; *Mg*, magnesium; *CYC*, cyclophosphamide

The final TESTING trial (2022) reported the results after methylprednisolone dose reduction (0.4 mg/kg/day, weaning over 6 months) and anti-pneumocystis prophylaxis. Combining the results of high and reduced methylprednisolone groups, benefits to all endpoints after 6 months of treatment were observed. However, serious adverse events and particularly infections were reported mostly, but not exclusively, in patients at higher doses of glucocorticoids [83].

#### Pediatric data

In children, evidence for treatment with glucocorticoids is scarce for IgAN [56]. Studies are limited by small sample size, variable baseline measurements, and different treatment protocols, leading to heterogeneity in results. The most robust and reliable estimates of treatment effectiveness were reported in severe cases with crescentic IgAN successfully treated with pulse glucocorticoid therapy [84].

Two small studies (Welch 1992 and Waldo 1993) were published 30 years ago [85, 86]. Both studies had a short follow-up and did not use RASB or supportive care. In children with IgAN, no prospective RCT with glucocorticoids versus supportive care including RASB is available. One RCT conducted in the United States on adolescents and young adults evaluating prednisone (60 mg/m^2^ every other day for 3 months, then 40 mg/m^2^ every other day for 9 months, then 30 mg/m^2^ every other day for 12 months) compared to omega-3 fatty acids (fish oil) (4 g/day for 2 years) failed to demonstrate the benefit of prednisone [87]. The limitations of this study included a short follow-up period, differing baseline proteinuria, and a small sample size, limiting definitive conclusions.

An RCT, conducted in Japan, compared the efficacy of glucocorticoids in combination with other drugs in children with IgAN, enrolling children with severe mesangial proliferation [88, 89]. The intervention arm, receiving prednisone, azathioprine, heparin-warfarin, and dipyridamole, and the control arm, receiving heparin-warfarin and dipyridamole, were compared over 2 years. A significant improvement, in terms of proteinuria, serum IgA concentration, mesangial IgA deposition, and prevention of glomerular sclerosis, was found in the intervention group. A long-term (10 years) follow-up showed that those in the intervention arm, despite no additional treatment, were less likely to reach the endpoint of eGFR < 60 mL/min/1.73 m^2^. The 10-year kidney survival probability of each group was 97.1% (95% CI, 81.4 to 99.6%) and 84.8% (95% CI, 55.4 to 95.5%), respectively [90]. One more study examining a similar combination therapy demonstrated the disappearance of IgA mesangial deposits after 2 years of treatment [91]. In addition, a more recent retrospective study from Japan examined the use of tonsillectomy plus methylprednisolone pulses in a group of children (*n* = 18) as initial treatment and in another group (*n* = 15) as rescue treatment [92]. In both groups, a significant reduction in urinary abnormalities was observed post-treatment. Moreover, this study demonstrated substantial improvements in glomerular inflammation, including mesangial, endocapillary, and extracapillary proliferation, and highlighted a significant reduction in the proportion of segmental sclerosis and glomerulosclerosis upon second-look biopsy, emphasizing the potential benefits of this therapeutic approach in pediatric Japanese patients [92].

In France, investigators conducted a retrospective study of children with severe clinical presentation and acute histologic features of IgAN treated with glucocorticoids (9 with added cyclophosphamide) and RASB, or RASB alone [93]. The groups had different levels of proteinuria at baseline and different pathological features. However, a benefit of glucocorticoid/immunosuppressive therapy was reported, with improvement in eGFR and a decrease in proteinuria after a follow-up of 6 months. Estimated GFR in the immunosuppressive group significantly improved between onset and 6 months after treatment from 89.9 (61.2–114.5) to 110.5 (93.7–120) mL/min/1.73 m^2^, *P* < 0.001. Proteinuria also changed from 1.6 (1–4.3) at baseline to 0.3 (0.2–0.7) g/g, *P* < 0.001, after treatment with immunosuppression, while both parameters remained stable in the children treated with RASB alone.

In conclusion, although data from RCTs in children with IgAN are available, they are scant and often inconclusive due to heterogeneity of baseline risk factors in the enrolled cohorts and short follow-up.

Presently, a general approach to risk assessment in children with IgAN is based on clinical and pathology features including proteinuria, mesangial proliferation, crescents, glomerular sclerosis, or interstitial lesions; however, no cut-offs have been identified in children, besides extensive crescents involving > 50% of glomeruli. Persistent proteinuria > 0.5 g/day/1.73 m^2^ is a generally accepted threshold for starting glucocorticoids, and it is adopted by KDIGO. However, lower levels of proteinuria (> 0.3 g/day/1.73 m^2^) may be a risk factor over long follow-up and the persistence of low levels of proteinuria between 0.2 and 0.5 mg/mg (20–50 mg/mmol) despite RASB treatment is a discussed reason for using additional treatment [70]. The results from new ongoing trials in adults, if confirmed in children, will provide useful advice for halting long-term progression in children with moderate-activity IgAN. However, differences between children and adults with IgAN should be considered, mainly the more frequent finding of intense inflammatory lesions with little to no sclerosis in kidney biopsies of children with IgAN, which potentially may be more prone to respond to glucocorticoids [10, 48]. Additionally, significant differences in the early glomerular lesions of IgAN between children and adults have been demonstrated [40, 94]. Glomerular hypercellularity in the mesangial area is prominent in children and significantly greater than in adults. In contrast, glomerular matrix expansion, crescent formation, and interstitial damage are more severe in adults compared to children. Glomerular hypercellularity correlated with proteinuria in children but not in adults, whereas glomerular matrix correlated with proteinuria and kidney function in adults but not in children [94]. These differences in the pathology of IgAN in children compared to adults explain how, in pediatric clinical studies, the proteinuria-reducing effect of glucocorticoids correlates well with improvement in histologic acute lesions and supports the use of glucocorticoids in pediatric IgAN [88, 89, 92]. Therefore, although high-quality RCTs and long-term follow-up studies are lacking in the pediatric IgAN population, indications for the use of glucocorticoids may differ between children and adults.

Overall, pediatric nephrologists often choose to treat children with IgAN with glucocorticoids in the presence of proteinuria > 0.5 mg/mg or 50 mg/mmol and active histological lesions, for fear of missing the reversible phase of the disease and not preventing the subsequent sclerotic progress. In children with proteinuria > 1 g/day or UPCR > 1 g/g (100 mg/mmol) and/or mesangial hypercellularity, glucocorticoids are often prescribed in addition to RAS blockade from the time of diagnosis [2]. Although we should refrain from treatments not based on evidence, and from uncontrolled expert opinion, the attempt to limit the progression of IgAN should be considered case by case [95]. Although children tend to tolerate short cycles of glucocorticoids better than adults, possible treatment-related side effects must be discussed with patients and caregivers, as well as the possibility of entering a trial with new experimental drugs.

The IPNA IgAN guideline committee was unable to draw any evidence-based conclusions on glucocorticoid treatment for IgAN in children, either regarding the selection of patients who will benefit from this treatment or regarding the dose and duration of glucocorticoid treatment. Based on consensus methods and iterative discussion and voting, the expert group has prepared the following suggestions:

1. In children with clinical risk of progression, i.e., those with:

- UPCR 0.5–1 mg/mg (50–100 mg/mmol) despite 3–6 months of RASB
- UPCR > 1 mg/mg (100 mg/mmol) despite 4 weeks of RASB
- Active MEST-C scores (1 or more of the following scores: M1, E1, S1 with podocyte lesions, C1) and/or UPCR consistently (i.e., persisting over 2–3 weeks in at least two measurements 1–2 weeks apart) > 1 mg/mg (100 mg/mmol)

A 4–6-month course of glucocorticoid treatment should be considered, either as oral prednisolone or intravenous pulses of methylprednisolone. In these children, glucocorticoids can be dosed as follows: conventional pediatric protocol for glucocorticoids of 2 mg/kg/day (max 60 mg/m^2^/day) of oral prednisone/prednisolone (or equivalent) for a maximum of 4 weeks followed by alternate-day dosing tapered over 5–6 months. Further extension of the duration may be useful in some cases. Lower doses as emerging from the adult TESTING trial (0.4 mg/kg/day of prednisone/prednisolone (or equivalent) for 2 months, tapering over 6 months) should be considered [83].

2) A methylprednisolone pulse regimen may be selected and modulated on an individual basis in patients with higher clinical and histological risk for progression, such as:

- Children with acute onset of IgAN and declining kidney function (eGFR < 90 mL/min/1.73 m.^2^) and/or PCR > 1 mg/mg (100 mg/mmol) with active severe MEST-C scores (2 or more of the following scores: M1, E1, S1 with podocyte lesions, C1)
- Children with crescentic forms of IgAN (C2). In cases with C2, irrespective of proteinuria, the use of i.v. glucocorticoids is suggested.

In these children, glucocorticoids can be used as follows: 3 methylprednisolone i.v. pulses given at the dose of 15 mg/kg/day each (max dose, 500 mg/dose) on 3 consecutive or alternate days followed by oral prednisone/prednisolone as indicated above. Alternatively, the i.v. pulses can be repeated three times at 2-month intervals, with oral prednisone/prednisolone given at 0.5 mg/kg/day for 2 months between pulse cycles, for a total of 6 months [77, 96].

The risk–benefit balance of glucocorticoid side effects must be considered.

1. In selected cases, the use of glucocorticoids can be considered concomitantly with RASB, particularly in the presence of nephrotic-range proteinuria, reduced eGFR, and crescentic or actively inflammatory histology.
2. Consider avoiding glucocorticoids when tubulo-interstitial scarring exceeds 50% of the cortex (T2) in the absence of active lesions.
3. The target of treatment is to reduce proteinuria to < 0.2 mg/mg (20 mg/mmol).
4. If there is no response within 3–6 months, consider re-biopsy and modifying treatment.

### Use of other immunosuppressive drugs in children with IgAN

The available data do not support an evidence-based recommendation on the use of other immunosuppressive drugs in children with IgAN. Recommendations presented here are based on consensus within the expert panel.

- Recommendation 3c
  - We suggest considering the use of additional immunosuppressive drugs in combination with glucocorticoids in selected settings (grade C, weak recommendation).
    - We recommend combination treatment in cases of rapidly progressive IgAN with acutely decreased eGFR using the treatment regimen proposed in the 2021 KDIGO Glomerular Disease Clinical Practice Guideline for adults for management of ANCA-associated vasculitis (see section below on IgAN atypical forms) (grade B, moderate recommendation).
    - We recommend combination treatment in cases of IgAN with nephrotic syndrome in case of failure of glucocorticoids alone (grade B, moderate recommendation).
    - In children with IgAN not benefiting from adequate diet, RASB, and glucocorticoids alone, we recommend, when available, enrollment in clinical trials.

#### Evidence and rationale

*Combined glucocorticoids and additional immunosuppressive drugs:* the KDIGO 2021 Glomerulonephritis Guideline reports that there is limited evidence for the additional use of non-glucocorticoid immunosuppressants. However, it suggests that the additive benefit of these drugs may be considered in more severe cases [2]. For example, children with nephrotic syndrome and histological features of minimal change disease (MCD) associated with IgAN should be treated as MCD [2]. Moreover, IgAN with a rapidly progressive decline in kidney function (> 50% reduction in eGFR in < 3 months), irrespective of the percentage of glomeruli with crescents, may benefit from glucocorticoids and cyclophosphamide, as in the treatment of kidney involvement of ANCA vasculitis [2].

*Cyclophosphamide*: The benefit of cyclophosphamide in combination with glucocorticoids was reported in several case series of adults with active disease and also in a small RCT (162 adults with progressive IgAN) [97]. The STOP-IgAN study in adults, however, did not lend additional support to these data [81].

*Azathioprine and mizoribine*: Azathioprine was not found to be of additional value in adults with IgAN receiving i.v. glucocorticoid pulses and increased the risk of adverse events [98]. Although azathioprine may be beneficial in Japanese children in addition to prednisone, it required frequent discontinuation, and mizoribine was used instead of azathioprine with improved tolerability [88, 89, 99, 100]. However, mizoribine is not available in many countries.

*Mycophenolate mofetil*: Several small trials in adults reported conflicting results. Among Asian adult patients, there may be a small steroid-sparing effect [101]. However, an RCT that included children and young adults investigating the effects of glucocorticoids, mycophenolate mofetil, and fish oil was prematurely terminated due to a lack of benefit [102]. Overall, the 2021 KDIGO Glomerulonephritis Guideline does not recommend mycophenolate mofetil in IgAN, due to the lack of trial evidence. After its publication, however, evidence of suggested benefit has been reported in a large prospective adult cohort of Chinese patients and in a smaller Korean prospective study [103, 104].

*Rituximab*: A small RCT of 34 adult patients that compared rituximab with supportive care found no change in eGFR decline or in proteinuria after 12 months [105]. Crayne et al. reported a retrospective analysis of eight children treated with rituximab for IgA vasculitis, which showed a reduction in hospitalizations and an increased likelihood of steroid discontinuation. Seven of eight children met remission criteria (no active rash, arthritis, nephritis, or gastrointestinal symptoms) following rituximab treatment [106].

*Calcineurin inhibitors*: A recent meta-analysis of seven trials found limited benefit on proteinuria and none on eGFR of calcineurin inhibitors when used in addition to prednisone, and showed a higher risk of adverse events [107].

*Hydroxychloroquine:* This drug showed some short-term reduction of proteinuria in uncontrolled studies in adults with IgAN [108]. No reports are available for children.

*Future perspectives*: As reviewed by Selvaskandan et al., the last couple of years have seen a tremendous increase in clinical trials for patients, mainly adults, with IgAN, culminating in the approval of two therapeutic agents [109]. Oral targeted enteral-release budesonide received approval from the United States Food and Drug Administration (FDA) in 2021 for the reduction of proteinuria in adult patients with IgAN at risk for rapid disease progression (defined as PCR ≥ 1.5 g/g; proteinuria > 2 g/day) [110]. Sparsentan, a non-immunosuppressive oral dual angiotensin receptor II and endothelin receptor blocker, also received accelerated approval by the FDA in 2023 for adult IgAN patients at risk for rapid progression [111]. The oral factor B inhibitor iptacopan, able to selectively inhibit the alternative pathway of complement, appears to significantly reduce proteinuria in phase II studies [112] and the phase III study should release results in the coming year [112], while the phase III double-blind, randomized, placebo-controlled study evaluating narsoplimab, a monoclonal antibody targeting mannan-binding lectin-associated serine protease-2 (MASP-2) of the lectin pathway, in patients with IgAN with high baseline proteinuria failed to reach its primary endpoint at 36 weeks [113]. Trials evaluating B-cell modulating agents (inhibiting APRIL, BAFF, or TACI), other complement inhibitors, and other anti-proteinuric agents (dapagliflozin, atrasentan) are also ongoing [114]. As these agents begin to be investigated in children, enrolling them in clinical trials and defining children at high risk of progression should be a priority. Ongoing clinical trials are listed here: https://www.clinicaltrials.gov/search?cond=IgA%20Nephropathy%20%5C(IgAN%5C)&term=Pediatric.

### Additional supportive measures necessary for children with IgAN

- Recommendation 3d

- Vitamin D
  - We recommend following international guidelines for vitamin D supplementation for healthy children (grade X, moderate recommendation).

- Immunizations
  - We recommend following national immunization guidelines, according to age (grade X, strong recommendation).
  - Live vaccines should not be given to children receiving daily glucocorticoids and/or immunosuppressive medications (grade C, moderate recommendation).

- Fish oil
  - We do not recommend the use of fish oil in children with IgAN (grade C, moderate recommendation).

#### Evidence and rationale

*Vitamin D*: Low levels of 25-hydroxy-vitamin D are associated with poor outcomes in adults with IgAN [115]. A single-center RCT on adults with IgAN and moderate proteinuria (urinary protein excretion 1.0–3.0 g/day) compared valsartan (160 mg/day) or valsartan plus activated vitamin D (calcitriol) 0.5 μg/day [116]. Valsartan plus activated vitamin D was found to be more effective than valsartan alone in reduction of moderate proteinuria and without an increase in adverse events, suggesting an additive positive effect of vitamin D. A meta-analysis of 7 RCTs evaluating the effect of calcitriol in 310 Chinese patients with IgAN and non-nephrotic-range proteinuria showed that calcitriol resulted in decreased proteinuria (standard mean difference (SMD) − 1.49, 95% CI (− 2.37, − 0.62); *P* = 0.0008) [117]. A small RCT in adult patients with IgAN showed a significant decrease in proteinuria when calcitriol was added to a RASB [118]. However, there are no controlled trials evaluating the effects of vitamin D supplementation in children with IgAN.

*Immunizations*: There are individual case reports and a few case series reporting episodes of gross hematuria, increasing proteinuria, and new-onset IgAN after immunization with the COVID-19 vaccine [119–121]*.* However, there are no controlled studies to establish a causal relationship. Given the strong scientific evidence that supports the benefit of immunizations in children, including COVID-19, the benefit-risk balance supports that the routine immunization schedule should not be altered in children with IgAN, except for avoiding live vaccines while the patient is receiving daily immunosuppressive therapy.

*Fish oil*: A multicenter RCT on 106 adults with IgAN and persistent proteinuria (> 1 g/day) demonstrated that treatment for 2 years with 12 g/day of fish oil slowed the progression of kidney disease, compared to placebo [122]*.* Further follow-up of these patients for 6.4 years demonstrated that longer treatment with fish oil slowed kidney disease progression for high-risk patients with IgAN [123]. Similarly, a small, controlled trial in Italy (30 adults) showed that treatment for 6 months with 3 g/day of polyunsaturated fatty acids in patients with proteinuric IgAN who were receiving ramipril and irbesartan had a stronger effect reducing proteinuria than ramipril-irbesartan alone [124]. However, a double-blind RCT on children and young adults with IgAN with moderate-to-severe proteinuria failed to show the superiority of omega-3 fatty acids or prednisone over placebo [87]. Noticeably, this study had multiple flaws, including a difference in the baseline proteinuria among the groups, with higher urinary protein excretion in the group that received omega-3 fatty acids. Altogether, given these conflicting results, the long-term efficacy of fish oil in decreasing proteinuria and progression to kidney failure in children with IgAN is unproven.

### Tonsillectomy

- Recommendation 3e
  - We do not recommend tonsillectomy as a treatment for IgAN in children (grade C, moderate recommendation).

#### Evidence and rationale

Currently, in clinical practice, tonsillectomy plus glucocorticoid pulse therapy is often used in adult patients with IgAN in Japan. However, there is little evidence supporting this practice even in Japan, and no evidence in pediatric patients. Although tonsillectomy in children with IgAN complicated with recurrent tonsillitis and concomitant hematuria makes sense, we should be more cautious than in adults on the indication for tonsillectomy in children, whose immune system is still developing. Therefore, there is no evidence in the present international report to recommend tonsillectomy in pediatric IgAN, and we suggest that it be considered in limited settings where repeated episodes of hematuria are associated with acute recurrent tonsillitis or when there is an otolaryngological indication for this surgical procedure. Given the potential complications of tonsillectomy in children, primarily bleeding, the risk–benefit balance of this procedure must be carefully considered.

### Atypical forms of IgAN: definitions and treatment

- Recommendation 4a
  - We recommend treating children presenting with nephrotic syndrome and MCD on biopsy with associated IgA deposition as per the IPNA Steroid Sensitive Nephrotic Syndrome (SSNS) Guideline (grade C, weak recommendation).

#### Evidence and rationale

*MCD with IgA deposits*: A small subset of children presenting with NS are found to have pathological features suggestive of MCD, with IgA deposition on immunofluorescence but lacking identifiable activity meeting a MEST-C score > 0, according to the revised Oxford Classification. In adults, NS has been associated with biopsy-proven IgA deposits and a primary podocytopathy which is glucocorticoid sensitive and largely indistinguishable from MCD. Literature is limited in children, but like adults, they appear to respond to glucocorticoid therapy; therefore, we suggest treatment as per the SSNS guideline [2, 125].

*IgAN with nephrotic syndrome*: Several children with nephrotic syndrome are found to have glomerulonephritis consistent with IgAN on biopsy, i.e., podocytopathy, and any two of M1, E1, S1, and C1-2 irrespective of T score. In a series of 426 Japanese children presenting with NS as per the International Study of Kidney Disease in Children (ISKDC) criteria, 7% (*n* = 30) were found to have IgAN on biopsy [126]. IgAN with NS is associated with higher levels of mesangial and endothelial hypercellularity and crescents on biopsy and a fivefold risk of progressive kidney disease when compared to other children with IgAN without NS at presentation [126]. Children with IgAN and NS have lower rates of complete or partial remission with glucocorticoids alone, with one study reporting 24% of children with IgAN and NS in complete remission with glucocorticoids alone [127]. Additional immunosuppressive therapies reported include azathioprine (2 mg/kg/day), mycophenolate mofetil (20–30 mg/kg/day), and cyclosporine with or without glucocorticoids and have been shown to improve rates of remission although studies are limited by small sample size and retrospective cohort design [126–128]. Among children treated with mycophenolate mofetil (20–30 mg/kg/day), 64% achieved complete remission with a further 18% achieving partial remission [127]. Similarly, a 70% rate of remission was seen when azathioprine (2 mg/kg/day) was used in conjunction with glucocorticoids and other adjunct therapy including heparin-warfarin and dipyridamole [126]. In a small series of children with IgAN presenting with NS where many patients received cyclosporine (4–5 mg/kg/day) in combination with glucocorticoids, 89% of patients went into remission [128].

### IgAN with rapidly progressive glomerulonephritis (RPGN)

- Recommendation 4b
  - We recommend performing a kidney biopsy in any child presenting with signs of IgAN and rapidly progressive glomerulonephritis (RPGN), defined as an unexplained > 50% decline in eGFR in ≤ 3 months (grade A, moderate recommendation).

- Recommendation 4c
  - We recommend defining IgAN with RPGN as IgAN presenting with a > 50% decline in eGFR over ≤ 3 months and evidence of endocapillary proliferation (at least E1) and crescents in at least 25% of glomeruli (C2) (grade X, moderate recommendation).
  - We recommend treating IgAN with RPGN with i.v. glucocorticoids + / − other immunosuppressive therapies such as cyclophosphamide or mycophenolate mofetil (grade C, moderate recommendation).

#### Evidence and rationale

Rapidly progressive IgAN is defined as the rapid decrease in kidney function over weeks to months as well as signs and symptoms of nephritic syndrome, such as proteinuria, glomerular hematuria, anemia, and oliguria, where other causes of a RPGN, such as ANCA-associated vasculitis (AAV), anti-GBM disease, drug toxicity, and pre- and post-kidney causes, are ruled out. Kidney biopsy is essential for these patients, and the pathology must show endocapillary proliferation (at least E1) and crescents in at least 25% of glomeruli (C2). Rapidly progressive IgAN is associated with poor kidney outcomes, and thus despite lack of evidence, this group of children should be offered treatment with glucocorticoids (usually as methylprednisolone pulses) and cyclophosphamide. It is reasonable to use a similar treatment protocol as that of AAV with kidney involvement [2].

### Remission and relapse

#### Remission definition

Remission of IgAN: resolution of proteinuria (UPCR < 0.2 mg/mg or 20 mg/mmol or proteinuria < 100 mg/m^2^ per day or < 0.2 g/day in 24-h collection) based on at least two urine samples collected at least 1 month apart in the presence of normal (≥ 90 mL/min/1.73 m^2^) or stable eGFR. Complete remission includes, in addition to these features, the resolution of hematuria, defined as a negative dipstick for blood and/or < 5 RBC/high-power microscopic field (grade X, moderate recommendation).

#### Evidence and rationale 

There are few studies of therapy-induced remission of IgAN in children, although a uniform definition of remission should be employed regardless of whether this is spontaneous or therapy-induced and regardless of the specific therapy given. In different studies, remission was defined by reduction of proteinuria below a certain threshold, with or without reduction of hematuria below a certain threshold, and adults and children were generally considered together. Commonly used thresholds are proteinuria of < 0.3 g/day (UPCR 0.3 g/g or 30 mg/mmol) and hematuria of < 5 RBC/high-power field although lower thresholds of proteinuria have also been used (< 0.15 g/day) [129–131]. In adults and children, IgAN with proteinuria of < 0.3 g/day is generally associated with minimal or very mild histologic lesions and an excellent prognosis without immunosuppressive therapy [40, 132, 133]. A 2024 publication on pediatric IgAN defined complete remission as remission of proteinuria (UPCR < 0.2 mg/mg or 20 mg/mmol) and microscopic hematuria (< 5 RBC/high-power field), with normal eGFR (≥ 90 mL/min/1.73 m^2^) and normal blood pressure, off all medication for 2 years [134]. Accordingly, we have chosen to err on the side of caution and use a slightly lower threshold, as it is the threshold that is used for remission of NS, the most frequent glomerular disease of childhood. A repeat biopsy is not felt to be necessary to define a remission of IgAN.

### Relapse of IgAN: diagnosis

#### Relapse definition

Relapse of IgAN: reappearance of PCR > 0.5 mg/mg (50 mg/mmol) or proteinuria > 0.5 g/day (> 0.25 g/m^2^ per day) in 24-h collection based on at least two urine samples collected 1–2 weeks apart in patients who previously achieved remission. This is further supported by concomitant hematuria and/or reduced eGFR (< 90 mL/min/1.73 m^2^) or declining eGFR after exclusion of other causes of kidney injury (grade X, moderate recommendation).

*Note:* This definition leaves a gray zone between 0.2 and 0.5 g/g (20–50 mg/mmol, 0.2–0.5 g/day, or 0.1–0.25 mg/m^2^ per day) of proteinuria. In a patient with IgAN in follow-up who previously achieved remission and who has developed proteinuria within this range despite adherence to RASB if prescribed, we recommend repeating proteinuria assessment three to four times within 4–8 weeks to ascertain the real value of proteinuria. The prognostic significance of this level of proteinuria, as of microhematuria, is uncertain, and while it may warrant optimizing conservative therapy, perhaps it does not warrant the use of immunosuppression.

#### Evidence and rationale

Again, there are few studies defining a relapse of IgAN following remission. The minimal criteria for a relapse are proteinuria of > 0.5 g/day, with or without hematuria and/or reduced kidney function. In studies where absent/minimal hematuria is a criterion for remission, the recurrence of hematuria above the threshold has been considered to represent a relapse [130]. Intuitively, hematuria might be taken as a marker of active glomerular inflammation, and clinically, children with IgAN are known to frequently recur with repeated episodes of synpharyngitic hematuria preceding the reappearance of significant proteinuria. However, reviews of clinical parameters in adults and children generally do not list hematuria as being significantly associated with kidney function decline or kidney failure by either univariate or multivariable analysis, while proteinuria, especially during follow-up, is a well-recognized risk factor for disease progression in IgAN in both children and adults [135, 136]. The usefulness of persistent microscopic hematuria in predicting IgAN progression has been recently re-evaluated in adults and studies are ongoing in children with IgAN [54].

### Management of relapse of IgAN

- Recommendation 5b
  - We recommend that follow-up with urinalysis, blood pressure assessment, and periodic clinical evaluation be continued lifelong in children with IgAN to detect relapses (grade X, strong recommendation).
  - We suggest the implementation of optimal conservative treatment, including moderate sodium intake (i.e., below 3–5 g/day) and maximal RASB, for all patients with evidence of a relapse of IgAN (grade C, weak recommendation).
  - Further treatment options include all therapies used in the initial treatment of IgAN as outlined in the above recommendations.

#### Evidence and rationale

There is no evidence to guide statements on the treatment of relapses of IgAN. When a child presents with worsening of proteinuria, ± increased microhematuria, and/or unexpected decline in eGFR, a new phase of activity needing treatment is suspected and a repeat kidney biopsy may be useful to verify changes compared to the first kidney biopsy.

### Treatment discontinuation in children with IgAN

- Recommendation 6a
  - We suggest immunosuppressive treatment be discontinued in children with IgAN who achieved a full remission (UPCR < 0.2 g/g or 20 mg/mmol) for at least 12 months (grade C, weak recommendation).
  - We suggest continuing supportive therapy with RASB and lifelong monitoring, individualized according to the severity and response to treatment (grade C, weak recommendation).

#### Evidence and rationale

Studies are lacking in directing the optimal time to stop immunosuppressive treatment in children with IgAN after achieving complete remission. Observational studies report a wide range of duration of treatment with glucocorticoids and glucocorticoid-sparing immunosuppressive treatments like mycophenolate mofetil, with some studies reporting as little as 6 months to others over 2 years. RASB is usually continued for a longer period even after discontinuation of immunosuppression.

### Recurrence of IgAN post-kidney transplantation: diagnosis

- Recommendation 7a
  - We recommend that diagnosis of recurrent IgAN post-transplantation requires the demonstration of dominant/co-dominant glomerular IgA deposits in a kidney transplant biopsy from a patient whose native kidney disease was IgAN. This finding should be considered the minimum diagnostic criterion for recurrent IgAN and should not include findings on a perioperative or post-implantation biopsy which may reflect IgA present in the donor kidney (grade X, moderate recommendation).

#### Evidence and rationale

There are presently no accepted guidelines regarding the treatment of recurrent IgAN after a kidney transplant. However, the issue of how to manage recurrent IgAN in the kidney transplant cannot be properly addressed so long as there is considerable variability in how such a recurrence is defined. In some studies, a diagnosis of recurrent IgAN requires only the presence of glomerular IgA deposits on a kidney allograft biopsy, while in others, additional urinary (hematuria and/or proteinuria) and/or histologic (e.g., mesangial hypercellularity) abnormalities are required. These differences account for the wide variability (10–60%) of reported IgAN recurrence rates [137, 138].

For native kidney IgAN, diagnosis requires a biopsy showing dominant/co-dominant glomerular IgA deposits by immunofluorescence or immunohistochemistry studies, and it is suggested that for recurrent IgAN in a transplant, similar diagnostic criteria should be applied to those for native kidney biopsies, noting that even in kidney transplant recipients with documented native kidney IgAN, the differential diagnosis of urinary abnormalities includes multiple etiologies that can only be distinguished by biopsy. While the minimal diagnostic criteria for recurrent IgAN are as noted above, several pieces of evidence indicate that a diagnosis of *clinically significant recurrent IgAN* should require urinary abnormalities in addition to glomerular IgA deposits. First, mesangial IgA deposits have been reported in a significant number of healthy kidney donors, usually 4–5% but as many as 16–20% in two studies, and as such IgA deposits on a post-implantation biopsy should not be regarded as diagnostic of recurrent IgAN [139, 140]. These deposits are resorbed over time in non-IgAN recipients [141]. Second, IgA deposits in native kidneys with minimal or no associated urinary abnormalities typically have no significant impact on long-term kidney function [133]. The exact amount of proteinuria and hematuria required, in addition to glomerular IgA deposits, to diagnose clinically significant recurrent IgAN is debatable; however, it was suggested that hematuria in the absence of proteinuria and proteinuria in the absence of hematuria are not reliable indicators of recurrence [138]. Thus, the presence of each of the three criteria, glomerular IgA deposits, proteinuria above a level typically seen in normally functioning allografts (e.g., > 0.3 g/day or g/g creatinine, after accounting for possible native kidney proteinuria), and hematuria, is suggested as a requirement for defining clinically significant recurrent IgAN. However, it should be noted that in some patients with glomerular IgA deposits, hematuria and/or proteinuria may develop later, so follow-up urinalysis is advised [138]. As opposed to proteinuria and hematuria, histologic findings (e.g., mesangial expansion) may be present in donor kidneys (e.g., with latent early diabetic nephropathy or hypertensive nephrosclerosis) and may be a manifestation of chronic rejection, and as such, their utility as a marker for clinically significant recurrent IgAN (even in the presence of mesangial IgA deposits) appears limited.

### Recurrence of IgAN post-kidney transplantation: management

- Recommendation 7b
  - We recommend optimizing supportive care of children with recurrence of IgAN post-kidney transplantation by providing dietary counseling to reduce salt intake. We also recommend regular aerobic exercise, targeting the ideal body weight, and no smoking or vaping (grade X, moderate recommendation).
  - We suggest maximal RASB for all children with evidence of IgAN recurrence post-kidney transplantation as defined above (grade C, weak recommendation).
  - Further treatment options for children with recurrence of IgAN post-kidney transplantation include all therapies used in the treatment of IgAN in the native kidney (see above) (grade X, moderate recommendation).
  - We recommend NOT implementing glucocorticoid withdrawal in children with recurrence of IgAN post-kidney transplantation (grade X, moderate recommendation).
  - We recommend enrolling children with recurrence of IgAN post-kidney transplantation in clinical trials if proteinuria persists despite adherence to transplant immunosuppressive regimen and optimal conservative therapy (see above) (grade X, moderate recommendation).

#### Evidence and rationale

There are no universally acceptable guidelines for the treatment of recurrent IgAN in children or adults. Risk of recurrence is reported in up to 60% of transplant recipients after 19 years [142]. Historically, this occurrence was considered benign [143, 144]. However, there is a risk of graft loss between 3 and 16% in different reports [145–150]. Glucocorticoid withdrawal may increase the risk of graft loss due to recurrent IgAN [151–153]. One small study showed that in children with IgAN recurrence post-transplantation, restarting treatment for 6 months (prednisone + azathioprine) resulted in decreased proteinuria and disappearance of gross hematuria [154]. The other single-center retrospective cohort study (54 children and adults) indicated that 11 patients (age < 20 years) diagnosed with the recurrent disease did not receive additional immunosuppressive therapy and there was no significant increase in graft loss compared with non-recurring patients [145].

RASB has also been recommended in patients with recurrent IgAN and proteinuria of > 0.5 g/day by KDIGO clinical practice guideline for the care of kidney transplant recipients based on two single-center studies (Oka et al. [21 adults] and Courtney et al. [75 adults]) [155–157]. Moreover, as with IgAN on native kidneys, moderate sodium intake (i.e., below 3–5 g/day), aerobic exercise, and avoidance of muscle-gaining mass protein powder or formulations and smoking and vaping are beneficial.

As stated above, a variety of innovative therapeutic approaches are being tested for IgAN and may well prove effective in recurrent disease [158]. Due to the significant risk of graft loss, whenever proteinuria persists despite optimal management, it is advisable to enroll these children in clinical trials, whenever possible.

### Clinical practice recommendations for IgA vasculitis nephritis (IgAVN)

#### Definitions

Recommended definitions are listed in Table 1 and in the text as appropriate.

## IgAVN in children: workup and diagnosis

### Diagnosis of IgAVN in children

- Recommendation 1a
  - There is no single diagnostic test for IgAV. Diagnosis therefore relies on clinical criteria and laboratory findings (grade X, strong recommendation).

#### Evidence and rationale

The diagnosis of IgAV is usually straightforward in a child with suggestive clinical features: palpable purpura in a typical distribution (predominantly extensor surfaces of lower limbs), abdominal pain, arthritis/arthralgia with the exclusion of alternative forms of autoimmune vasculitides (such as ANCA-associated vasculitis, polyarteritis nodosa, or lupus), and alternative causes such as leukemia or autoimmune thrombocytopenia [21]. Rarely, other organ systems such as the central nervous system or respiratory tract can also be affected [21].

There are no validated definitions or diagnostic criteria for IgAV in children [21]. The nomenclature used in the Chapel Hill Consensus Conference (CHCC) definition has never been validated for formal classification or diagnostic purposes [159]. Classification criteria may be used to inform clinical or epidemiological studies of IgAV in children but should not be used to diagnose IgAV in individual patients since they are not validated [21, 160, 161]. Two main sets of classification criteria have been described for IgAV: the American College of Rheumatology (ACR) classification criteria and the Ankara 2008 criteria [160, 162, 163]. The Ankara 2008 criteria should be used to classify pediatric IgAV. These criteria were informed by a large international registry of patients, and validated specifically for childhood-onset disease [163].

#### Diagnostic utility of skin biopsy

Palpable purpura predominantly (but not exclusively) on the buttocks and lower limbs is typical. Leukocytoclastic vasculitis associated with IgA deposition in a skin biopsy is helpful in the diagnosis [21]. Typical lesions, however, do not warrant a skin biopsy. Atypical cases should be investigated with a skin biopsy with specific staining for IgA. Examples of such scenarios are patients with extensive lesions or when there is a need to exclude alternative diagnoses (e.g., ANCA-associated vasculitis in older children and adolescents). The absence of IgA staining on skin biopsy does not completely exclude the diagnosis of IgAV [164]. Similarly, the finding of IgA on tissue biopsy does not necessarily completely secure the diagnosis of IgAV [165]. The skin biopsy should sample the most recent (fresh) lesions and should be of sufficient depth to include dermal capillaries to optimally detect IgA staining [21].

### Initial assessments (clinical or laboratory) to evaluate kidney involvement

- Recommendation 1b
  - Kidney involvement should be assessed in all children with IgAV with clinical and laboratory findings including evaluation of peripheral edema, measurement of blood pressure and plasma albumin, calculation of eGFR, and urinalysis (hematuria and proteinuria quantification) (grade X, strong recommendation).

#### Evidence and rationale

Kidney involvement of IgAV occurs in 20–80% of children. Presentations can range from isolated microscopic or macroscopic hematuria with or without proteinuria, nephritic, and/or nephrotic syndrome. Mild kidney involvement can lead to an excellent prognosis, although definitions of what constitutes mild, moderate, or severe kidney involvement vary across studies [21, 166]. The key goal for the initial diagnostic workup is early detection of IgAVN [21]. Persistent kidney inflammation may progress to permanent kidney damage and scarring [38, 167]. However, signs of IgAVN usually are limited to urine abnormalities without overt clinical symptoms in children; they often have normal blood pressure and kidney function. Nephritis may recover without treatment [21].

All children with suspected IgAV must therefore be actively investigated with blood pressure measurement, careful clinical assessment for peripheral edema, first-morning void urinalysis, and assessment of kidney function with eGFR [21]. Urinalysis should include documenting the presence or absence of hematuria and quantification of proteinuria with first-morning void UPCR (see Table 1) [21]. Urinalysis should be performed at IgAV onset and repeated at least monthly subsequently for at least 6 months (see below). It is also important to exclude orthostatic proteinuria. With severe abdominal pain, an ultrasound should be performed to exclude intestinal intussusception [21].

### Indications for kidney biopsy in children with suspected IgAVN

- Recommendation 1c-(i)
  - We recommend performing a kidney biopsy in children with IgAV and nephrotic-range proteinuria (UPCR > 2 mg/mg or 200 mg/mmol) (grade X, strong recommendation).

- Recommendation 1c-(ii)
  - We recommend performing a kidney biopsy in children with IgAV and eGFR < 90 mL/min/1.73 m^2^ irrespective of the level of proteinuria (grade X, moderate recommendation).

- Recommendation 1c-(iii)
  - We suggest performing a kidney biopsy in children with IgAV and moderate proteinuria (UPCR 1–2 mg/mg or 100–200 mg/mmol) for 2–4 weeks (grade D, weak recommendation).
  - We suggest performing a kidney biopsy in children with IgAV and mild proteinuria (UPCR 0.2–0.5 mg/mg or 20–50 mg/mmol) for more than 4 weeks (grade D, weak recommendation).

#### Evidence and rationale

**Fig. 3:**
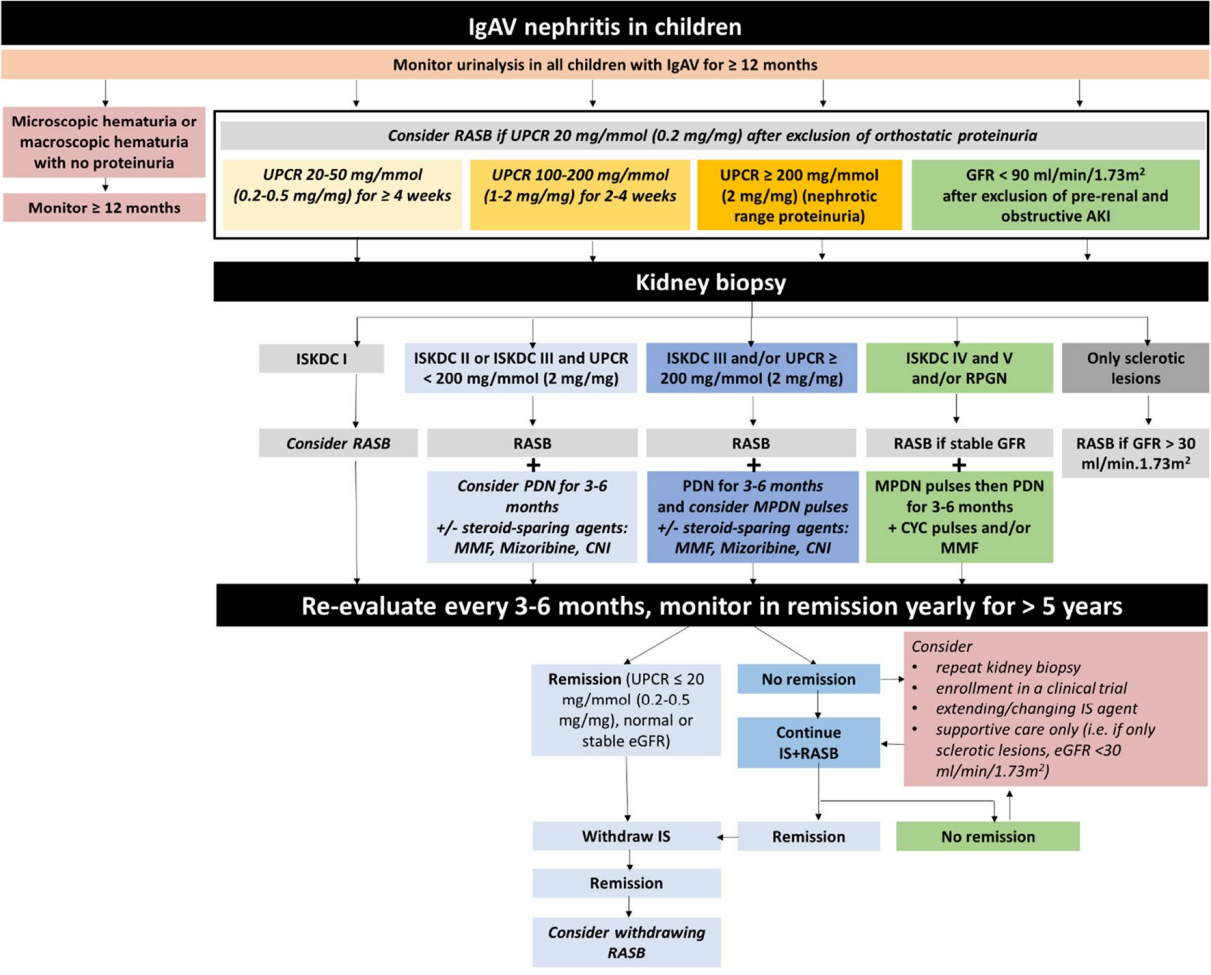
Management algorithm for IgAVN

There are no prospective observational studies to inform kidney biopsy in IgAV. We recommend, as per the SHARE guidelines, performing a kidney biopsy in children with IgAV and persistent proteinuria, nephrotic-range proteinuria, and/or reduced eGFR (Fig. 3) [21]. In addition, relative indications are also listed in Table 5. Some experts administer RASB prior to kidney biopsy, but this may mask the disease activity of IgAVN (see below). The use of immunosuppressive drugs for the treatment of nephritis may also mask active lesions in a subsequently performed kidney biopsy. However, glucocorticoids are frequently necessary to treat extra-kidney manifestations (cutaneous, intestinal, etc.) [21].

**Table 5:** Indications for kidney biopsy in IgA vasculitis

Clinical or laboratory feature	Level of evidence
**Absolute indications**	
Severe proteinuria > 2 mg/mg or 200 mg/mmol	D
Persistent moderate proteinuria: 1–2 mg/mg or 100–200 mg/mmol for 2–4 weeks	D
Impaired eGFR: < 90 mL/min/1.73 m^2^	D
**Relative indications**	
Persistent mild proteinuria: 1–2 mg/mg or 20–100 mg/mmol for 12 weeks	D
Hypertension	D

*eGFR*, estimated glomerular filtration rate

At present, the best-studied histologic scoring system for IgAVN is the International Study for Kidney Disease in Children (ISKDC) since validation of MEST-C scores in IgAV is very limited, although additional studies of the latter are in progress. The biopsy should be processed for light microscopy (with hematoxylin/eosin, periodic acid Schiff [PAS], Masson’s trichrome, and methenamine silver stains); immunofluorescence/immunohistochemistry for immunoglobulins (IgG, IgA, IgM), complement components (C3, C1q, possibly C4), kappa and lambda light chains, and fibrin; and electron microscopy (when available, at the discretion of the pathologist), and scored using the ISKDC scoring system [168]. Recently, however, an international study of 361 patients (262 children, 99 adults) showed that endocapillary hypercellularity (E1), which is not considered in ISKDC, is an important predictor of late eGFR decline (more so than crescents, which are the main discriminator between ISKDC grades III, IV, and V). Thus, while the use of ISKDC is recommended at the present time, the inclusion of MEST-C scores appears to be valuable and the inclusion of both ISKDC and MEST-C in the biopsy report should be considered [169]. Of note, this study showed more mesangial and endocapillary hypercellularity in children than in adults, suggesting that in those children with an initial clinical presentation severe enough to warrant a biopsy, the clinical course of IgAVN may be less benign than was previously thought [169].

### Follow-up and subsequent assessments

- Recommendation 1d-(i)
  - Follow-up assessments should be tailored to the detection of late kidney involvement, even if there was no kidney involvement at the first presentation, and should include all the assessments as per recommendation 1b. Follow-up should be at least monthly for the first 6 months even if urinalysis remains normal (grade X, moderate recommendation).

- Recommendation 1d-(ii)
  - Patients who had clinical nephritis should be followed closely for at least 5 years and longer in patients with initially active disease. Follow-up may be conducted by primary care providers (grade X, strong recommendation).

- Recommendation 1d-(iii)
  - We suggest a minimal frequency of monitoring for those with kidney involvement: monthly for 6 months, every 3 months for an additional 6 months, and thereafter every 6 months for a minimum of 5 years (grade X, weak recommendation).

#### Evidence and rationale

There are no robust prospective studies that directly inform guidelines on follow-up. That said, several important and consistent observations emerged from retrospective studies that clinicians can use when considering the follow-up of individual patients.

1. A meta-analysis of 1133 children followed for a minimum of 6 weeks and a maximum of 36 years found that no long-term kidney impairment occurred after normal urine analysis, with no need to follow up after 6 months if the urine analysis remains normal [170].
2. Patients with relapsing IgAV or severe abdominal involvement are at higher risk of developing kidney involvement even if this was absent at first presentation [21, 171].
3. Patients with mild nephritis may also develop poor long-term kidney outcomes. This suggests that current definitions of mild, moderate, and severe IgAVN may require reappraisal. Future prospective studies might explore the clinical impact of performing kidney biopsy even in children with apparently milder forms of proteinuria at first presentation [21].
4. Broadly speaking, ISKDC histopathological features equate to the severity of the clinical kidney involvement at presentation but they are overall a less reliable determinant of long-term kidney outcomes [38]. This is possibly due to the effect of immunosuppressive treatment ameliorating acute small vessel vasculitis, although this remains unproven [172].
5. Histopathology of kidney biopsy (glomerulosclerosis) suggestive of scarring is associated with poor long-term kidney prognosis [173]. This might argue for doing a kidney biopsy earlier in the diagnostic journey than is currently recommended.
6. Persistent proteinuria (UPCR > 1 mg/mg or 100 mg/mmol) at 1 year is associated with poor long-term kidney prognosis and is not amenable at this stage to immunosuppression since it is likely a reflection of kidney scarring that may not warrant immunosuppression [38, 166].
7. A suggested minimal frequency of monitoring for those with kidney involvement: monthly for 6 months, every 3 months for 6 months, thereafter, every 6 months for at least 5 years [174].
8. Lifelong yearly evaluation of urinalysis and blood pressure may be prudent in patients with moderate to severe forms of IgAVN [61, 62, 167, 173].

## Prevention and management of IgAVN in children

### Prevention of IgAVN

- Recommendation 2a-(i)
  - We do NOT recommend using glucocorticoids to prevent IgAVN (grade B, moderate recommendation).

- Recommendation 2a-(ii)
  - We do NOT recommend using heparin, dipyridamole, aspirin, or montelukast to prevent IgAVN (grade B, moderate recommendation).

#### Evidence and rationale

A recent Cochrane review summarized the data of 11 studies enrolling children with IgAV and no kidney involvement or mild degrees of hematuria and proteinuria [23]. The analysis concluded that oral glucocorticoids compared with placebo did not significantly reduce the risk of development of nephritis (5 studies on 746 children) or persistence of kidney involvement (2 studies on 418 children) at 3 months or 12 months with moderate and high certainty evidence, respectively. Among these, one large RCT allocated 352 children with IgAV to receive either prednisolone (2 mg/kg/day (maximum dose 80 mg) for 7 days, followed by 1 mg/kg/day (maximum dose 40 mg) for 7 days) or placebo for 14 days [175]. The incidence of proteinuria (UPCR > 0.2 mg/mg or 20 mg/mmol) was similar between the two groups at 12 months. Two smaller randomized trials compared oral prednisone with placebo and found no difference in the occurrence of microscopic hematuria or proteinuria within 1 year of the diagnosis of IgAV [176, 177]. Similarly, three small studies found the addition of antiplatelet therapies (dipyridamole, cyproheptadine, aspirin) to supportive care did not induce any reduction in IgAVN incidence [178, 179]. It is uncertain whether heparin has any effect on the outcomes since two studies (He 2002, Tian 2015) yielded contradictory results [23, 180]. Due to the potential adverse effects of such therapies, we do not recommend their use to prevent IgAVN in children. One study compared a 3-month course of montelukast sodium as add-on therapy with a placebo in 84 children with IgAV without nephritis [181]. This intervention did not prevent further relapse of IgAV, or alter the outcome of nephritis. Current low-level evidence does support the use of glucocorticoids for the extra-kidney manifestation of IgAV, as recommended by SHARE [21].

### Treatment of IgAVN

The guideline committee was unable to draw any evidence-based conclusions on treatment for IgAVN in children, either regarding the selection of patients who will benefit from treatment or regarding the dose and duration of treatment. Based on consensus methods and iterative discussion and voting, the expert group has prepared the following statements:

- Recommendation 2b-(i)
  - We suggest monitoring for the development of proteinuria in children with IgAVN for at least 12 months (grade D, weak recommendation).

- Recommendation 2b-(ii)
  - The target of treatment is to reduce proteinuria to UPCR < 0.2 mg/mg (20 mg/mmol). The follow-up must be continued to detect relapses for at least 5 years (grade X, moderate recommendation).

- Recommendation 2b-(iii)
  - We do NOT recommend the use of glucocorticoids for IgAVN in the following situations: isolated microscopic or macroscopic hematuria with no proteinuria (UPCR < 0.2 mg/mg or 20 mg/mmol) (grade B, moderate recommendation).

- Recommendation 2b-(iv)
  - We suggest treating children with nephrotic-range proteinuria (UPCR > 2 mg/mg or 200 mg/mmol) or RPGN and histological risk for progression (ISKDC ≥ II) with a 3–6-month course of glucocorticoid, either as i.v. pulses followed by tapering oral daily dose or as an oral course (grade D, weak recommendation).

- Recommendation 2b-(v)
  - We suggest using other immunosuppressive agents in addition to glucocorticoids (e.g., CNIs, cyclophosphamide, mizoribine where available, or mycophenolate mofetil) in the following indications: to reduce the glucocorticoid dose and/or if PCR > 2 mg/mg (200 mg/mmol) and/or insufficient response to glucocorticoids (grade D, weak recommendation).

- Recommendation 2b-(vi)
  - We suggest considering the use of RASB (ACEi or ARB) in children with IgAVN and proteinuria (UPCR ≥ 0.2 mg/mg (20 mg/mmol)) (grade D, weak recommendation).

- Recommendation 2b-(vii)
  - We suggest performing a repeat kidney biopsy for histological re-evaluation of the treatment if proteinuria persists for > 4 weeks (grade D, weak recommendation).

#### Evidence and rationale

Currently, due to the paucity of RCTs with head-to-head comparisons, there is little evidence to guide the choice of treatments in children with IgAVN. Furthermore, these studies have not addressed relapses of IgAVN. A 2023 Cochrane review summarizing data from 11 studies found no evidence that some agents are more effective than others in treating kidney involvement of IgAVN [23], and these studies are described in more detail in Supplementary Tables S7 and S8. Based on consensus methods and iterative discussion and voting, the working group has proposed a management algorithm for IgAVN (Fig. 3) which also takes into consideration the natural history of IgAVN which can manifest during the first year from presentation with subsequent relapses presenting over years of follow-up.

Children with no risk for progression may be those with isolated microscopic hematuria or gross hematuria which are completely resolved, and thus, they are without any persistent urinary abnormalities. Such children should not receive treatment; however, careful follow-up is needed.

Treatment with glucocorticoids and other immunosuppressants is determined based on both histologic (ISKDC and/or MESCT-C grading) (Supplementary Table S5) and clinical severity, considering the risk–benefit profile for individual patients.

*Glucocorticoids*: Although glucocorticoids are recommended as the primary treatment for children with IgAVN, the data to support their use are scarce and primarily come from observational studies [159]. Most published trials include glucocorticoids in their treatment protocols, but the heterogeneity of the data precludes drawing any definitive conclusions. Some protocols use methylprednisolone pulses followed by oral glucocorticoids in tapering doses, especially in the most severe cases when kidney histology shows active proliferative lesions in > 50% of glomeruli (ISKDC ≥ IV), or when there is nephrotic proteinuria, or RPGN [159]. Others will use oral prednisone or prednisolone only [159].

One prospective, uncontrolled study used three intravenous methylprednisolone pulses followed by oral prednisone for 3 months in 38 children presenting with the NS and/or crescents affecting more than 50% of glomeruli [182]. Four of these 38 progressed to kidney failure, and three of these were treated late in the course of their disease. This 1998 study suggested that early therapy may be beneficial in preventing irreversible glomerular injury.

A suggested protocol for glucocorticoid is 1–2 mg/kg/day of oral prednisolone/prednisone (maximum dose 60 mg daily) for 4 weeks, followed by alternate-day dosing tapered over 3–6 months (Table 4) (Fig. 3). Intravenous methylprednisolone can be used for patients with nephrotic-range proteinuria, ISKDC ≥ III, or RPGN with suggested three doses given daily for 3 days at induction, 500 mg/m^2^ per dose (maximum dose of 1000 mg). Some treatment protocols use repeated doses of methylprednisolone [108].

Moreover, a short course of glucocorticoids may be needed for extra-kidney manifestations, specifically severe bowel symptoms after ruling out intussusception, severe arthritis, orchitis, etc. However, this does not prevent kidney involvement.

*Other immunosuppressive agents*: Three published small RCTs assessed other immunosuppressive agents. Tarshish et al. compared oral cyclophosphamide 90 mg/m^2^/day for 42 days, with no therapy, in 56 children with IgAVN with crescents and found little difference in kidney outcomes (persistent kidney disease, severe kidney disease as defined by heavy proteinuria, reduced eGFR, or kidney failure) after a mean follow-up of 7 years [183]. Jauhola et al. compared cyclosporine to methylprednisolone pulses in a small number of children with biopsy-proven crescentic IgAVN of ISKDC grade III or IV, or grade II with NS [184]. All children treated with cyclosporine achieved remission of nephrotic-range proteinuria at 3 months compared to only 50% of those treated with prednisolone. Limitations of this study included a small sample size, incomplete randomization, and non-blinding. Zhang et al. studied standard therapy (glucocorticoids and i.v. cyclophosphamide) versus tacrolimus in Chinese children and showed improvement in hematuria and proteinuria in both groups at 2 years, although better in the tacrolimus group; a key limitation was the open-label nature of the study, resulting in reporting bias [23, 117, 185].

There are two published observational studies examining the use of mizoribine for IgAVN. In 2011, Ninchoji et al. reported combination therapy including glucocorticoids, azathioprine or mizoribine, warfarin, and dipyridamole in 19 childhood IgAVN cases with serum albumin levels ≤ 2.5 g/dL and ISKDC classification Grade IV or higher, and found that proteinuria disappeared in all patients, without kidney dysfunction, and no serious side effects [186]. In 2023, Nagai et al. reported favorable outcomes with combination treatment with glucocorticoids, mizoribine, and RAS inhibitors for pediatric IgAVN in whom RAS inhibitors alone were ineffective [187].

Published observational studies on other immunosuppressive agents in children with IgAVN with and without glucocorticoids are generally small. The largest single-center study (*n* = 61) found that addition of mycophenolate mofetil (20–30 mg/kg/day) to glucocorticoids in patients with IgAVN and nephrotic-range proteinuria resulted in a higher rate of complete remission at 6 months (*n* = 26/41) when compared to glucocorticoids alone (*n* = 7/20); however, by 23 months of follow-up, there were similar rates of complete remission between the two groups [188]. Other smaller single-center observational studies have shown similar favorable responses to mycophenolate mofetil although the duration of therapy varied [189–192]. Given the paucity of evidence on optimal immunosuppressive agents for the treatment of IgAVN, we suggest the use of mycophenolate mofetil or mizoribine given their favorable side effect profile, until high-quality evidence is available.

*RASB*: RASB (ACE inhibitors or ARBs) are widely used as proteinuria-lowering therapies in children with IgAVN, but there are no prospective studies to support their use or to guide the timing of initiation. Some experts report on the efficacy of early administration whereas others advocate for late introduction to avoid masking the disease activity of IgAVN.

### Antihypertensives

- Recommendation 2b-(viii)
  - We recommend maintaining BP within normal values (systolic BP < 90th percentile for age, sex, and height) (grade B, strong recommendation).
  - In children with CKD stage 2 or higher, we recommend targeting 24-h mean arterial BP ≤ 50th percentile or targeting ≤ 75th percentile for age, sex, and height by ambulatory 24-h BP monitoring in those presenting with UPCR > 0.5 mg/mg (50 mg/mmol) or no proteinuria, respectively (grade B, moderate recommendation).
  - We recommend adding antihypertensive medications of other classes (i.e., non-RASB) to maintain BP within normal levels when optimal BP control (BP < 90th percentile for age, sex, and height, and < 50th percentile for proteinuria > 0.5 mg/mg (50 mg/mmol)) is not obtained with maximum tolerated doses of RASB (grade C, moderate recommendation).

### Other supportive measures

- Recommendation 2b-(ix)
  - We recommend yearly lifelong ongoing monitoring of blood pressure and urinalysis for patients with a history of pediatric IgAVN (grade X, moderate recommendation).

## Diagnosing and treating relapse in IgAVN, and treatment discontinuation

### Definition of remission

Remission of IgAVN: resolution of proteinuria (UPCR < 0.2 mg/mg or 20 mg/mmol or proteinuria < 100 mg/m^2^ per day or < 0.2 g/day in 24-h collection) based on at least two urine samples collected at least 1 month apart in the presence of normal (≥ 90 mL/min/1.73 m^2^) or stable eGFR. Complete remission includes, in addition to these features, the resolution of hematuria, defined as a negative dipstick for blood and/or < 5 RBC/high-power microscopic field (grade X, moderate recommendation).

### Definition of relapse

Relapse of IgAVN: recurrence of hematuria (gross hematuria or ≥ 1 + in dipstick or 5 RBC/ high-power microscopic field) and/or proteinuria (UPCR ≥ 0.2 mg/mg or 20 mg/mmol) in at least two urine samples and/or reduced kidney function (eGFR < 90 mL/min/1.73 m^2^ or > 25% reduction from baseline) in a patient who has achieved a complete remission for at least 1 month (grade X, moderate recommendation).

### Repeat biopsy

- Recommendation 3a
  - We suggest considering a repeat biopsy in case of a relapse in proteinuria ± an unexplained decline in eGFR or when proteinuria persists despite treatment (grade D, weak recommendation).

#### Evidence and rationale

There is no standard definition or consensus on the definition of relapse of IgAVN. Most of the definitions used in the medical literature have been established as inclusion criteria to initiate therapy in specific clinical trials. In general, a relapse of vasculitis has been defined as the recurrence of clinical signs and symptoms or the occurrence of new symptoms after an initial remission, requiring the resumption or the increase of immunosuppressive therapy [163–170]. Many authors consider a relapse of IgAV when a patient who was previously diagnosed with IgAV and who had been asymptomatic for at least 1 month presents with a new flare of cutaneous lesions or with other systemic symptoms [193–198]. Others define a relapse of IgAV when symptoms recur at least 3 months after remission [199, 200]. A relapse of IgAVN can present concomitantly to manifestations of a systemic vasculitis or as a kidney-limited relapse, defining nephritis as the presence of macroscopic or microscopic hematuria (≥ 5 RBC/high-power microscopic field) with or without proteinuria (UPCR > 0.2 mg/mg or 20 mg/mmol) [194].

The Pediatric Vasculitis Activity Score defines proteinuria as urine protein excretion > 0.3 g/day or UPCR > 0.2 mg/mg or 20 mg/mmol and hematuria as a dipstick ≥ 2 + or 5 RBCs/high-power microscopic field or red cell casts and decreased eGFR as a decline of > 25% [201].

### Evaluation of relapses

- Recommendation 3b
  - When treating relapses of IgAVN, we suggest evaluating urine sediment, PCR on the first-morning void, and eGFR (grade D, weak recommendation).

### Treatment of relapses

- Recommendation 3c
  - In children with relapses of IgAVN, we suggest treating relapses following the recommendations for treatment of the initial episode (grade X, moderate recommendation).
  - The decision should take into consideration the response to a specific drug during the initial episode and the toxicity associated with the treatment (grade X, moderate recommendation).

#### Evidence and rationale

There are no universally accepted guidelines/recommendations for the treatment of recurrent IgAVN in native kidneys in children or adults, and no high-quality clinical trial has demonstrated a clear benefit or superiority of a specific agent. One retrospective cohort study showed a reduction in proteinuria with use of oral glucocorticoids and RASB in 7/20 children with IgAVN who had a proteinuric relapse (PCR > 1 mg/mg or 100 mg/mol) after initial remission with oral glucocorticoids (20/20), RASB (15/20), and cyclophosphamide (13/20) [202]. Given the lack of scientific evidence to support any specific drug, participation in clinical trials is encouraged (ongoing studies are listed here: https://clinicaltrials.gov/ct2/results?cond=IgA+Vasculitis&term=&cntry=&state=&city=&dist =).

### Treatment discontinuation

- Recommendation 3d
  - We suggest using immunosuppressive treatment in children with IgAVN for at least 8–12 weeks (grade D, weak recommendation).
  - We suggest discontinuing immunosuppressive treatment in children with IgAVN after at least 4 weeks of remission of proteinuria (PCR < 0.2 mg/mg (20 mg/mmol)) and absence of gross hematuria with normal eGFR (> 90 mL/min/1.73 m^2^) (grade D, weak recommendation).

### Follow-up

- Recommendation 3f-(i)
  - We suggest monitoring children with IgAVN with evaluation of urinalysis, eGFR, and blood pressure for at least 5 years after the initial episode. This follow-up may be conducted by the primary care provider (grade D, weak recommendation).

- Recommendation 3f-(ii)
  - We suggest lifelong monitoring, individualized according to the severity and response to treatment, for children who received therapy (grade D, weak recommendation).

#### Evidence and rationale

There are no studies specifically investigating the optimal time to discontinue treatment in children with IgAVN. Cohort studies provide insight into the rates of remission and timing of relapse. Rates of complete remission in children are reported to be around 32%, with a further 40% found to have minimal or moderate proteinuria with otherwise normal kidney function [203]. Small clinical series report that many of the patients develop remission within the first 3 years [204]. Approximately, 12% of children with IgAVN will relapse between 1 month and 5 years after primary onset, suggesting that although remission in IgAVN is common, a low risk of relapse is still present for several years [196]. Given the potential serious toxicity of many immunosuppressive agents commonly used to treat IgAVN, we think it is reasonable to consider stopping immunosuppressive therapy > 4 weeks after remission has been achieved, for a total treatment duration of at least 8–12 weeks. Treatment with ACEi/ARB may need to be more prolonged. Because of the potential for delayed relapses and the long-term effects of some immunosuppressive agents, intermittent monitoring of urinalysis, eGFR, and BP for a lifetime is suggested for children who received pharmacotherapy or did not achieve complete remission. Compared to IgAN, the treatment for IgAVN can be much shorter; response is generally rapid, and there is no evidence of benefit of prolonged treatment on outcome or on relapse prevention, although some children may be at risk of late decline in kidney function after years of follow-up.

## How should transition be managed in children with either IgAN or IgAVN?

Empowering young people by equipping them with the knowledge and skills to self-manage aspects of their healthcare successfully and confidently underpins the transitional care planning process, which can begin as early as 11 years old.

- We recommend that:
  - Appropriate staff members in adult units are trained to help young people and their parents or caregivers with the transition to adult services.
  - All young people with IgAN and IgAV should have a transition plan, developed between the multi-professional team and the young person.
  - A named transition worker should be allocated.
  - Support should be offered for a minimum of 6 months before and after transfer.
  - Support should include self-management to encourage the young person to make decisions and choices that improve health, well-being, and related behavior, e.g., regularly taking their BP medications and RASB.
  - Young adult-specific information should be provided in adult units.
  - Counseling and career, financial, and benefits advice should be easily accessible.
  - Access to a peer support network should be provided (grade X, moderate recommendation).

## Supplementary Information

Below is the link to the electronic supplementary material.

Supplementary file1 (DOCX 6004 KB)

## References

[1] Wyatt RJ, Julian BA (2013) IgA Nephropathy. N Engl J Med 368:2402–2414. 10.1056/NEJMra1206793 23782179 10.1056/NEJMra1206793

[2] Rovin BH, Adler SG, Barratt J et al (2021) KDIGO 2021 clinical practice guideline for the management of glomerular diseases. Kidney Int 100:S1–S276. 10.1016/j.kint.2021.05.021 34556256 10.1016/j.kint.2021.05.021

[3] Schena FP, Nistor I (2018) Epidemiology of IgA nephropathy: a global perspective. Semin Nephrol 38:435–442. 10.1016/j.semnephrol.2018.05.013 30177015 10.1016/j.semnephrol.2018.05.013

[4] Yeo SC, Cheung CK, Barratt J (2018) New insights into the pathogenesis of IgA nephropathy. Pediatr Nephrol 33:763–777. 10.1007/s00467-017-3699-z 28624979 10.1007/s00467-017-3699-z PMC5861174

[5] Selewski DT, Ambruzs JM, Appel GB et al (2018) Clinical characteristics and treatment patterns of children and adults with IgA nephropathy or IgA vasculitis: findings from the CureGN study. Kidney Int Rep 3:1373–1384. 10.1016/j.ekir.2018.07.021 30450464 10.1016/j.ekir.2018.07.021 PMC6224619

[6] Utsunomiya Y, Koda T, Kado T et al (2003) Incidence of pediatric IgA nephropathy. Pediatr Nephrol 18:511–515. 10.1007/s00467-003-1127-z 12720079 10.1007/s00467-003-1127-z

[7] Willey CJ, Coppo R, Schaefer F et al (2023) The incidence and prevalence of IgA nephropathy in Europe. Nephrol Dial Transplant 38:2340–2349. 10.1093/ndt/gfad082 37156519 10.1093/ndt/gfad082 PMC10539204

[8] Shibano T, Takagi N, Maekawa K et al (2016) Epidemiological survey and clinical investigation of pediatric IgA nephropathy. Clin Exp Nephrol 20:111–117. 10.1007/s10157-015-1129-8 26041644 10.1007/s10157-015-1129-8

[9] Kamei K, Harada R, Hamada R et al (2016) Proteinuria during follow-up period and long-term renal survival of childhood IgA nephropathy. PLoS ONE 11:e0150885. 10.1371/journal.pone.0150885 10.1371/journal.pone.0150885 PMC4792393 26978656

[10] Coppo R, Robert T (2020) IgA nephropathy in children and in adults: two separate entities or the same disease? J Nephrol 33:1219–1229. 10.1007/s40620-020-00725-0 32314305 10.1007/s40620-020-00725-0

[11] Rodrigues JC, Haas M, Reich HN (2017) IgA nephropathy. Clin J Am Soc Nephrol 12:677–686. 10.2215/CJN.07420716 28159829 10.2215/CJN.07420716 PMC5383386

[12] Barbour SJ, Espino-Hernandez G, Reich HN et al (2016) The MEST score provides earlier risk prediction in lgA nephropathy. Kidney Int 89:167–175. 10.1038/ki.2015.322 26759049 10.1038/ki.2015.322

[13] Yata N, Nakanishi K, Shima Y et al (2008) Improved renal survival in Japanese children with IgA nephropathy. Pediatr Nephrol 23:905–912. 10.1007/s00467-007-0726-5 18224344 10.1007/s00467-007-0726-5 PMC2335295

[14] Pitcher D, Braddon F, Hendry B et al (2023) Long-term outcomes in IgA nephropathy. Clin J Am Soc Nephrol 18:727–738. 10.2215/CJN.0000000000000135 37055195 10.2215/CJN.0000000000000135 PMC10278810

[15] Yeo SC, Goh SM, Barratt J (2019) Is immunoglobulin A nephropathy different in different ethnic populations? Nephrology 24:885–895. 10.1111/nep.13592 30977248 10.1111/nep.13592

[16] Kiryluk K, Li Y, Sanna-Cherchi S et al (2012) Geographic differences in genetic susceptibility to IgA nephropathy: GWAS Replication Study and Geospatial Risk Analysis. PLoS Genet 8:e1002765. 10.1371/journal.pgen.1002765 22737082 10.1371/journal.pgen.1002765 PMC3380840

[17] Su B, Jiang Y, Li Z et al (2024) Are children with IgA nephropathy different from adult patients? Pediatr Nephrol 39:2403–2412. 10.1007/s00467-024-06361-1 38578470 10.1007/s00467-024-06361-1 PMC11199250

[18] Sestan M, Jelusic M (2023) Diagnostic and management strategies of iga vasculitis nephritis/Henoch-Schönlein purpura nephritis in pediatric patients: current perspectives. Pediatric Health Med Ther 14:89–98. 10.2147/PHMT.S379862 10.2147/PHMT.S379862 PMC10008002 36915829

[19] Jauhola O, Ronkainen J, Koskimies O et al (2010) Clinical course of extrarenal symptoms in Henoch-Schonlein purpura: a 6-month prospective study. Arch Dis Child 95:871–876. 10.1136/adc.2009.167874 20371584 10.1136/adc.2009.167874

[20] Peruzzi L, Coppo R (2021) IgA Vasculitis nephritis in children and adults: one or different entities? Pediatr Nephrol 36:2615–2625. 10.1007/s00467-020-04818-7 33219450 10.1007/s00467-020-04818-7

[21] Ozen S, Marks SD, Brogan P et al (2019) European consensus-based recommendations for diagnosis and treatment of immunoglobulin A vasculitis—the SHARE initiative. Rheumatology 58:1607–1616. 10.1093/rheumatology/kez041 30879080 10.1093/rheumatology/kez041

[22] Chen Y, Yang K, Marušic A et al (2017) A reporting tool for practice guidelines in health care: the RIGHT statement. Ann Intern Med 166:128–132. 10.7326/M16-1565 27893062 10.7326/M16-1565

[23] Hahn D, Hodson EM, Craig JC (2023) Interventions for preventing and treating kidney disease in IgA vasculitis. Cochrane Database Syst Rev 2023:CD005128. 10.1002/14651858.CD005128.pub4 10.1002/14651858.CD005128.pub4 PMC9972777 36853224

[24] Steering Committee on Quality Improvement and Management (2004) Classifying recommendations for clinical practice guidelines. Pediatrics 114:874–877. 10.1542/peds.2004-1260 15342869 10.1542/peds.2004-1260

[25] Trautmann A, Boyer O, Hodson E et al (2023) IPNA clinical practice recommendations for the diagnosis and management of children with steroid-sensitive nephrotic syndrome. Pediatr Nephrol 38:877–919. 10.1007/s00467-022-05739-3 36269406 10.1007/s00467-022-05739-3 PMC9589698

[26] Schwartz GJ, Brion LP, Spitzer A (1987) The use of plasma creatinine concentration for estimating glomerular filtration rate in infants, children, and adolescents. Pediatr Clin North Am 34:571–590. 10.1016/S0031-3955(16)36251-4 3588043 10.1016/s0031-3955(16)36251-4

[27] Schwartz GJ, Work DF (2009) Measurement and estimation of GFR in children and adolescents. Clin J Am Soc Nephrol 4:1832–1843. 10.2215/CJN.01640309 19820136 10.2215/CJN.01640309

[28] Yoshikawa N, Ito H, Nakamura H (1988) IgA nephropathy in children from Japan. Clinical and Pathological Features. Child Nephrol Urol 9:191–199 3076503

[29] Southwest Pediatric Nephrology Study Group (1982) A multicenter study of IgA nephropathy in children. A report of the Southwest Pediatric Nephrology Study Group. Kidney Int 22:643–652. 10.1038/ki.1982.224 6761487

[30] Rivera F, López-Gómez JM, Pérez-García R (2004) Clinicopathologic correlations of renal pathology in Spain. Kidney Int 66:898–904. 10.1111/j.1523-1755.2004.00833.x 15327378 10.1111/j.1523-1755.2004.00833.x

[31] Kashtan CE (2022) Genetic testing and glomerular hematuria—a nephrologist’s perspective. Am J Med Genet C Semin Med Genet 190:399–403. 10.1002/ajmg.c.31987 35775584 10.1002/ajmg.c.31987 PMC9796064

[32] Wang F, Zhao D, Ding J et al (2012) Skin biopsy is a practical approach for the clinical diagnosis and molecular genetic analysis of X-linked Alport’s syndrome. J Mol Diagn 14:586–593. 10.1016/j.jmoldx.2012.06.005 22921432 10.1016/j.jmoldx.2012.06.005

[33] Nakanishi K, Yoshikawa N, Iijima K et al (1994) Immunohistochemical study of alpha 1–5 chains of type IV collagen in hereditary nephritis. Kidney Int 46:1413–1421. 10.1038/ki.1994.413 7853802 10.1038/ki.1994.413

[34] Trimarchi H, Barratt J, Cattran DC et al (2017) Oxford classification of IgA nephropathy 2016: an update from the IgA nephropathy classification working group. Kidney Int 91:1014–1021. 10.1016/j.kint.2017.02.003 28341274 10.1016/j.kint.2017.02.003

[35] Wu H, Fang X, Xia Z et al (2020) Long-term renal survival and undetected risk factors of IgA nephropathy in Chinese children—a retrospective 1243 cases analysis from single centre experience. J Nephrol 33:1263–1273. 10.1007/s40620-020-00767-4 32507961 10.1007/s40620-020-00767-4

[36] Shima Y, Nakanishi K, Hama T et al (2012) Validity of the Oxford classification of IgA nephropathy in children. Pediatr Nephrol 27:783–792. 10.1007/s00467-011-2061-0 22134880 10.1007/s00467-011-2061-0

[37] Coppo R, Troyanov S, Camilla R et al (2010) The Oxford IgA nephropathy clinicopathological classification is valid for children as well as adults. Kidney Int 77:921–927. 10.1038/ki.2010.43 20200498 10.1038/ki.2010.43

[38] Edstrom Halling S, Soderberg MP, Berg UB (2012) Predictors of outcome in paediatric IgA nephropathy with regard to clinical and histopathological variables (Oxford classification). Nephrol Dial Transplant 27:715–722. 10.1093/ndt/gfr339 21750154 10.1093/ndt/gfr339

[39] Le W, Zeng C-H, Liu Z et al (2012) Validation of the Oxford classification of IgA nephropathy for pediatric patients from China. BMC Nephrol 13:158. 10.1186/1471-2369-13-158 23181565 10.1186/1471-2369-13-158 PMC3519602

[40] Haas M, Rahman MH, Cohn RA et al (2008) IgA nephropathy in children and adults: comparison of histologic features and clinical outcomes. Nephrol Dial Transplant 23:2537–2545. 10.1093/ndt/gfn014 18263928 10.1093/ndt/gfn014

[41] Bergstein J, Leiser J, Andreoli S (2005) The clinical significance of asymptomatic gross and microscopic hematuria in children. Arch Pediatr Adolesc Med 159:353–355 15809388 10.1001/archpedi.159.4.353

[42] Youn T, Trachtman H, Gauthier B (2006) Clinical spectrum of gross hematuria in pediatric patients. Clin Pediatr 45:135–141 10.1177/000992280604500204 16528433

[43] Yuan X, Su Q, Wang H et al (2023) Genetic variants of the COL4A3, COL4A4, and COL4A5 genes contribute to thinned glomerular basement membrane lesions in sporadic IgA nephropathy patients. J Am Soc Nephrol 34:132–144. 10.1681/ASN.2021111447 36130833 10.1681/ASN.2021111447 PMC10101589

[44] Cambier A, Robert T, Hogan J et al (2021) Rare collagenous heterozygote variants in children with IgA nephropathy. Kidney Int Rep 6:1326–1335. 10.1016/j.ekir.2021.02.022 34013111 10.1016/j.ekir.2021.02.022 PMC8116726

[45] Cambier A, Roy J-P, Dossier C et al (2024) IgA nephropathy in children with minimal proteinuria: to biopsy or not to biopsy? Pediatr Nephrol 39:781–787. 10.1007/s00467-023-06121-7 37698655 10.1007/s00467-023-06121-7

[46] Hogg R (2022) An evaluation of the roles of hematuria and uric acid in defining the prognosis of patients with IgA nephropathy. Pediatr Nephrol 37:947–958. 10.1007/s00467-021-05092-x 33982147 10.1007/s00467-021-05092-x

[47] Kincaid-Smith P, Bennett WM, Dowling JP, Ryan GB (1983) Acute renal failure and tubular necrosis associated with hematuria due to glomerulonephritis. Clin Nephrol 19:206–210 6851258

[48] Coppo R (2017) Clinical and histological risk factors for progression of IgA nephropathy: an update in children, young and adult patients. J Nephrol 30:339–346. 10.1007/s40620-016-0360-z 27815919 10.1007/s40620-016-0360-z

[49] Reich HN, Troyanov S, Scholey JW, Cattran DC (2007) Remission of proteinuria improves prognosis in IgA nephropathy. Clin J Am Soc Nephrol 18:3177–3183. 10.1681/ASN.2007050526 10.1681/ASN.2007050526 17978307

[50] Suh J-S, Jang KM, Park YH et al (2020) Remission of proteinuria may protect against progression to chronic kidney disease in pediatric-onset IGA nephropathy. J Clin Med 9:1–17. 10.3390/jcm9072058 10.3390/jcm9072058 PMC7408672 32629965

[51] Inker LA, Lambers-Heerspink H, Tighiouart H et al (2021) Association of treatment effects on early change in urine protein and treatment effects on GFR slope in IgA nephropathy: an individual participant meta-analysis. Am J Kidney Dis 78:340-349.e1. 10.1053/j.ajkd.2021.03.007 33775708 10.1053/j.ajkd.2021.03.007 PMC8384669

[52] Sevillano AM, Gutiérrez E, Yuste C et al (2017) Remission of hematuria improves renal survival in IgA nephropathy. J Am Soc Nephrol 28:3089–3099. 10.1681/ASN.2017010108 28592423 10.1681/ASN.2017010108 PMC5619972

[53] El Karoui K, Fervenza FC, De Vriese AS (2024) Treatment of IgA nephropathy: a rapidly evolving field. J Am Soc Nephrol 35:103–116. 10.1681/ASN.0000000000000242 37772889 10.1681/ASN.0000000000000242 PMC10786616

[54] Zand L, Fervenza FC, Coppo R (2023) Microscopic hematuria as a risk factor for IgAN progression: considering this biomarker in selecting and monitoring patients. Clin Kidney J 16:ii19–ii27. 10.1093/ckj/sfad232 38053974 10.1093/ckj/sfad232 PMC10695511

[55] Shima Y, Nakanishi K, Hama T et al (2013) Spontaneous Remission in Children with IgA Nephropathy. Pediatr Nephrol 28:71–76. 10.1007/s00467-012-2294-6 22940909 10.1007/s00467-012-2294-6

[56] Coppo R (2008) Pediatric IgA nephropathy: clinical and therapeutic perspectives. Semin Nephrol 28:18–26. 10.1016/j.semnephrol.2007.10.003 18222343 10.1016/j.semnephrol.2007.10.003

[57] Moriyama T, Tanaka K, Iwasaki C et al (2014) Prognosis in IgA nephropathy: 30-year analysis of 1,012 patients at a single center in Japan. PLoS ONE 9:e91756. 10.1371/journal.pone.0091756 10.1371/journal.pone.0091756 PMC3962373 24658533

[58] Koyama A, Igarashi M, Kobayashi M (1997) Natural History and Risk Factors for Immunoglobulin A Nephropathy in Japan. Am J Kidney Dis 29:526–532. 10.1016/S0272-6386(97)90333-4 9100040 10.1016/s0272-6386(97)90333-4

[59] Worawichawong S, Plumworasawat S, Liwlompaisan W et al (2021) Distribution pattern of mesangial C4d deposits as predictor of kidney failure in IgA nephropathy. PLoS ONE 16:e0252638. 10.1371/journal.pone.0252638 10.1371/journal.pone.0252638 PMC8174712 34081719

[60] Yoshikawa N, Tanaka R, Iijima K (2001) Pathophysiology and treatment of IgA nephropathy in children. Pediatr Nephrol 16:446–457. 10.1007/s004670100582 11405121 10.1007/s004670100582

[61] Calderon-Margalit R, Golan E, Twig G et al (2018) History of childhood kidney disease and risk of adult end-stage renal disease. N Engl J Med 378:428–438. 10.1056/NEJMoa1700993 29385364 10.1056/NEJMoa1700993

[62] Stern-Zimmer M, Calderon-Margalit R, Skorecki K, Vivante A (2021) Childhood risk factors for adulthood chronic kidney disease. Pediatr Nephrol 36:1387–1396. 10.1007/s00467-020-04611-6 32500249 10.1007/s00467-020-04611-6

[63] Yap HK, Teo S, Ng KH (2022) Pediatric nephrology: on-the-go, illustrated edn. World Scientific, Singapore

[64] Barbour SJ, Coppo R, Er L et al (2021) Updating the international IgA nephropathy prediction tool for use in children. Kidney Int 99:1439–1450. 10.1016/j.kint.2020.10.033 33220356 10.1016/j.kint.2020.10.033

[65] Lurbe E, Agabiti-Rosei E, Cruickshank JK et al (2016) 2016 European Society of Hypertension Guidelines for the Management of High Blood Pressure in Children and Adolescents. J Hypertens 34:1887–1920. 10.1097/HJH.0000000000001039 27467768 10.1097/HJH.0000000000001039

[66] Pattrapornpisut P, Avila-Casado C, Reich HN (2021) IgA nephropathy: core curriculum 2021. Am J Kidney Dis 78:429–441. 10.1053/j.ajkd.2021.01.024 34247883 10.1053/j.ajkd.2021.01.024

[67] Coppo R, Peruzzi L, Amore A et al (2007) IgACE: a placebo-controlled, randomized trial of angiotensin-converting enzyme inhibitors in children and young people with IgA nephropathy and moderate proteinuria. J Am Soc Nephrol 18:1880–1888. 10.1681/ASN.2006040347 17513327 10.1681/ASN.2006040347

[68] Nakanishi K, Iijima K, Ishikura K et al (2009) Efficacy and safety of lisinopril for mild childhood IgA nephropathy: a pilot study. Pediatr Nephrol 24:845–849. 10.1007/s00467-008-1006-8 18825420 10.1007/s00467-008-1006-8

[69] Shima Y, Nakanishi K, Sako M et al (2019) Lisinopril versus lisinopril and losartan for mild childhood IgA nephropathy: a randomized controlled trial (JSKDC01 study). Pediatr Nephrol 34:837–846. 10.1007/s00467-018-4099-8 30284023 10.1007/s00467-018-4099-8

[70] Higa A, Shima Y, Hama T et al (2015) Long-term outcome of childhood IgA nephropathy with minimal proteinuria. Pediatr Nephrol 30:2121–2127. 10.1007/s00467-015-3176-5 26238276 10.1007/s00467-015-3176-5

[71] Thompson A, Carroll K, Inker L et al (2019) Proteinuria reduction as a surrogate end point in trials of IgA nephropathy. Clin J Am Soc Nephrol 14:469–481. 10.2215/CJN.08600718 30635299 10.2215/CJN.08600718 PMC6419287

[72] Coppo R (2021) Treatment of IgA nephropathy in children: a land without KDIGO guidance. Pediatr Nephrol 36:491–496. 10.1007/s00467-020-04486-7 32060820 10.1007/s00467-020-04486-7

[73] Shen W, Chen H, Chen H et al (2010) Obesity-related glomerulopathy: body mass index and proteinuria. Clin J Am Soc Nephrol 5:1401–1409. 10.2215/CJN.01370210 20498244 10.2215/CJN.01370210 PMC2924407

[74] Kittiskulnam P, Kanjanabuch T, Tangmanjitjaroen K et al (2014) The beneficial effects of weight reduction in overweight patients with chronic proteinuric immunoglobulin A nephropathy: a randomized controlled trial. J Ren Nutr 24:200–207. 10.1053/j.jrn.2014.01.016 24759301 10.1053/j.jrn.2014.01.016

[75] Kataoka H, Ohara M, Shibui K et al (2012) Overweight and obesity accelerate the progression of IgA nephropathy: prognostic utility of a combination of BMI and histopathological parameters. Clin Exp Nephrol 16:706–712. 10.1007/s10157-012-0613-7 22350469 10.1007/s10157-012-0613-7

[76] Yin T, Chen Y, Tang L et al (2022) Relationship between modifiable lifestyle factors and chronic kidney disease: a bibliometric analysis of top-cited publications from 2011 to 2020. BMC Nephrol 23:120–135. 10.1186/s12882-022-02745-3 35337272 10.1186/s12882-022-02745-3 PMC8957172

[77] Pozzi C, Bolasco PG, Fogazzi GB et al (1999) Corticosteroids in IgA nephropathy: a randomised controlled trial. Lancet 353:883–887. 10.1016/s0140-6736(98)03563-6 10093981 10.1016/s0140-6736(98)03563-6

[78] Pozzi C, Andrulli S, Del Vecchio L et al (2004) Corticosteroid effectiveness in IgA nephropathy: long-term results of a randomized, controlled trial. J Am Soc Nephrol 15:157–163. 10.1097/01.ASN.0000103869.08096.4F 14694168 10.1097/01.asn.0000103869.08096.4f

[79] Manno C, Torres DD, Rossini M et al (2009) Randomized controlled clinical trial of corticosteroids plus ACE-inhibitors with long-term follow-up in proteinuric IgA nephropathy. Nephrol Dial Transplant 24:3694–3701. 10.1093/ndt/gfp356 19628647 10.1093/ndt/gfp356

[80] Lv J, Zhang H, Wong MG et al (2017) Effect of oral methylprednisolone on clinical outcomes in patients with IgA nephropathy: the TESTING randomized clinical trial. JAMA 318:432–442. 10.1001/jama.2017.9362 28763548 10.1001/jama.2017.9362 PMC5817603

[81] Rauen T, Eitner F, Fitzner C et al (2015) Intensive supportive care plus immunosuppression in IgA nephropathy. N Engl J Med 373:2225–2236. 10.1056/NEJMoa1415463 26630142 10.1056/NEJMoa1415463

[82] Rauen T, Wied S, Fitzner C et al (2020) After ten years of follow-up, no difference between supportive care plus immunosuppression and supportive care alone in IgA nephropathy. Kidney Int 98:1044–1052. 10.1016/j.kint.2020.04.046 32450154 10.1016/j.kint.2020.04.046

[83] Lv J, Wong MG, Hladunewich MA et al (2022) Effect of oral methylprednisolone on decline in kidney function or kidney failure in patients with IgA nephropathy: the TESTING randomized clinical trial. JAMA 327:1888–1898. 10.1001/jama.2022.5368 35579642 10.1001/jama.2022.5368 PMC9115617

[84] Niaudet P, Murcia I, Beaufils H et al (1993) Primary IgA nephropathies in children: prognosis and treatment. Adv Nephrol Necker Hosp 22:121–140 8427055

[85] Welch TR, Fryer C, Shely E et al (1992) Double-blind, controlled trial of short-term prednisone therapy in immunoglobulin A glomerulonephritis. J Pediatr 121:474–477. 10.1016/S0022-3476(05)81808-6 1517929 10.1016/s0022-3476(05)81808-6

[86] Waldo FB, Wyatt RJ, Kelly DR et al (1993) Treatment of IgA nephropathy in children: efficacy of alternate-day oral prednisone. Pediatr Nephrol 7:529–532 8251315 10.1007/BF00852535

[87] Hogg RJ, Lee J, Nardelli N et al (2006) Clinical trial to evaluate omega-3 fatty acids and alternate day prednisone in patients with IgA nephropathy: report from the Southwest Pediatric Nephrology Study Group. Clin J Am Soc Nephrol 1:467–474. 10.2215/CJN.01020905 17699247 10.2215/CJN.01020905

[88] Yoshikawa N, Ito H, Sakai T et al (1999) A controlled trial of combined therapy for newly diagnosed severe childhood iga nephropathy. J Am Soc Nephrol 10:101–109. 10.1681/ASN.V101101 9890315 10.1681/ASN.V101101

[89] Yoshikawa N, Honda M, Iijima K et al (2006) Steroid treatment for severe childhood IgA nephropathy: a randomized, controlled trial. Clin J Am Soc Nephrol 1:511–517. 10.2215/CJN.01120905 17699253 10.2215/CJN.01120905

[90] Kamei K, Nakanishi K, Ito S et al (2011) Long-term results of a randomized controlled trial in childhood IgA nephropathy. Clin J Am Soc Nephrol 6:1301–1307. 10.2215/CJN.08630910 21493743 10.2215/CJN.08630910 PMC3109925

[91] Shima Y, Nakanishi K, Kamei K et al (2011) Disappearance of glomerular IgA deposits in childhood iga nephropathy showing diffuse mesangial proliferation after 2 years of combination/ prednisolone therapy. Nephrol Dial Transplant 26:163–169. 10.1093/ndt/gfq387 20601366 10.1093/ndt/gfq387

[92] Kawasaki Y, Maeda R, Kanno S et al (2017) Long-term follow up of pediatric immunoglobulin A nephropathy treated with tonsillectomy plus methylprednisolone pulse therapy. Pediatr Int 59:41–47. 10.1111/ped.13074 27341677 10.1111/ped.13074

[93] Cambier A, Rabant M, Peuchmaur M et al (2018) immunosuppressive treatment in children with IgA nephropathy and the clinical value of podocytopathic features. Kidney Int Rep 3:916–925. 10.1016/j.ekir.2018.03.013 29988999 10.1016/j.ekir.2018.03.013 PMC6035143

[94] Ikezumi Y, Suzuki T, Imai N et al (2006) Histological differences in new-onset IgA nephropathy between children and adults. Nephrol Dial Transplant 21:3466–3474 16935895 10.1093/ndt/gfl455

[95] Coppo R (2019) Pediatric IgA nephropathy in Europe. Kidney Dis 5:182–188. 10.1159/000495751 10.1159/000495751 PMC6587208 31259180

[96] Pozzi C, Andrulli S, Pani A et al (2010) Addition of azathioprine to corticosteroids does not benefit patients with IgA nephropathy. J Am Soc Nephrol 21:1783–1790. 10.1681/ASN.2010010117 20634300 10.1681/ASN.2010010117 PMC3013548

[97] Ballardie FW, Roberts ISD (2002) Controlled prospective trial of prednisolone and cytotoxics in progressive IgA nephropathy. J Am Soc Nephrol 13:142–148. 10.1681/ASN.V131142 11752031 10.1681/ASN.V131142

[98] Sarcina C, Tinelli C, Ferrario F et al (2016) Changes in proteinuria and side effects of corticosteroids alone or in combination with azathioprine at different stages of IgA nephropathy. Clin J Am Soc Nephrol 11:973–981. 10.2215/CJN.02300215 27129712 10.2215/CJN.02300215 PMC4891742

[99] Yoshikawa N (2008) Treatment of childhood IgA nephropathy. Nippon Jinzo Gakkai Shi 50:468–472 18546876

[100] Shima Y, Nakanishi K, Kaku Y et al (2018) Combination therapy with or without warfarin and dipyridamole for severe childhood IgA nephropathy: an RCT. Pediatr Nephrol 33:2103–2112. 10.1007/s00467-018-4011-6 29987456 10.1007/s00467-018-4011-6

[101] Hou J-H, Le W-B, Chen N et al (2017) Mycophenolate mofetil combined with prednisone versus full-dose prednisone in IgA nephropathy with active proliferative lesions: a randomized controlled trial. Am J Kidney Dis 69:788–795. 10.1053/j.ajkd.2016.11.027 28215945 10.1053/j.ajkd.2016.11.027

[102] Hogg RJ, Bay RC, Jennette JC et al (2015) Randomized controlled trial of mycophenolate mofetil in children, adolescents, and adults with IgA nephropathy. Am J Kidney Dis 66:783–791. 10.1053/j.ajkd.2015.06.013 26209543 10.1053/j.ajkd.2015.06.013

[103] Hou FF, Xie D, Wang J et al (2023) Effectiveness of mycophenolate mofetil among patients with progressive IgA nephropathy: a randomized clinical trial. JAMA Netw Open 6:e2254054. 10.1001/jamanetworkopen.2022.54054 36745456 10.1001/jamanetworkopen.2022.54054 PMC12578496

[104] Han SY, Jung C-Y, Lee SH et al (2022) A multicenter, randomized, open-label, comparative, phase IV study to evaluate the efficacy and safety of combined treatment with mycophenolate mofetil and corticosteroids in advanced immunoglobulin A nephropathy. Kidney Res Clin Pract 41:452–461. 10.23876/j.krcp.21.146 35545228 10.23876/j.krcp.21.146 PMC9346400

[105] Lafayette RA, Canetta PA, Rovin BH et al (2017) A randomized, controlled trial of rituximab in IgA nephropathy with proteinuria and renal dysfunction. J Am Soc Nephrol 28:1306–1313. 10.1681/ASN.2016060640 27821627 10.1681/ASN.2016060640 PMC5373458

[106] Crayne CB, Eloseily E, Mannion ML et al (2018) Rituximab treatment for chronic steroid-dependent Henoch-Schonlein purpura: 8 cases and a review of the literature. Pediatr Rheumatol Online J 16:71. 10.1186/s12969-018-0285-2 30428889 10.1186/s12969-018-0285-2 PMC6236882

[107] Song Y-H, Cai G-Y, Xiao Y-F et al (2017) Efficacy and safety of calcineurin inhibitor treatment for IgA nephropathy: a meta-analysis. BMC Nephrol 18:61–69. 10.1186/s12882-017-0467-z 28193247 10.1186/s12882-017-0467-z PMC5307812

[108] Liu L-J, Yang Y, Shi S-F et al (2019) Effects of hydroxychloroquine on proteinuria in IgA nephropathy: a randomized controlled trial. Am J Kidney Dis 74:15–22. 10.1053/j.ajkd.2019.01.026 30922594 10.1053/j.ajkd.2019.01.026

[109] Selvaskandan H, Barratt J, Cheung CK (2024) Novel treatment paradigms: primary IgA nephropathy. Kidney Int Rep 9:203–213. 10.1016/j.ekir.2023.11.026 38344739 10.1016/j.ekir.2023.11.026 PMC10851020

[110] US Food and Drug Administration (2021) FDA approves first drug to decrease urine protein in IgA nephropathy, a rare kidney disease. https://www.fda.gov/drugs/fda-approves-first-drug-decrease-urine-protein-iga-nephropathy-rare-kidney-disease. Accessed 15 May 2024

[111] Rovin BH, Barratt J, Heerspink HJL et al (2023) Efficacy and safety of sparsentan versus irbesartan in patients with IgA nephropathy (PROTECT): 2-year results from a randomised, active-controlled, phase 3T trial. Lancet 402:2077–2090. 10.1016/S0140-6736(23)02302-4 37931634 10.1016/S0140-6736(23)02302-4

[112] Zhang H, Rizk DV, Perkovic V et al (2024) Results of a randomized double-blind placebo-controlled phase 2 study propose iptacopan as an alternative complement pathway inhibitor for IgA nephropathy. Kidney Int 105:189–199. 10.1016/j.kint.2023.09.027 37914086 10.1016/j.kint.2023.09.027

[113] Williams J (2023) Omeros corporation provides update on interim analysis of ARTEMIS-IGAN phase 3 trial of narsoplimab in IgA nephropathy. https://investor.omeros.com/news-releases/news-release-details/omeros-corporation-provides-update-interim-analysis-artemis-igan. Accessed 15 May 2024

[114] Caster DJ, Lafayette RA (2024) The treatment of primary IgA nephropathy: change, change, change. Am J Kidney Dis 83:229–240. 10.1053/j.ajkd.2023.08.007 37742867 10.1053/j.ajkd.2023.08.007

[115] Li X-H, Huang X-P, Pan L et al (2016) Vitamin D deficiency may predict a poorer outcome of IgA nephropathy. BMC Nephrol 17:164. 10.1186/s12882-016-0378-4 27806690 10.1186/s12882-016-0378-4 PMC5094030

[116] Xiaowei L, Bo W, Li L, Peng Z (2020) Comparison of the effects of valsartan plus activated vitamin D versus valsartan alone in IgA nephropathy with moderate proteinuria. Int Urol Nephrol 52:129–136. 10.1007/s11255-019-02329-5 31768803 10.1007/s11255-019-02329-5

[117] Deng J, Zheng X, Xie H, Chen L (2017) Calcitriol in the treatment of IgA nephropathy with non-nephrotic range proteinuria: a meta-analysis of randomized controlled trials. Clin Nephrol 87:21–27. 10.5414/CN108915 27900938 10.5414/CN108915

[118] Liu L-J, Lv J-C, Shi S-F et al (2012) Oral calcitriol for reduction of proteinuria in patients with IgA nephropathy: a randomized controlled trial. Am J Kidney Dis 59:67–74. 10.1053/j.ajkd.2011.09.014 22019331 10.1053/j.ajkd.2011.09.014

[119] Zan J, Ma J, Man Q et al (2022) Safety evaluation of COVID-19 vaccine in patients with IgA nephropathy or IgA vasculitis nephritis. Kidney Int Rep 7:1435–1436. 10.1016/j.ekir.2022.03.025 35399860 10.1016/j.ekir.2022.03.025 PMC8977377

[120] Hanna C, Hernandez LPH, Bu L et al (2021) IgA nephropathy presenting as macroscopic hematuria in 2 pediatric patients after receiving the Pfizer COVID-19 vaccine. Kidney Int 100:705–706. 10.1016/j.kint.2021.06.032 34237324 10.1016/j.kint.2021.06.032 PMC8256683

[121] Ma Y, Xu G (2022) New-onset IgA nephropathy following COVID-19 vaccination. QJM 116:26–39. 10.1093/qjmed/hcac185 10.1093/qjmed/hcac185 PMC9450102 35920797

[122] Donadio JV, Bergstralh EJ, Offord KP et al (1994) a controlled trial of fish oil in IgA nephropathy. J Am Soc Nephrol 331:1194–1199. 10.1056/NEJM199411033311804 10.1056/NEJM199411033311804 7935657

[123] Donadio JVJ, Grande JP, Bergstralh EJ et al (1999) The long-term outcome of patients with IgA nephropathy treated with fish oil in a controlled trial. J Am Soc Nephrol 10:1772–1777. 10.1681/ASN.V1081772 10446945 10.1681/ASN.V1081772

[124] Donadio JV, Larson TS, Bergstralh EJ, Grande JP (2001) A randomized trial of high-dose compared with low-dose omega-3 fatty acids in severe IgA nephropathy. J Am Soc Nephrol 12:791–799. 10.1681/ASN.V124791 11274240 10.1681/ASN.V124791

[125] Shen X-H, Liang S-S, Chen H-M et al (2015) Reversal of active glomerular lesions after immunosuppressive therapy in patients with IgA nephropathy: a repeat-biopsy based observation. J Nephrol 28:441–449. 10.1007/s40620-014-0165-x 25585823 10.1007/s40620-014-0165-x

[126] Shima Y, Nakanishi K, Sato M et al (2017) IgA nephropathy with presentation of nephrotic syndrome at onset in children. Pediatr Nephrol 32:457–465. 10.1007/s00467-016-3502-6 27714465 10.1007/s00467-016-3502-6

[127] Kang Z, Li Z, Duan C et al (2015) Mycophenolate mofetil therapy for steroid-resistant IgA nephropathy with the nephrotic syndrome in children. Pediatr Nephrol 30:1121–1129. 10.1007/s00467-014-3041-y 25773534 10.1007/s00467-014-3041-y PMC4446504

[128] Pradhan SK, Sivaraj P, Das L, Swain A (2015) Spectrum of clinico-pathological profile and treatment response in children with nephrotic immunoglobulin A nephropathy. Saudi J Kidney Dis Transpl 26:708–711. 10.4103/1319-2442.160149 26178542 10.4103/1319-2442.160149

[129] Yang D, He L, Peng X et al (2016) The efficacy of tonsillectomy on clinical remission and relapse in patients with IgA nephropathy: a randomized controlled trial. Renal Fail 38:242–248. 10.3109/0886022X.2015.1128251 10.3109/0886022X.2015.1128251 26727697

[130] Suzuki T, Yamamoto T, Ohura M et al (2004) Clinicopathologic findings relevant to disappearance or relapse of proteinuria following corticosteroid treatment in IgA nephropathy patients with proteinuria of 0.5 to 2.0 g/day. Clin Exp Nephrol 8:243–249. 10.1007/s10157-004-0299-6 15480902 10.1007/s10157-004-0299-6

[131] Shima Y, Mukaiyama H, Tanaka Y et al (2024) Factors Related To Recurrence Of Proteinuria In Childhood Iga Nephropathy. Pediatr Nephrol 39:463–471. 10.1007/s00467-023-06116-4 37594578 10.1007/s00467-023-06116-4

[132] Haas M (1997) Histologic subclassification of IgA nephropathy: a clinicopathologic study of 244 cases. Am J Kidney Dis 29:829–842. 10.1016/S0272-6386(97)90456-X 9186068 10.1016/s0272-6386(97)90456-x

[133] Gutierrez E, Rojas-Rivera J, Praga M et al (2012) Long-term outcomes of IgA nephropathy presenting with minimal or no proteinuria. J Am Soc Nephrol 23:1753–1760. 10.1681/ASN.2012010063 22956820 10.1681/ASN.2012010063 PMC3458461

[134] Antonucci L, Fuiano L, Gargiulo A et al (2024) Childhood-onset IgA nephropathy: is long-term recovery possible? Pediatr Nephrol 39:1837–1846. 10.1007/s00467-023-06259-4 38225439 10.1007/s00467-023-06259-4

[135] Coppo R, Lofaro D, Camilla RR et al (2017) Risk factors for progression in children and young adults with IgA nephropathy: an analysis of 261 cases from the VALIGA European cohort. Pediatr Nephrol 32:139–150. 10.1007/s00467-016-3469-3 27557557 10.1007/s00467-016-3469-3

[136] D’Amico G (2004) Natural history of idiopathic IgA nephropathy and factors predictive of disease outcome. Semin Nephrol 24:179–196. 10.1016/j.semnephrol.2004.01.001 15156525 10.1016/j.semnephrol.2004.01.001

[137] Cochat P, Fargue S, Mestrallet G et al (2009) Disease recurrence in paediatric renal transplantation. Pediatr Nephrol 24:2097–2108. 10.1007/s00467-009-1137-6 19247694 10.1007/s00467-009-1137-6 PMC2753770

[138] Moroni G, Belingheri M, Frontini G et al (2019) Immunoglobulin A nephropathy Recurrence after renal transplantation. Front Immunol 10:1332. 10.3389/fimmu.2019.01332 31275309 10.3389/fimmu.2019.01332 PMC6593081

[139] Suzuki K, Honda K, Tanabe K et al (2003) Incidence of latent mesangial IgA deposition in renal allograft donors in Japan. Kidney Int 63:2286–2294. 10.1046/j.1523-1755.63.6s.2.x 12753320 10.1046/j.1523-1755.63.6s.2.x

[140] Gaber LW, Khan FN, Graviss EA et al (2020) Prevalence, characteristics, and outcomes of incidental IgA glomerular deposits in donor kidneys. Kidney Int Rep 5:1914–1924. 10.1016/j.ekir.2020.08.018 33163712 10.1016/j.ekir.2020.08.018 PMC7609995

[141] Silva FG, Chander P, Pirani CL, Hardy MA (1982) Disappearance of glomerular mesangial IgA deposits after renal allograft transplantation. Transplantation 33:241–246 7036478

[142] Odum J, Peh CA, Clarkson AR et al (1994) Recurrent mesangial IgA nephritis following renal transplantation. Nephrol Dial Transplant 9:309–312 8052439

[143] Berger J (1988) Recurrence of IgA nephropathy in renal allografts. Am J Kidney Dis 12:371–372. 10.1016/s0272-6386(88)80027-1 3055960 10.1016/s0272-6386(88)80027-1

[144] Fisher NC, Nightingale PG, Gunson BK et al (1998) Chronic renal failure following liver transplantation: a retrospective analysis. Transplantation 66:59–66. 10.1097/00007890-199807150-00010 9679823 10.1097/00007890-199807150-00010

[145] Bumgardner GL, Amend WC, Ascher NL, Vincenti FG (1998) Single-center long-term results of renal transplantation for IgA nephropathy. Transplantation 65:1053–1060 9583865 10.1097/00007890-199804270-00008

[146] Choy BY, Chan TM, Lo SK et al (2003) Renal transplantation in patients with primary immunoglobulin A nephropathy. Nephrol Dial Transplant 18:2399–2404. 10.1093/ndt/gfg373 14551373 10.1093/ndt/gfg373

[147] Floege J (2004) Recurrent IgA nephropathy after renal transplantation. Semin Nephrol 24:287–291. 10.1016/j.semnephrol.2004.01.008 15156532 10.1016/j.semnephrol.2004.01.008

[148] Ohmacht C, Kliem V, Burg M et al (1997) Recurrent immunoglobulin A nephropathy after renal transplantation: a significant contributor to graft loss. Transplantation 64:1493–1496. 10.1097/00007890-199711270-00024 9392321 10.1097/00007890-199711270-00024

[149] Ponticelli C (2001) The activity of the division of nephrology and dialysis. Osp Magg 95:116–119

[150] Briganti EM, Russ GR, McNeil JJ et al (2002) Risk of renal allograft loss from recurrent glomerulonephritis. N Engl J Med 347:103–109. 10.1056/NEJMoa013036 12110738 10.1056/NEJMoa013036

[151] Clayton P, McDonald S, Chadban S (2011) Steroids and recurrent IgA nephropathy after kidney transplantation. Am J Transplant 11:1645–1649. 10.1111/j.1600-6143.2011.03667.x 21797974 10.1111/j.1600-6143.2011.03667.x

[152] Leeaphorn N, Garg N, Khankin EV et al (2018) Recurrence of IgA nephropathy after kidney transplantation in steroid continuation versus early steroid-withdrawal regimens: a retrospective analysis of the UNOS/OPTN database. Transpl Int 31:175–186. 10.1111/tri.13075 28926143 10.1111/tri.13075 PMC5762402

[153] Di Vico MC, Messina M, Fop F et al (2018) Recurrent IgA nephropathy after renal transplantation and steroid withdrawal. Clin Transplant 32:e13207. 10.1111/ctr.13207 29345747 10.1111/ctr.13207

[154] Andreoli SP, Bergstein JM (1989) Treatment of severe IgA nephropathy in children. Pediatr Nephrol 3:248–253. 10.1007/BF00858524 2702102 10.1007/BF00858524

[155] Eckardt K-U, Kasiske BL, Zeier MG (2009) Special issue: KDIGO Clinical Practice Guideline for the Care of Kidney Transplant Recipients. Am J of Transplant 9:S1–S155. 10.1111/j.1600-6143.2009.02834.x 10.1111/j.1600-6143.2009.02834.x 19845597

[156] Oka K, Imai E, Moriyama T et al (2000) A clinicopathological study of IgA nephropathy in renal transplant recipients: beneficial effect of angiotensin-converting enzyme inhibitor. Nephrol Dial Transplant 15:689–695. 10.1093/ndt/15.5.689 10809812 10.1093/ndt/15.5.689

[157] Courtney AE, McNamee PT, Nelson WE, Maxwell AP (2006) Does angiotensin blockade influence graft outcome in renal transplant recipients with IgA nephropathy? Nephrol Dial Transplant 21:3550–3554. 10.1093/ndt/gfl506 16968729 10.1093/ndt/gfl506

[158] Glassock RJ (2021) IgA nephropathy: “The Times They Are a-Changin.” Glomerular Dis 2:4–14. 10.1159/000515199 36751269 10.1159/000515199 PMC9677731

[159] Jennette JC, Falk RJ, Bacon PA et al (2013) 2012 Revised International Chapel Hill Consensus Conference Nomenclature of Vasculitides. Arthritis Rheum 65:1–11. 10.1002/art.37715 23045170 10.1002/art.37715

[160] Hunder GG (1998) The use and misuse of classification and diagnostic criteria for complex diseases. Ann Intern Med 129:417–418. 10.7326/0003-4819-129-5-199809010-00013 9735071 10.7326/0003-4819-129-5-199809010-00013

[161] Robson J, Grayson P, Ponte C et al (2022) 2022 American College of Rheumatology/European Alliance of Associations for Rheumatology Classification Criteria for Granulomatosis with Polyangiitis. Ann Rheum Dis 81:315–320. 10.1136/annrheumdis-2021-221795 35110333 10.1136/annrheumdis-2021-221795

[162] Mills JA, Michel BA, Bloch DA et al (1990) The American College of Rheumatology 1990 Criteria for the Classification of Henoch-Schönlein Purpura. Arthritis Rheum 33:1114–1121. 10.1002/art.1780330809 2202310 10.1002/art.1780330809

[163] Ozen S, Pistorio A, Iusan SM et al (2010) EULAR/PRINTO/PRES criteria for Henoch-Schönlein purpura, childhood polyarteritis nodosa, childhood wegener granulomatosis and childhood takayasu arteritis: Ankara 2008. Part II: Final Classification Criteria. Ann Rheum Dis 69:798–806. 10.1136/ard.2009.116657 20413568 10.1136/ard.2009.116657

[164] Linskey KR, Kroshinsky D, Mihm MC, Hoang MP (2012) Immunoglobulin-A–associated small-vessel vasculitis: a 10-year experience at the Massachusetts General Hospital. J Am Acad Dermatol 66:813–822. 10.1016/j.jaad.2011.06.012 21798626 10.1016/j.jaad.2011.06.012

[165] Haas M, Jafri J, Bartosh SM et al (2000) ANCA-associated crescentic glomerulonephritis with mesangial IgA deposits. Am J Kidney Dis 36:709–718. 10.1053/ajkd.2000.17615 11007672 10.1053/ajkd.2000.17615

[166] Coppo R, Andrulli S, Amore A et al (2006) Predictors of outcome in Henoch-Schonlein nephritis in children and adults. Am J Kidney Dis 47:993–1003 16731294 10.1053/j.ajkd.2006.02.178

[167] Ronkainen J, Nuutinen M, Koskimies O (2002) The adult kidney 24 years after childhood Henoch-Schönlein purpura: a retrospective cohort study. Lancet 360:666–670. 10.1016/S0140-6736(02)09835-5 12241872 10.1016/S0140-6736(02)09835-5

[168] Koskela M, Ylinen E, Ukonmaanaho E-M et al (2017) The ISKDC classification and a new semiquantitative classification for predicting outcomes of Henoch-Schönlein purpura nephritis. Pediatr Nephrol 32:1201–1209. 10.1007/s00467-017-3608-5 28197887 10.1007/s00467-017-3608-5

[169] Barbour SJ, Coppo R, Er L et al (2024) Histologic and clinical factors associated with kidney outcomes in IgA vasculitis nephritis. Clin J Am Soc Nephrol 19:438–451. 10.2215/CJN.0000000000000398 38261310 10.2215/CJN.0000000000000398 PMC11020428

[170] Narchi H (2005) Risk of long term renal impairment and duration of follow up recommended for Henoch-Schönlein purpura with normal or minimal urinary findings: a systematic review. Arch Dis Child 90:916–920. 10.1136/adc.2005.074641 15871983 10.1136/adc.2005.074641 PMC1720564

[171] Kawasaki Y, Suzuki J, Sakai N et al (2003) Clinical and pathological features of children with Henoch-Schoenlein Purpura nephritis: risk factors associated with poor prognosis. Clin Nephrol 60:153–160 14524577 10.5414/cnp60153

[172] Edström Halling S, Söderberg MP, Berg UB (2009) Treatment of severe Henoch-Schönlein and immunoglobulin A nephritis A single center experience. Pediatr Nephrol 24:91–97. 10.1007/s00467-008-0990-z 18931859 10.1007/s00467-008-0990-z

[173] Goldstein AR, White RHR, Akuse R, Chantler C (1992) Long-term follow-up of childhood Henoch-Schönlein nephritis. Lancet 339:280–282. 10.1016/0140-6736(92)91341-5 1346291 10.1016/0140-6736(92)91341-5

[174] Counahan R, Winterborn MH, White RH et al (1977) Prognosis of Henoch-Schönlein nephritis in children. Br Med J 2:11–14. 10.1136/bmj.2.6078.11 871734 10.1136/bmj.2.6078.11 PMC1631306

[175] Dudley J, Smith G, Llewelyn-Edwards A et al (2013) Randomised, double-blind, placebo-controlled trial to determine whether steroids reduce the incidence and severity of nephropathy in Henoch-Schönlein purpura (HSP). Arch Dis Child 98:756–763. 10.1136/archdischild-2013-303642 23845696 10.1136/archdischild-2013-303642

[176] Huber AM, King J, McLaine P et al (2004) A randomized, placebo-controlled trial of prednisone in early Henoch Schönlein purpura [ISRCTN85109383]. BMC Med 2:7. 10.1186/1741-7015-2-7 15059282 10.1186/1741-7015-2-7 PMC400510

[177] Ronkainen J, Ala-Houhala M, Autio-Harmainen H et al (2006) Long-term outcome 19 years after childhood IgA nephritis: a retrospective cohort study. Pediatr Nephrol 21:1266–1273. 10.1007/s00467-006-0163-x 16838184 10.1007/s00467-006-0163-x

[178] Peratoner L, Longo F, Lepore L, Freschi P (1990) Prophylaxis and therapy of glomerulonephritis in the course of anaphylactoid purpura: the results of a polycentric clinical trial. Acta Paediatr 79:976–977. 10.1111/j.1651-2227.1990.tb11365.x 10.1111/j.1651-2227.1990.tb11365.x 2264475

[179] Yoshimoto M, Ito H, Shindo S, Yamashita F (1987) Evaluation of the preventive role of dipyridamole and aspirin against renal complication in Schonlein-Henoch purpura [abstract]. Pediatr Nephrol 1:C47

[180] Tian M, Liu C (2015) Heparin calcium treated Henoch-Schönlein purpura nephritis in children through inhibiting hyperfibrinolysis. Ren Fail 37:1100–1104. 10.3109/0886022X.2015.1061668 26133741 10.3109/0886022X.2015.1061668

[181] Wu S-H, Liao P-Y, Chen X-Q et al (2014) Add-on therapy with montelukast in the treatment of Henoch-Schönlein purpura. Pediatr Int 56:315–322. 10.1111/ped.12271 24299021 10.1111/ped.12271

[182] Niaudet P, Habib R (1998) Methylprednisolone pulse therapy in the treatment of severe forms of schönlein-henoch purpura nephritis. Pediatr Nephrol 12:238–243. 10.1007/s004670050446 9630046 10.1007/s004670050446

[183] Tarshish P, Bernstein J, Edelmann CM (2004) Henoch-Schönlein purpura nephritis: course of disease and efficacy of cyclophosphamide. Pediatr Nephrol 19:51–56. 10.1007/s00467-003-1315-x 14634864 10.1007/s00467-003-1315-x

[184] Jauhola O, Ronkainen J, Autio-Harmainen H et al (2011) Cyclosporine A vs. methylprednisolone for Henoch-Schönlein nephritis: a randomized trial. Pediatr Nephrol 26:2159–2166. 10.1007/s00467-011-1919-5 21626222 10.1007/s00467-011-1919-5

[185] Zhang H, Li X, Xu H et al (2021) Effect and safety evaluation of tacrolimus and tripterygium glycosides combined therapy in treatment of Henoch-Schönlein purpura nephritis. Int J Urol 28:1157–1163. 10.1111/iju.14665 34378238 10.1111/iju.14665

[186] Ninchoji T, Kaito H, Nozu K et al (2011) Treatment strategies for Henoch-Schonlein purpura nephritis by histological and clinical severity. Pediatr Nephrol 26:563–569. 10.1007/s00467-010-1741-5 21203777 10.1007/s00467-010-1741-5

[187] Nagai S, Horinouchi T, Ninchoji T et al (2023) Long-term outcome of combination therapy with corticosteroids, mizoribine and RAS inhibitors as initial therapy for severe childhood IgA vasculitis with nephritis. Pediatr Nephrol 38:4023–4031. 10.1007/s00467-023-06052-3 37380934 10.1007/s00467-023-06052-3

[188] Lu Z, Song J, Mao J et al (2017) Evaluation of mycophenolate mofetil and low-dose steroid combined therapy in moderately severe Henoch-Schönlein purpura nephritis. Med Sci Monit 23:2333–2339. 10.12659/msm.904206 28515415 10.12659/MSM.904206 PMC5444683

[189] Hackl A, Becker JU, Korner LM et al (2018) Mycophenolate mofetil following glucocorticoid treatment in Henoch-Schonlein purpura nephritis: the role of early initiation and therapeutic drug monitoring. Pediatr Nephrol 33:619–629. 10.1007/s00467-017-3846-6 29177628 10.1007/s00467-017-3846-6

[190] Kallash M, Vogt BA, Zeid A et al (2022) The scope of treatment of pediatric IgA vasculitis nephritis and its outcome: a pediatric nephrology research consortium study. Pediatr Nephrol 37:2687–2697. 10.1007/s00467-022-05496-3 35233641 10.1007/s00467-022-05496-3

[191] Kurt-Şükür ED, Tullus K (2022) Response to: timely and individualized use of immunosuppression is associated with favourable outcomes in paediatric IgA vasculitis nephritis. Pediatr Nephrol 37:915–915. 10.1007/s00467-021-05416-x 35048165 10.1007/s00467-021-05416-x

[192] Du Y, Hou L, Zhao C et al (2012) Treatment of children with Henoch-Schonlein purpura nephritis with mycophenolate mofetil. Pediatr Nephrol 27:765–771. 10.1007/s00467-011-2057-9 22081165 10.1007/s00467-011-2057-9

[193] Blanco R, Martínez-Taboada VM, Rodríguez-Valverde V et al (1997) Henoch-Schönlein purpura in adulthood and childhood: two different expressions of the same syndrome. Arthritis Rheum 40:859–864. 10.1002/art.1780400513 9153547 10.1002/art.1780400513

[194] Shin JI, Park JM, Shin YH et al (2006) Predictive factors for nephritis, relapse, and significant proteinuria in childhood Henoch-Schönlein purpura. Scand J Rheumatol 35:56–60. 10.1080/03009740510026841 16467044 10.1080/03009740510026841

[195] Gökçe Ş, Kurugöl Z, Koturoğlu G, Aslan A (2020) Predictive role of laboratory markers and clinical features for recurrent Henoch-Schönlein purpura in childhood: a study from Turkey. Mod Rheumatol 30:1047–1052. 10.1080/14397595.2019.1690966 31711347 10.1080/14397595.2019.1690966

[196] Rigante D, Candelli M, Federico G et al (2005) Predictive factors of renal involvement or relapsing disease in children with Henoch-Schönlein purpura. Rheumatol Int 25:45–48. 10.1007/s00296-004-0452-2 15007622 10.1007/s00296-004-0452-2

[197] Ekinci RMK, Balci S, Melek E et al (2020) Clinical manifestations and outcomes of 420 children with Henoch Schönlein purpura from a single referral center from Turkey: a three-year experience. Mod Rheumatol 30:1039–1046. 10.1080/14397595.2019.1687074 31662011 10.1080/14397595.2019.1687074

[198] Calvo-Río V, Hernández JL, Ortiz-Sanjuán F et al (2016) Relapses in patients with Henoch-Schönlein purpura: analysis of 417 patients from a single center. Medicine 95:e4217. 10.1097/MD.0000000000004217 27428226 10.1097/MD.0000000000004217 PMC4956820

[199] Karadağ ŞG, Tanatar A, Sönmez HE et al (2019) The clinical spectrum of Henoch-Schönlein purpura in children: a single-center study. Clin Rheumatol 38:1707–1714. 10.1007/s10067-019-04460-1 30734116 10.1007/s10067-019-04460-1

[200] Lei W-T, Tsai P-L, Chu S-H et al (2018) Incidence and risk factors for recurrent Henoch-Schönlein purpura in children from a 16-year nationwide database. Pediatr Rheumatol Online J 16:25. 10.1186/s12969-018-0247-8 29661187 10.1186/s12969-018-0247-8 PMC5902957

[201] Dolezalova P, Price-Kuehne FE, Özen S et al (2013) Disease activity assessment in childhood vasculitis: development and preliminary validation of the paediatric vasculitis activity score (PVAS). Ann Rheum Dis 72:1628–1633. 10.1136/annrheumdis-2012-202111 23100606 10.1136/annrheumdis-2012-202111

[202] Pirojsakul K, Tangnararatchakit K, Chalermsanyakorn P, Tapaneya-Olarn W (2012) Clinical outcome of children with Henoch-Schönlein purpura nephritis. J Med Assoc Thai 95:878–883 22919981

[203] Coppo R, Mazzucco G, Cagnoli L et al (1997) Long-term prognosis of Henoch-Schönlein nephritis in adults and children. Italian Group of Renal Immunopathology Collaborative Study on Henoch-Schönlein Purpura. Nephrol Dial Transplant 12:2277–2283. 10.1093/ndt/12.11.2277 9394311 10.1093/ndt/12.11.2277

[204] Delbet J-D, Geslain G, Auger M et al (2020) Histological prognostic factors in children with Henoch-Schönlein purpura nephritis. Pediatr Nephrol 35:313–320. 10.1007/s00467-019-04363-y 31696358 10.1007/s00467-019-04363-y

