# Supplementary material for: IPNA clinical practice recommendations for the diagnosis and management of children with IgA nephropathy and IgA vasculitis nephritis
Source: Pediatr Nephrol. 2024 Sep 27;40(2):533–69. doi: 10.1007/s00467-024-06502-6 (PMC11666671; doi:10.1007/s00467-024-06502-6)
Supplement: Supplementary file 1 — Supplementary file1 (DOCX 6004 KB) [file 467_2024_6502_MOESM1_ESM.docx]

**IPNA Clinical Practice Recommendations for the Diagnosis and Management of Children with IgA Nephropathy and IgA Vasculitis Nephritis**

**Supplementary Materials**

| **Table Number** | **Title** | **Page Number** |
| --- | --- | --- |
| S1 | Guideline Expert Panel | 2 |
| S2 | IgA Nephropathy Randomized Controlled Trials | 4 |
| S3 | IgA Nephropathy  Observational Studies | 14 |
| S4 | Major Differential Diagnosis of Dominant/Codominant Glomerular IgA Deposits. | 27 |
| S5 | The Oxford (MEST-C) Classification of IgA Nephropathy | 28 |
| S6 | Studies Validating Oxford (MEST-C) Classification in Pediatric Populations. | 29 |
| S7 | IgA Vasculitis Nephritis  Interventions – randomized controlled trials | 31 |
| S8 | IgA Vasculitis relapse therapy – observational studies | 138 |
| S9 | Search Strategy IgAN/IgAVN | 173 |

**Table S1. Guideline Committee Members, Affiliation, Role and Expertise**

| **Team Member** | **Institution, Country** | **Role** | **Expertise** |
| --- | --- | --- | --- |
| Marina Vivarelli | Bambino Gesù Children's Hospital, Italy | Chair | Pediatric Nephrology |
| Koichi Nakanishi | University of the Ryukyus, Japan | Co-Chair | Pediatric Nephrology |
| Olivia Boyer | Paris Descartes University, France | Core group Member | Pediatric Nephrology |
| Dieter Haffner | Hannover Medical School, Germany | Core group Member | Pediatric Nephrology |
| Keisha Gibson | University of North Carolina at Chapel Hill, United States | Core group Member | Pediatric Nephrology |
| Jonathan Barrett | University of Leicester, UK | Core group Member | Adult Nephrology & IgAN KDIGO lead |
| Melvin Bonilla-Felix | University of Puerto Rico, Puerto Rico | Core group Member | Pediatric Nephrology |
| Rosanna Coppo | Fondazione Ricerca Molinette, Regina Margherita Hospital, Italy | Core group Member | Pediatric Nephrology |
| Mark Haas | Cedars-Sinai Medical Center, United States | Core group Member | Pediatric Nephrology |
| Susan Samuel | University of Calgary, Canada | Evidence review Team leader and Core group member | Epidemiology, Pediatric Nephrology |
| Hernan Trimarchi | Hospital Britanico de Buenos Aires, Argentina | Core group Member | Adult Nephrology |
| Marta Adragna | Hospital Garrahan, Argentina | Core group Member | Pediatric Nephrology |
| Paul Brogan | University College London Great Ormond Street Institute of Child Health, England | Core group Member | Pediatric Nephrology and Rheumatology |
| Maher Ahmed Abdel-Hafez | Tanta University, Egypt | Core group Member | Pediatric Nephrology |
| Zhi-Hong Liu | Nanjing University School of Medicine, China | Core group Member | Pediatric Nephrology |
| Isaac Liu Desheng | Raffles Hospital, Singapore | Core group Member | Pediatric Nephrology |
| Masaki Shimizu | Tokyo Medical and Dental University, Japan | Core group Member | Pediatric Nephrology and Rheumatology |
| Qian Shen | Children's Hospital of Fudan University, China | Core group Member | Pediatric Nephrology |
| Siah Kim | Children's Hospital at Westmead, Australia | Core group Member | Pediatric Nephrology and Clinical Epidemiology |
| Yuko Shima | Wakayama Medical University, Japan | Core group Member | Pediatric Nephrology |
| Mukta Mantan | University of Delhi, India | Core group Member | Pediatric Nephrology |
| Areefa Alladin | University of Calgary, Canada | Evidence Review Team | Epidemiology, Systematic Review |
| Ken Pfister | University of Calgary, Canada | Evidence review team | Epidemiology, Systematic Review |
| Bonnie Schneider | IgA Nephropathy Foundation | Patient representative |  |
| Rachel McDonald |  | Patient representative |  |
| Elisabeth Hodson | Cochrane Kidney and Transplant | Non-voting Core group member | Pediatric Nephrology and Clinical Epidemiology |
| Deirdre Hahn | Children's Hospital at Westmead, Australia | Non-voting Core group member | Pediatric Nephrology and Clinical Epidemiology |

**Table S2. IgA Nephropathy Randomized Controlled Trials**

| 1st , 2nd Author, Year, Country of Origin | Title of Publication and citation | Study Design and setting | Keywords | Participants | Intervention and Comparator | Outcomes |
| --- | --- | --- | --- | --- | --- | --- |
| Kawasaki, Y., Takano, K., 2006, Japan | Efficacy of Tonsillectomy Pulse Therapy versus Multiple-Drug Therapy for IgA Nephropathy  Kawasaki Y., Tanako, K., et al. Pediatric Nephrol. 2006 21: 1701-1706. doi: 10.1007/s00467-006-0272-6. | Prospective randomized controlled trial.  Multicenter (4)  Inclusion criteria:   1. Diagnosis of diffuse IgAN via renal biopsy in the absence of systemic disease (HSPN) 2. Age at start of therapy <15 years 3. No previous treatment with corticosteroids or immunosuppressive drugs   Study groups:  Group 1: Tonsillectomy plus pulse therapy  Group 2: PWDM alone | IgA nephropathy, Prednisolone, Mizoribine, Tonsillectomy plus methylprednisolone pulse, Children | N= 32 analyzed  Group 1 (Tonsillectomy + pulse therapy):  n=16/16 analyzed  Group 2 (PWDM):  n=16/16 analyzed  Mean age:  Group 1: 13 (SD ± 2 yrs)  Group 2: 11.3 (SD ± 3 yrs)  Sex (M/F): Group 1: 9/7 Group 2: 8/8 | Group 1 (n=16):  Tonsillectomy + prednisolone, warfarin, and dipyridamole including methylprednisolone pulse therapy (three courses, each consisting of high-dose methylprednisolone, 20-25 mg/kg/day for 3 days).  Prednisolone given orally at a dose of 2 mg/kg/day (3 times a day, not to exceed 60 mg/day) for 2 weeks – followed by 1.5 mg/kg/day for 2 weeks, 1.0 mg/kg/day for 4 weeks, 0.5 mg/kg/day for 4 weeks, 1.0 mg/kg/2 days for 9 months, and 0.5 mg/kg/2 days for 21 months.  Warfarin given orally in a single morning dose of 1-2 mg/day.  Dipyridamole given orally at a dose of 5 mg/kg/day, 3 times a day (max 300 mg) for 24 months.  Group 2 (n=16):  PWD given in same regimen as G1.  Mizoribine given orally at a dose of 5 mg/kg/day, 2 times a day for 24 months. | After 6-months of therapy  Group 1:  Mean urinary protein excretion decreased from 97 ± 45 mg/m^2^/h to 26 ± 15 mg/m^2^/h (p<0.05)  Group 2:  Mean urinary protein excretion decreased from 93 ± 36 mg/m^2^/h to 25 ± 17 mg/m^2^/h (p<0.05)  After 24-months of therapy  Group 1:  Mean urinary protein excretion was 13 ± 12 mg/m^2^/h  Group 2:  Mean urinary protein excretion was 12 ± 8 mg/m^2^/h  Differences in mean urinary protein excretion between the two groups after 6-months and 24-months was not significant.  At most recent follow-up  Group 1:  Mean urinary protein excretion was 8 ± 8 mg/m^2^/h  Group 2:  Mean urinary protein excretion was 10 ± 8 mg/m^2^/h  The difference between groups was not significant.  The difference between the groups in the incidence of hematuria, serum albumin and creatinine levels was not significant.  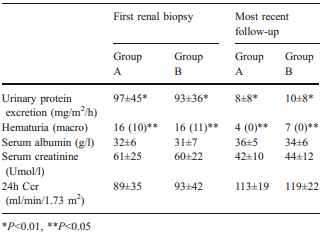  Clinical stages  Group 1:  Recovered clinically: n=12  Minor urinary abnormalities: n=4  Persistent Nephropathy: n=0  Renal insufficiency: n=0  Group 2:  Recovered clinically: n=9  Minor urinary abnormalities: n=6  Persistent Nephropathy: n=1  Renal insufficiency: n=0  Pathological findings of the tonsils (G1 only)  Peritonsillar abscess: n=14  Chronic tonsillitis: n=16  Adverse events  No significant differences between the groups in incidence of cushingoid changes, glaucoma, or hypertension.  3 patients in G2 developed hyperuricemia.  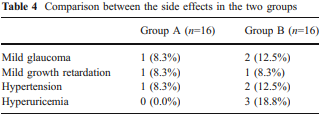  0 patients in Group 1 & 6 patients in Group 2 experienced an acute exacerbation of IgAN as a result of tonsillitis (p<0.05). |
| Welch T, Fryer c et al. USA (Ohio) | Double-blind controlled trial of short-term prednisone therapy in Immunoglobulin a glomerulonephritis: Journal of Pediatrics 1992;121(3):474-477 | Double Blind RCT: 1st course of Tx assigned by random number table.  Patients & investigators blinded. | IgA nephropathy, Prednisone | N=20 children  Sex (M/F): 15/5  Mean age: 13 yrs (SD not given)  Ethnicity: 1 Black  Inclusion  All patients with biopsy proven IgA nephropathy diagnosed at Children's Hospital Medical Center in 1983-1989.  Exclusion  1). sCr>140µmol  2). HTN | Each patient randomized to either steroid or placebo as first therapy  a) 3 mo. steroids separated by  b) 3 mo. rest period then  c) 3 mo. placebo given in an identical manner to the steroid course.  Steroid protocol  Prednisolone 2mg/kg/d x 2 wk then every 2d x 10 wk.  There was no comparison group.  Split person design (patients were their own control) | Proteinuria  No significant changes in urine protein excretion in either phase  Prednisolone phase (mg/hr):  Change from 28 to 25 (p=0.19 within group)  Placebo phase (mg/hr):  Change from 16 to 25 (p=0.12 within group)  Serum creatinine  Mean sCr at entry was 70 umol/L and at exit was 60 umol/L (at end of 9 mo).  Data not stratified by treatment phases.  Hematuria  No significant changes in hematuria  Prednisolone phase (mg/hr):  Change from 7.5 to 4.1 (p=0.29 within group)  Placebo phase (mg/hr):  Change from 1.4 to 1.9 (p=0.42 within group)  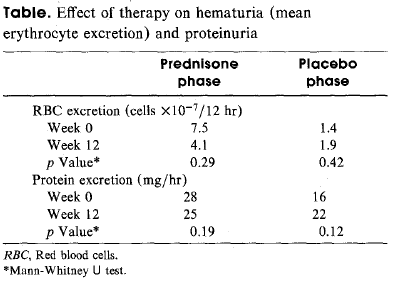  Adverse events  No serious side effects detected in either phase. |
| Kamei K, Nakanishi K, Japan, 2011 | Long-term results of a randomized controlled trial in childhood IgA nephropathy.  Clin J Am Soc Nephrol. 2011 Jun; 6(6):1301-7. doi:10.2215/CJN.08630910. | Prospective randomized controlled trial  20 Japanese renal centres  Objective: Rreport the long-term outcome of patients who were included in [an earlier] trial and elucidate the effectiveness of the combination therapy in childhood IgA nephropathy with diffuse mesangial proliferation  78 eligible children > 15 years old with biopsy-proven IgA nephropathy + diffuse mesangial proliferation, between 1990 and 1993.  Inclusion and exclusion criteria were similar to those used in Yoshikawa et al. 1999  Outcomes:  1) primary = ESRF needing replacement therapy  2) renal insufficiency via eGFR < 60 ml/min per 1.73 m^2^  3) proteinuria: urinary protein  amount per day (g/1.73 m^2^ per day) during the initial 2-year study period  *After that, the early morning urinary  protein/creatinine ratio (uP/Cr) was used  Heavy, mild, and no proteinuria were defined as ≥1.0, 0.2 to 1.0 and <0.1 g/1.73 m^2^ per day, or ≥1.0, 0.2 to 1.0 and <0.2 g/g |  | N= 78 analyzed  Group 1 (combination)  n=40  Group 2 (control)  n=38  Mean age:  Group 1: 12.2 ± 3.0 yrs Group 2: 11.6 +/- 2.3 yrs  Sex (M/F): Group 1: 22/18 Group 2: 29/9 | Group 1: prednisolone,  azathioprine, heparin-warfarin, and dipyridamole  for 24 months  Group 2: only heparin-warfarin and dipyridamole for 24 months  Prednisolone: orally, 2 mg/kg per day (maximum, 80 mg/d) in three divided  doses for 4 weeks, followed by 2 mg/kg given as a single  dose in the morning of every other day for 4 weeks, 1.5  mg/kg every other day for 4 weeks, and 1 mg/kg every  other day for 21 months  Azathioprine: Orally, 2 mg/kg per day as a single morning dose for 24 months  Heparin: Continuous intravenous infusion in sufficient doses to keep the partial thromboplastin  time at 60 seconds for 28 days, followed  by oral warfarin given as a single morning dose to maintain the thrombotest at 30 to 50% for 23 months  Dipyridamole: orally, 5 mg/kg per day (400 mg/d) in three divided doses for 24 months  After the trial  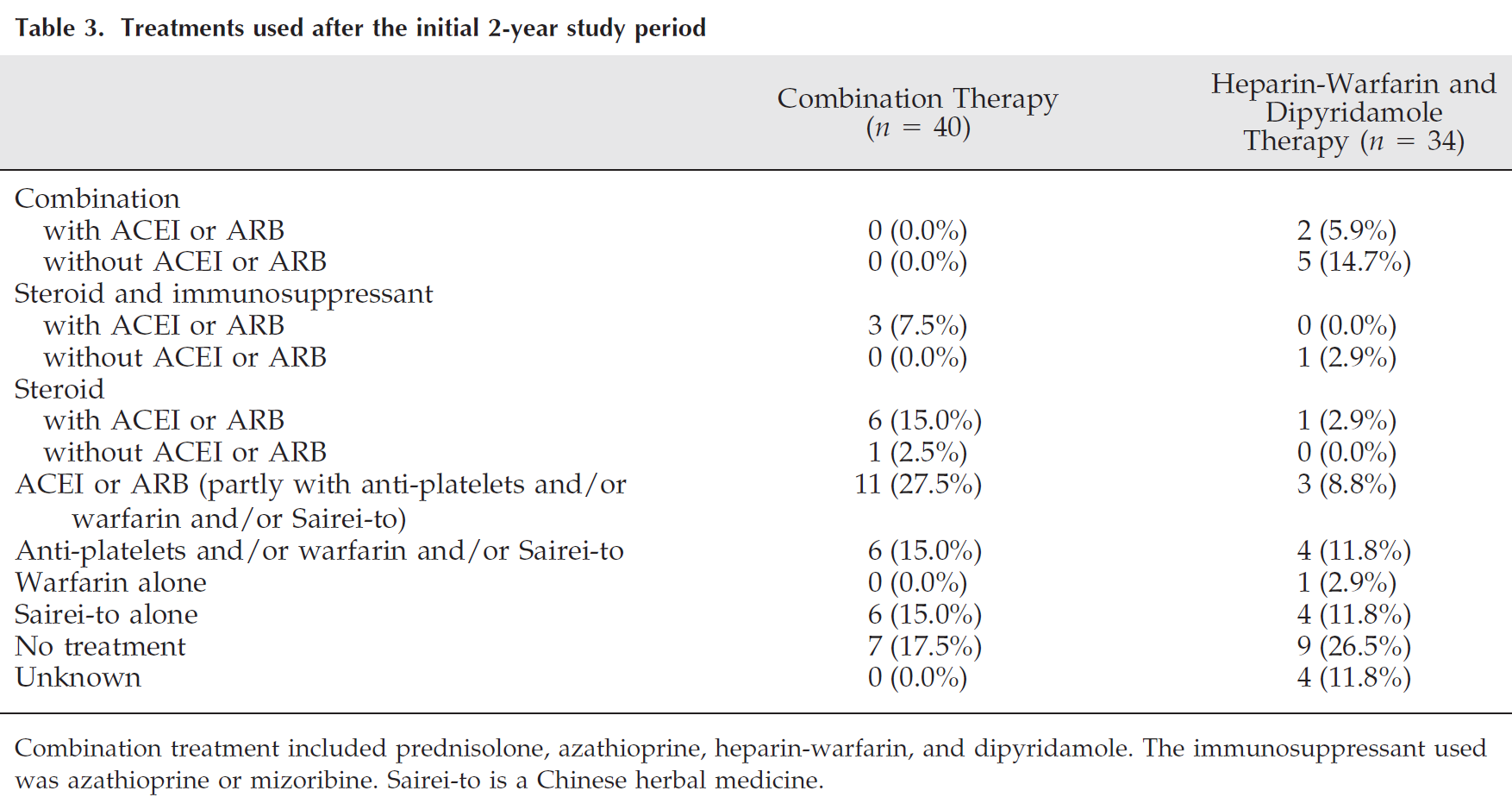 | Primary (at the end of the 2-year trial)  Urinary protein excretion  Group 1: from 2.09 +/- 1.78 g/1.73 m^2^/day to 0.28 +/- 0.36 g/1.73 m^2^/day  Group 2: from 1.35 +/- 1.26 g/1.73 m^2^/day to 1.07 +/- 1.57 g/1.73 m^2^/day  CrC  Group 1: from 144 +/- 52 ml/min per 1.73 m^2^ to 147 +/- 33 ml/min per 1.73 m^2^  Group 2: from 152 +/- 47 ml/min per 1.73 m^2^ to 145 +/- 44 ml/min per 1.73 m^2^  Differences in CrC between groups were non-significant at the start of the trial (p=0.35) and after (p=0.89)  Differences in PER had a p=0.02 at the start of the trial and p= 0.01 after  Significant differences within groups were not commented on in this paper (referring to Yoshikawa et al. 1999 may be informative)  After the 2-year trial 2 (5.0%) patients in group 1 and 10 (23.5%) patients in group 2 (p=0.009) had heavy proteinuria and 20 (50.0%) patients from group 1 and 6 (17.6%) patients from group 2 (p=0.007) had no proteinuria  After the trial (N=74 out of 78 initially)  No proteinuria:  Group 1=24 (60.0%)  Group 2=18 (52.9%)  Mild proteinuria:  Group 1=12 (30.0%)  Group 2=6 (17.6%)  Heavy proteinuria:  Group 1=1 (2.5%)  Group 2=4 (11.8%)  Renal insufficiency:  Group 1=1 (2.5%)  Group 2=1 (2.9%)  ESRF:  Group 1=2 (5.0%)  Group 2=5 (14.7%) |
| Shima, Nakanishi. 2018. Japan | Combination therapy with or without warfarin and dipyridamole for severe childhood IgA nephropathy: a RCT.  Pediatr Nephrol. 2018; 33(11):2103–12. | Non-double-blinded RCT involving Japanese pediatric renal centers  (Japanese Pediatric IgAN Treatment group) | IgA nephropathy, Immunosuppression, Corticosteroids, Anticoagulants, Antiplatelet | N=71 pts  Inclusion  1) Newly diagnosed severe IgAN (Diffuse mesangial proliferation biopsy).  2) 2-18 yr at study entry  3) Protein > 0.3g/dL on first void  4) Low serum protein <60 mmol/L  5) Sufficient biopsy specimen (≥10 glom)  6) No prior immunosuppression  Exclusion  IgAV, SLE, Secondary IgAN | Group 1 (n=34)  Sex M/F: 10/24  Protocol:  -prednisolone, mizoribine, warfarin, and dipyridamole x 24 mo.  Prednisolone:  2 mg/kg/d x 4wk,  then 2 mg/kg EOD x 4 wk, then 1.5 mg/kg EOD x 4 wk, then 1 mg/kg EOD x 21 mo  Mizoribine:  4 mg/kg/d x 24 mo  Warfarin:  OD to maintain the  thrombotest at 20–50% x 24 mo.  Dipyridamole:  6 mg/kg/ d x 24  Group 2 (n=36)  Sex M/F: 24/12  Protocol: Prednisolone and mizoribine for 24 mo (according to dose in group 1) | Primary:  Disappearance of proteinuria  a) Dichotomous outcome (first void UPCR < 0.2 g/dL).  At end of 2-yr treatment, 30/34 (88.2%) of gp 1 and 27/36 (75.0%) of gp 2 reached primary endpoint.  b) Time to disappearance of proteinuria (mo), significantly shorter in gp 1 (log rank p=0.04)  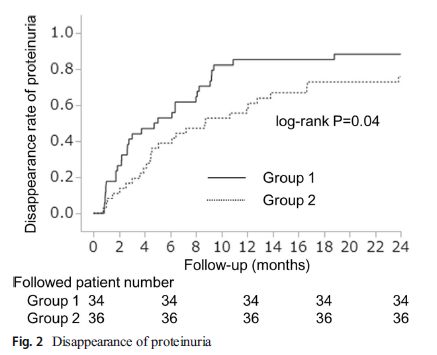  Secondary:  **Change in UPC  Group 1: Median UP excretion reduced from 0.88 g/m2 /d to 0.09 g/m2/d (p < 0.0001).  Group 2: median UP reduced from 0.81 g/m2/d to 0.12 g/m2/d (P < 0.0001).  Biopsy changes  33/34 in group 1 and 36/36 in group 2 had re-biopsy.  a) Change in % of sclerosed glomeruli:  global sclerosis was increased in group 1 when compared to group 2  start end p  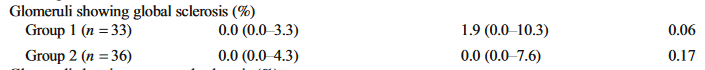  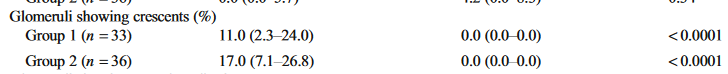  Crescents was significantly decreased in both groups  (P < 0.0001).  Side effects:  Obesity was most common SE in both groups due to steroid. (p=0.85)  Headache in group 1 (p=0.008)  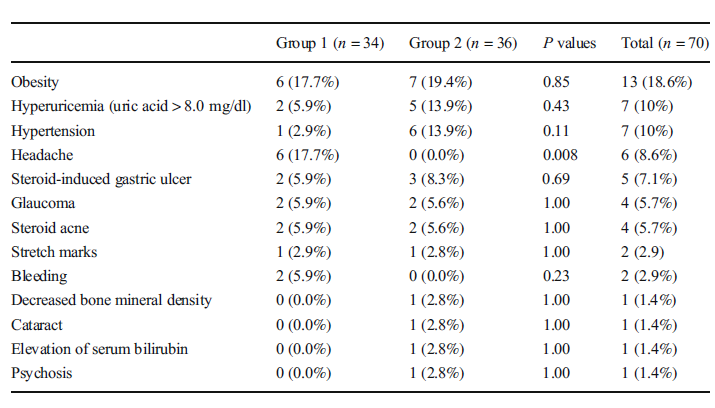  eCrCl and mean BMI unchanged in both groups.  At the end of 2 yr., there was no significant difference between the two groups in clinical and pathological findings |
| Yoshikawa, Ito, 1999 Japan | A Controlled Trial of Combined Therapy for Newly Diagnosed Severe Childhood IgA Nephropathy.  J Am Soc Nephrol 10: 101–109, 1999 | RCT, 20 Japanese Pediatric centers (Japanese Pediatric IgAN Treatment group). Randomization was done by a sealed envelope technique in blocks of 4. |  | Inclusion (N=78)  1) Newly diagnosed IgAN  2) biopsy between 1990-1993 showing IgAN and diffuse mesangial proliferation  3) Age≤15 yrs at study entry  4) No previous immunosuppression  5) sufficient renal biopsy (≥10 glom)  Exclusion  IgAV, SLE | Group 1 (n=40)  Sex M/F: 22/28  Prednisolone, azathioprine,  heparin-warfarin, and dipyridamole x 24 mo  Prednisolone: 2 mg/kg/d x 4wk  then 2 mg/kg EOD x 4 wk, then 1.5 mg/kg EOD x 4 wk,  then 1 mg/kg EOD x 21 mo  Aza: 2mg/kg/d x 24 mo  Heparin iv to keep PTT at 60sec x 1mo then  Warfarin: OD to maintain  thrombotest at 30–50% x 23 mo.  Dipyridamole: 5 mg/kg/ TID x 24mo  (n=38 at end)  Group 2 (n=38)  Sex M/F:29/9  Heparin-warfarin, and dipyridamole x 24 mo according to protocol for group 1 (n=33 at end) | Followed for 2 yr.  Change in Proteinuria  Within groups  Group 1: 1.35 ± 1.01 g/d at start, 0.22 ± 0.31at end (p<0.001)  Group 2: 0.98 ± 0.99 g/d at start, 0.88 ± 1.34 (p0.16)    Change in Hematuria  Group1: 2.9 ± 0.8 at start, 0.5 ± 1.0 at end (p<0.001)  Group 2: 2.7 ± 1.0 at start, 1.5 ± 1.1 at end (p=0.0002)  Serum IgA  Group 1: 290 ± 115 mg/dl at start, 229 ± 87 mg/dl (p<0.0001)  Group 2: 280 ± 100 mg/dl at start, 281 ± 92 mg/dl (p0.88) unchanged  Pathology (%)  Group 1: repeat biopsy n=35  Sclerosis: 5.2 ±7.7 at start; 5± 6.9 at end (p0.65)  Crescents: 24.5 ±20.9 at start; 0.4± 1.1 at end (p<0.001)  Group 2: repeat biopsy n=28  Sclerosis: 3.9 ±6.1 at start; 16.4±23 at end (p0.006)  Crescents: 21.5 ±18.2 at start; 4.4± 10.3 at end (p<0.001)  Adverse events:  Group 1:  Alopecia (n=1) due to Aza  Anaemia (n=1) due to Aza  Leukopenia (n=30  Glaucoma (n=1)  Cataract (n=1)  Depression (n=21)  Peptic ulcer (n=1)  Increase Liver enzyme (n=1)  Mean SD score for height reduced from 20.01± 1.01 at the start to -0.31 ± 1.16 at the end (P 0.001).  Mean obesity score increased from - 4.0 ± 11.4% to 4.6 ± 12.6% (P< 0.0001)  Group 2:  Bleeding (n=2) due to warfarin  Increase Liver enzyme (n=1)  Urticaria (n=1)  No reduction in height noted (p=0.1)  Mean obesity score increased from 3.4 ± 19.3% to 7.3 ±21.3%  (P 0.03) |
| Yoshikawa, Honda. 2006, Japan | Steroid Treatment for Severe Childhood IgA Nephropathy:  A Randomized, Controlled Trial.  Clin J Am Soc Nephrol 1: 511–517, 2006. | Unblinded RCT.  20 Japanese Pediatric centers (Japanese Pediatric IgAN Treatment group). Randomization was done by a sealed envelope technique in blocks of four. |  | Inclusion (N=80)  1) Newly diagnosed IgAN  2) biopsy between 1994-1998 showing IgAN and diffuse mesangial proliferation  3) Age≤15 yrs at study entry  4) No previous immunosuppression  5) sufficient renal biopsy (≥10 glom)  Exclusion  IgAV, SLE, | Group 1 (n=40)  Sex M/F: 22/18  Prednisolone, azathioprine,  warfarin, and dipyridamole x 24 mo  Prednisolone: 2 mg/Kg/d x 4w  Then 2 mg/kg EOD x 4 w, then 1.5 mg/kg EOD x 4 w, then 1 mg/kg EOD x 21 m  Aza: 2mg/kg/d x 24 m  Warfarin: OD to maintain the  thrombotest at 30–50% x 23 m.  Dipyridamole: 5 mg/kg/ TID x 24mo  (n=39 at end)  Group 2 (n=40)  Sex M/F:21/19  Prednisolone as per group 1 protocol  (n=39 at end) | Primary outcome  Disappearance of proteinuria (<0.1g/m2/d) – Kaplan Meier  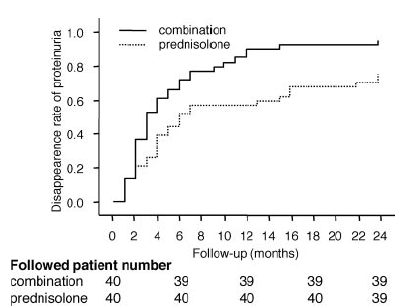  Between group difference was 17.9% (95%CI 1.8-34)  Disappearance was significantly higher in Gp1 (Log rank P=0.007)  Secondary outcomes  Change in urine protein excretion  Group 1: Protein changed from 1.29g/m2/d to 0.1 (p<0.0001)  Group 2: protein changed from 1.16 to 0.12 (P=0.0001)  No significance between groups  Change in % of sclerosed glomeruli (mean SD)  Group 1: 32/40 had repeat biopsy  Group 2: 30/40 had repeat biopsy  Group 1:  Global Sclerosis: 5 (9.1) to 4.6 (16.5) p= 0.74  Crescents: 17.3 (16.6) to 1.7 (3) p<0.001  Group 2:  Global Sclerosis: 3.1(4.8) to 14.6(15.2) p=0.0003  Crescents: 19.1 (17.1) to 0.9 (1.9) p<0.0001  Adverse events:  10 pts in each group had AE  Total # of events in group 1=14  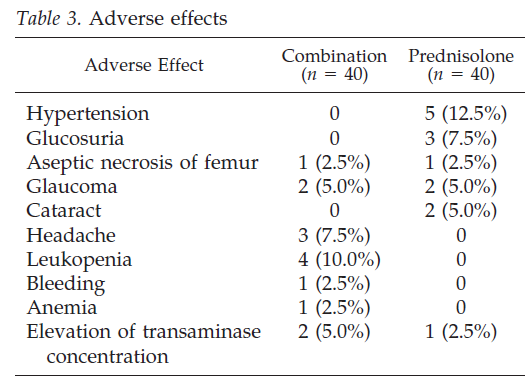  Mean BMI in both groups significantly increased |

**Table S3. IgA Nephropathy Observational Studies**

| **1st , 2nd Author, Year, Country of Origin** | **Title of Publication and citation** | **Study Design and setting** | **Keywords** | **Participants** | **Intervention and Comparator** | **Outcomes** |
| --- | --- | --- | --- | --- | --- | --- |
| Yagi, K., Okada, M. 2003, Japan | Comparison of Antiproteinuric Effects of Two Different Combination Therapies in Children with IgA Nephropathy.  Yagi, K., Okada, M., et al. Clin Exp Nephrol. 2003 7(4):270-274. doi: 10.1007/s10157-003-0255-x. | Retrospective non-randomized trial  **Objective:** To evaluate whether adding mizoribine and an ACEI to steroids attenuated the proteinuric state in children with IgAN.  **Inclusion criteria:**   1. Primary IgAN proven by renal biopsy. 2. 24-h proteinuria of 0.5 g/m^2^ or more 3. Renal histology showing marked diffuse mesangial proliferation and/or 25% or more of glomeruli involved with crescents, sclerosis, and capsular adhesions 4. Normal creatinine clearance (≤70 ml/min per 1.73m^2^)   **Study groups:**  **Group 1:** Prednisolone, mizoribine, dipyridamole, ACEI  **Group 2:** Prednisolone, dipyridamole, cyclophosphamide  **Outcome measures:**  At every 6-month check-up, response to treatment was evaluated on the basis of a reduction of 24-h proteinuria from the baseline (pre-treatment) value, and arbitrarily classified as  **“no response”** (greater than the baseline value)  **“poor response”** (reduction of less than 25% from the baseline value)  **“intermediate response”** (25%–50% reduction from baseline)  **“good response”** (more than 50% reduction from baseline).  The primary endpoint of this study was no response or poor response. | IgA nephropathy,Proteinuria,Mizoribine, Angiotensin-converting enzyme inhibitors, Cyclophosphamide | N= 30 analyzed  **Group 1** (Prednisolone, mizoribine, dipyridamole, ACEI):  n=12/12 analyzed  **Group 2** (Prednisolone, dipyridamole, cyclophosphamide):  n=18/18 analyzed  Mean age:  **Group 1:** 11.3 (SD ± 5.5 yrs)  **Group 2:** 10.6 (SD ± 4.1 yrs)  Sex (M/F): **Group 1:** 7/5 **Group 2:** 11/7 | **Group 1** (n=12):  Prednisolone given orally at a dose of 2mg/kg/day (3x per day-maximum 60 mg) for 4 weeks, followed by alternate day dose of 2 mg/kg/day for 4 weeks, 1.5 mg/kg/day for 4 weeks, 1 mg/kg/day for 4 weeks, and 0.5 mg/kg/day for 4 weeks.  Mizoribine was given orally at a dose of 5 mg/kg/day (2x per day) for 6 months.  *continuously administered beyond 6-months until a decrease in proteinuria (<0.5 g/m^2^ in 24-h).  Dipyridamole was given orally at a dose of 5 mg/kg/day (2-3x per day) for 6 months.  *continuously administered beyond 6-months until a decrease in proteinuria (<1 + by dipstick or <0.2 g/m^2^ in 24-h).  ACEI was given orally at a dose of 0.1-0.3 mg/kg/day (max 5 mg/day) for 6 months.  *continuously administered beyond 6-months until a decrease in proteinuria (<1 + by dipstick or <0.2 g/m^2^ in 24-h).  **Group 2** (n=18):  Prednisolone and dipyridamole given in same manner as G1.  Cyclophosphamide was given orally at a dose of 1 mg/kg/day for 6 months. | **Group 1:**  3 patients reached the primary endpoint at the first 6-month check-up. The remaining patients showed a decrease in proteinuria of 1.2 ± 0.7g/m^2^ at entry  0.64 ± 0.4g/m^2^ at 6 months  0.53 ± 0.5g/m^2^ at 12 months  0.58 ± 0.4g/m^2^ at 18 months.  During the follow-up period, worsening of proteinuria (not associated with infection) was observed at 12 months in one patient and at 24 months in two patients and was judged as the endpoint.  **Group 2:**  all patients revealed intermediate or good response in 24-h proteinuria up to the 18-month check-up:  1.7 ± 1.2g/m^2^ at entry 0.4 ± 0.5g/m^2^ at 6 months 0.2 ± 0.2g/m^2^ at 12 months  0.3 ± 0.3g/m^2^ at 18 months  This indicated a significant (P< 0.05) decrease in proteinuria compared with the value at entry.  One patient showed exacerbation of proteinuria at 24 months, and one showed exacerbation at 30 months after the treatment, which was judged as the endpoint.  **Kaplan-Meier Analysis:**  The proportion of patients that reached the primary endpoint in group 1 was significantly higher than that in group 2 (logrank test, P=0.024)  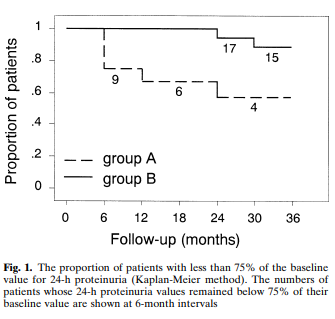  **Comparison of 24-h proteinuria (<0.5 g/m^2^) during follow-up between groups:**  12 months:  **Group 1:** n= 7  **Group 2:** n=17  24 months:  **Group 1:** n=6  **Group 2:** n=15  The proportion of patients persistently having 24-h proteinuria below 0.5g/m^2^ was significantly higher in Group 2 than in Group 1 (P < 0.05; Fisher’s exact test)  **Adverse events:**  1 person in Group 2 developed herpes zoster. |
| Tanaka, H., Suzuki, K., 2004  Japan | Combined Therapy of Enalapril and Losartan Attenuates Histologic Progression in Immunoglobulin A Nephropathy  Tanaka, H., Suzuki, K., et al. Pediatric International. 2004; 46 (5):576-579. | Retrospective Cohort  (Hirosaki University Hospital)  **Objective:** To evaluate whether combined treatment (immunosuppression + ACEI and ARB) was beneficial for histologic chronicity.  **Inclusion:**  9 patients diagnosed with severe IgA nephropathy following renal biopsy between 1991 to 2000. All patients had persistent proteinuria >2 g/day.  **Study groups:**  **Group 1:** Prednisolone, azathioprine, dipyridamole, enalapril, losartan  **Group 2:** Prednisolone, azathioprine, dipyridamole  **Outcome measures:**  Proteinuria (g/day)  Serum creatinine 9mg/dL)  Activity index  Chronicity index  Tubulointerstitial scores | Enalapril, histologic progression, immunoglobulin A nephropathy, losartan, tubulointerstitial changes | N= 9 analyzed  **Group 1** (Prednisolone, azathioprine, dipyridamole, enalapril, losartan):  n=4/4 analyzed  **Group 2** (Prednisolone, azathioprine, dipyridamole):  n=5/5 analyzed  Mean age:  **Group 1:** 12.5 (SD ± 1.3 yrs)  **Group 2:** 12.2 (SD ± 2.5 yrs)  Sex (M/F): **Group 1:** 2/2 **Group 2:** 3/2 | **Group 1** (n=4)  Prednisolone given orally at a dose of 1 mg/kg/day (max 60 mg) for 4 weeks, followed by alternate day dose of 1 mg/kg for 12 weeks. The dose was decreased gradually for the next 8 weeks.  Azathioprine was given at a dose of 2 mg/kg/day (max 100 mg) for 24 weeks.  Dipyridamole was given at a dose of 5 mg/kg/day (max 300 mg) for 24 weeks.  Enalapril was given at a dose of 0.1 mg/kg/day (max 50 mg) for 24 months.  Losartan was given at a dose of 1 mg/kg/day (max 50 mg) for 24 months.  **Group 2** (n=5)  Prednisolone, azathioprine, and dipyridamole were given in same manner as Group 1. | **First biopsy: Group 1**  mean urine protein excretion: 2.6 ± 0.6 g/day  activity index: 5.0 ± 1.0 chronicity index: 5.0 ± 1.0  TI scores: 4.3 ± 1.0  **First biopsy: Group 2**  mean urine protein excretion: 2.2 ± 0.6 g/day  activity index: 4.8 ± 0.8  chronicity index: 4.8 ± 1.3  TI scores: 3.6 ± 0.5  No statistical differences reported between Group 1 and Group 2.  **Second biopsy: Group 1**  Mean urine protein excretion decreased from  2.6 ± 0.6 g/day to 0.5 ± 0.4 g/day  Activity index decreased from 5.0 ± 1.0 to 3.0 ± 1.7  Chronicity score and TI score unchanged or slightly increased.  **Secondary biopsy: Group 2**  Mean urine protein excretion decreased from  2.2 ± 0.6 g/day to 0.7 ± 0.4 g/day  Activity index decreased from 4.8 ± 0.8 to 2.6 ± 0.5  Chronicity score and TI score unchanged or slightly increased.  **Second biopsy: Comparison between Group 1 and Group 2**  Significant suppression in increasing the chronicity index and TI scores obtained at the second renal biopsy in patients was observed.  **Group 1:** 4.3 ± 1.2 and 3.0 ± 0.0  **Group 2:** 6.0 ± 0.7 and 4.4 ± 0.9 (P < 0.05).  **Adverse events:**  None observed. |
| Bulut, I K., Mir, S., 2012  Turkey | Outcome Results in Children with IgA Nephropathy: A Single Center Experience  Bulut, I K., Mir, S., et al.  Int J Nephrol Renovascular Dis. 2012 5:23-28. | Retrospective cohort  Single center  **Inclusion:**  All patients <18 years old with biopsy-proven IgAN hospitalized at the Ege University, Faculty of Medicine, Department of Pediatric Nephrology, from 1991 to 2005 and followed-up for at least 5 years.  **Study groups:**  **Group 1:** Patients with recurrent macroscopic hematuria without proteinuria, and with normal kidney function  **Group 2:** Patients with proteinuria (range 0.5–3 g/1.73 m^2^ /day) with or without microscopic hematuria  **Group 3:** Patients with nephrotic syndrome  **Outcomes:**  Outcomes were graded as follows:   1. Normal (no hypertension, no urinary abnormality and no protein excretion and normal plasma creatinine concentration) 2. Minor urinary abnormalities (proteinuria <1 g/1.73 m^2^ /day with or without microscopic-recurrent macroscopic hematuria) 3. Active renal disease (proteinuria >1 g/1.73 m^2^ /day and/or elevated plasma creatinine level) 4. renal insufficiency (GFR <60 mL/min/1.73 m^2^ ) | immunoglobulin A nephropathy, childhood, prognosis | N=39 analyzed  **Group 1** (Omega-3:Fish oil)  n= 19/19 analyzed  **Group 2** (Fish oil, ACEI)  N=10/10 analyzed  **Group 3** (Fish oil, ACEI, corticosteroid therapy-prednisolone or pulse methylprednisolone)  N=10/10 analyzed  Mean age:  9.5 (SD ± 3.75 yrs)  Sex (M/F): 29/10 | All patients were treated with fish oil after diagnosis.  29/39 (74.4%) were treated with ACEIs  10/39 (25.6%) with corticosteroids.  3/39 (7.7%) patients had corticosteroid plus cytotoxic agents including cyclosporine-A, cyclophosphamide, and azathioprine.  4 patients with nephrotic syndrome, 3 patients with nephritic syndrome, 2 patients with hematuria ± proteinuria and 1 patient with recurrent macroscopic hematuria were given steroid therapy. | Mean follow-up time:  10.4 (SD ± 3.51 years)  At end of follow-up for all patients (n=39)  Outcome A: n=18 (46%)  Outcome B: n=15 (38.5%)  Outcome C: n=3 (7.7%)  Outcome D: n=3 (7.7%)  At end of follow-up for patients receiving steroid treatment (n=10)  Outcome A: n=2 (20%)  Outcome B: n=2 (20%)  Outcome C: n=3 (30%)  Outcome D: n=3 (30%)  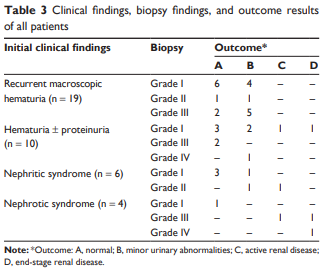 |
| Cambier, A., Rabant, M., 2018, France | Immunosuppressive Treatment in Children  With IgA Nephropathy and the Clinical  Value of Podocytopathic Features.  Cambier A, Rabant M, et al. Kidney Int Rep. 2018 Mar 29; 3(4):916-925. doi: 10.1016/j.ekir.2018.03.013. | Retrospective bicentric study (2 university hospitals in Paris)  1) Necker Enfants Malades Hospital  2)Robert-Debré Hospital  **Objective:** “describe the potential causative effect of steroids on the renal outcome of 82 children with IgAN diagnosed over the past 2 decades”  I**nclusion:**  82 consecutive cases <18 years, newly diagnosed with primary IgAN following renal biopsy; recorded between 1990 and 2015  **Study groups**: immunosuppressant vs. supportive therapy/no treatment  **Exclusion**: systemic disease/associated minimal change disease | Children, histopathology, IgA nephropathyrenal biopsy, steroid | N= 82 analyzed  **Group 1 (immunosuppressive):** n=51  n=15 family history  **Group 2 (RASB or no treatment):**  n= 31 (n=20 with RASB (Group 3), n=11 no treatment)  Mean age: **Group 1**: 9.1 (SD ± 3 yrs) **Group 2:** 11.6 (SD ± 4.8 yrs)  Sex (M/F): **Group 1**:31/20  **Group 2**:21/8 | **Group 1** (n=51)  Pulse (+oral) + Cyclo: n=9  Pulse followed by Oral: n=23  Oral only: n=19  **Pulse steroid therapy**:  I.v. methylprednisolone 500 mg/m2 for 3 consecutive days.  **Oral steroid therapy:** prednisolone 30 mg/m2 daily (Necker Hospital) vs.  60 mg/m2 daily (Robert-Debré Hospital) for the first  month, then 30 mg/m2 1of2 days for the next 2 months (Necker Hospital) vs. 60 mg/m2 1of2 days for the next 2 months (Robert-Debré Hospital),  then 15 mg/m2 1of1 days (Necker Hospital) for the next 18 months or 30 mg/m2 every other day for the next 2 months and then 15 mg/m2 every other day over the next 18 months (Robert-Debré Hospital)  **Cyclophosphamide:** 3 or 6 i.v. pulses of 500 mg/1.73 m2 in persistent  nephrotic syndrome despite pulse steroid and oral steroid therapy.  **Group 2**  Supportive (RASB) + no treatment; n= 31  **Group 3**  RASB only (subset of group 2); n=20 | Compared within group at month 0 (M0) and month 6 (M6)  **Primary**  PCR:  **Group 1**: 1.6 (1-4.3) g/g creatinine at  M0 to 0.3 (0.2-0.7) g/g creatinine  at M6 (p<0.001)  **Group 3**: 0.5 (0.21-1.4) g/g creatinine at  M0 to 0.5 (0.3-0.9) g/g creatinine at M6 (p = 0.4)  eGFR  **Group 1**: 89.9 (61.2–114.5) ml/min per 1.73 m^2^ at  M0 to 110.5 (93.7–120.0) ml/min per  1.73 m^2^ at M6 (p<0.001)  **Group 3**: 111.7 (101.7–120.0) ml/min per 1.73 m^2^ at  M0 to 114.7 (102.6–120.0) ml/min per 1.73 m^2^ at M6 (p = 0.12) |
| Higa, A., Shima, Y., 2015, Japan | Long-term outcome of childhood IgA nephropathy with minimal proteinuria  Higa A, Shima Y, et al. Pediatr Nephrol. 2015 Dec; 30(12):2121-7. doi: 10.1007/s00467-015-3176-5. | Retrospective review of consecutive clinical records of kids diagnosed with IgAN at Kobe University and Wakayama Medical University hospitals between June 1976 and July 2009 and who underwent routine renal biopsies before the start of treatment  **Objective:** “elucidate the characteristics and long-term outcome of patients with a diagnosis  of childhood MP-IgAN”  **Outcomes**: end-point of renal outcome ≥ stage III  chronic kidney disease  Minimal proteinuria at biopsy = <0.5 g/day/1.73m2   | End-stage renal disease, Crescents, Mesangial score, Angiotensin converting enzyme inhibitor, Immunosuppressive therapy | N= 385 (kidney biopsy available)  1) No medication (39, 36.8 %)  2) Antiplatelet and/or anticoagulant (14, 13.2 %)  3) Prednisolone ± antiplatelet and/or anticoagulant (2, 1.9 %)  4)Prednisolone+immunosuppressant ± antiplatelet and/or anticoagulant (2, 1.9 %)  5) Chinese herb (Saireito) (9, 8.5 %)  6) Angiotensin-converting enzyme inhibitor (ACEI) and/or angiotensin II receptor blocker (ARB) (32, 30.2 %)  7) Unknown treatment (8, 7.5 %)  Mean age:  MP: 11.5 (9.0 - 13.7 yrs)  Non MP: 10.3 (8.1 - 12.5 yrs)  Immuno: 10.5 (9.4–12.0 yrs)  No Immuno: 11.4 (8.8–13.7 yrs)  Sex (M/F):  MP: 58/48  Non MP: 57/49  Immuno: 3/1  No Immuno: 51/43 | MP vs Non MP IgAN  Immunosuppression vs no immunosuppression (only in MP)  Meds vs no meds  These comparisons are described but without relevant results or analytic plans | Only outcomes available for meds vs no meds  Of 98 patients, 20 were observed to have spontaneous remission (it is unclear how many patients in each med vs no med group had spontaneous remission)  Graph is helpful for natural history  Renal survival: free from stages III–V CKD |
| Kawasaki, Y., Maeda, R., 2018, Japan | Comparison of long-term follow-up outcomes between multiple drugs combination therapy and tonsillectomy pulse therapy for pediatric IgA nephropathy  Kawasaki Y, Maeda R, et al. Clin Exp Nephrol. 2018 Aug;22(4):917-923. doi: 10.1007/s10157-017-1515-5. | Retrospective cohort study  **Objective:** Multiple drug combo vs tonsillectomy  **Inclusion:**  1) IgAN: presence of IgA as the sole/predominant immunoglobulin in the mesangium; absence of systemic disease (Henoch-Schoenlein purpura nephritis or systemic lupus erythematosus)  2) Under 15 years of age as of the start of therapy and followed up for more than 3 years  3) no previous treatment with corticosteroids/  immunosuppressives  4) Available renal biopsy tissue (minimum of 10 glomeruli).  **Outcome:**  **Stage 1 (Normal):** the results of the physical examination were normal, and the patient had normal urine and normal renal function  **Stage 2 (Hematuria only):** the results of the physical examination were normal, but urinalysis revealed microscopic hematuria or proteinuria of less than 5 mg/m2/h  **Stage 3**  **(minor urinary abnormalities):** the results of the physical  examination were normal, but urinalysis revealed microscopic hematuria or proteinuria of 5–20 mg/m2/h  **Stage**  **4 (persistent nephropathy):** the patient had 20 mg/m2/h or greater proteinuria, and e-GFR was 60 ml/min/1.73 m2 or  greater  **Stage 5 (renal insufficiency):** the patient had an e-GFR value of less than 60 ml/min/1.73 m2 | IgA nephropathy,Tonsillectomy plus methylprednisolone pulse therapy, Long-term follow-up, Outcome, Pediatrics | N=61  PWDM: n=44  TPT: n=17  **Severe** vs mild IgAN:  1) heavy proteinuria (an early morning urinary protein to creatinine ratio > 1.0  2) > 80% of glomeruli showing  moderate or severe mesangial cell proliferation, crescent  formation, adhesion, or sclerosis or cases with > 30% of glomeruli showing crescent formation  Mean age:  PWDM: 12 (SD 9.8–14.3 years)  TPT: 12 (SD 11.0–13.0 yrs)  Sex (M/F):  PWDM: 28/26  TPT: 10/7 | **PWDM:**  Prednisolone was given orally at a dose of 2 mg/kg body wt. per day in three divided doses for a total dose of not more than 60 mg/day for 2 weeks, followed by 1.5 mg/kg per day for 2 weeks, 1.0 mg/kg per day for 4 weeks, 0.5 mg/kg per day for 4 weeks, 1.0 mg/kg every 2 days for 9 months, and 0.5 mg/kg every 2 days for 12 months.  MZB was given orally at a dose of 5 mg/kg body wt. per day in two divided doses for 24 months.  Warfarin was given orally at a dose of 1–2 mg/day.  Dilazep dihydrochloride was given orally at a dose of 5 mg/kg per day in three divided doses for 24 months.  An angiotensin-converting enzyme inhibitor (ACEI) or Angiotensin II Receptor Blocker (ARB) were administered to patients with persistent nephropathy and  exacerbation of proteinuria  **TPT:**  Tonsillectomy plus prednisolone, warfarin, and dipyridamole including methylprednisolone (MP) pulse therapy (three courses)  High-dose MP at 20–25 mg/kg/day for 3 days per week.  Prednisolone was given orally at a dose of 2 mg/kg body wt. per day in three divided doses for a total dose of not more than 60 mg/day for 2 weeks, followed by 1.5 mg/kg per day for 2 weeks, 1.0 mg/kg per day for 4 weeks, 0.5 mg/kg per day for 4 weeks, 1.0 mg/kg every 2 days for 9 months, and 0.5 mg/kg every 2 days for 12 months | Unclear timeline for second biopsy  **Primary**  eGFR  **PWDM:** 97 (85–104) ml/min/1.73 m^2^ at first biopsy to 103 (90–110) ml/min/1.73 m^2^ at second biopsy to 104 (91–110) ml/min/1.73 m^2^ at most recent follow up  **TPT:** 92 (86–99) ml/min/1.73 m^2^ at first biopsy to 110 (104–112) ml/min/1.73 m^2^ at second biopsy to 104 (100–112) ml/min/1.73 m^2^ at most recent follow up  Proteinuria  **PWDM:** 1.4 (1.0–2.0) g/day at first biopsy to 0.11 (0–0.24) g/day at second biopsy (p<0.01) to 0 (0-0) at most recent follow up  **TPT:** 1.7 (1.2–2.2) g/day at first biopsy to 0 (0–0.1) g/day at second biopsy (p<0.01) to 0 (0-0) at most recent follow up  Note: significance is reported with respect to changes within groups. There was no significant difference in between group changes.  **Secondary**  10 patients in the PWDM group and 3 patients in the TPT group had mild growth retardation.  6 patients in the PWDM group and 2 patients in the TPT group had glaucoma.  **Outcomes:**  First biopsy:  **PWDM** 43 patients (98%) in stage 4 and 1 patient (2%) in stage 5.  **TPT** 15 patients (88%) in stage 4 and 2 patients (12%) in stage 5.  Second biopsy:  **PWDM** 30 patients (68%) in stage 1, 4 patients (9%) in stage 2, 5 patients (11%) in stage 3, 4 patients (9%) in stage 4 and 1 patient (2%) in stage 5.  **TPT** 15 patients (88%) in stage 1 and 2 patients (12%) in stage 3.  Differences are non-significant. |
| Wu, H., Fang, X., 2020, China | Long‑term renal survival and undetected risk factors of IgA nephropathy in Chinese children—a retrospective 1243 cases analysis from single centre experience  Wu H, Fang X, et al. J Nephrol. 2020 Dec; 33(6):1263-1273. doi: 10.1007/s40620-020-00767-4. | Retrospective cohort study  **Inclusion:**  Children with IgAN who underwent renal biopsy between  January 2000 and December 2017 in Jinling hospital    **Exclusion:**  1) insufficient glomeruli on biopsy  2) combined with Hep B or Alport’s  3) initial eGFR ≤ 15 ml/min/1.73 m2  4) Henoch–Schonlein purpura, liver cirrhosis and patients with other secondary IgAN  **Objective:**  1) “Confirm the long-term renal survival and some undetected risk factors for renal outcome in Chinese children.”  2) “Assess the benefits of immunosuppressive therapy overall and in subgroups of interest defined by the initial eGFR and proteinuria.”  **End-point:**  The primary endpoint of this retrospective study was a composite event of either ≥50% reduction eGFR or ESRD or death. | IgA nephropathy, Chinese children, Renal survival, Long-term observation,Undetected risk factors | N=1243  Participants were Chinese Han (i.e. major ethnic group)  RASB: n=868  RASB+IS: n=788  Mean age: 13.70 (± 3.70 yrs)  Sex (M/F): 841/402 | RASB  RASB+IS  1)Corticosteroids  n=557  2) CS + other (i.e. mycophenolate mofetil, leflunomide, mizoribine, and tacrolimus)  n=231 | Renal outcomes were better in the RASB+IS group compared with RASB group  RASB+IS benefited children with an initial eGFR > 50 ml/min/1.73 m^2^  GFR ≤ 50 ml/min/1.73 m^2^, there was no significant difference in renal outcomes between RASB and RASB+IS  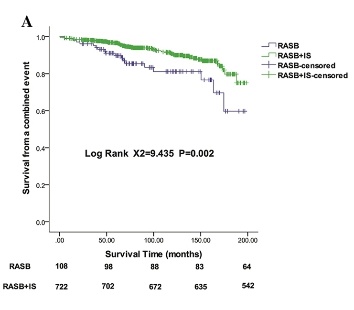  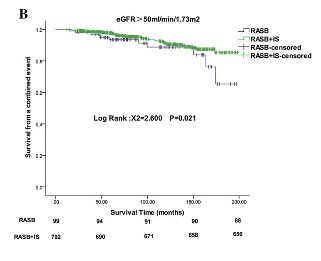  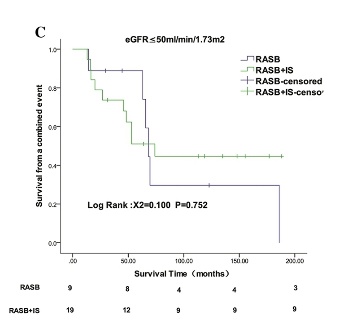  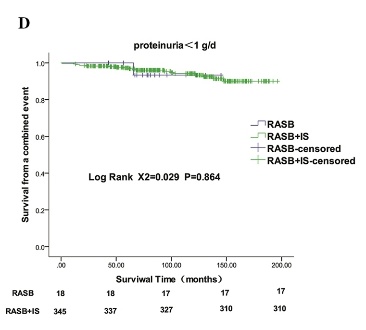  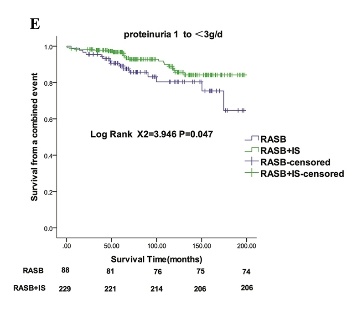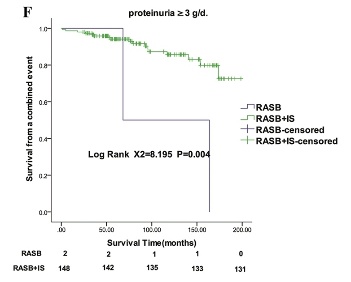 |
| Waldo, F. Bryson; Wyatt, Robert J.  1993. USA | Treatment of IgA nephropathy in children:  Efficacy of alternate-day oral prednisone.  Pediatric Nephrology 1993;7(5):529-532 | Observational study | IgA nephropathy, treatment, prednisone | N=28 patients  n=13 for treated and 15 for untreated  **Inclusion**  **Treated group**  1) Diagnosed by biopsy between 1979-1989.  2) Proteinuria >1 g/m2 per day or biopsy findings (presence of glomerular sclerosis or tubular atrophy and interstitial fibrosis).  (4 pts had both the clinical and pathological criteria,  6 met pathological alone and 3 met clinical alone).  Mean age=11 yr.  Sex M/F: 10/3,  Follow up = 67 mo.  **Untreated group (control)**  1) Diagnosed between 1979 and 1989 and did not receive steroids  2) sCr< 145 µmol/L  3) Biopsy grade 3/3 (based on Southwest Pediatric Nephrology Study scoring system).  Mean age 12 yrs  Sex M/F: 10/5  Follow up= 96 mo.  **Exclusion**  HSP | **Treatment: Alternate day steroid**  **Treatment protocol**  Initial Tx of 60 mg/m2 prednisone every other morning x 3 mo.  Next 9 mo., dose decreased from 60 to 30 mg/m2 every other morning. (One patient (no. 7) received only 1 month of prednisone at  60 mg/m2 because of complaints of weight gain).  Between 12 and 24 months, dose decreased from 30 to 15 mg/m 2 every other morning.  Between 2-4 yr., dose gradually reduced or discontinued, based on current biopsy results and clinical evidence of disease activity. Rate of dose reduction varied considerably and was based on the presence of hematuria and persistent activity on repeat biopsy. At 2 yr patients with persistent microscopic hematuria (usually <2+) or persistent glomerular hypercellularity (activity index of >4) were continued on prednisone at 15 mg/m2 every other morning for another 12-24 mo.  Mean follow-up =5.2 yr. | **Urinalysis normal**  Treated=12/13 patients untreated =1/15), p<0.001.  **Proteinuria >1g/1.73m2/d**  Treated =1/13 patients, Untreated = 4/15 (p non-significant)  **ESRD or GFR<70ml/min 1.73m2**  Treated=1/13 patients Untreated= 7/15; p=0.03.  **ESRD: (subgroup of outcome 3)**  Treated=0/13 patients, Untreated= 5/15; p=0.04.  Important finding: There was GFR preservation in the treatment group.  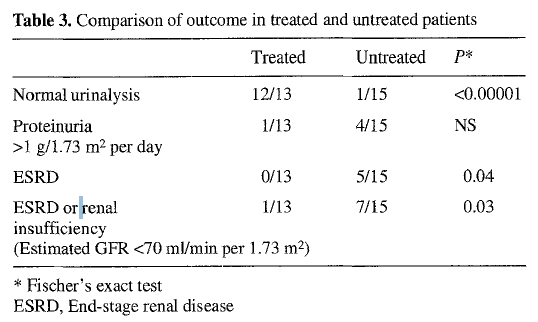  **Complications**  Weight gain: Most patients had mild weight gain in first 6-12 mo which was lost with dose reduction.  HTN: No patient developed HTN requiring treatment during therapy.  Other: No cataracts, no bone disease.  Growth: No growth retardation (z-scores reported) (treatment group in this study was compared with the untreated group from another cohort) |

**Table S4. Major Differential Diagnosis of Dominant/Codominant Glomerular IgA Deposits**

| IgA Nephropathy^1^   - Primary - Secondary (most common causes of secondary IgA nephropathy include hepatobiliary diseases [cirrhosis, hepatitis], gastrointestinal diseases [especially IBD], rheumatologic diseases, HIV infection, sarcoidosis, psoriasis) - Familial   IgA Vasculitis (Henoch-Schonlein Purpura) Nephritis^2^  Lupus Nephritis^3^   - C1q Nephropathy - HIV-Associated Lupus-Like GN   IgA-Dominant Postinfectious (Post-Staphylococcal) GN^4^  Mixed Lesions^5^   - ANCA-Associated GN & Mesangial IgA - Minimal Change Disease & Mesangial IgA - Membranous Nephropathy & Mesangial IgA   Incidental (Latent) Mesangial IgA Deposits (without urinary abnormalities)^6^ |
| --- |
| Differential diagnoses 1, 2, and 6 are distinguished on clinical grounds, whereas 3-5 show pathologic features that distinguish these from IgA nephropathy: lupus nephritis, C1q nephropathy, and HIV-associated lupus-like GN each show glomerular C1q deposits that are typically absent in IgAN; IgA-dominant postinfectious GN shows subepithelial hump-like deposits by EM; ANCA-associated GN shows glomerular crescents and/or necrotizing lesions with little or no hypercellularity in glomeruli without crescents/necrosis; minimal change disease shows diffuse podocyte foot process effacement which is rare in IgAN, especially in cases with mild histologic lesions; membranous nephropathy shows glomerular capillary wall IgG staining and numerous subepithelial deposits by EM. |

**Table S5. The Oxford (MEST-C) Classification of IgA Nephropathy**

| **Parameter** | **Histological Description** | **Score** |
| --- | --- | --- |
| **M** | Mesangial hypercellularity  (>4 mesangial cells in any mesangial area of glomerulus) | M0: <50% glomeruli showing mesangial hypercellularity  M1: >50% glomeruli showing mesangial hypercellularity |
| **E** | Endocapillary hypercellularity | E0 absent  E1 present in >1 glomerulus |
| **S** | Segmental glomerulosclerosis/  adhesions | S0 absent  S1 present in >1 glomerulus |
| **T** | Tubular atrophy/ interstitial fibrosis | T0-0-25% of cortical area  T1-26-50% of cortical area  T2->50% of cortical area |
| **C** | Crescents (cellular or fibrocellular) | C0: no crescents  C1: crescents in <25% of glomeruli  C2: crescents in >25% of glomeruli |

**Table S6. Studies Validating Oxford (MEST-C) Classification in Pediatric Populations**

| **Study** | **Patients** | **Endpoint** | **Biopsy findings** | **Associations of biopsy finding with composite endpoint** |
| --- | --- | --- | --- | --- |
| Wu *et al. 2020*  China | 1243 patients, all <18 years old, median follow-up 7.2 years | ≥50% reduction eGFR or ESKD or death  14% of patients reached the composite endpoint | 29% M1  35% E1  37% S1  23% T1  4.3% T2  44% C1  4.6% C2 | Univariate regression analysis: S1, T1/2, C1/2 significantly associated with composite endpoint  Multivariable regression analysis: S1 and T1/2 significantly associated with composite endpoint; C1/2 significantly associated with composite endpoint only in patients not receiving immunosuppression |
| Shima *et al. 2012*  Japan | 161 patients, all <20 years old, median follow-up 52 months from biopsy, 7.4 years from disease onset | eGFR< 60ml/min/1.73m²  7 patients reached endpoint |  | Univariate and multivariable regression analysis: M1, T1/2, crescents in >30% of glomeruli significantly associated with endpoint |
| Coppo *et al. 2019*  European cohort  (13 countries) | 174 patients <18 years old, median follow-up 4.7 years | ESKD or >50% decline in eGFR  6.3% of patients reached the composite endpoint | 22% M1  14% E1  43% S1  6.3% T1/T2  15% C1/C2 | None of the MEST-C scores were significantly associated with the composite endpoint or slope of eGFR by univariate analysis |
| Halling *et al. 2012*  Sweden | 99 children and adolescents (mean age 12 + 3.6 years), all followed >5 years (mean 13 years) | ESKD or >50% decline in eGFR  18% of patients reached the composite endpoint | 31% M1  10% E1  23% S1  12% T1  3% T2  18% C1/C2 | Univariate and multivariable regression analysis: M1, E1, T1/2, C1/2 significantly associated with composite endpoint |
| Le *et al. 2012*  China (7 centres) | 218 patients, all <18 years old, median follow-up 56 months | ESKD or >50% decline in eGFR  12.4% of patients reached the composite endpoint | 45% M1  23% E1  62% S1  6% T1  1% T2  44% C1/C2 | Univariate regression analysis: S1, T1/2 significantly associated with composite endpoint  Multivariable regression analysis: only T1/2 significantly associated with composite endpoint |
| Abbreviations: eGFR: Estimated glomerular filtration rate; ESKD: End stage kidney disease; M1: >50% glomeruli showing mesangial hypercellularity; E1: Endocapillary hypercellularity present in >1 glomerulus; S1: Segmental glomerulosclerosis/adhesions present in >1 glomerulus; T1: Tubular atrophy/interstitial fibrosis in 26-50% of cortical area; T2: Tubular atrophy/interstitial fibrosis in >50% of cortical area; C1: Crescents (cellular or fibrocellular) in <25% of glomeruli; C2: Crescents (cellular or fibrocellular) in >25% of glomeruli. | | | | |

**Table S7. IgA Vasculitis Nephritis Randomized Controlled Trials**

| 1st , 2nd Author, Year, Country of Origin | Title of Publication and citation | Study Design and setting | Keywords | Participants | Intervention and Comparator | Outcomes |
| --- | --- | --- | --- | --- | --- | --- |
| Du Y, Zhang Z et al., 2016, China  Abstract only | Comparison of Leflunomide and  Mycophenolate mofetil in children with Henoch-Schonlein  nephritis [abstract].  Pediatric Nephrology 2016;31(10):1817  doi: 10.1007/s00467-016-3467-5 | Randomized controlled trial (open label).  Inclusion  HSPN children failed steroid treatment with nephrotic-range proteinuria and estimated glomerular filtration rate (eGFR)>60ml/min.1.73m^2^ | N/A | N= 18 children  Sex (M/F): N/A  Mean age: N/A  Ethnicity: N/A | Group 1 (n= 8):  Leflunomide  Group 2 (n= 10)  Leflunomide and mycophenolate mofetil  Both groups also receive fosinopril and tapered prednisone | 24-hour urine protein  1-month  Group 1: 0.48×0.56 g/d  Group 2: 1.35×1.12 g/d  3-month  Group 1: 0.22×0.28 g/d  Group 2: 0.58×0.58 g/d  6-month  Group 1: 0.057×0.037 g/d  Group 2: 0.21×0.31 g/d  9-month  Group 1: 0.031×0.023g/d  Group 2: 0.08×0.07 g/d  Between and within groups:  24-hour urine protein and serum albumin (not shown) were significantly different at 1-month between groups (p<0.05).  Within groups, both had significantly reduced 24-hour urine protein over time (p<0.05), but not different between groups (p>0.05).  Mean proteinuria level at 9-months:  Group 1: 0.03×0.02 g/d  Group 2: 0.08×0.07 g/d    Adverse events  No serious side effects detected in group 1. One participant in group 2 had elevated glutamate pyruvate transaminase, but was resolved during trial. |
| Dudley J, Smith G, 2013, United Kingdom | Randomised, double-blind, placebo-controlled trial to determine whether steroids reduce the incidence and severity of nephropathy in Henoch-Schonlein Purpura (HSP).  Archives of Disease in Childhood 2013;98(10):756-63.  doi: 10.1136/archdischild-2013-303642 | Double-blind randomized-controlled trial.  Inclusion:  diagnosis of HSP (HSP), based on the American College of Rheumatology criteria  Exclusion:   - were already receiving steroid/immunosuppressive therapy; - were receiving ACE inhibitors; - had pre-existing renal disease (excluding urinary tract infections); - had pre-existing hypertension; - had evidence of immunodeficiency/systemic infection; - had contraindications or relative contraindications for steroid therapy (epilepsy, diabetes mellitus, glaucoma or peptic ulceration); - had had the characteristic purpuric rash for more than 7 days. | General Paediatrics; Nephrology | N = 352 entered and 247 with endpoint data  Group 1: 181 entered and 123 analysed at endpoint  Group 2: 171 entered and 124 analysed at endpoint  Median age, range (years): Group 1: (6.34, 1 to 15.7); Group 2: (6.12, 0.5 to 13.9)  Sex (M/F):  Group 1: (93/88)  Group 2: (100/72) | Group 1: prednisolone at a dose of 2 mg/kg/day (max 80 mg) for 7 days, followed by 1 mg/kg/day for 7 days (max 40 mg)  Group 2: placebo for 14 days | Urine protein : creatinine ratio >20 mg/mmol:  Group 1: 18/123 (18%)  Group 2: 13/124 (10%)  OR (95%CI) =1.46 (0.68,3.14)  P=0.03  Need for additional treatment:  Group 1: 5/164 (3%; 95% CI: 1,7)  Group 2: 9/159 (6% 95% CI: 3,11)  HR (95% CI) = 0.53 (0.18, 1.58)  Trial Induced toxicity:  Group 1: 2/158 (1%)  Group 2: 1/153 (1%)  Adverse Events:  Group 1: 27/180 (15%)  Group 2: 23/170 (14%) |
| Fuentes Y, Valverde S, 2010, Mexico  Abstract only | Comparison of azathioprine vs mofetil mycophenolate for Henoch-Schonlein Nephritis treatment [abstract]  Pediatric Nephrology 2010;25(9):1802  doi: 10.1007/s00467-010-1577-z  Efficacy of mycophenolate mofetil and prednisone compared to azathioprine and prednisone treatment in children with Henoch-Shonlein purpura nephritis [abstract]  Pediatric Nephrology 2016;31(10):1753-4  doi: 10.1007/s00467-016-3466-6 | Randomised controlled trial  Inclusion:  Children with HSPN and renal biopsy two weeks prior to treatment  Exclusion:  Nephropathy class of IV or V | N/A | N= 26  Group 1; 13  Group 2: 13  Mean age ± SD (years):  Group 1 (6.8 ± 2.1)  Group2: (6.9± 2.2)  Sex (M/F):  Group 1: 10/3  Group 2: 7/6 | Group 1:  mofetil mycophenolate 1000 mg/m2/day for 12 months  Group 2:  Azathioprine 2mg/kg/d for 12 months | 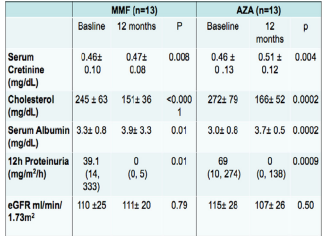 |
| Geng Hy, Chen CY, 2021, China | Efficacy and safety of mycophenolate mofetil versus cyclophosphamide in the treatment of Henoch-Schonlein purpura nephritis with nephrotic-range proteinuria in children: a prospective randomized controlled trial  Zhongguo Dangdai Erke Zazhi 2021;23(4):338-42  doi: 10.7499/j.issn.1008-8830.2012145 | Randomised controlled trial  Inclusion criteria:  In line with the HSPN diagnostic criteria formulated by the Nephrology Group of the Pediatric Branch of the Chinese Medical Association in 2009  Exclusion criteria:   - WBC count <3×109/L. - Haemoglobin levels< 90g/L. - Liver function impairment. - Patients with active infections. - Kidney biopsy results showing moderate or higher levels of renal tubular atrophy and/or interstitial fibrosis - Have a previous history of using immunosuppressive medications other than prednisone | Henoch-Schönlein purpura nephritis; Pharmacotherapy; Immunosuppressive agent; Mycophenolate mofetil; Child | N = 68  Group 1: 33  Group 2: 35  Median age (IQR) years:  Group 1: 8.3 years (IQR 7.0, 11.6  Group 2: 8.1 years (IQR 6.8, 11.2)  Sex (M/F):  Group 1: 17/16  Group 2: 21/14 | Group 1: Mycophenolate mofetil  15-30 mg/kg dose, twice a day orally (Maximum dose of 2 g/d) for 3 months.  If treatment effective MMF continued to 6 months & then dose reduced.  If urine protein levels did not decrease by 50% by 3 months, MMF was replaced with a different immunosuppressive drug.  Group 2: IV cyclophosphamide  8-12 mg/kg/day for 2 days every two weeks for 6 treatments in 3 months.  If treatment effective, regimen was changed to 2 days every 1 month till maximum dose of 168 mg/kg reached    Both groups: Prednisone orally: 1.5-2mg/kg/d for 4-6 weeks  Methylprednisolone x 3 pulses IV given if no reduction in urine protein at 4 weeks of oral prednisone followed by further course of oral prednisone | Remission: 24hour urine protein was negative and urine protein<150mg:  3month:  Group 1: 17 (52%)  Group 2: 15 (43%)  P=0.475  6month  Group 1: 25 (76%)  Group 2: 26 ( 74%)  P=0.889  12month  Group 1: 27 (82%)  Group 2: 27 (77%)  P=0.634  Partial Remission (24hour urine protein negative but 24hr protein levels decreased by >50%) + Complete Remission:  3month:  Group 1: 28 (85%)  Group 2: 34 (97%)  P=0.174  6month  Group 1: 28 (85%)  Group 2: 34 (97%)  P=0.174  12month  Group 1: 28 (85%)  Group 2: 34 (97%)  P=0.174 |
| He YY, Pan W, 2002, China  Abstract only | Preventive effect of heparin on the development of nephropathy in Henoch-Schoenlein purpura-a randomized controlled clinical trial.  Chinese Journal of Pediatrics 2002;40(2):99-102  doi: 10.3760/j.issn:0578-1310.2002.02.011 | Randomised controlled trial  Inclusion:  Children at onset or relapse of HSP but presumed to be without kidney disease | Purpura; allergic heparin; Nephritis; randomised controlled trial | N = 228  Group 1: 119  Group 2: 109  Sex: N/A  Age: N/A | Group 1: Heparin: sodium heparin 120 to 150 IU/kg/d IV for 5 days or calcium heparin 10 IU/kg/ subcutaneously twice daily for 7 days given at onset or relapse of HSP  Group 2: Placebo injection of 10% glucose only  Both Groups: Oral Vitamin C and rutin (not defined) | Nephritis:  Group 1: 9 (7.6%)  Group 2: 30 (27.5%)  P<0.01  Time to nephritis:  Group 1 (days): 82 ± 64  Group 2 (days): 34 ± 32  P<0.01  Hematuria:  Group 1: 2 cases  Group 2: 12 cases  Hematuria + proteinuria:  Group 1: 6 cases  Group 2: 15 cases  Hematuria + nephropathy:  Group 1: 1 case  Group 2: 3 cases |
| Huber, A.M., King, J., 2004,  Canada | A randomized, placebo-controlled  trial of prednisone in early Henoch Schonlein purpura.  BMC Medicine 2004;2:7.  doi: 10.1186/1741-7015-2-7 | Prospective randomized controlled trial.  Double-blind  Placebo-controlled study  Objective: To determine if the early use of prednisone therapy in children with HSP could prevent the development of renal involvement at one year, or the development of gastrointestinal complications in the acute period.  Inclusion criteria:   1. Diagnosis of HSP between September 1, 1996 and January 31, 2000 2. Aged 2-15 years within 7 days of disease onset   Exclusion criteria:   1. Children with another reason for purpura 2. Known underlying systemic vasculitis 3. Steroids in previous month 4. Underlying kidney, gastrointestinal or immunodeficiency illness 5. Active infection 6. Life threatening complication of HSP   Study groups:  Group 1: Treatment  Group 2: Placebo  Outcomes:   1. Renal involvement at 1-year (described as hematuria on microscopy >5 red blood cells per high powered field, red cell casts, proteinuria >0.3 g/l, hypertension >90^th^ percentile for age and gender) 2. Acute gastrointestinal complications (defined as GI bleeding requiring transfusion or fluid resuscitation, intussusception, perforation or need for operative intervention) 3. Duration of symptoms 4. Admission, recurrence, and withdrawals   HSP defined as: palpable purpura and 1 or more of arthritis, kidney disease or gut involvement  Kidney disease defined as: haematuria 5 or more RBC/HPF or RBC casts, proteinuria 0.3g/L or more, hypertension 90th percentile for age/sex or above. | n/a | N= 40 analyzed  Subjects were evaluated at baseline, two weeks, one month, and one year  Urine collections at 2, 3, and 6 months  Group 1 (Treatment):  n=21/21 analyzed  Group 2 (Placebo):  n=19/19 analyzed  Note: 3 withdrawals, leaving 16 patients completing protocol  Mean age:  Group 1: 5 (2-11)  Group 2: 6.1 (3-15)  Sex (M/F): Group 1: 13/8 Group 2: 12/7  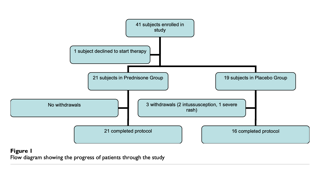  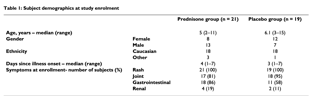 | Group 1 (n=21):  Oral prednisone 2 mg/kg for seven days  Followed by a weaning dose of prednisone for the next seven days (75% on days 8 and 9, 50% on days 10 and 11, 25% on days 12, 13, and 14, then discontinue)  Group 2 (n=19):  Received an identical number of pills, and followed the same schedule | Renal Involvement  One-Year  Group 1: 3 children (14.2%)  Group 2: 2 children (10.5%)  Note: all these children had persistent hematuria and/or proteinuria  P=1.0  Two Weeks, Three Months, Two Months, One Month  No differences in renal involvement seen between the 2 groups   - 2 weeks: 3 in group 1; 1 in group 2 - One month: 3 in group 1; 3 in group 2 - Two months: 4 in group 1; 3 in group 2 - Three months: 3 in group 1; 2 in group 2 - Six months: 2 in group 1; 1 in group 2   Follow-Up  Presence of renal involvement not different between the 2 groups  Group 1: 9 children  Group 2: 5 children  Gastrointestinal Complications  Group 1: 2 children (9.5%)  Both severe abdominal pain requiring admission but no other intervention  Group 2: 3 children (15.8%)  Two intussusceptions, both of which required operative intervention, and one with severe abdominal pain required admission but no other intervention  P=0.7  Duration of Symptoms  Summarized by table 2  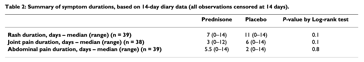  Admission  Group 1: 2 children (9.5%)   - Both severe abdominal pain   Group 2: 3 children (15.8%)   - Two intussusceptions and one with severe abdominal pain   Recurrences  Group 1: 2 children (9.5%)   - Primarily rash   Group 2: 4 children  Withdrawals  Group 1: 0 children  Group 2: 3 children (15.8%)   - The 2 children with intussusceptions and one with severe and worsening rash   Adverse events  n/a |
| Jauhola O, Ronkainen J, 2011, Finlan | Cyclosporine A vs. methylprednisolone for Henoch-Schonlein nephritis: a randomized trial.  Pediatric Nephrology 2011;26(12):2159-66  doi: 10.1007/s00467-011-1919-5 | Controlled trial with some randomisation.  Kidney biopsy diagnosis of crescentic HSP-associated kidney disease of ISKDC grade III or IV or grade II with nephrotic syndrome  Exclusion criteria: medication known to interact with Cyclosporine A | Vasculitis; Glomerulonephritis;  Immunosuppressive treatment; Proteinuria;  Hematuria;  Biopsy; Outcome | N = 24 total (15 randomised and 9 not randomised (NR))  Group 1: 7 + 4 NR  Group 2: 8 + 5 NR  Randomised group characteristics:  Mean age, range (years): Group 1: 9.2 (2 to 18); Group 2: 7.9 (4.0 to 14.8)  Sex (M/F):  Group 1: (5/2)  Group 2: (6/2) | Group 1: Cyclosporine A 5 mg/kg/d for 12 months, titrated according to whole blood CyA concentration  Group 2: Methylprednisolone 30 mg/kg IV x 3 in 1 week Prednisone 30 mg/m^2^/d on intermediate days and for 1 month after, then tapered over 3 months  Both Groups:  ACEI enalapril at a dose of 0.1–0.5 mg/kg/day | Remission of nephrotic range proteinuria:  Group 1: 100% by 3 month  Group 2: ~50% by 3 month  P=0.016  Group 1: 100% at 24 months  Group 2: ~ 75% at 24 months  P=0.223  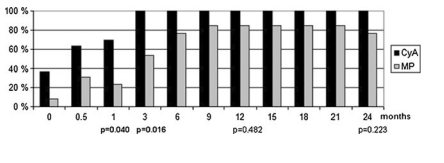Nephrotic range: 24-h protein >  40 mg/m2 /h or by estimating the 24-h protein from the urine protein/creatinine ratio  Hematuria:  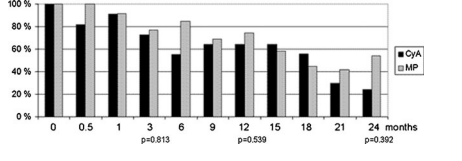 |
| Liu N, Ma ZZ, 2019, China | Clinical effect of double filtration plasmapheresis combined with glucocorticoid  and immunosuppressant in  treatment of children with severe Henoch-Schonlein purpura nephritis  Zhongguo  Dangdai Erke Zazhi 2019;21(10):955-9.  doi: 10.7499/j.issn.1008-8830.2019.10.001 | Randomized controlled trial  Inclusion Criteria: Children with HSPN aged < 18 yrs; Nephrotic syndrome with > 2 gm/d proteinuria: biopsy grade IIIB HSP or more severe. Diagnosis of HSP based on rash +/- gastrointestinal symptoms &/or joint symptoms.  Exclusion Criteria: Patients with coagulopathy; patients who had been previously treated with the treatment regimen used in this study; Severe infections. | Henoch-Schönlein purpura nephritis; Double filtration plasmapheresis; Methylprednisolone; Cyclophosphamide; Child | N = 60  Group 1: 30  Group 2: 30  Mean age:  Group 1: 10.4±1.9 yrs; Group 2: 9.8±1.6 yrs  Sex (M/F):  Group 1: (18/12)  Group 2: (21/9) | Group 1:  Double filtration plasmapheresis (DFPP) 2 hrs/day, every 3 days x 4-5 treatments. Started 3-5 days after pulse methylprednisolone/cyclophosphamide though exact timing unclear  Methylprednisolone (MP) 15-20 mg/kg IV x 3 days; 3 courses at 5-7 d intervals then prednisone po 1 mg/kg/d. Unclear how long this was continued  Cyclophosphamide 8-10 mg/kg IV daily x 2d; repeated for total of 6-7 courses. Max dose CPA 120-130 mg/kg  Group 2:  Methylprednisolone (MP) 15-20 mg/kg IV x 3 days; 3 courses at 5-7 d intervals then prednisone po 1 mg/kg/d. Unclear how long this was continued  Cyclophosphamide 8-10 mg/kg IV daily x 2d; repeated for total of 6-7 courses. Max dose CPA 120-130 mg/kg | Complete remission: haematuria disappears, 24 hr urine protein <150 mg, urine series micro-proteins normal, and renal function normal:  Group 1: 10 (33%)  Group 2: 7 (23%)  Partial remission: hematuria is significantly improved; 24 hr urine protein levels and urine series trace protein were better:  Group 1: 12 (47%)  Group 2: 12 (40%)  Ineffective:  Group 1: 11 (37%)  Group 2: 6 (20%)  Between Groups:  P = 0.174 |
| Mollica F, Li Volti S, 1992, Italy | Effectiveness of early prednisone treatment in preventing the development of nephropathy in anaphylactoid purpura  European Journal of Pediatrics 1992;151(2):140-4.  doi: 10.1007/BF01958961 | Randomised Controlled trial  Unselected children with anaphylactoid purpura  Exclusion criteria: 34 children with haematuria, proteinuria or both on initial presentation | Anaphylactoid purpura; Nephropathy;  Prednisone; Schonlein-Henoch purpura | N = 168 included with follow up  Group 1: 84  Group 2: 84  Age: not reported Sex (M/F): not reported | Group 1: Prednisolone: 1 mg/ kg orally for 2 weeks  Group 2: No treatment | Number of patients who develop AP nephropathy with 2 or more of: proteinuria ≥ 4 mg/m2/hr haematuria ≥> 10 RBC/HPF BP ≥ 2 SD above normal for age BUN ≥ 54 mg/dL Cr ≥ 0.8 mg/dL/m2  Group 1: 0/84 (0%)  Group 2: 10/84 (11.9%)  P<0.001 |
| Peratoner L, Longo F, 1990, Italy | Prophylaxis and therapy of  glomerulonephritis in the course of anaphylactoid purpura.  The results of a  polycentric clinical trial.  Acta Paediatrica Scandinavica 1990;79(10):976-7.  doi: 10.1111/j.1651-2227.1990.tb11365.x | Randomised Controlled trial  Inclusion criteria Children aged 2 to 14 years with anaphylactoid purpura | N/A | Two populations received the treatments: N = 101  AP Only: 82 total  Group 1: 47  Group 2: 35  AP + GN: 19 total  Group 1: 13  Group 2: 6  Age: not reported  Sex (M/F): not reported | Group 1: Dipyridamole: 4 mg/kg/d orally in 3 doses Cyproheptadine: 0.5 mg/kg/d orally in 3 doses Salicylates: 10 mg/kg/d orally in one dose for 8 weeks.  Group 2: symptomatic treatment if necessary (anti-pyretics, antibiotics) | GN during 1 year of follow-up, assessed using the following scoring system:  AP Only:  Group 1: 6 (12.8%)  Group 2: 3 (8.8%)    AP + GN:  Severity “were similar” for both treatment groups  GN scored using: haematuria > 5 RBC/mm3 (score 0 to 2) cylinduria (0-1) hypertension (0-1) reduced CrCl (0-2) |
| Ronkainen J, Koskimies O, 2006, Finalnd | Early prednisone therapy in Henoch-Schonlein purpura: a randomized double blind,  placebo-controlled  trial  Journal of Pediatrics 2006;149(2):241-7  doi: 10.1016/j.jpeds.2006.03.024 | Double-Blind, Placebo, Randomised Controlled trial  Children ≤ 16 years; clinical diagnosis of newly diagnosed HSP (purpura, petechiae ± gut/joint pain)  Exclusion criteria: established kidney disease (haematuria > 10 RBC/HPF or proteinuria > 300 mg/L on initial presentation); thrombocytopenia; systemic vasculitis; prednisone contraindicated | N/A | N (analyzed) = 171  Sample (randomised/analyzed): Group 1: (87/84)  Group 2: (89/87)  Mean age, range (years): Group 1: 6.8 (2.0 to 15.2) Group 2: 7.3 (1.7 to 15.6)  Sex (M/F):  Group 1: (49/35)  Group 2 (44/43) | Group 1:  Prednisone: 1 mg/kg/d orally for 14 days; 0.5 mg/kg/d for 7 days; 0.5 mg/kg on alternate days for 7 days  Group 2:  Placebo tablets of similar size | Renal Involvement:  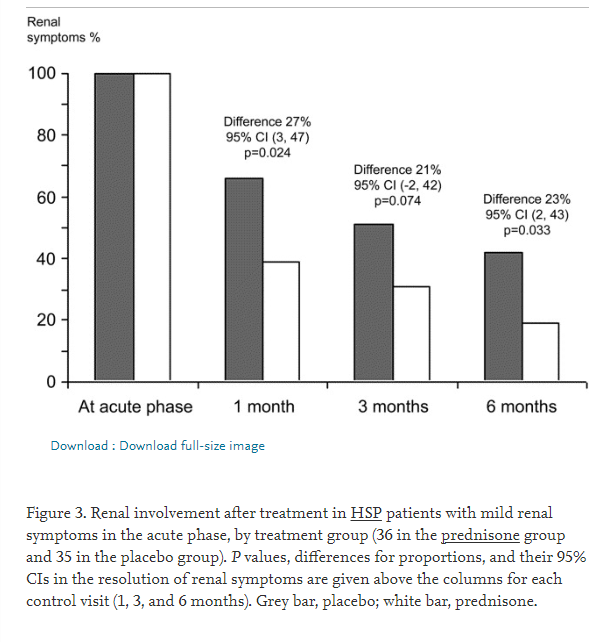  Abdominal & Joint Pain:  Mean sum of scores within 2 weeks was lower in Group 1 for abdominal pain (P=0.029) and joint pain (P = 0.030). Mean sum of total day with abdominal pain was 1.2 days less in group 1 (P = 0.028) and 1.3 days less for joint pain (P=0.076).  Abdominal pain requiring admission:  Group 1: 5 (6%)  Group 2: 9 (10%)  Renal involvement defined using urinary findings (U-protein, U-erythrocytes) |
| Tarshish P, Bernstein J, 2004, Europe, USA, Canada | Henoch-Schonlein purpura nephritis: course of disease and efficacy of cyclophosphamide.  Pediatric Nephrology 2004;19(1):51-6.  doi: 10.1007/s00467-003-1315-x | Randomised Controlled trial  Age 12 weeks to 16 years; HSP: purpura plus urticaria with one or more of the following: joint pain and swelling, kidney disease, abdominal pain and intestinal bleeding; eGFR > 35 mL/min/1.73 m2; proteinuria > 40 mg/m2/hr for > 1 month; histopathology: Crescents/segmental lesions (ISKDC classification)  Exclusion criteria: HSP present > 3 months; prior use of immunosuppressive or cytotoxic therapy other than steroids; concurrent or pre-existing kidney disease | Purpura; Henoch-Schonlein; Cyclophosphamide; Nephritis; Natural History; Pediatrics; Clinical Trial | N = 56  Group 1: 28  Group 2: 28  Age: not reported Sex (M/F): not reported | Treatment group CPA: 90 mg/m2/d orally for 42 days  Control group No therapy | Total number of patients with mild kidney disease  Group 1: 8 (29%)  Group 2: 6 (21%)  Number with severe kidney disease (decreased GFR, severe proteinuria, ESRD)  Group 1: 4 (14%)  Group 2: 4 (14%)  Number of patients with ESRD  Group 1: 3 (11%)  Group 1: 4 (14%)  All comparisons are non-significant between groups |
| Tian M, Liu C, 2015, China | Heparin calcium treated Henoch-Schönlein purpura nephritis in children through inhibiting hyperfibrinolysis  Renal Failure 2015;37(7):1100-4.  doi: 10.3109/0886022X.2015.1061668 | Randomised Controlled trial  Children with HSPN (based on the classification from the Chinese Medical Association (Pediatric Nephrology group)  Exclusion criteria: Hormone therapy, severe infection, severe bleeding tendency, leucopenia, pre-existing shock or HSP patients. | Fibrinolytic System; Henoch-Schönlein Purpura Nephritis; low-molecular-weight-heparin calcium | N = 89  Group 1: 44  Group 2: 45  Mean age ± SD (years): Group 1: 7.94 ± 2.90 years: Group 2: 8.46 ± 2.61 years  Sex (M/F):  Group 1: 25/19  Group 2: 29/16 | Group 1:  low-molecular weight heparin calcium 100 U/kg/d for 4 weeks, then 50 U/kg/d for 4 weeks.  +  Conventional treatment (cimetidine, vitamin C, antihistamines)    Group 2:  Conventional treatment only | HSPN clinical outcomes:  Less failure in Group 1 compared with Group 2 (P=0.02)  Proteinuria:  Less failure and more improvement and excellence in Group 1.  Haematuria:  Non-significant difference between groups.  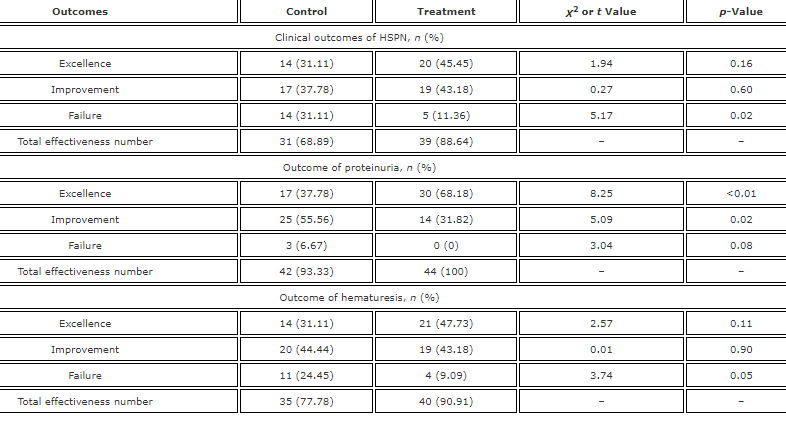  Excellence: Clinical symptoms disappeared, urine protein turned negative and urine RBC disappeared.  Improvement: Clinical symptoms alleviated, urine protein and RBC was reduced.  Failure: Clinical symptoms were not obviously improved and urine protein/RBC were not obviously decreased. |
| Wu SH, Liao PY, 2014, China | Add-on therapy with montelukast in the treatment of Henoch-Schonlein purpura  Pediatrics International 2014;56(3):315-22.  doi: 10.1111/ped.12271 | double-blind, placebo-controlled, parallel paired comparative study  Inclusion: Previously healthy children aged 2-14 years with HSP | Henoch–Schönlein purpura; interleukin; leukotrienes; montelukast; nephritis. | N = 130 Total  Sample 1 -- HSP and no nephritis: N = 84  Sample 2 -- HSP + nephritis (proteinuria and/or hematuria without rapidly progressive nephritis): N = 46  Sample 1:  Group 1: N = 42  Group 2: N = 42  Mean age ± SD (years): Group 1: 6.6 ± 1.7  Group 2 6.8 ± 1.5  Sex (M/F):  Group 1: 26/16  Group 2: 27/15  Sample 2:  Group 1: N =23  Group 2: N = 23  Mean age ± SD (years): Group 1: 7.2 ± 2.0  Group 2 7.3 ± 1.8  Sex (M/F):  Group 1: 13/10  Group 2: 15/8 | Samples 1 and 2  Group 1: Montelukast sodium chewable tablets for 3 months, 4 mg/day (ages 2–5 years), 5 mg/day (ages 6–14 years)  Group 2: Placebo in same dosages as montelukast  Sample 2  Mild nephritis (16 participants with histological grade IIa or proteinuria <25 mg/kg/d): captopril and dipyridamole for 3 months  Moderate nephritis: (22 participants with histological grade IIb, IIIa or proteinuria 25–50 mg/kg per day): captopril, dipyridamole for 3 months; prednisone for 6 months  Severe nephritis: (8 participants with histological grade IIIb, IV, V or proteinuria >50 mg/kg per day): captopril, dipyridamole for 3 months; prednisone for 6 months; mycophenolate mofetil for 6 months | In both samples, significantly different reduction in clinical features of HSP 1, 2, and 4, weeks for treatment Group 1. No difference at 3 months or 6 months.  In Sample 2, treatment group 1 had significantly different reduction in proteinuria and hematuria at 2 weeks, 4 weeks, and 3 months. No difference between groups at 6 months.  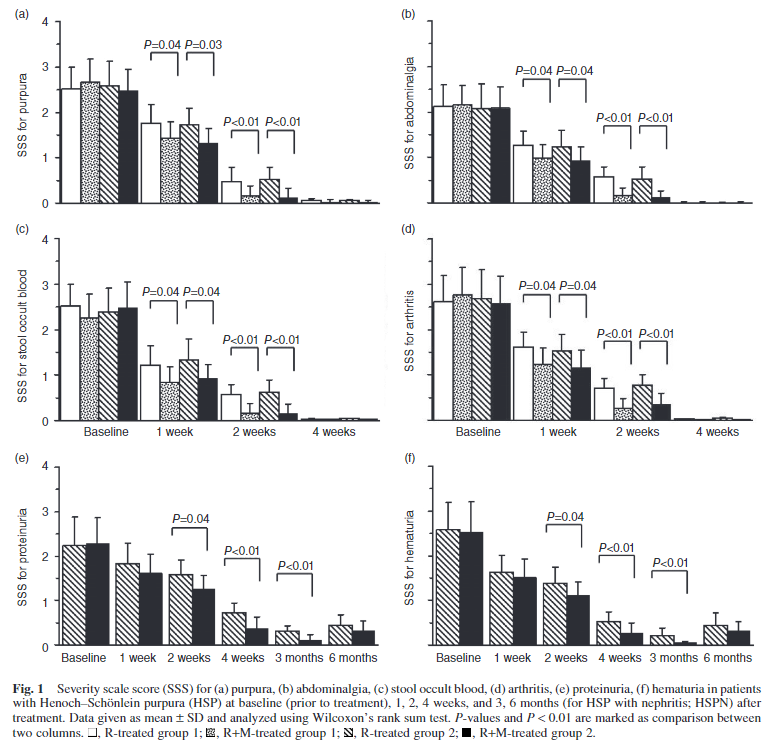  Clinical features, proteinuria, and hematuria measured with Severity Scale Score.  “the 3 month montelukast treatment lowered the HSP relapse rate during the 3 months of treatment, even though the lowered relapse rate with nephritis in sample 1 did not reach statistical significance. The HSP relapse rate, however, did not stay lowered after the 3 month treatment had ended.” |
| Xu J, Chong H, 2009, China  Abstract only | Efficacy of fosinopril on proteinuria in children with Henoch-  Schonlein purpura nephritis  Zhongguo Dangdai Erke Zazhi 2009;11(3):229-30 | Randomised Controlled trial  Children with HSP aged 4 to 14 years; proteinuria ≥ 150 mg/d; medications ceased 2 weeks prior to study | N/A | Number: 48  Group 1: 27  Group 2: 21  Mean age (range): 9 years (4 - 14) Sex (M/F): 28/20 | Group 1:  Fosinopril : < 5 years 5 mg daily 8 weeks, 5 to 12 years 10 mg daily 8 weeks  +  Standard therapy: penicillin, claratyne, fraxiparine, dipyridamole, nifedipine and low salt diet  Group 2: received Standard Therapy. | Proteinuria Complete Relief:  Group 1: 15 (55.6%)  Group 2: 2 (9.5%)  P < 0.05  Proteinuria Partial Relief:  Group 1: 5 (18.5%)  Group 2: 9 (42.9%)  P < 0.05  Partially Valid:  Group 1: 4 (14.8%)  Group 2: 4 (19.1%)  P < 0.05  Invalid:  Group 1: 3 (11.1%)  Group 2: 6 (28.6%)  P < 0.05 |
| Yoshimoto M, Ito H, 1987, Japan  Abstract only | Evaluation of the preventive role of  dipyridamole and aspirin against renal complication  in Schonlein-Henoch purpura  [abstract]  Pediatric Nephrology 1987;1:C47  doi: 10.1007/BF00866894 | Randomised Controlled trial  Inclusion: Children admitted with HSP without kidney disease aged 3 to 10 years  Exclusion criteria: known underlying systemic vasculitis; steroids in previous month; underlying kidney, gastrointestinal or immunodeficiency illness; active infection; a life threatening complication of HSP | N/A | N = 28 Total  Group 1: N = 10  Group 2: N = 9  Group 3: N =9  Age Range: 3 to 10 years Sex (M/F): 13/15 | Group 1: Dipyridamole: 5 mg/kg/d for 5 weeks  Group 2:  Aspirin: 5 mg/kg/d for 5 weeks  Control group: Vitamin pills 2.0g/day for 5 weeks | Renal involvement:  Group 1: 3 (30%)  Group 2: 0 (0%)  Group 3:3 (33%) |
| Zhang H, Li X, 2021, China | Effect and safety evaluation of tacrolimus and tripterygium glycosides combined therapy in treatment of Henoch-Schonlein  purpura nephritis.  International Journal of Urology 2021 Nov;28(11):1157-1163.  doi: 10.1111/iju.14665 | Randomised Controlled trial  Inclusion: Children aged 4 to 18 years admitted with HSPN for at least two months and kidney disease (24-h proteinuria ≥50 mg/kg and urine erythrocyte ≥10/high-power field) for at least one week. No history of glucocorticoid or immunosuppressive drugs, such as tripterygium glycoside, cyclophosphamide or mycophenolatemofetil  Exclusion criteria: known underlying systemic vasculitis; steroids in previous month; underlying kidney, gastrointestinal or immunodeficiency illness; active infection; a life threatening complication of IgAV | Henoch–Schonlein purpura; Henoch–Schonlein purpura nephritis; tacrolimus; Tripterygium glycosides | N = Total of 279 enrolled and 255 evaluated.  Group 1: (enrolled 93/evaluated 87)  Group 2: (enrolled 93/evaluated 85);  Group 3: (enrolled 93/evaluated 83)  Mean age ± SD (years): Group 1 (evaluated): 8.52 ± 3.05 yrs  Group 2 (evaluated): 8.69 ± 3.01 yrs;  Group 3 (evaluated): 8.74 ± 2.79 yrs  Sex (M/F):  Group 1 (evaluated): 43/44 Group 2 (evaluated): 43/42 Group 3 (evaluated): 47/36 | Group 1 (TA):  Tacrolimus orally 0.1– 0.15 mg/kg/day to achieve blood levels of 7–10 ng/mL. Dose reduced by 20-25% every 7-10 days when blood level exceeded range or nephrotoxicity developed. Duration 6 months.  Group 2 (TA + TG): Same as group 1 + Tripterygium 2ml/kg/day for 15 days, reduced to 1.5 ml/kg/day for 15 days, then 1 mg/kg/day for the next 3 months.  Group 3 (Control):  cyclophosphamide: 500–750 m2 IV monthly for six months. Maximum total dose < 150 mg/kg  All Groups:  Prednisolone: 1.5–2 mg/kg/day for 4–8 weeks; then 1.5–2 mg/kg/day every 3 days for 4–8 weeks and reduced gradually and ceased after 6 months | 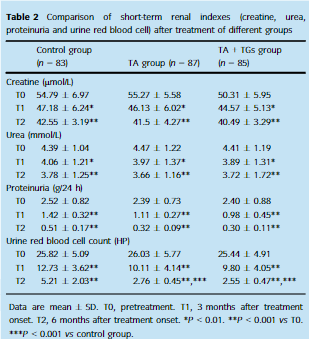  Short term, each treatment saw reduction over time, but were not different from each other at 3 months.  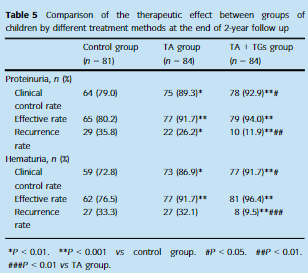  Both treatments reduced proteinuria recurrence and improved control and effective rates. Group 2 reduced recurrence of hematuria more than Group 1. Both groups improve control and effective rate compared with control.  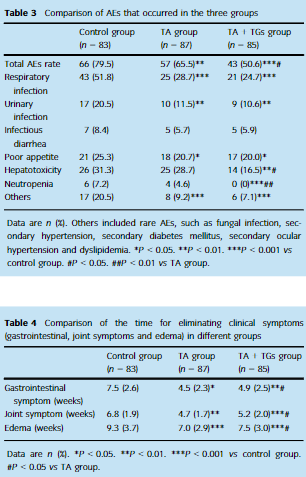  Group 2 demonstrated fewer adverse events than Groups 1 and 3. Group 1 had fewer adverse events than Group 3. |
| Kanik, A., Baran, M.,  2015,  Turkey | Faecal calprotectin levels in children with Henoch–Schönlein purpura: is this a new marker for gastrointestinal involvement?  European Journal of Gastroenterology & Hepatology 2015, 27:254–258 | Prospective observational study.  Objective: To investigate the significance of faecal calprotectin (FC) levels in children diagnosed with Henoch–Schönlein purpura (HSP) and examine its relationships with gastrointestinal system (GIS), renal involvement and with clinical findings.  Inclusion criteria:   1. Pediatric patients first diagnosed with HSP   Exclusion criteria:   1. Cases receiving anti-inflammatory treatment at the time of presentation   Study groups:  Group 1: HSP   - Further grouped based on no-GIS involvement and GIS involvement (mild or severe)   Group 2: Healthy Control  Outcomes:   1. Renal involvement 2. GIS involvement & cases 3. Arthralgia/arthritis 4. Fecal concentrations (FC) | Children, faecal calprotectin, gastrointestinal involvement, Henoch–Schönlein purpura | N= 91 analyzed  Group 1 (HSP):  n=25/25 analyzed  Group 2 (Healthy Control):  n=66/66 analyzed  Mean age:  Group 1: 7.5 (SD ± 2.9)  Group 2: n/a  Sex (M/F): Group 1: 29/37 Group 2: n/a  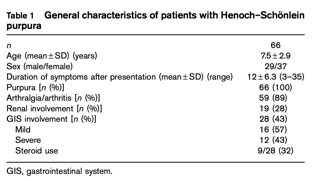 | Group 1 (n=66):  n/a  Group 2 (n=25):  n/a | Renal Involvement, GIS Involvement, and Arthralgia/arthritis  Summarized in table 1  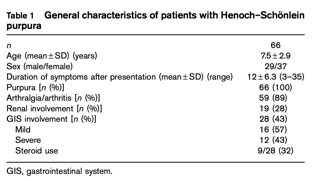  Fecal Concentrations with or without GIS Involvement  Mean FC at 3 days of disease onset (FC1)  Group 1: 111.3 ± 182.7 μg/g  Group 2: 24.5 ± 12.8 μg/g  Significant difference between both groups  P=0.02  GIS-Involvement group: 124.2 (430.7) ug/g   - Mild group: 50.3 (241) ug/g - Severe group: 392 (524.6) ug/g   No-GIS-Involvement group: 16.57 (17.8) ug/g  Significant difference detected between both GIS and no-GIS groups  P=0.02  Difference between Mean FC1 and FC at 15 days (FC2) in GIS Involvement Group  Median FC1: 124.2 (430.7) ug/g  Median FC2: 64.3 (128.3) ug/g  P=0.004  Significant difference observed  Mean FC2  Higher in GIS involvement group  Note: no significant differences in FC2 levels between mild and severe GIS [median, 43.8 (132.0) and 83.6 (118.0) μg/g, respectively].  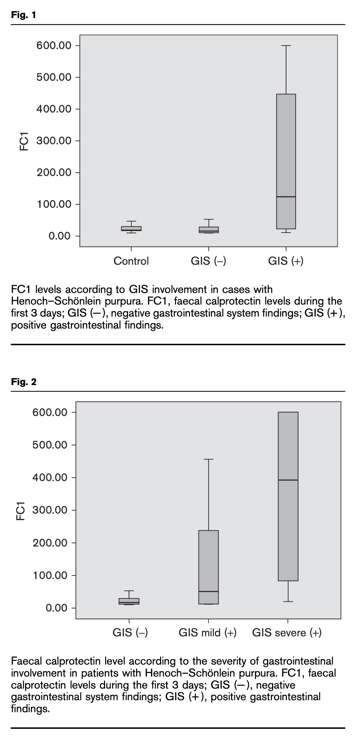  Fecal Concentrations with or without Renal Involvement  Mean FC at 3 days of disease onset (FC1)   - Significantly higher in cases with renal findings compared to those without (P=0.017)   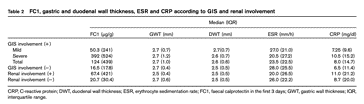  Adverse events  n/a |
| Fan, L., Heng, L.,  2017,  China  Abstract only | Association of serum vitamin D level with severity and treatment in children with Henoch-Schönlein purpura Fan, L., Liu, H., Wang, Y. C., Chen, L., Zhou, J. J., & Cui, Y. X. (2017). Zhongguo dang dai er ke za zhi = Chinese journal of contemporary pediatrics, 19(7), 796–799. https://doi.org/10.7499/j.issn.1008-8830.2017.07.013 | Retrospective study.  Objective: investigate the association of serum vitamin D [25-(OH) D3] level with the severity and treatment in children with HSP.  Inclusion criteria:   1. Newly diagnosed HSP between January and December   Exclusion criteria:  n/a  Study groups:  Group 1: HSP   - Normal group (>20 ng/ml) - Insufficiency group (15-20 ng/ml) - Deficiency group (15 or less ng/ml) - Severe deficiency group (5 or less ng/ml)   Group 2: Control  Outcomes:   1. Fasting serum sample 2. Serum 25-(OH) D3 level 3. Incidence rate of renal involvement 4. Rate of hormone application | n/a | N= 99 analyzed  Group 1 (HSP):  N=50  Normal group (>20 ng/ml)  n=9  Insufficiency group (15-20 ng/ml)  n=15  Deficiency group (15 or less ng/ml)  n=25  Severe deficiency group (5 or less ng/ml)  n=1  Group 2 (Control):  n=49/49 analyzed  Mean age:  Group 1: n/a  Group 2: n/a  Sex (M/F): Group 1: n/a  Group 2: n/a | n/a | Serim 25-(OH) D3 Level   - HSP group: significantly lower serum 25-(OH)D_3_ level than the control group (16±6 ng/mL vs 29±5 ng/mL; P<0.01).   Incidence rate of renal involvement, rate of hormone application, median length of hospital stay   - Compared with the normal and insufficiency groups, the deficiency and severe deficiency groups had significant increases (P<0.05), while there was no significant difference in course of disease before admission (P>0.05). |
| Foster, B.J., Bernard, C.,  2000,  Canada | Effective therapy for severe Henoch-Schonlein purpura nephritis with prednisone and azathioprine: A clinical and histopathological study | Retrospective observational study.  Objective: To validate a scoring system to assess the severity of renal lesions and to correlate histology with clinical findings. Efficacy of treatment with prednisone (1 to 2 mg/kg/d) and azathioprine (1-2mg/kg/d) for severe Henoch-Schonlein purpura nephritis was also examined.  Inclusion criteria:   1. Patients treated between 1965 and 1995 with severe HSPN 2. Patients had to have proteinuria of at least 1g/24 h to be considered for treatment   Exclusion criteria:   1. Those treated for recurrent episodes of gross hematuria, but with <1 g/24 h proteinuria   Study groups:  Group 1: Treatment  Group 2: Control  Outcomes:   1. Histopathological Evaluation 2. Outcome comparison between study and historical groups | Children, faecal calprotectin, gastrointestinal involvement, Henoch–Schönlein purpura | N= 79 analyzed  Group 1 (Treatment):  n=20/20 analyzed  Group 2 (Control):  n=59/59 analyzed  Mean age:  Group 1: 7.5 (SD ± 2.9)  Group 2: n/a  Sex (M/F): Group 1: 12/8  Group 2: n/a | Group 1 (n=20):  Oral prednisone (1 to 2 mg/kg/d) in 3 divided doses for 4 weeks followed by maintenance with prednisone 1-2 mg/kg/d in a single dose every second day   - Tapered toward end of therapy   Azathioprine (1-4mg/kg/d) administered in a single daily dose from the beginning  Total duration of combination therapy: 8-75 weeks (median: 46.7)  After therapy, patients were monitored for median of 5.4 years  Group 2 (n=59):  n/a | Histopathological Analysis   - Proportion of glomeruli with crescents: correlated with clinical severity - Tubulointerstitial score: correlated most strongly with clinical severity - Degree of proteinuria, hypoalbuminemia, and hematuria and presentation of hypertension: only one found to be important was serum albumin, which had a strong negative correlation with tubulointerstitial score at first biopsy (demonstrated by figure 2)   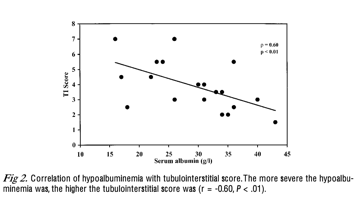  Outcome Comparison Between Study and Historical Groups   - Note: 17 of the 20 patients were included in this comparison   Summarized by table 2 with respect to disease severity:  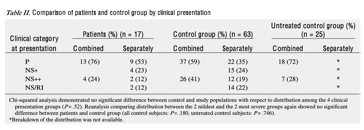  Adverse events   - One patient had severe pancytopenia after 8 days of azathioprine - Most patients had mild cushingoid appearance - Acne developed in a few - 4 patients became more aggressive or emotional with therapy - 4 patients had recurrent gross hematuria without other evidence of recurrent vasculitis |
| J. I. Shin, J. M. Park.,  2009,  Korea | Henoch–Schönlein purpura nephritis with nephrotic‐range proteinuria: histological regression possibly associated with cyclosporin A and steroid treatment  J. I. Shin, J. M. Park, Y. H. Shin, J. H. Kim, J. S. Lee & H. J. Jeong (2005) Henoch–Schönlein purpura nephritis with nephrotic‐range proteinuria: histological regression possibly associated with cyclosporin A and steroid treatment, Scandinavian Journal of Rheumatology, 34:5, 392-395 | Retrospective study.  Objective: To clarify the therapeutic role of cyclosporin A (CyA) for patients with Henoch–Scho ̈nlein purpura nephritis (HSPN) showing nephrotic-range proteinuria.  Inclusion criteria:   1. Children diagnosed with HSPN between 1986 to 2000 2. Had nephrotic-range proteinuria 3. Treated with CyA and prednisolone   Note: nephrotic range proteinuria is defined as urinary protein excretion of >40 mg/m^2^/h  Exclusion criteria:  n/a  Study groups:  n/a  Outcomes:   1. Mean interval between onset of disease and onset of nephritis 2. Mean interval between onset of nephritis and initiation of CyA 3. Renal failure, hypertension, nephritic features at onset 4. Macroscopic hematuria 5. Proteinuria 6. Hematuria 7. Graded clinical outcomes 8. Proteinuria at latest observation 9. End of CyA therapy outcomes   The clinical outcome at the end of therapy and at the latest observation was graded as follows (4):  A, normal urine and renal function;  B, microscopic haematuria or proteinuria v20 mg/m2/h  C, active renal disease with proteinuria w20 mg/m2/h and GFR>60 mL/min/1.73 m2  D, renal insufficiency | n/a | N= 8 analyzed  Mean duration of follow-up from onset of HSP: 3.8 years (1-8)  Mean duration of follow-up after cessation of therapy: 2.2 (0.1-6.5)  Mean age:  7.7 (3.7-15.5)  Sex (M/F): 6/2  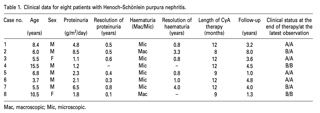 | No groups  Treatment protocol:  The starting dose of CyA (Cipol-N, Chong Kun Dang Pharma) for patients was 5 mg/kg per day and the desired level was kept at 100–200 ng/mL. We monitored the CyA level weekly at the beginning of treatment and thereafter every month. CyA was given for a mean duration of 10.8 months (range 8– 12 months). We started prednisolone at the same time as CyA and it was given as 1–2 mg/kg per day in three divided doses for 8 weeks followed by maintenance with single dose of 1–2 mg/kg per day every alternate day. The mean duration of prednisolone tapering was 1.5 years (range 0.7–2.9 years). | Mean interval between onset of disease and onset of nephritis   - 4 weeks (range: 2-8 weeks)   Mean interval between onset of nephritis and initiation of CyA   - 1 week (range: 0-4 weeks)   Renal failure, hypertension, nephritic features at onset:   - No patients showed these features   Initial Macroscopic Hematuria   - 2 patients (case 2 and 8)   Proteinuria   - Disappeared after mean period of 0.5 years (range: 0.1-0.8 years)   Hematuria   - Resolved after mean period of 1.8 years (range: 0.8-4.0 years)   Graded Clinical Outcomes (at end of therapy)   - State A: 4 patients - State B: 4 patients   Proteinuria at Latest Observation   - Disappeared in 7/8 patients (case 4 had mild proteinuria of 0.3g/m^2^/d   Note: all patients were without any medication at latest observation  End of CyA Therapy Outcomes   - Urinary protein excretion: decreased significantly (p=0.008) - Serum albumin: increased within normal range (p=0.008) - Serum creatinine: no change - Serum creatinine clearance: no change - Activity index: decreased substantially from 8.3+/-1.6 to 3.5+/-1.5 (p=0.031) - Chronicity index and TI scores: did not change   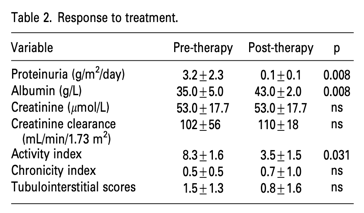  Adverse events   - No serious complications - One patient (case 3) showed characteristic lesions of chronic CyA toxicity |
| Park, J.M., Won, S.C.,  2011,  Korea | Cyclosporin A therapy for Henoch–Schönlein nephritis with nephrotic-range proteinuria  Pediatr Nephrol (2011) 26:411–417 DOI 10.1007/s00467-010-1723-7 | Retrospective study.  Objective: To evaluate the therapeutic role of cyclosporin A (CyA) for the treatment of HSPN.  Inclusion criteria:   1. Patients with HSP that visited the studies’ institution from 1995 to 2009   Exclusion criteria:  n/a  Study groups:  n/a  Outcomes:   1. Development of nephrotic range proteinuria 2. Clinical characteristics 3. Response to treatment 4. Remission 5. Dependence on treatment 6. Clinical status at end of therapy and at latest observation 7. Mean serum albumin, urinary protein excretion, serum creatinine 8. Renal function   The clinical status of each patient at the end of therapy and at the latest observation was classified as follows:   - State A, normal: normal physical examination, urine, and renal function - State B, minor urinary abnormalities: normal physical examination with microscopic hematuria or proteinuria less than 40 mg/m2/h - State C, active renal disease: hypertension or proteinuria of 40 mg/m2/h or greater, with glomerular filtration rate (GFR) of 60 ml/min/1.73 m2 or greater - State D, renal insufficiency: GFR less than 60 ml/min/ 1.73 m2 or greater (including dialysis/transplant or death)   Nephrotic-range proteinuria was defined as proteinuria greater than 40 mg/m2/h or 2.0 mg/mg of spot urine protein/creatinine or 4+ proteinuria on urinalysis.  Nephrotic syndrome was defined as hypoalbuminemia of less than 2.5 g/dl with urinary protein excretion greater than 40 mg/m2/h, hypercholesterolemia, and generalized edema.  The glomerular changes were graded according to the classification of the International Study of Kidney Disease in Childhood (ISKDC):   - grade I, minimal alterations - grade II, mesangial proliferation - grade III (a), focal or (b), diffuse proliferation or sclerosis with <50% crescents - grade IV (a), focal or (b), diffuse mesangial proliferation or sclerosis with 50–75% crescents - grade V (a), focal or (b), diffuse mesangial proliferation or sclerosis with >75% crescents - grade VI, membranoproliferative-like lesion.   Resolution of proteinuria was defined as trace to negative protein on urinalysis examination. | n/a | N= 29 analyzed  Mean duration of CyA treatment: 12.3 months (2.6-55 months)  Mean follow-up were 3.7 years (1.2-12.9 years)  No groups  Mean age:  8.6 (2-15.5)  Sex (M/F): 18/11 | No groups  Treatment protocol:  Received steroids (1 mg/kg/day of prednisolone) for 2 weeks during the acute stage of HSP.  When nephrotic-range proteinuria developed in HSP patients later, oral prednisolone (2 mg/kg/day, maximum 60 mg/day) used for the treatment of HSN before the treatment of CyA.  Note: Intravenous methylprednisolone pulse therapy in any of the patients.  The steroids were tapered off after the initiation of CyA.  Angiotensin-converting enzyme (ACE) inhibitors (ramipril 5 mg once daily) were used concurrently in all patients.  The starting dose of CyA 100 mg, 25 mg capsule) was 5 mg/kg per day, and the desired drug level was kept at 50–150 ng/ml. We never increased the dose of CyA more than 5 mg/kg | Development of Nephrotic-Range Proteinuria   - All patients at a mean interval of 4.4 months   Clinical characteristics  Summarized by table 1  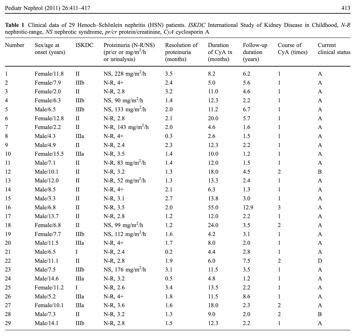  Response to treatment   - Not all patients responded to oral prednisolone - All patients responded to CyA treatment (at mean of 1.8 months)   Remission   - Stable remission: 23 patients (mean follow-up duration of 3.2 years)   CyA Dependency   - 6 patients (patients 12, 16, 18, 22, 27, 28; received more than 2 courses of CyA treatment)   Clinical Status at End of Therapy   - State A: 26 patients (89.6%) - State B: 2 patients (6.9%) - State D: 1 patient (3.5%)   Mean Urinary Protein Excretion   - Baseline: 3,718+/-2,320 mg/day - End of CyA: 80 +/- 60 mg/day [significant decrease]   Mean Serum Albumin   - Baseline: 3.17+/-0.5 g/dl - End of CyA: 4.44 +/- 0.2 g/dl [returned to normal limits]   Mean Serum Creatinine   - Baseline: 0.57+/-0.1 g/dl - End of CyA: 0.69 +/- 0.2 g/dl [returned to normal limits]   Renal Function   - Preserved in all patients except the one who developed ESRD   Adverse events   - Hirsutism (all patients) - Anemia (in 4 patients) - Abdominal symptoms (3 patients) - High levels of lipids (4 patients; returned to normal after cessation of CyA treatment) |
| Niaudet, P., Habib, R., 1998  France | Methylprednisolone pulse therapy in the treatment of severe forms of Schoenlein-Henoch purpura nephritis  Pediatr Nephrol (1998) 12: 238-243 | Prospective study.  Objective: To evaluate the effects of methylprednisolone pulse therapy on the outcome of the nephropathy.  Inclusion criteria:   1. Children with HSP 2. Clinical severity of the renal disease, based on the presence of a nephrotic syndrome (defined by heavy proteinuria R 50 mg/kg per day with hypoprotidemia and hypoalbuminemia < 30 g/l), and/or the severity of the histopathology, based on the presence of crescents in 50%+ glomeruli on renal biopsy   Exclusion criteria:  n/a  Study groups:  n/a  Outcomes:   1. Clinicopathological correlations at the time of the initial renal biopsy 2. Tolerance of treatment 3. Effect of therapy on renal symptoms 4. Clinicopathological correlations at latest follow-up 5. Result of repeat renal biopsies | Schoenlein-Henoch purpura nephritis, Methylprednisolone pulse therapy, Repeat biopsies | N= 38 analyzed  No groups  Mean age:  7 years 7 months (3 – 14 years 2 months)  Sex (M/F): 25/13 | No groups  Treatment protocol:  Methylprednisolone: at a dose of 1000 mg/1.73 m2 given intravenously by means of an electric pump over a 6-h period.   - Each patient received three pulses every other day. - Thereafter, the patients were given oral prednisone, 30 mg/m2 per day for 1 month, 30 mg/m2 on alternate days for 2 months, and 15 mg/m2 for 2 weeks.   Seven patients whose initial renal biopsy had shown 6 80% crescentic glomeruli also received cyclophosphamide 2.5 mg/ kg per day for 2 months and 1 was also treated with plasma exchanges.  Five patients received a second series of methylprednisolone pulses 8 months to 4 years after the first, following an aggravation of renal symptoms with recurrent episodes of macroscopic hematuria and an increase in proteinuria. | Clinicopathological correlations at the time of the initial renal biopsy  Hematuria   - Pre-biopsy: hematuria present in all patients   Proteinuria   - Varied between 75-350 mg/kg/day (mean 162+/-68 mg/kg/day)   Nephrotic Syndrome   - Developed in 36 patients   Creatinine Clearance   - Decreased in 3 patients (range: 55-70 ml/min/1.73m^2^   Tolerance of Treatment   - Methylprednisolone: Well tolerated in all patients without significant side effects - Cyclophosphamide: all patients tolerated it well   Effect of Therapy on Renal Symptoms  Mean urinary protein excretion   - Baseline: 162 +/- 68 mg/kg/d - After treatment (5 moths later): 38+/-15 mg/kg/d - 1-year after start of treatment: 17+/-6 mg/kg/d   Creatinine clearance   - Returned to normal values in 3patients who had mild renal impairment at start   Clinicopathological correlations at latest follow-up  Mean duration of follow-up   - 5 years and 7 months (1-16 years)   Creatinine clearance   - Returned to normal values in 3patients who had mild renal impairment at start   Clinical Recovery   - 27 patients   Minimal urinary abnormalities   - 3 patients   Persistent nephropathy   - 4 patients   Progression to ESRF   - 4 patients   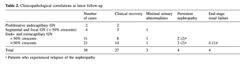  Results of repeat renal biopsies   - Performed in 30 patients   Second Biopsy  5 patients:   - Performed in 5 patients at the time they experienced relapses of the renal disease characterized by recurrent macroscopic hematuria and an increase in proteinuria, 7-24 months after first treatment course - Amount of mesangial IgA deposit: remained constant in 2 patients; increased and associated with peripheral deposits in 3 patients   Remaining 25 Patients:  Remaining 25 patients had second biopsy performed to assess the efficacy of therapy (results correlated with clinical outcome, as shown in Table 3)  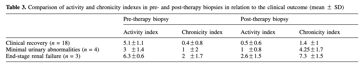  Among the 18 patients who had clinically recovered, the activity index decreased significantly from 5.1+/-1.1 before therapy to 0.5+/-0.6 after therapy (P S 0.0001)   - chronicity index increased significantly from 0.4+/-0.8 to 1.4+/-1 (P < 0.002). - The chronicity index increased from 0 to 1±3 in 11 patients, while it remained unchanged in 7 - Mesangial cellularity returned to normal in all patients.   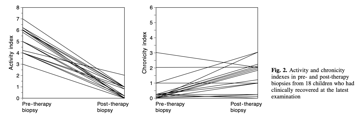  Adverse events  None reported |
| Deng, F., Lu, L.,  2012,  China | Improved Outcome of Henoch-Schonlein Purpura Nephritis by Early Intensive Treatment  Indian J Pediatr (February 2012) 79(2):207–212 | Retrospective study.  Longitudinal  Objective: To evaluate the different therapies used to treat mild, moderate and severe Henoch-Schonlein purpura nephritis (HSPN) patients and to identify the most effective treatment.  Inclusion criteria:   1. Diagnosis of HSP based on EULAR/PReS endorsed consensus criteria 2. Had renal involvement 3. Patients with hospital files in the Pediatric Nephrology Clinics of the first affiliated hospital of Anhui Medical University between 2001 and December 2007   The diagnosis was based on the EULAR/ PReS endorsed consensus criteria proposed by Ozen and coworkers as following: Palpable purpura (mandatory criterion) and the presence of at least one of the following four features: (1) diffuse abdominal pain, (2) any biopsy showing predominant IgA deposition, (3) arthritis or arthralgia, and (4) renal involvement (any hematuria and/ or proteinuria)  Exclusion criteria:  n/a  Study groups:  Group 1: Mild condition  Group 2: Moderate condition  Group 3: Severe condition  “mild group” was defined by the presence of mild proteinuria (<20 mg/m2/h) and/or hematuria  “moderate group” was defined by the presence of moderate proteinuria (20–40 mg/ m2/h) or acute nephritic syndrome (microscopic or macro- scopic hematuria with at least 2 of the following 3 findings: oliguria, hypertension, and raised serum urea or creatinine)  “severe group” was defined by the presence of one or more of the following signs and symptoms: proteinuria > 40 mg/ m2/h, rapidly progressive glomerulonephritis, nephrotic syndrome or a renal biopsy specimen showing cellular crescents  Outcomes:   1. Abnormal urinalysis 2. Blood pressure at onset 3. Serum urea 4. Creatinine 5. Differences in sex, age at diagnosis, and amount of proteinuria 6. Treatment effects at 4 wk 7. Recovery 8. Difference between non-steroid therapy and HCSS therapy   Follow-up results | Henoch-Schonlein purpura, nephritis, childhood, therapy | N= 186 analyzed  Group 1 (Mild):  n=65/65 analyzed  Group 2 (Moderate):  n=83/83 analyzed  Group 3 (Severe):  n=38/38 analyzed  Mean age:  Group 1a (non-steroid group): 9.56 (SD ± 2.43)  Group 1b (HCSS therapy group): 8.93 (SD ± 2.07)  Group 2a (HCSS therapy group): 10.03 (SD ± 2.32)  Group 2b (MP therapy): 10.09 (SD ± 2.29)  Group 3a (MP therapy group): 9.86 (SD ± 1.79)  Group 3b (MP + TG therapy): 10.25 (SD ± 2.42)  Sex (M/F): Group 1: 38/27  Group 2: 54/29  Group 3: 18/20 | Group 1 (n=65):   - Non-steroid group - HCSS therapy group   Group 2 (n=83):   - HCSS therapy group - MP therapy group   Group 3 (n=38):   - MP therapy group - MP + TG therapy group   Treatment protocol:  All patients received anticoagulant, antioxidant (vitamin C) and angiotensin-converting enzyme inhibitor (ACEI) therapy (Ramipril: 2.5–5 mg/day for 3–6 months).  The mild group was divided into non-steroid group and hydrocortisone sodium succinate (HCSS)group:   - Patients in non-steroid group were just given above therapy - Patients in the HCSS therapy group were initially treated intravenously with HCSS 4–8 mg/kg daily for 1–2 wk, then administered oral steroid at 0.5–1 mg/kg daily.   The moderate group was divided into HCSS and methylprednisolone (MP) pulse therapy groups   - Patients in MP pulse therapy group were initially treated intravenously with MP 10–15 mg/kg for three or six alternate days, then were given oral steroid at 0.5–1 mg/kg daily.   The severe group was divided into MP pulse and MP in combination with tripterygium glycoside (TG) therapy groups   - Patients in the MP combination with TG therapy group were initially treated intravenously with MP 10–15 mg/kg for six alternate days, then were given oral steroid at 0.5–1 mg/kg daily, while at - the same time given oral TG at 1 mg/kg daily for 3–6 months. | Abnormal urinalysis   - All patients - 3 had hematuria - Other 183 had proteinuria   Blood pressure at onset   - All patients had normal   Serum urea and Creatinine   - Elevated in 3 patients (grouped into MP + TG group)   Differences in sex, age at diagnosis, and amount of proteinuria in mild, moderate, severe groups   - No differences between two therapy groups   Treatment effects at 4 wk  Shown in table 2  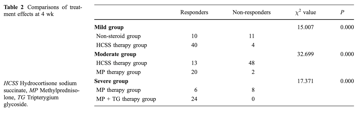  Recovery   - 113 patients (60.8%) had obvious recovery (they all had disappearance of hematuria or decreasing proteinuria)   Difference between non-steroid therapy and HCSS therapy   - Mild group: difference was statistically signficiant - Moderate group: more likely to respond to MP therapy than to HCSS (P<0.05) - Severe group: more likely to respond to MP in combination with TG than to single MP therapy (P<0.05)   Follow-Up Results  Shown in table 3  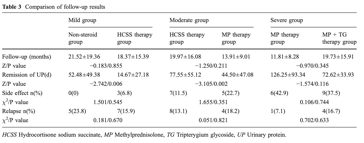  Complete remission   - All patients   Urinalysis, blood pressure, serum urea, serum creatinine   - All patients had normal levels   Time for the Remission of Proteinuria   - Severe group: not statistically significant (P>0.05) - Mild group: shorter in the HCSS group than non-steroid group (P<0.05) - Moderate group: shorter in MP pulse therapy than HCSS therapy (P<0.05)   Relapse   - 29 patients (all had proteinuria recurrence) - Most had one relapse; six patients had 2 or 3 relapses - No significant difference in relapse between different therapies (P>0.05)   Adverse Effects   - Liver functional impairment: 7 patients   Hypertension: 23 patients |
| Rosenblum, N.D.,  1987,  USA | Steroid Effects on the Course of Abdominal Pain in Children With Henoch-Schonlein Purpura  PEDIATRICS Vol. 79 No. 6 June 1987 | Retrospective study.  Objective: To assess the effect of corticosteroids on the outcome of abdominal pain in children with HSP.  Inclusion criteria:   1. Children with HSP admitted to The Children’s Hospital, Boston between 1974-1985   A diagnosis of Henoch-Schonlein purpura was made if the major manifestations of the illness consisted of a purpunic rash and abdominal pain without thrombocytopenia. Additional features including arthritis and nephnitis were accepted as being consistent with the diagnosis.  Exclusion criteria:  n/a  Study groups:  Group 1: Steroid treated  Group 2: Non-steroid Treated  Outcomes:   1. Clinical features   Treatment and outcome | n/a | N= 48 analyzed  Group 1 (Steroid):  n=n/a analyzed  Group 2 (Non-steroid):  n=n/a analyzed  Mean age:  Group 1: n/a (SD ± n/a)  Group 2: n/a (SD ± n/a)  Sex (M/F): Group 1: n/a  Group 2: n/a | Group 1 (n=65):   - Non-steroid group - HCSS therapy group   Group 2 (n=83):   - HCSS therapy group - MP therapy group   Treatment protocol:  Prednisone: 1-2mg/kg/d  Steroid regimen was introduced within 4 days of onset of abdominal pain in 75% and within 12 days in 96% of steroid-treated patients | Clinical Features  Abdominal Pain   - 43 patients - Chief complaint in all patients - Note: those with this complaint were included in the study - Presented within 8 days of onset of rash in 75% of patients   Rash   - 42/43 (97%) patients - 4/42 patients: rash was atypical   Arthritis   - 28/43 patients (65%)   Preceding Infection   - 16/43 (37%) patients (episodes were either an upper respiratory tract infection or pharyngitis)   Vomiting, melena, and hematemesis   - Occurred in 60%, 19%, and 7% of patients   Abdominal tenderness   - 75% of patients (but rebound tenderness was infrequent (9%))   Guaiac positive stool   - ~one half of patients   Treatment and Outcome  Steroid treatment   - Treated with prednisone: 25 (58%) - Not treated with prednisone: 18 (42%)   Other therapy   - IV hydration: 22/43 (51%) - Total restriction of oral intake: 19/43 (44%) - Continuous nasogastric suction: 6/43 (14%) - Antibiotic therapy: 7/43 (16%)   Resolution of abdominal pain   - 42/43 (97%) - Steroid group: pain resolved within 24 hours of beginning of therapy in 44% and within 48 hours in 65% of patients - non-steroid: pain resolved within 24 hours of admission in 14% and within 48 hours in 45% (figure 2)   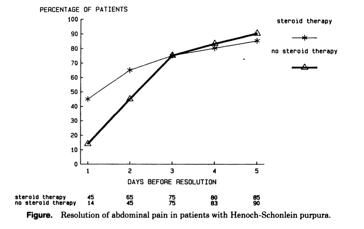  Adverse Effects  Intussusception in one patient |
| Zhao, Z., Hao, L.,  2017,  France  Abstract only | OFF-LABEL USE OF TACROLIMUS IN CHILDREN WITH HENOCH-SCHONLEIN PURPURA NEPHRITIS: EFFICACY AND SAFETY | Retrospective study.  Objective: to assess its efficacy and safety of tacrolimus in treatment of HSPN in children  Inclusion criteria:   1. Children with HSPN receiving tacrolimus and steroids as empirical treatment   Exclusion criteria:  n/a  Study groups:  n/a  Outcomes:   1. Proteinuria 2. Duration of treatment   Pharmacogenetic analysis was performed on the CYP3A5 gene. | n/a | N= 25 analyzed  No groups  Mean age:  7.2 (3-12 years)  Sex (M/F):  n/a | n/a | Proteinuria   - Returned to negative in 21 patients with a mean treatment duration of 101 (SD 75) days.   Duration of treatment   - Patients with CYP3A5*1/*3 had longer duration of treatment achieving negative proteinuria as com- pared with patients with CYP3A5*3/*3 (131±97 versus 80 ±39 days).   No patients discontinued the tacrolimus treatment due to adverse events, and no drug-related adverse events were shown to have a causal association with tacrolimus therapy. |
| Shin, J.I., Park, J.M.,  2005,  Korea | Can azathioprine and steroids alter the progression of severe Henoch-Schonlein nephritis in children?  Pediatr Nephrol (2005) 20:1087–1092 DOI 10.1007/s00467-005-1869-x | Non-randomized concurrent cohort comparison  Objective: To evaluate the effect of azathioprine with steroids on the clinical course and histologic parameters of severe HSN.  Inclusion criteria:   1. HSN patients with nephrotic-range proteinuria 2. Aged 4.4-17 years 3. Referred to study’s unit between 1986 and 2000   Exclusion criteria:   1. Patients previously treated with other cytotoxic drugs 2. Patients who received azathioprine at 14 months from the onset of nephritis   Study groups:  Group A: Azathioprine with steroids (combined)  Group B: Steroids  Outcomes:   1. Characteristics at onset 2. Outcome classification 3. Repeat biopsies   The clinical status of each patient at the end of therapy and at the latest observation was classified as follows:  –  State A. Normal: normal physical examination, urine, and renal function  –  State B. Minor urinary abnormalities: normal on physical ex- amination with microscopic hematuria or proteinuria less than 40 mg/m2/h  – State C. Active renal disease: proteinuria of 40 mg/m2/h or greater or hypertension, and glomerular filtration rate (GFR) of 60 ml/min/1.73 m2 or greater  – State D. Renal insufficiency: GFR less than 60 ml/min/1.73 m2 (including dialysis/transplant or death) | Azathioprine, Henoch-Schönlein nephritis, Nephrotic-range proteinuria, Steroids | N= 20 analyzed  Group A (Steroid):  n=10/10 analyzed  Group B (Non-steroid):  n=10/10 analyzed  Mean age:  Group A: n/a (SD ± n/a)  Group B: n/a (SD ± n/a)  Sex (M/F): Group A: n/a  Group B: n/a | Group A (n=10):  Initial pulse methylprednisolone (six alternate-day i.v. doses of 30 mg/kg; maximum dose 1.0 g) followed by oral prednisolone (2 mg/kg/day in three divided doses for 4 weeks).  The patients received 2 mg/kg/day of azathioprine for 8 months. Once azathioprine treatment began, daily oral prednisolone was converted to single-dose alternate-day (2 mg/kg/day for 2 months) and gradually reduced over the period of azathioprine treatment (1.5=> 1.0=>0.5 mg/kg/day, each for 2 months)  Group B (n=10):  Oral prednisolone was started at the daily divided-dose (2 mg/kg/day for 4 weeks) and was gradually reduced to single-dose alternate-day of 5–10 mg/day (1.5=>1.0=>0.5 mg/kg/day=>5–10 mg/day, each for 2 months).  Note: For those patients who had shown mild proteinuria after therapy the prednisolone therapy in both groups extended to the time when desired outcome was achieved. Therefore, the duration and tapering period of prednisolone therapy were similar in both groups, ranging 9–19 months (median 1.7 years), which were longer than those of azathioprine.  Note: Decision as to whether a patient should be treated with azathioprine was influenced by the treating nephrologist’s choice  Note: median duration of follow-up: 4.8 years (1-14 years) | Characteristics at onset  Summarized by table 1  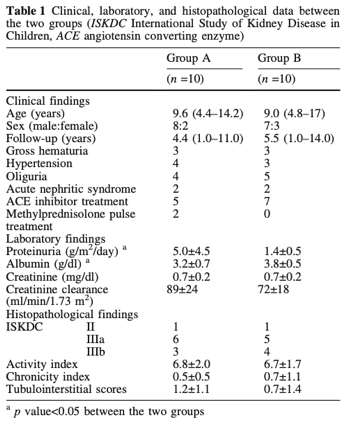   - No differences in clinical, lab, and histopathological severities between the 2 groups, except for significantly lower values of serum albumin and higher levels of urinary protein excretion in group A   Outcome  Rate of Resolution of Proteinuria or Hematuria   - No significant differences between the two groups   Note: One patient in group B had active renal disease; 10 patients in group A showed normal urinalysis or minor urinary abnormalities at latest observation  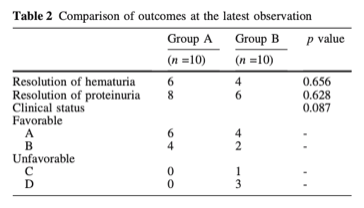  Clinical and Histological Features of the Patients who progressed to chronic renal insufficiency   - Shown in table 3 - All 3 patients had hypertension and one of the three had acute nephrotic syndrome   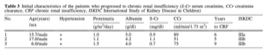  Repeat Biopsies  Light and immunofluorescent (IF) findings of the 10 patients treated with azathioprine are shown in table 4  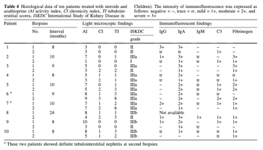  Histological Grades of ISKDC   - Pre-therapy: one grade II; six grade IIIa; three grade IIIb - Post-therapy: one grade I, four grade II, four grade IIIa, one grade IIIb   Activity Index   - Significantly decreased from 6.8+/-2 to 4.3+/-2.3   Chronicity Index   - Increased statistically (0.5+/-0.5 vs 1.4+/-1.1, p=0.031), and the   TI Scores   - Increased with borderline significance (1.2+/-1.1 vs 2.5+/-2.7, p=0.063) compared with initial biopsy   Adverse Effects  None reported. |
| Singh, S., Devidayal, L.K., 2002  India | Severe Henoch-Schonlein nephritis: resolution with azathioprine and steroids  Rheumatol Int(2002)22:133–137 DOI 10.1007/s00296-002-0208-9 | Retrospective  Objective: To assess the efficacy long-term azathioprine and steroids for treatment of severe nephritis in children with Henoch-Schonlein purpura (HSP).  Inclusion criteria:   1. Diagnosis of HSN   Diagnosis of HSN was based on the presence of hematuria and/or proteinuria along with characteristic purpuric rash and abdominal or joint pain, or both, plus evidence of leukocytoclastic vasculitis on skin biopsy  Exclusion criteria:  n/a  Study groups:  Group 1: Oral steroids only   - For 2 patients whose biopsies showed little or no changes on light microscopy   Group 2: aggressive therapy with steroids and Azathioprine (combined)   - For other 9 patients   Outcomes:   1. Clinical and laboratory data (for the 9 patients treated with steroids and azathioprine) 2. Renal histopathological evaluation   Definitions used   1. Hematuria: ‘‘microscopic’’ if urine examination showed five or more RBCs/HPF and ‘‘gross’’ if visible to the naked eye [11] 2. Gross proteinuria: >40 mg/m2 per h with or without edema [11] 3. Acute nephritic syndrome: hematuria with at least two conditions among hypertension, increased plasma creatinine, and oliguria caused by nephritis [8] 4. Hypertension: systolic or diastolic blood pressure >95th per- centile for the specific age, based on the Second Task Force recommendations [12]   Clinical status evaluation  Clinical status was evaluated on initial encounter with the patient and at the latest observation, and each patient was classified into clinical state A, B, C, or D as adapted from the Goldstein et al. [13] classification on outcome in HSN:  A. Normal: normal physical examination, urine and renal function  B. Minor urinary abnormalities: microscopic hematuria or proteinuria (<40mg/m2per h) but normal physical examination  C. Active renal disease: proteinuria of 40mg/m2per h or more and serum creatinine <3 mg% with or without hematuria and hypertension  D. Renal insufficiency: serum creatinine >3 mg%, ESRD  Note: Ten out of 11 patients were categorized with clinical status C, while one patient had clinical status B at the time of initial evaluation (Table 1). The majority of our patients had hypoalbuminemia, the mean serum albumin being 3.3 g/dl (range 2.8–3.6 g/dl). | Henoch-Schonlein nephritis, Immunosuppressive therapy, Azathioprine, Steroids | N= 11 analyzed  Group 1 (Oral Steroids):  n=2/2 analyzed  Group 2 (Combined):  n=9/9 analyzed  Mean age:  Group 1: n/a (SD ± n/a)  Group 2: 9.4 years (range 6-12)  Sex (M/F): Group 1: n/a  Group 2: 6/4 | Group 1 (n=2):  Treated with oral prednisolone alone  Group 2 (n=9):  Seven out of nine received initial pulse steroids intravenously (methylprednisolone 30 mg/kg per day or dexamethasone 5 mg/kg per day) for 3 days followed by oral prednisolone, while the remaining two received oral steroids, right from the beginning, along with azathioprine (usually 2 mg/kg per day).  Prednisolone was given as 1–2 mg/kg per day in three divided doses for approximately 8 weeks followed by maintenance with single dose of 1– 2 mg/kg per day every alternate day. This was tapered off once the urinary abnormalities had stabilized for 2–3 months. The duration of prednisolone therapy ranged from 6–16 months (mean 12.1). Azathioprine was given for a mean duration of 14.7 months (range 6–24) and tapered off over 1–2 months. Supportive therapy was given for other manifestations (e.g., hypertension) as and when required. | Characteristics at onset  Summarized by table 1  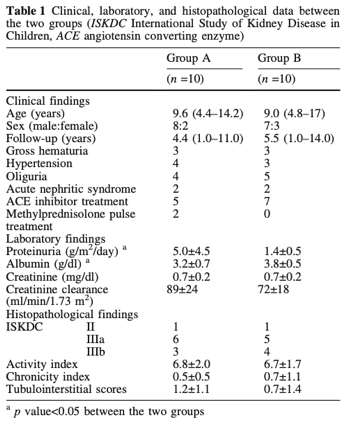   - No differences in clinical, lab, and histopathological severities between the 2 groups, except for significantly lower values of serum albumin and higher levels of urinary protein excretion in group A   Initial gross proteinuria   - All patients - Mean: 3.9 g/m^2^ per day   Recurrence of proteinuria   - Group 2: occurred twice in one of the nine patients (patient 4)   Hematuria   - Gross hematuria: 6 patients - Microscopic hematuria: 5 patients   Hematuria resolution   - Group 2: Occurred over a mean of 6.6 months   Clinical status   - Group 2: improved from status C at onset to status A at latest observation - Group 1: one patient (patient 10) improved from status C at onset to status A at latest observation   Renal histopathological evaluation  Shown by table 2  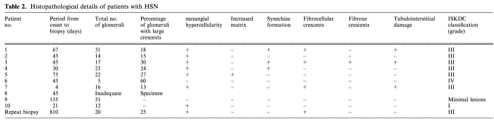  Mean interval between onset of nephritis and performance of kidney biopsy   - 50.6 days   Average number of glomeruli per specimen   - 20.5 days   Features of activity (mesangial hypercellularity, cellular crescents, increased matrix, lobular accentuation)   - Noted in 10/12 specimens   Features of chronicity (fibrous crescents, synechiae formation, tubulointerstitial damage, glomerulosclerosis)   - Seen in 4/12 specimens   Glomerular changes (according to ISKDC classification)   - Grade 3: 7 patients - Grade 4: 1 patient - Grade 1 progression to grade 3: patient 11   Deposition of IgA (revealed by immunofluorescence studies)   - Significant deposition (2+ to 3+) mainly in mesangium   Deposition of fibrin and C3   - Variable deposits in mesangium and capillary loops   Repeat Biopsies  Light and immunofluorescent (IF) findings of the 10 patients treated with azathioprine are shown in table 4  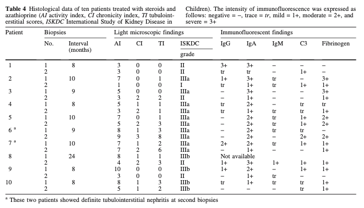  Histological Grades of ISKDC   - Pre-therapy: one grade II; six grade IIIa; three grade IIIb - Post-therapy: one grade I, four grade II, four grade IIIa, one grade IIIb   Activity Index   - Significantly decreased from 6.8+/-2 to 4.3+/-2.3   Chronicity Index   - Increased statistically (0.5+/-0.5 vs 1.4+/-1.1, p=0.031), and the   TI Scores   - Increased with borderline significance (1.2+/-1.1 vs 2.5+/-2.7, p=0.063) compared with initial biopsy   Adverse Effects  None reported |
| Lu, Z., Song, J.,  2017,  China | Evaluation of Mycophenolate Mofetil and Low- Dose Steroid Combined Therapy in Moderately Severe Henoch-Schönlein Purpura Nephritis  Med Sci Monit, 2017; 23: 2333-2339 DOI: 10.12659/MSM.904206 | Retrospective  Objective: To evaluate the clinical therapeutic effects of mycophenolate mofetil and low-dose steroid in Henoch-Schönlein purpura nephritis (HSPN) with nephrotic-range proteinuria and pathological classification less than IV in children  Inclusion criteria:   1. Children hospitalized in the Department of Nephrology, the Children’s Hospital of Zhejiang University School of Medicine from 2012-2015 2. Pediatric HSPN patients with nephrotic-range proteinuria 3. Normal kidney function 4. <50% crescents or sclerosing lesions on renal biopsy   Exclusion criteria:   1. Patients with ISKDC grade more than grade III   Study groups:  Group 1: Administration of CellCept plus low-dose prednisone (MMF+GC)  Group 2: full-dose prednisone (GC)  Outcomes:   1. Baseline patient characteristics 2. Pathological characteristics 3. Short term response to therapy and long-term follow-up 4. Description of MPA-AUC and ROC curve analysis 5. Survival curve analysis 6. Adverse effects and extra-renal manifestations during follow-up   Clinical outcome was graded as A, B, C, and D according to Meadow’s criteria [18]. Category A was favorable outcomes, while categories B, C, and D were unfavorable outcomes. | Henoch-Schonlein nephritis, Immunosuppressive therapy, Azathioprine, Steroids | N= 61 analyzed  The patients were followed up (median: 23 months, range: 12~44 months).  Group 1 (MMF+GC):  n=41/41 analyzed  Group 2 (GC):  n=20/20 analyzed  Mean age:  Group 1: 7.61 (SD ± 2.87)  Group 2: 7.79 years (SD ± 2.88)  Sex (M/F): Group 1: 23/18  Group 2: 9/11 | Group 1 (n=41):  Received oral CellCept® at a dosage of 20–30 mg/kg a day with a course of about 1 year and the dose was not changed during the first 6 months unless the drug had intolerable adverse effects such as severe gastrointestinal reaction, drug allergy, and severe liver or kidney function damage.  Prednisone at a dosage of 1 mg/kg·d (maximum daily dose 30 mg) was given for 4 weeks, after which prednisone was tapered off gradually within 6–9 months. We reduced the prednisone dose by about 5 mg/m2 every 4 weeks until the end of the study  Group 2 (n=20):  Treated with 2 mg/kg·d of prednisone (maximum daily dose 60 mg) alone for 4 weeks, reduced to 1 mg/kg for 4 weeks, and then tapered off gradually within 6–9 months.  Note: Patients in both groups also received ACEI and supportive therapies. | Baseline patient characteristics  Shown in table 1  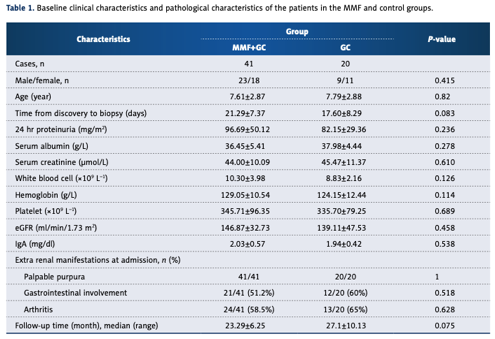  Pathological characteristics of the patients  Shown in table 2  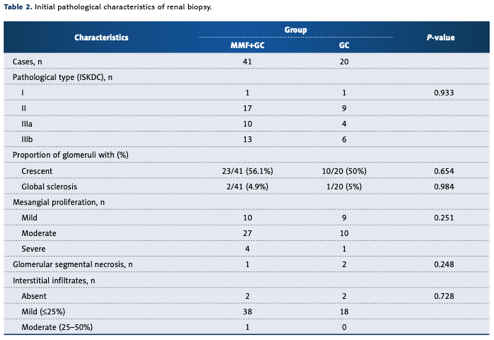  Most common histological type   - Grade III (33/61, 54.1%)   Most common renal pathological lesions   - Moderate mesangial proliferation (37/61, 60.7%) - Mild interstitial infiltrates (56/61, 91.8%)   Note: no significant differences in the pathological characteristics between the 2 groups were found  Short-term response to therapy and long-term follow-up  Shown in table 3  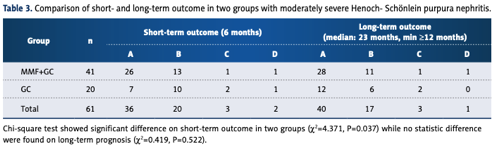   - Short-term follow-up: significant differences between 2 groups (P=0.037) - Long-term follow-up: no significant differences were found (P=0.522)   Description of MPA-AUC and ROC curve analysis  Group 1:   - 34.53±8.86μg·h/mL, ranging from 19.04 to 63.74 μg·h/mL - Short-term follow-up: 26/41 children (63.4%) in remission   Survival curve analysis   - Difference in long-term prognosis (the remission of proteinuria) between the 2 groups was analyzed by Kaplan-Meier curves; no significant difference was found (c2=1.112, P=0.292) (Figure 2).   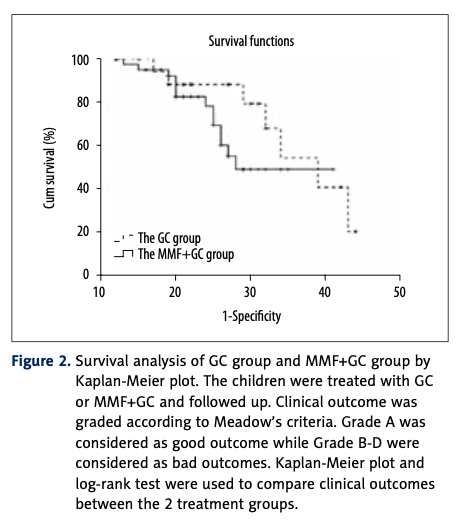  Adverse effects and extra-renal manifestations during follow-up  Listed in table 4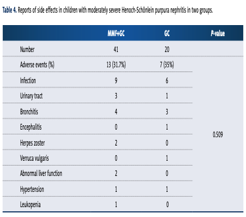 |
| Koskela, M., Jahnukainen, T.,  2019,  Finland | Methylprednisolone or cyclosporine a in the treatment of Henoch-Schönlein nephritis: a nationwide study  Pediatric Nephrology (2019) 34:1447–1456 https://doi.org/10.1007/s00467-019-04238-2 | Retrospective  Longitudinal  Objective: To describe the long-term outcome of these patients to analyze the efficacy of our current HSN treatment strategy  Inclusion criteria:   1. Pediatric HSN patients treated with MP or CyA in 1996-2011 at pediatric nephrology clinics of the 5 university hospitals in Finland 2. Childhood onset (age<17 years) onset HSN with MP or CyA as initial immunosuppressive therapy   Exclusion criteria:   1. Patients with ISKDC grade more than grade III   Study groups:  Group 1: methylprednisolone pulses (MP)  Group 2: cyclosporine A (CyA)  Outcomes:   1. Treatment response 2. Outcome at the end of follow-up 3. Five-year renal prognosis 4. Health questionnaire 5. Side effects | Outcome, Immunosuppression, Angiotensin-converting enzyme inhibitor, Vasculitis, IgA glomerulonephritis, Nephrotic syndrome, Children | N= 62 analyzed  The patients were followed up (median: 23 months, range: 12~44 months).  Group 1 (MP):  n=42/42 analyzed  Group 2 (CyA):  n=20/20 analyzed  Mean age:  All:  9.9 (SD ± 3.4)  Group 1: 9.5 (SD ± 3.3)  Group 2: 10.7 (SD ± 3.4)  Sex (M/F):  All: 36/26  Group 1: 24/18  Group 2: 12/8  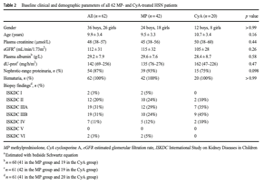 | Treatment Protocol   - Treatment of all 62 patients was initiated either with MP or CyA   MP treatment consisted of three i.v. pulses with a dosage of 15– 30 mg/kg (maximum 1 g) given within a period of 1 week. MP pulses were followed by oral prednisone 30 mg/m2 for 1 month, after which the aim was to taper steroid treatment in 3 months.  CyA was started with an initial trough level of 150–200 μg/L for 3 to 6 months. After that, the dosage was lowered, aiming at a trough level of 80–100 μg/L, so that the total treatment time would be 1–2 years | Baseline patient characteristics  Shown in table 2  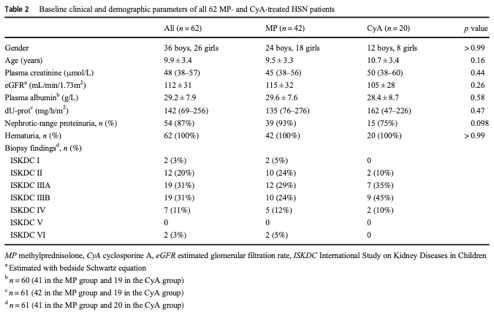  Treatment Response  Switch of Treatment   - Group 1: 16 (38%) group 1 patients were switched to group 2 treatment after median of 2 months - Group 2: 2 (10%) group 2 patients received MP pulses and oral prednisone after a mean of 1.1 months after initial treatment   Additional Immunosuppressive Therapy   - Higher among patients treated initially with MP compared to patients treated with CyA (MP 38% vs. CyA 10%, RR 3.81, 95% CI 1.16 to 14.3, p = 0.035)   ESRD Development   - 1 patient (this patient had nephrotic-range proteinuria and ISKDC grade IIIa)   Renal Function   - Decreased renal function (eGFR < 60ml/min/1.73m^2^): 1 patient (had nephrotic range proteinuria and ISKDC grade IV - Mildly decreased renal function (eGFR 60– 89 mL/min/1.73m^2^): 6 patients (10%, 5 MP, 1 CyA)   PCr, Cystatin C, Urea, eGFR   - No differences between initial MP and CyA treatment groups at end of follow-up   Proteinuria and/or Hematuria at end of follow-up   - 18 (29%) patients (three having both)   Proportions of Patients with Proteinuria, Blood Pressure Medication, or Hematuria (according to initial treatment at the end of follow-up)   - No significant differences   5-Year Renal Prognosis  eGFR from time of renal biopsy to 5-year follow-up shown in figure 2a  patients with eGFR values available from all measurement points shown in figure 2b  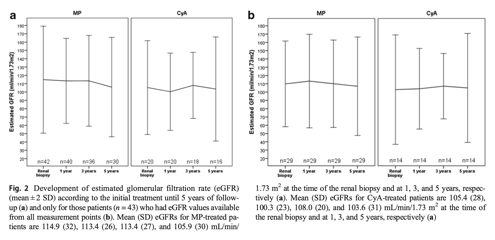  Difference of means between MP and CyA-treated patients   - Before treatment: 9.5 - 1-year follow-up: 13 - 3-year follow-up: 5.4 - 5-year follow-up: 2.3   Health Questionnaire  Obtained from 41 (66%) patients  Report of recurrence of extrarenal HSP   - 10 patients   Report of recurring symptoms during or after an infectious disease   - 5 patients   Report of degenerations in spinal vertebrae   - 1 patient   Report of pregnancies   - 4 patients (3 (2 MP and 1 CyA) had proteinuria and/or hypertension as a pregnancy complication)   Report of HSP being diagnosed in a first degree relative   - 1 patient   Adverse Effects  Most common ones of MP and prednisone treatment:   - Weight gain (74%) - Cushings symptoms (57%) - Striae (23%) - Mood fluctuations (23%)   Most common for CyA treatment:   - Elevations of PCr (treatment had to be stopped, but was recontinued) - Convulsions (6%) - Hirsutism (61%) - Gingival hypertrophy (25%) - Headache (19%) |
| Chen, L., Wang, X.,  2020,  China | Effects of dexamethasone and gamma globulin combined with prednisone on the therapeutic effect and immune function of Henoch-Schonlein purpura nephritis in children  J Clin Lab Anal. 2021;35:e23580. https://doi.org/10.1002/jcla.23580 | Retrospective  Objective: To compare and analyze the therapeutic effect of dexamethasone and gamma globulin combined with prednisone on children with HSPN, to find a better treatment for HSPN.  Inclusion criteria:   1. Children who met the clinical diagnostic criteria of HSPN   Exclusion criteria:   1. Children treated with other hormones and immunosuppressants other than those used in this study within 1 week 2. Children with renal injury caused by viral hepatitis, vasculitis, lupus erythematosus, or other diseases 3. Children with nephrotic syndrome 4. Children with other system disorders   Study groups:  Group 1: treatment (dexamethasone and gamma globulin combined with prednisone)  Group 2: control (dexamethasone and gamma globulin)  Outcomes:   1. Comparison of clinical manifestations between two groups 2. Comparison of therapeutic effects between the 2 groups of children treated by different treatment methods 3. Comparison of immune function between the two groups before and after treatment 4. Changes of serum inflammatory factors in two groups before and after treatment 5. Changes of coagulation function in the two groups before and after treatment 6. Comparison of urine routine indexes between the two groups before and after treatment   Comparison of renal function indexes between the two groups before and after treatment | Dexamethasone, gamma globulin, immune function, pediatric Henoch-Schonlein purpura nephritis, prednisone | N= 115 analyzed  Group 1 (Treatment):  n=55/55 analyzed  Group 2 (Control):  n=60/60 analyzed  Mean age:  All patients: 6.49 (SD ± 1.42)  Sex (M/F):  65/50 | Treatment Protocol  Group 1:  Dexamethasone at 0.25 mg/(kg.d) and gamma globulin at 400 mg/(kg.d)  Received intravenous injection of prednisone at 1 mg/(kg.d) 3 days later  Group 2:  Dexamethasone at 0.25 mg/(kg.d) and gamma globulin at 400 mg/(kg.d)  Children in both groups were treated continu-ously for 1 month/course | Comparison of clinical manifestations between two groups  Average renal involvement time   - After treatment: 4.03+/-0.67   Urinary protein excretion   - After treatment: 347.35+/-132.43 mg/d   Serum B2 microglobulin   - After treatment: 0.23+/-0.07 mg/L   All mean values dramatically better than those in the control group (P<.05)  Comparison of therapeutic effects between the 2 groups of children treated by different treatment methods   - Total effective rate in group 1 was markedly higher than that in the control group (P<.05)   Comparison of immune function between the two groups before and after treatment  Immune function in indexes of CD3+, CD4+, CD8+, CD4+/CD8+, IgA   - Before treatment: No significant differences between 2 groups - After treatment: levels of CD3+, CD4+, CD8+ increased; CD4+/CD8+ and IgA decreased   Extent of change in group 1 was more obvious than that in control group  Changes of serum inflammatory factors in two groups before and after treatment   - Before treatment: No marked differences were noticed in serum inflammatory factors levels represented by IL-8, IL-10, and TNF-α between the two groups (P > .05). - After treatment: the levels of IL-8 and TNF-α in the two groups decreased, while IL-10 level increased   Change in the research group was more significant than that in the control group (P < .05)  Changes of coagulation function in the two groups before and after treatment   - Before treatment: no significant differences in coagulation function indexes between the two groups (P > .05). - After treatment: FIB decreased while PT and APTT increased in the two groups, with a larger change extent in the research group (P < .05).   Comparison of urine routine indexes between the two groups before and after treatment   - Before treatment: urine protein and urine red blood cell routine indexes did not identify any marked difference between the two groups (P > .05). - After treatment: urine protein and urine red blood cell routine indexes dropped in both groups   Change in the research group was more significant than that in the control group (P < .05).  Comparison of renal function indexes between the two groups before and after treatment   - Renal function indexes of Scr and BUN did not differ significantly between the two groups - After treatment: Scr and BUN in both groups decreased   Change in the research group was more obvious than that in the control group (P < .05). |
| Aslan, C., Goknar, N.,2022,  Turkey | Long-term Results in Children with Henoch-Schönlein Nephritis  Medeni Med J 2022; 37:159-164 | Retrospective  Objective: To investigate the relationship of laboratory, clinical, and histopathological findings with the long-term prognosis in children who were diagnosed with HSP nephritis.  Inclusion criteria:   1. Diagnosed with HSPN 2. Presented to Istanbul Medeniyet University between January 2010 and January 2019   Exclusion criteria:  n/a  Study groups:  Group 1: Mild  Group 2: Severe  According to the clinical and laboratory findings at the presentation, the patients were divided into five grades:   - Grade I: defined as isolated hematuria (macroscopic or microscopic) - Grade II: hematuria and non- nephrotic-range proteinuria (4-40 mg/m2/h) - Grade III: nephritic syndrome (hematuria, reduced GFR, oliguria, hypertension, and edema) - Grade IV: nephrotic syndrome (>40 mg/m2/h proteinuria, hypoalbuminemia, hyperlipidemia, and edema) - Grade V: nephritic/ nephrotic syndrome.   According to this grading, grades I and II were classified as mild groups and grades III, IV, and V were classified as severe groups.  Outcomes:   1. Evaluation of renal involvement 2. Joint findings 3. GI findings 4. Kidney biopsy 5. Most common histopathological grade 6. Treatment 7. Division of patients according to chronic phase meadow grading | Outcome, Immunosuppression, Angiotensin-converting enzyme inhibitor, Vasculitis, IgA glomerulonephritis, Nephrotic syndrome, Children | N= 90 analyzed  Group 1 (mild):  n= n/a  Group 2 (severe):  n= n/a  Mean age:  All:  8.8 (SD ± 3.2)  Sex (M/F):  All: 59/31 | Treatment Protocol  n/a | Evaluation of renal involvement findings in the acute phase using the Meadow acute phase classification   - Grade I: 46 (51.1%) patients - Grade II: 26 (28.9%) patients - Grade III: 4 (4.4%) patients - Grade IV: 9 (10%) patients - Grade V: 5 (5.6%) patients   Joint findings   - Statistically significantly higher rate in the severe group (72%) compared to mild group (43%) (p=0.027)   GI findings   - No significant difference found between the 2 groups (p=0.596)   Kidney biopsy   - Performed in acute phase in 33 patients (21 males and 12 females)   Most common histopathological grade   - ISKDC 3B   Of the 33 patients who underwent biopsy, 14 (42%) patients had grade 3B findings.  According to the acute phase Meadow findings, grade 3B findings were found in only 4 (25%) patients in the mild group, while pathological findings of grade 3 and above were found in 12 (75%) patients in the severe group.  The histopathological grades of the patients who had severe clinical findings according to the Meadow classification in the acute phase were statistically significantly more severe compared to the mild group (p=0.022).  Treatment  Immunosuppressive treatment   - Mild group: 32 (58%) patients - Severe group: 18 (100%) patients   ACEI/ARB Therapy   - Simultaneously initiated in all patients with proteinuria   Treatment distribution shown in table 3  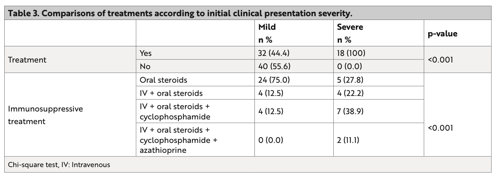  Division of Patients According to Chronic Phase Meadow Grading   - 86 (95.6%) patients were classified as group A - 4 (4.4%) as group B (Table 4).   No patients who had compatible findings with groups C and D in the chronic phase.  Two of our patients who had grade 4B and 6 findings indicating severe histopathological involvement continue to have non-nephrotic-range proteinuria.  Our other two patients were surprisingly considered grade 3A and 2B in the acute phase (Table 5).  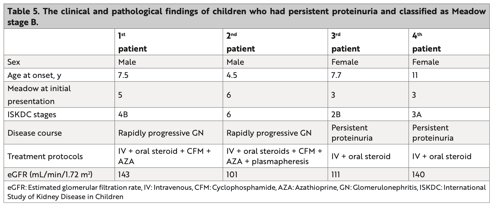 |
| Feng, D., Huang, W.Y., 2017,  China | A single-center analysis of Henoch-Schonlein purpura nephritis with nephrotic proteinuria in children  Feng et al. Pediatric Rheumatology (2017) 15:15 DOI 10.1186/s12969-017-0146-4 | Retrospective  Objective: to assess the clinical and pathological features of HSPN with nephrotic proteinuria in a single center.  Inclusion criteria:   1. Children diagnosed with HSPN 2. Admitted to Shanghai Children’s Hospital between 2009 and 2013   Exclusion criteria:  n/a  Study groups:  Group 1: Nephrotic proteinuria group (NP)   - 24-h urinary protein ≥50 mg/kg   Group 2: Non-nephrotic proteinuria group (NNP)   - 24-h urinary protein <50 mg/kg   Outcomes:   1. Clinical features 2. Laboratory examination 3. Immunologic analysis 4. Pathological features 5. Immune complex deposition 6. Tubulointerstitial injuries 7. Nephrotic-range proteinuria in HSPN prognostic analysis 8. Correlation analysis of the clinical classification and prognosis 9. Pathological grading and prognosis analysis 10. Tubulointerstitial injuries and prognosis analysis   Treatment and prognosis analysis | Outcome, Immunosuppression, Angiotensin-converting enzyme inhibitor, Vasculitis, IgA glomerulonephritis, Nephrotic syndrome, Children | N= 137 analyzed  Group 1 (NP):  n=54/54 analyzed  Group 2 (NNP):  n=83/83 analyzed  Mean age:  Group 1: 8.39 (SD ± 2.85)  Group 2: 9.21 (SD ± 2.94)  Sex (M/F):  Group 1: 34/20  Group 2: 46/37 | n/a | Clinical Features  Joint Symptoms   - Group 1: 8 (14.81%) - Group 2: 9 (16.67%)   GI Symptoms   - Group 1: 10 cases (12.04%) - Group 2: 12 cases (14.46%)   Compared with the NNP group, the NP group had more patients with joint and gastrointestinal symptoms.  Laboratory examination  Summarized in table 1  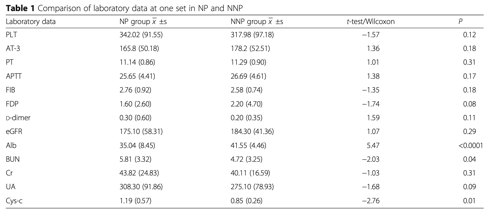  Immunologic analysis  Abnormalities Type   - Humoral and cellular immunities abnormalities   Level of blood IgG of the NP group   - Lower than that if NNP group (P<0.05)   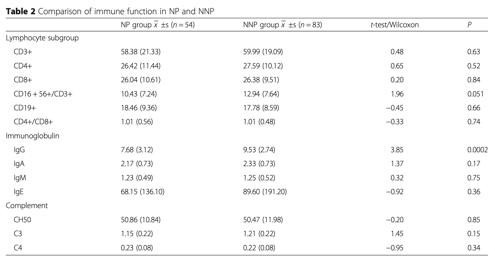  Pathological features  ISKDC Grading Score   - Grade I: 35 patients - Grade II: 36 patients - Grade IIIa: 48 - Grade IIIb: 11 - Above grade IV: 6   NP Group:   - Grade I: 5 (9.26%) patients - Grade II: 15 (27.78%) patients - Above grade IIIa: 34 (62.97%)   NNP Group:   - Grade I: most patients (37.35%) - Grade IIIa: 32.53%   Immune complex deposition  NP Group:   - IgA deposits: 17 cases - IgA + IgM deposits: 15 - IgA + IgM + IgG deposits: 14   NNP Group:   - Most patients had IgA deposits but followed by IgA + IgM + IgG deposits   Tubulointerstitial injuries by grade   - Grade 1: 91 (66.42%) patients - Grade 2: 37 (27.01%) patients - Grade 3: 8 (5.84%) - Grade 4: 1 (0.73%)   The difference in tubulointerstitial injuries between the two groups had a statistical significance (P < 0.01)  Nephrotic-range proteinuria in HSPN prognostic analysis   - 68.52% had a good outcome (A) - 27.78% had mild urinary anomalies (B) - only 2 had a remained active renal disease (C) - no patient had an ESRD progression (D).   Correlation analysis of the clinical classification and prognosis   - 37 patients (68.52%) with a complete remission (A) - only 2 patients (3.7%) had an active renal dis- ease (C). - clinical manifestations in 6 patients with nephrotic syndrome (11.11%) and mild urinary anomalies (B). - One case of radical nephritis developed into minor urinary abnormalities (B). - One case of rapidly progressive glomerulonephritis developed into active renal disease (C).   As shown in Table 7, the different clinical classifications of the prognostic difference in the NP group had a statistical significance (P < 0.01).  The more severe the clinical manifestations were, the worse the prognosis was.  Pathological grading and prognosis analysis  NP group:   - all patients with grade I had a complete remission (A) - 8 (14.81%) with grade II showed a complete remission (A) - 6 (11.11%) had mild urinary anomalies (B) - only 1 (1.85%) showed an active renal disease (C). - Twenty-five patients (46.29%) with grades III–VI had good outcomes (A), 8 (14.82%) had mild urinary anomalies, and only 1 (1.85%) showed an active renal disease (C).   No correlation existed between the pathological grading and prognosis (Table 8).  Tubulointerstitial injuries and prognosis analysis  Of 54 patients:   - 66.67% with grade 1 had a complete remission (A) - 8 (29.62%) had a poor prognosis for mild urinary anomalies (B) - 13 (65.00%) with grade 2 showed a complete remission (A) - 7 (35.00%) had a poor prognosis for mild urinary anomalies (B) - Six patients (85.17%) with grades III–IV had good outcomes (A). Only 1 patient (1.85%) showed an active renal disease (C). No correlation existed between the tubulointerstitial injuries and prognosis (P > 0.05).   Treatment and prognosis analysis  The therapies of HSPN include:   - steroids, immunosuppressive agents, angiotensin converting enzyme inhibitors (ACE-I) and/or angiotensin receptor blockers (ARB), plasmapheresis, and tonsillectomy.   Main treatment in the NP group: steroids combined with mycophenolate mofetil (46.30%).  The secondary treatment was methylprednisolone pulse therapy (20–30 mg/kg/d, maximal dose 1 g) for 3 days; steroids combined with mycophenolate mofetil (25.93%) were used thereafter.  Thirty-eight patients (70.37%) had a complete remission (A). Only 2 patients (3.70%) showed an active renal disease (C).  Prognosis of the NP group improved after a timely and early treatment.  therapies had no obvious adverse effects |
| Anderson, R.F., Subak, S., 2009,  Denmark | Early high-dose immunosuppression in Henoch–Schönlein nephrotic syndrome may improve outcome  R. F. Andersen, S. Rubak, B. Jespersen & S. Rittig (2009) Early high-dose immunosuppression in Henoch–Schönlein nephrotic syndrome may improve outcome, Scandinavian Journal of Urology and Nephrology, 43:5, 409-415, DOI: 10.3109/00365590903164480 | Retrospective  Objective: To report the prevalence of severe renal complications to HSP and the clinical course and long-term outcome of six patients with HSP and nephrotic range proteinuria treated at Aarhus University Hospital from 2000 to 2007.  Inclusion criteria:   1. Age below 16 years at presentation 2. Diagnostic criteria of HSP and nephrotic range proteinuria (defined as proteinuria >40 mg/m2/h. Nephrotic syndrome was defined as proteinuria􏰃40 mg/m2/h, oedemas and a plasma albumin levelB25 g/l)   HSP was defined as palpable purpura and at least one of the following symptoms: diffuse abdominal pain, arthritis or arthralgia, renal involvement, e.g. haematuria and/or proteinuria, and any biopsy-proven immuno- globulin A (IgA) deposition [16].  Exclusion criteria:  n/a  Study groups:  No groups  Outcomes:   1. Basic characteristics 2. Nephrotic-range proteinuria 3. Hypertension 4. Treatment 5. Histological characteristics 6. Proteinuria 7. Outcome   Outcome data were obtained at the date of the last contact during the study period.  Long-term out- comes were classified by Meadow:  (A) Normal. No renal or urinary abnormalities. No hypertension.  (B) Persistent urinary abnormalities. Low-grade proteinuria (B40 mg/m2/h) or microscopic haematuria. Normal plasma creatinine and GFR. No hypertension.  (C) Active renal disease. Proteinuria >40 mg/ m2/h, hypertension/need for antihypertensive therapy or GFR below 75 ml/min/1.73 m  (D) ESRD. Need for dialysis and renal replacement therapy. | ESRD, Henoch-Schoenlein purpura, immunosuppressive therapy, nephrotic syndrome, tacrolimus | N= 6 analyzed  Mean age at HSP presentation:  13.2 (6.3-15.8 years)  Sex (M/F):  4/2 | The protocol of immunosuppressive treatment at this centre included as first line therapy oral prednisolone 60 mg/m2/day (maximum 80 mg/day) for 6 weeks followed by 6 weeks with alternate-day prednisolone 40 mg/m2 and then a slowly tapering dose of alternate-day prednisolone (5-10 mg) for several months.  Second line immunosuppressants were used in patients without significant reduction in proteinuria during the first 4 weeks of prednisolone therapy, including: (1) cyclophosphamide (2 mg/kg/day for 12 weeks), (2) CyA (initially 5 mg/kg/day, C2 blood level kept at 600-800 mg/l), (3) mycophenolate mofetile (25 mg/kg/day), and (4) tacrolimus (initially 0.1 mg/kg/day, blood level kept at 5-10 mg/l)). Second line modalities were initially administered in combination with high-dose prednisolone that was tapered to 5-10 mg on alternate days after a period.  All patients were treated with the ACE inhibitor ramipril (2.5-5 mg/day) during periods with significant proteinuria and/or elevated blood pressure. | Basic characteristics  Summarized in table 1  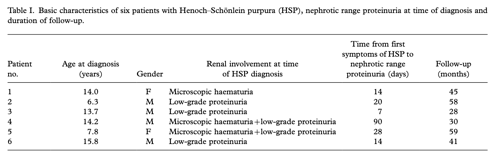  Nephrotic-range proteinuria   - Median: 277 mg/m2/ h (65-640 mg/m2/h)   Hypertension   - Seen in 3 patients at initial presentation   Treatment   - Prednisolone: all patients received before first renal biopsy but no other immunosuppressants   First line immunosuppressive therapy with prednisolone   - Initiated in 5 patients - 3 patients (2, 3, 4) responded to this protocol and proteinuria was reduced below 40mg/m^2^/h - Patients 5 and 6 did not reduce proteinuria within 4 weeks of high-dose prednisolone therapy and cyclophosphamide was added in combination with prednisolone   Histological characteristics (according ISDKC classification)   - Grade IIIa: 3 patients - Grade IIIb: 2 patients - Grade IVb: 1 patients   Proteinuria   - Follow-up period: all patients developed proteinuria   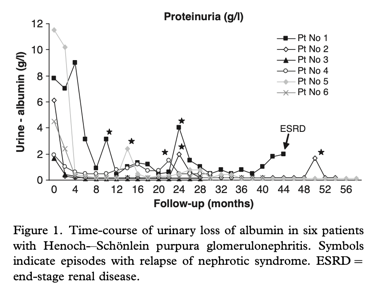  Outcome  The five patients receiving early high-dose immunosuppression according to the study protocol had preserved kidney function at follow-up, while the patient receiving low-doses of immunosuppressant therapy progressed to ESRD during follow-up.  Table III summarizes long-term outcome as purposed by Meadow in relation to initial characteristics and ISKDC biopsy classification  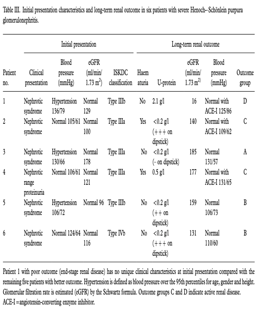 |
| Hisano, S., Hatae, K.,  1989,  Japan | High-dose methylprednisolone therapy for nephrotic syndrome in Henoch-Schoenlein nephritis  Japanese Journal of Nephrology Vol. 31, No. 10, 1989 | Retrospective  Objective: to describe the effectiveness of high-dose intravenous MP in the treatment of patients with NS in HS nephritis based on our own clinical experience  Inclusion criteria:   1. Patients with NS in HS nephritis 2. Aged 4-14 3. All patients had both hematuria and proteinuria associated with characteristic manifestations   NS was defined based on urinary protein >40 mg/m2/hr and hypoalbuminemia < 2.5g/dl.  Acute nephritic syndrome was defined as hematuria associated with at least two of the following features: (a) hypertension, i.e., blood pressure >95th percentile for age from the Task Force on Blood Pressure Control in Children [10], (b) oliguria, and (c) endogenous creatinine clearance (Ccr) < 60 ml/min/1.73 m2.  Exclusion criteria:  n/a  Study groups:  No groups  Outcomes:   1. Clinical outcome 2. Relationship between renal pathology and clinical outcome 3. Side effects | methylprednisolone, nephrotic syndrome, Henoch-Schoenlein nephritis, renal failure | N= 12 analyzed  No groups  Mean age:  8.3 (range: 4-14 years)  Sex (M/F):  10/2 | Treatment Protocol  Soon after a definite diagnosis of NS was made, all patients were given a dose of 20 mg/kg (maximum 1g) of intravenous MP over a one-hour period.  Such intravenous MP was administered on each of nine alternate days. After completion of this course of MP therapy, oral prednisolone of 1 mg/kg (maximum 40 mg) was given daily in a single morning dose to 8 patients for a period of 2 to 4 weeks because of proteinuria > 1 g/day.  Then, a dose of this regimen of 2 mg/kg (maximum 60 mg) on alternate days was given to these 8 patients for 4 weeks.  Subsequently, the prednisolone was withdrawn through a stepwise reduction at a dosage of 10mg on alternate days at 4-week intervals for 4 months.  After completion of this course of MP therapy, the remaining 4 children received a dose of 2 mg/kg (maximum 60 mg) of oral prednisolone on alternate days for 4 weeks because of proteinuria < 1 g/day, and the prednisolone was then reduced and withdrawn at 4-week intervals for 4 months in the same manner as described above.  No immunosuppressive drugs were given to any patient throughout the follow-up period. | Clinical outcome  Acute nephritic syndrome at onset of NS   - 5/12 patients - Displayed in table 2   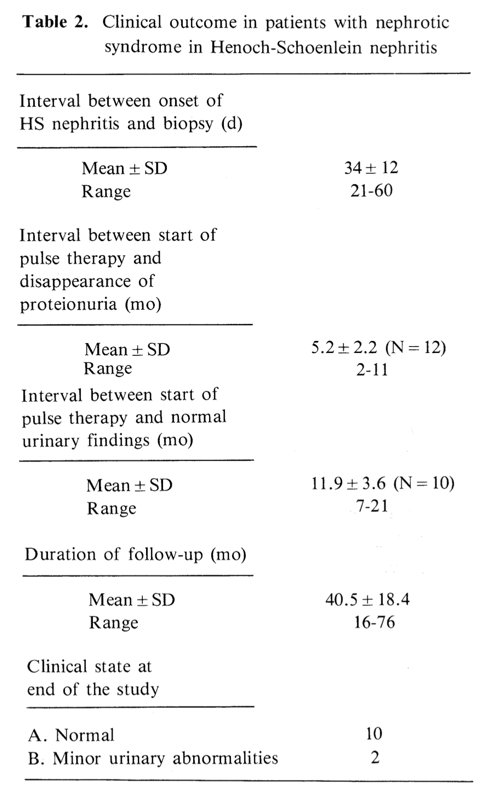  Renal insufficiency   - 4 patients (1, 2, 9, and 10)   Hypertension   - 1 patient (patient 3)   Renal insufficiency and hypertension shown in table 1  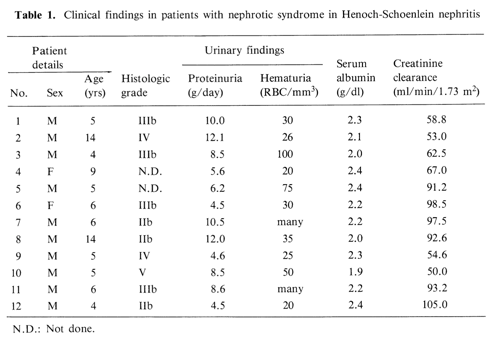  Biopsy   - Performed on 10/12 patients at mean interval of 34 days after onset of HSN   Disappearance of urine protein   - Observed in all the 12 patients at a mean interval of 5.2 months after start of MP therapy   Urinary findings   - 10/12 patients presented (normalized at mean interval of 11.9 months)   Relationship between renal pathology and clinical outcome  Shown in table 3  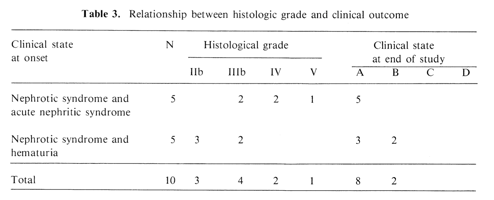  Results of the 5 patients with combination of NS and hematuria at onset   - Grade IIb: 3/5 patients - Grade IIIb: 2/5 patients - State A: 3/5 patients   Results of the 5 patients with a combination of NS and hematuria at onset   - Grade IIb: 3/5 patients - Grade IIIb: 2/5 patients - State A: 3/5 patients - State B: 2/5 patients   IgA deposition   - Mesangium and along adjacent extending capillary walls: 10 patients   IgG deposition along capillary walls   - 6 patients   C3 deposition along capillary walls   - 7 patients   C4 deposition along capillary walls   - 3 patients   Fibrinogen deposition along capillary and extra capillary walls   - 8 patients   Severity of deposition of Ig’s, complements, and fibrinogen   - No differences noted between patients with and those without acute nephritic syndrome   Results of follow-up biopsies   - Minor glomerular abnormalities at 2 years after initial biopsy: 1 patient with grade IV - In a patient with grade V, LM revealed diffuse mesangial proliferation and segmental glomerular sclerosis accompanied by cellular and fibro cellular deposits in 14/17 glomeruli at initial biopsy (figure 1a)   However, LM demonstrated fibrocellular crescents in 4 of 13 glomeruli, and improvement of the mesangial proliferation and segmental glomerular sclerosis at 6 months after the initial biopsy (Fig. lb).  The tubular atrophy and interstitial fibrosis disappeared. IgA and fibrinogen deposition became weaker than at the initial biopsy on IF, and IgG and C3 deposition along the capillary walls disappeared.  Note: The 2 above patients were in state A at end of study  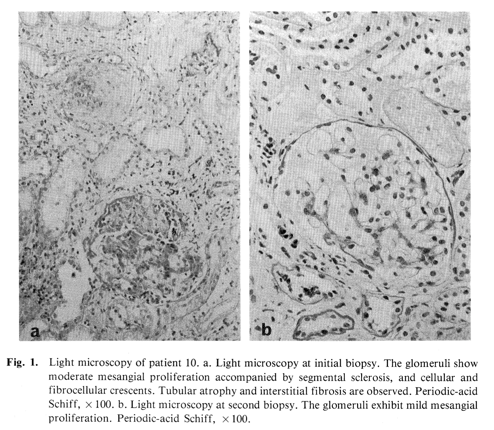  Side effects of high-dose MP followed by oral prednisolone   - Obesity: one patient - Stria: one patient - Hypertension: one patient   Cataract: one patient |
| Oner, A., Tinaztepe, K., 1995,  Turkey | The effect of triple therapy on rapidly progressive type of Henoch-Schonlein nephritis  Pediatr Nephrol (1995) 9: 6-10 | Prospective  Objective: to evaluate the clinical and laboratory effect of triple therapy consisting of corticosteroids, cyclophosphamide, and dipyridamole on RPGN in HSR and to derive prognostic factors and clinicopathological correlation    Inclusion criteria:   1. Children with HSN 2. Had rapidly progressive clinical course   Exclusion criteria:  n/a  Study groups:  No groups  Outcomes:   1. Clinical findings 2. Histopathological findings 3. Treatment and outcome 4. Clinicopathological correlations | Henoch-Sch6nleinnephritis, Rapidly progressive glomerulonephritis, Triple therapy, Prognosis | N= 12 analyzed  No groups  Mean age:  10.3 (range: 6-14 years)  Sex (M/F):  7/5 | n/a | Clinical findings  Summarized in table 1  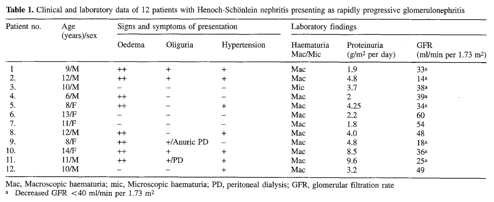  Histopathological findings  Percutaneous renal biopsy   - Performed in 10 patents between 2-8 weeks after disease onset   Results of 9 biopsies   - Glomeruli affected by extensive crescent formation: 60-90% 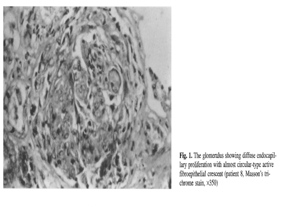(figure 1) - Epithelial and fibroepithelial crescents: predominant in 8 - Fibrous crescents were predominant: predominant in 2 - Tubular atrophy and interstitial fibrosis: prominent in 4 patients (nos. 2, 6, 11, 12)   Results of fluorescence microscopy   - Available in 8 renal biopsies - Prominent IgA deposition in dermal capillaries: 1 patient - Diffuse granular mainly mesangial IgA deposition: present in all glomeruli of all renal biopsies - C3, IgG, IgM, and fibrinogen deposition: demonstrated in association with IgA fluorescence   Treatment and outcome  Received Triple therapy   - 9 patients with severe glomerulonephritis and 2 patients who fulfilled the clinical and lab criteria of RPGN   Interval between disease onset and initiation of therapy   - Varied from 2-6 weeks: 10 patients - Exceeded 2 months: 2 patients (nos. 2 and 11)   Treatment protocol  30mg/kg per day intravenous pulse for 3 consecutive days, followed by oral prednisolone at a dosage of 45 mg/m^2^ per day tapered over 3 months, 2 mg/kg per day cyclophosphamide for 2 months and 5 mg/kg/d dipyridamole for 6 months  Results at end of triple therapy   - GFR: returned to normal values (80-95 ml/min per 1.73 m2, mean 86.3+/-4.5) in all except patient 2 (GFR 35 ml/min per 1.73m2)   Proteinuria   - Disappeared in 8 patients 2-20 months from onset   Remission   - Complete remission: 7 (58%) patients (had no relapses) - Partial remission: 3 patients with persistence of proteinuria and hematuria   Clinicopathological correlations  Outcome vs renal histopathology   - Shown in table 2   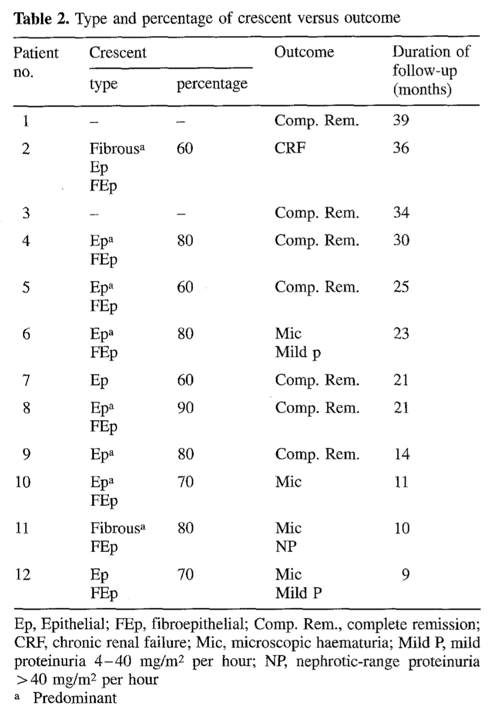  Patients with epithelial and fibroepithelial crescents   - Considerably better prognosis than those with fibrous crescents (patients 2 and 11)   Renal outcome   - No directly related to extent of crescent formation   Recovery   - Patients with >90% crescent formation: recovered completely - Patients with 60-80 crescent formation (patients 2 and 11): had worse clinical course   Severity of tubular atrophy and interstitial fibrosis   - Related to duration of the disease and timing of renal biopsy |
| Mizerska-Wasiak, M., Maldyk, J.,  2016,  Poland  Abstract only | Treatment Outcomes in Children with Henoch-Schönlein Nephritis Mizerska-Wasiak, M., Małdyk, J., Demkow, U., Roszkowska-Blaim, M., Pańczyk-Tomaszewska, M. (2016). Treatment Outcomes in Children with Henoch-Schönlein Nephritis. In: Pokorski, M. (eds) Prospect in Pediatric Diseases Medicine. Advances in Experimental Medicine and Biology(), vol 912. Springer, Cham. P. 65-72. https://doi.org/10.1007/5584_2016_231 | Objective: to quantify the effects of treatment of children with Henoch-Schönlein nephritis (HSN) evaluated on the basis of kidney biopsy findings  Inclusion criteria:   1. Patients diagnosed with HSN as confirmed by renal biopsy   Exclusion criteria:  n/a  Study groups:  No groups  Outcomes:   1. Proteinuria 2. Histological improvement | n/a | N= 32 analyzed   - 19 with nephrotic syndrome/nephrotic proteinuria NS/NP - 13 with nephritic syndrome NphS   No groups  Mean age:  9.3 (SD +/- 3.5 years)  Sex (M/F):  n/a | Treatment  Patients received immunosuppressive treatment (azathioprine or cyclophosphamide) and/or steroids and renoprotection according to a defined protocol.  Patients were referred to a specific treatment protocol selected based on clinical symptoms of nephropathy (NS/NP or NphS) and histopathological grade according to the WHO classification:   - Grade I–II changes were defined as mild HSN, and grade III–V WHO as severe HSN. | Proteinuria   - Following treatment, proteinuria resolved in 78 % children with mild HSN and 87 % children with severe HSN.   Histological Improvement   - In kidney biopsy, histological improvement was seen in 59 % children and no worsening in 35 %. |
| Kawasaki, Y., Suzuki, J., 2004,  Japan  Abstract only | Efficacy of methylprednisolone and urokinase pulse therapy combined with or without cyclophosphamide in severe Henoch-Schoenlein nephritis: a clinical and histopathological studyNephrol Dial Transplant. 2004 Apr; 19(4): 858-64. | Retrospective  Objective: to evaluate the efficacy of methylprednisolone and urokinase combined with cyclophosphamide for patients with HSPN of at least grade IVb  Inclusion criteria:   1. Patients diagnosed with HSPN of at least grade IVb   Exclusion criteria:  n/a  Study groups:  Group A: methylprednisolone and urokinase pulse therapy (MP+Uro)  Group B: methylprednisolone and urokinase pulse therapy combined with cyclophosphamide (Combined)  Outcomes:   1. Clinical features 2. Laboratory and pathological findings | n/a | N= 37 analyzed  Follow-up:  Group A: 24 months  Group B: 24 months  Group A (MP+Uro):  n=20/20 analyzed  Group B (Combined):  n=17/17 analyzed  Mean age:  Group A:  8.0 (SD +/- 2.8 years)  Group B: 7.1 (SD +/- 3.1 years)  Sex (M/F):  Group A: 9/11  Group B: 8/9 | Treatment protocol  n/a | Clinical & Laboratory Features  Mean urinary protein excretion (after 6 months treatment):   - Group B: significantly decreased compared with Group A   Proteinuria (mg/h/m^2^)   - Group A: 154+/-73 - Group B: 181+/-85   eGFR (ml/min/1.73m^2^)   - n/a   Serum albumin (g/L)   - Group A: 29+/-4 - Group B: 27+/-6   Pathological Findings   - Activity index of both groups at the second biopsy was lower than that at the first. - At the second biopsy, the chronicity index of Group B was lower than that of Group A. - Four patients of Group A but none of Group B had persistent nephropathy (P<0.05). |

**Table S8. IgA Vasculitis Nephritis Observational Studies**

| 1st , 2nd Author, Year, Country of Origin | Title of Publication and citation | Study Design and setting | Keywords | Participants | Intervention and Comparator | Outcomes |
| --- | --- | --- | --- | --- | --- | --- |
| Shin, J.I., Park, M.,  2006,  Korea | Predictive factors for nephritis, relapse, and significant proteinuria in childhood Henoch–Schönlein purpura  Shin, J.I., Park, M., Predictive factors for nephritis, relapse, and significant proteinuria in childhood Henoch–Schönlein purpura. Scand J Rheaumatol, 2006. 35: p. 56-60. | Retrospective non-randomized trial  Single centre  Objective: To identify predictive factors for nephritis, relapse, and significant proteinuria in childhood Henoch– Scho ̈nlein purpura (HSP)  Inclusion criteria:   1. Diagnosis of HSP (According to the criteria of Michel et al [reference 10]) 2. Age at onset: 20 years or younger 3. No medication   Exclusion criteria:   1. Less than 1 year follow-up 2. Those referred from other hospitals   Study groups:  Group 1: Prednisolone, mizoribine, dipyridamole, ACEI  Group 2: Prednisolone, dipyridamole, cyclophosphamide  Outcome measures:  During the follow-up the patients were regularly monitored for clinical and laboratory parameters for renal sequelae and relapse. | N/a | N= 206 analyzed  Duration of follow-up was 3.1 ± 2.2 years  No groups  Mean age at onset:  7.2 (SD ± 2.8 yrs)  Sex (M/F):  113/93 | n/a | Univariate Analysis of Predictive Factors  Significant Factor for Nephritis & Significant Proteinuria:   1. Age of more than 10 years   Persistent purpura, severe bowel angina, relapse   1. More common in patients with nephritis   Significant proteinuria:   1. More common in patients without nephritis   Prevalence of arthritis:   1. Lower in patients with nephritis   p=0.003  Gastrointestinal bleeding   1. Not different in patients with or without nephritis 2. Closely associated with severe bowel angina   p=0.002  Serum IgA mean levels:   1. Not different in patients with or without nephritis   Relapse-related factors:   1. Age of 10+ years at onset 2. Persistent purpura 3. Severe bowel angina 4. Leucocytosis   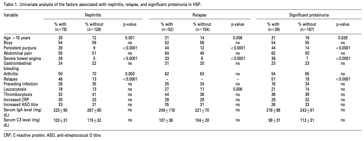  Factors related to development of hypertension:   1. Age of 10+ years 2. Severe bowel angina 3. Gastrointestinal bleeding   Multivariate Analysis of Predictive Factors  Factors associated with nephritis:   1. Age of more than 10 years 2. Persistent purpura 3. relapse   Factors associated with relapse:   1. Age of more than 10 years 2. Persistent purpura 3. Severe bowel angina   Predictive factors of significant proteinuria:   1. Severe bowel angina 2. Relapse   Independent risk factors of hypertension:   1. Age of more than 10 years 2. Gastrointestinal bleeding   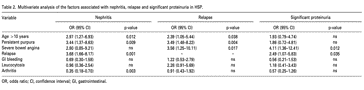  Predictive model for nephritis and significant proteinuria Predictive models were designed for nephritis and significant proteinuria based on the variables with a p-value lower than 0.05  Variables in these models included an age of more than 10 years at onset, persistent purpura, severe bowel angina, and relapse  The model gave one point to each one of these four variables associated with nephritis and significant proteinuria.  The risk of nephritis or significant proteinuria increased on increasing the sum of scores composed of these predictive factors (Figure 1).  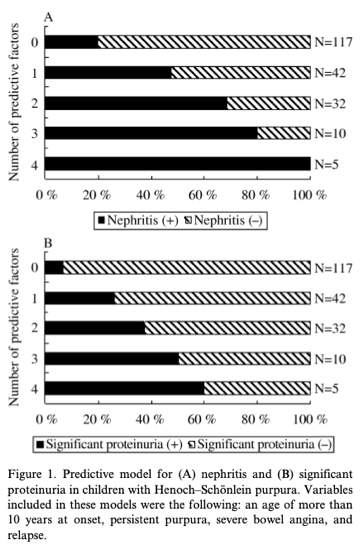  Adverse events:  n/a |
| Rigante, D., Candelli, M.,  2005,  Italy | Predictive factors of renal involvement or relapsing disease in children with Henoch-Scho ̈nlein purpura  Rigante, D., Candelli, M., Predictive factors of renal involvement or relapsing disease in children with Henoch-Scho ̈nlein purpura. Rheumatol Int, 2005. 25: p. 45-48 | Retrospective non-randomized trial  Objective: To evaluate the clinical and laboratory parameters in relation to HSP evolution, with particular attention to the occurrence of renal involvement and relapsing disease.  Inclusion criteria:   1. Diagnosis of HSP based on clinical/laboratory data   Exclusion criteria:  Twelve children that were submitted to steroid therapy at the onset of HSP because of gastrointestinal, renal, or neurological complications and another eight children with initial signs of renal disease were excluded from our statistical evaluation  Study groups:  n/a  Outcome measures:   1. Clinical and laboratory features of children with Henoch-Schonlein purpura   Probability of renal complications or relapse occurrence during follow-up | Child, Henoch-Scho ̈ nlein purpura, Relapse, Renal disease | N= 94 analyzed  Mean study duration was 6±3 years  No groups  Mean age at onset:  6.33 (SD ± 2.54 yrs)    Sex (M/F): 46/48 | n/a | 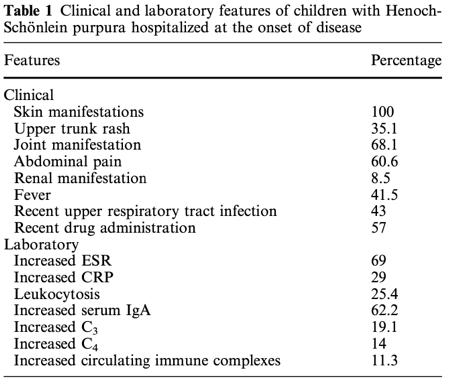Clinical Features of Children at the Onset of Disease  Follow-Up  Note: follow-up was completed in the group of 74 children examined statistically  Urinary signs:   1. Normalized completely in a week in children with renal involvement at onset appearing with isolated microhematuria and in those appearing with microhematuria associated with proteinuria after a period between 9 months and 5 years   Renal involvement presentation (%)   1. 18.3% of children (appeared between 1 month to 2 years after onset of HSP)   Presentation of one or more episodes of relapse (%):   1. 12.2% children (between 1 month and 5 years after the primary onset of HSP)   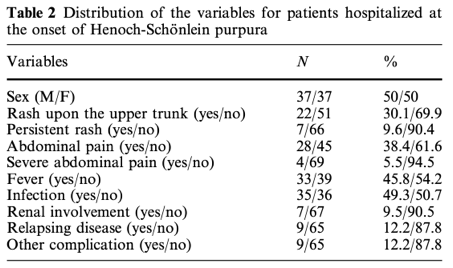Statistical Evaluation  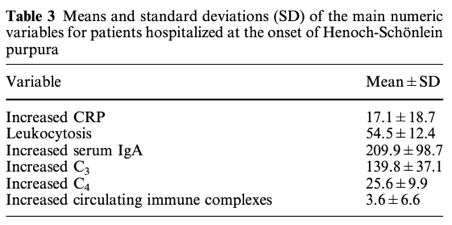  Factors significantly related with renal involvement (according to simple logistic regression):   1. Persistent rash 2. Severe abdominal pain   P=0.01  Factors significantly related with relapse (according to simple logistic regression):   1. Persistent rash   P=0.01  Factors related to renal involvement when adjusted for sex and age or sex, age, and severe abdominal pain (according to multiple logistic regression):   1. Persistent rash   Factors related to renal involvement when adjusted for sex (according to multiple logistic regression):   1. Severe abdominal pain (untreated to renal involvement when adjusted for sex, age, and persistent rash)   Factors related to possibility of relapse when adjusted for sex and age or for sex, age, and abdominal pain (according to multiple logistic regression)   1. Persistent rash   Adverse events:  1 person in Group 2 developed herpes zoster. |
| Elkici, R.M.K., Balci, S.,  2020,  Turkey | Clinical manifestations and outcomes of 420 children with Henoch Schonlein Purpura from a single referral center from Turkey: A three-year experience  Elkici, R.M.K., Balci, S., Clinical manifestations and outcomes of 420 children with Henoch Schonlein Purpura from a single referral center from Turkey: A three-year experience. MODERN RHEUMATOLOGY, 2020. 30(6): p. 1039-1046. | Retrospective review of medical records  Objective: To evaluate the clinical features, seasonal variation, treatment outcomes and the possible predicting factors related to outcome among a large cohort of pediatric HSP patients.  Inclusion criteria:   1. Diagnosis of HSP according to the Ankara 2008 criteria   Exclusion criteria:  n/a  Study groups:  n/a  Outcome measures:   1. Clinical characteristics 2. Laboratory work-up 3. Possible predicting factors related to clinical manifestations and outcomes   Further information on the diagnosis of HSP  Patients were diagnosed with HSP according to the Ankara 2008 criteria, which was verified by European League Against Rheumatism, Pediatric Rheumatology International Trials Organization, and Pediatric Rheumatology European Society (EULAR/ PRINTO/PRES)  In addition to the presence of palpable pur- pura as the mandatory criteria, one of the following items, including abdominal pain, arthralgia or arthritis, renal involvement, and predominant IgA deposition in any biopsy, is sufficient to classify a patient with HSP | n/a | N= 420 analyzed  Duration of follow-up was a median of 19.6 months (range: 6-30 months)  No groups  Mean age at diagnosis:  7.68 (SD ± 3.15 yrs)  Sex (M/F):  216/204 | n/a | Clinical Characteristics & Laboratory Work-Up  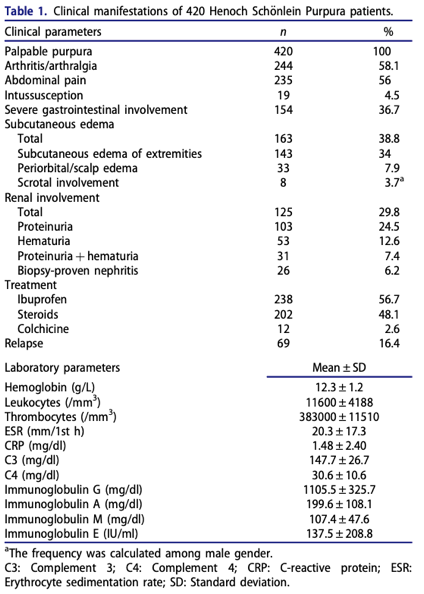Summarized by Table 1  Possible Predicting Factors Related to Clinical Manifestations and Outcomes  Effect of Seasons that HSP Occurred in on Clinical Manifestations   - Frequencies of subcutaneous edema, arthralgia and renal involvement did not differ between seasons - Severe GI involvement and intussusception rates were found higher in autumn and summer - Relapse rate was significantly more common in patients diagnosed in summer and least in spring - 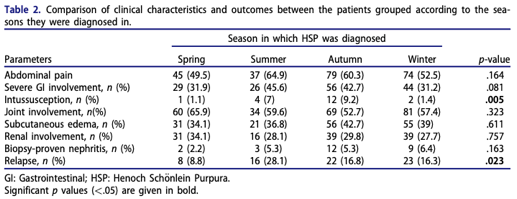Frequency of biopsy-proven nephritis was more common in patients that had HSP diagnosis during autumn than other seasons (difference was not statistically significant)   Comparison of Clinical Characteristics of Patients According to Presence of Severe Gastrointestinal System Involvement  2 groups created: those with severe GI involvement (group 1) & those without (group 2)  Gender, age at disease onset, periorbital/scalp edema and relapse rate:   1. Similar among both groups   Subcutaneous edema (particularly on extremities), arthritis/arthralgia:   1. Group 1: significantly less frequent than group 2   Frequency of Renal Involvement and Biopsy-Proven Nephritis:   1. Group 1: higher than in group 2   Leukocyte count:   1. Group 1: significantly higher than in group 2   Serum IgG and IgM Levels:   1. Group 1: lower than in group 2   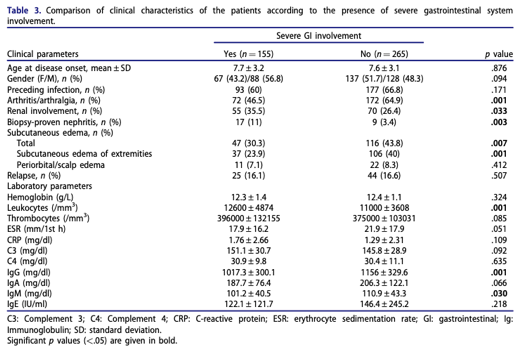  Comparison of Clinical Characteristics of Patients According to Presence of Biopsy-Proven Nephritis  2 groups created: those with biopsy-proven nephritis (group 1) & those without (group 2)  Age at disease onset, gender, overall subcutaneous edema, edema on extremities, and relapse rate:   1. Similar among both groups   Frequencies of preceding infection and joint involvement:   1. Group 1: lower than in group 2   Periorbital/scalp edema:   1. Group 1: higher than in group 2 (not statistically significant)   Rates of abdominal pain, intussusception, severe GI involvement, and systemic steroid administration:   1. Group 1: higher than in group 2   Hemoglobin, Serum IgG and IgM Levels:   1. Group 1: lower than in group 2   Leukocyte count and CRP levels   1. Group 1: higher than in group 2   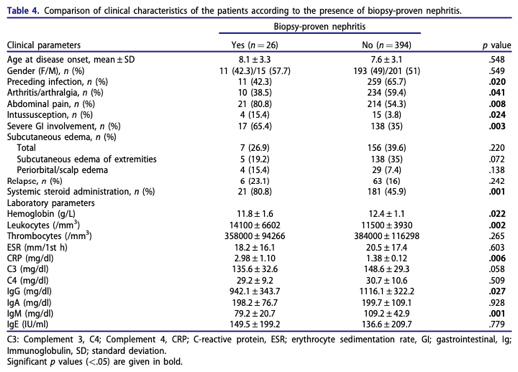  Comparison of Clinical Characteristics of Patients According to Having Relapse  2 groups created: those with relapse (group 1) & those without (group 2)  Mean age at disease onset:   1. Group 1: significantly higher than in group 2   Joint involvement and subcutaneous edema:   1. Group 1: found less frequent than in group 2   Intussusception rate:   1. Group 1: higher than in group 2 (not statistically significant)   Remaining disease characteristics and laboratory parameters:   1. Did not differ between these 2 groups   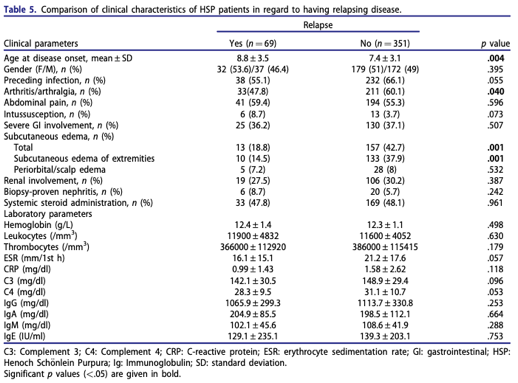  Adverse events:  n/a |
| Karadag, S.G., Tanatar, A.,  2019,  Turkey | The clinical spectrum of Henoch–Schönlein purpura in children: a single-center study  Karadag, S.G., Tanatar, A., The clinical spectrum of Henoch–Schönlein purpura in children: a single-center study. Clinical Rheumatology, 2019. 38: p. 1707-1714. | Prospective study  Objective: To evaluate the demographic and clinic findings of patients with HSP and also to determine predictive factors for assessing the development of gastrointestinal system (GIS) and renal involvement.  Inclusion criteria:   1. Diagnosis of HSP 2. Age of <18 years 3. Patients followed up in the Pediatric Rheumatology Unit of Health Sciences University Kanuni Sultan Suleyman Training and Research Hospital between January 2016 and January 2018   Exclusion criteria:  n/a  Study groups:  n/a  Variables analyzed:   1. Clinical characteristics 2. Laboratory work-up 3. Common disease onset seasons 4. Predisposing factors 5. Treatment 6. Intussusception 7. Nephrotic proteinuria and performance of renal biopsy procedure 8. Fever, subcutaneous edema, and scrotal involvement 9. MEFV mutation analysis   Definition of Relapse  Relapse was described as presence of a new disease-related symptom after an asymptomatic period of at least 3 months | n/a | N= 265 analyzed  Duration of follow-up was a median of 1.3+/-0.7 years (range: 0.5-2.5 years)  No groups  Mean age at diagnosis:  7.5 (SD ± 3.2 yrs)  Sex (M/F):  137/128 | n/a | Clinical Characteristics & Laboratory Work-Up  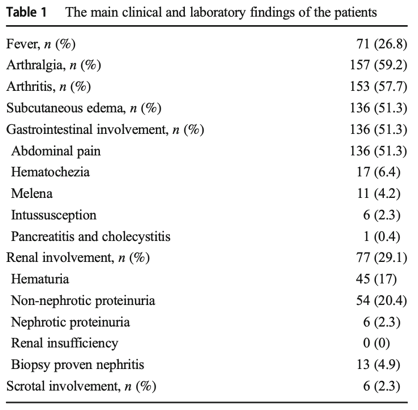Summarized by Table 1  Common Disease Onset Season   1. Spring: 31.7% of patients (most common) 2. Winter: 30.2% of patients 3. Autumn: 23.4% of patients 4. Summer: 14.7% of patients   Predisposing Factors   - 98 patients (36.9%) reported a predisposing factor  1. Upper respiratory infection: 67 patients 2. Gastrointestinal tract infections: 31 patients   Treatment (n, %)   1. Methylprednisolone (2 mg/kg/day): 92 (34.7%) 2. Pulse methylprednisolone (30 mg/kg): 19 (7.2%) 3. Non-steroidal anti-inflammatory drugs (NSAIDs): 143 (54%) 4. Cyclophosphamide: 9 (3.4%) 5. Mycophenolate mofetil: 3 (1.1%) 6. Colchicine: 8 patients 7. Plasmapheresis: 2 patients   Intussusception   1. More common in patients with renal involvement (p=0.002)   Nephrotic proteinuria & performance of biopsy procedure   1. More common among relapsing patients (p=0.004)   Fever, subcutaneous edema, and scrotal involvement   1. Statistically more frequent in patients with arthritis compared to others   MEFV Mutation Analysis   - Performed in 32 (12%) patients  1. Carried mutations (at least one allele): 14 (43.8%)   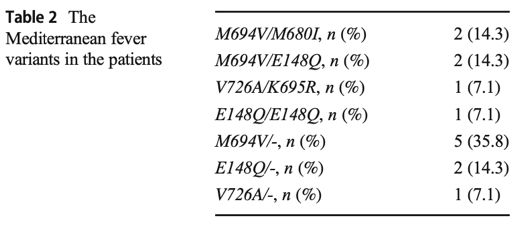  Comparison According to Gender   1. GIS bleeding: more frequent in males (p=0.007) 2. Clinical findings: similar in both genders 3. GIS involvement: more common in boys younger than 7 years (when 7 years or older, they had significantly more common GIS bleeding)   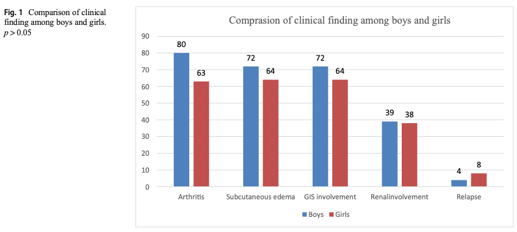  Adverse events:  n/a |
| Gokce, S., Kurugol, Z.,  2020,  Turkey | Predictive role of laboratory markers and clinical features for recurrent Henoch-Schonlein Purpura in childhood: A study from Turkey  Gokce, S., Kurugol, Z., Predictive role of laboratory markers and clinical features for recurrent Henoch-Schonlein Purpura in childhood: A study from Turkey. MODERN RHEUMATOLOGY, 2020. 30(6): p. 1047-1052. | Retrospective study  Objective: To evaluate whether there is a clinical significance of laboratory parameters and clinical features on the prediction of the recurrent Henoch Schonlein Purpura in children.  Inclusion criteria:   1. Diagnosis of HSP (according to the EULAR/PRINTO/PRES criteria) 2. Discharged from Ege University School of Medicine between January 2014 and January 2016   Exclusion criteria:   1. Patients diagnosed with HSP before January 2014   Study groups:  Group 1: first attack HSP  Group 2: recurrent HSP  Variables analyzed:   1. Demographic, etiologic, epidemiological, and clinical differences 2. Sex, age, seasonal pattern, and laboratory findings 3. Involvement of organs/body parts 4. Duration of first rash 5. C-reactive protein and albumin levels 6. Immunoglobulin A levels 7. Prediction for Risk of Recurrent HSP in Children   Definition of Relapse  Recurrence was defined when a patient previously diagnosed with HSP and was asymptomatic for at least 1 month without medication, presented again with a new flare of cutaneous lesions or other systemic manifestations of the vasculitis | n/a | N= 99 patients hospitalized with HSP analyzed  Duration of follow-up was a median of 1.3+/-0.7 years (range: 0.5-2.5 years)  Group 1 (First Attack HSP):  n=82/99 analyzed  Group 2 (Recurrent HSP):  n=17/99 analyzed  Mean age:  Group 1: 7.78 (SD ± 2.6 yrs)  Group 2: 6.8 (SD ± 3 yrs)  Sex (M/F): Group 1: 47/35 Group 2: 11/6 | n/a | Demographic, Etiologic, Epidemiological, and Clinical Differences  Summarized by Table 1  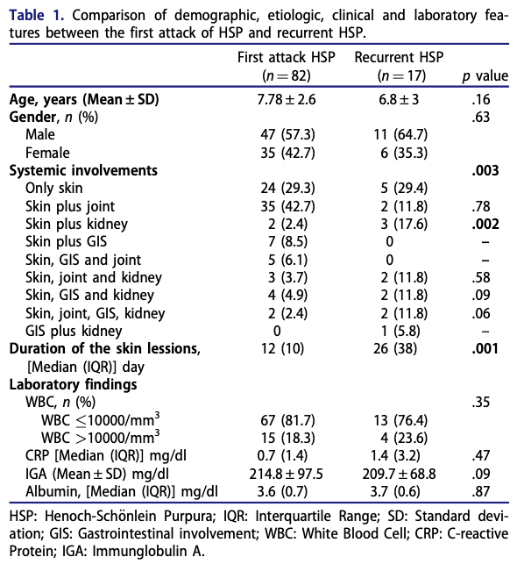  Sex, Age, Seasonal Pattern, and Lab Findings   1. No statistically significant difference between the groups   Involvement of Organs/Body Parts   1. Group 1: skin, and skin plus joint 2. Group 2: skin with kidneys   Duration of First Rash   1. Statistically longer in group 2 than group 1   C-Reactive Protein Albumin Levels   1. Within normal rage and no significant differences between groups   Immunoglobulin A Levels   1. No significant differences between groups   Prediction for Risk of Recurrent HSP  Laboratory Markers (IgA level, albumin level)   1. Not found to be predictive of recurrence   Skin Involvement During First Attack   1. Not found to be predictive of recurrence   Presence of the 4 System Involvement (Skin, Joint, Kidney, and GI Involvement all together)   1. Found to have a 17.2-fold higher risk of recurrence   Age and History of the Previous Infection   1. Not found to be related to recurrent HSP (according to multiple logistic regression)   Duration of Skin Manifestation   1. Not found to be predictive of recurrence (according to multiple logistic regression)   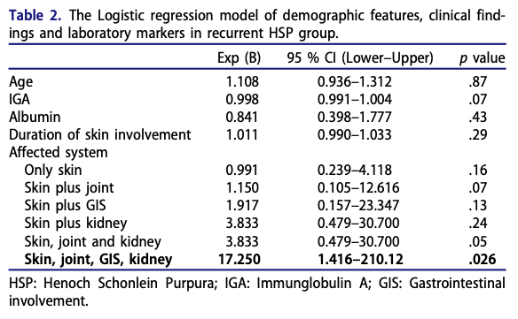  Adverse events:  n/a |
| Palmou, N., Calvo-Rio, V., 2015, Spain | Relapses and Predicitve Factors in Henoch-Schonlein Purpura. Study of 417 Patients  Data extracted from abstract | Retrospective non-randomized trial  Single centre  Objective: to analyze the frequency, type and risk factors for relapses in a large series of unselected patients with HSP.  Inclusion criteria:   1. Diagnosis of HSP (according to criteria proposed by Michel et al (J Rheumatol 1992; 19: 721-28))   Study groups:  n/a  Outcome measures:   1. Clinical manifestations 2. Treatment 3. Recovery 4. Relapse   Relapse Definition  Relapse was defined as a new outbreak of HSP in a previously asymptomatic patient (at least for one month) | n/a | N= 417 analyzed   - 315 (75.5%) were children or young people (20 years or less) - 102 (24.5%) adults   No groups  Mean age:  7.5 years  Sex (M/F): Group 1: 7/5 Group 2: 11/7 | No groups | Clinical Manifestations (onset/HSP established, %)   1. Skin lesions: 55.9/100 2. Nephropathy: 24/41.2 3. GI involvement: 13.7/64.5 4. Joint symptoms: 9.1/63.1 5. Fever: 6.2/20.4   Treatment (% of patients)   1. Corticosteroids: 35% (most frequently used) 2. NSAIDs: 14% 3. Cytotoxic agents: 5%   Recovery   1. Complete recovery: observed in most cases (n=346; 83.2%)   Relapses   1. Occurred in 133 patients (31.9%)   Note: median number of relapses was 2.4  Clinical Manifestations During Relapse   1. Cutaneous (89.6%) 2. Abdominal (27.1%) 3. Renal (25.9%) 4. Articular (16.8%)   Predictors of Relapse   1. Joint manifestations at disease onset 2. GI manifestations during course of disease 3. Corticosteroid treatment at the time of the first episode of HSP   Adverse events:  n/a. |
| Pirojsakul K., Tangnararatchakit K., 2012, Thailand | Clinical outcome of children with Henoch-Schonlein purpura nephritis  Journal of the Medical Association of Thailand / 2012;95(7):878-883 | Retrospective Cohort  Medical records of children diagnosed with HSP between 1998 and 2010 at Ramathibodi Hospital. The patients with urine protein to creatinine ratio (UPCR) of more than 1.0 g/g were identified and underwent renal biopsies. | Henoch-Schönlein purpura, Outcome, Nephritis, Renal, Pediatric patients | N = 20  Group 1 (n=7): Relapse group  Group 2 (n=13): Remission group  Age (median (range)):  Group 1: 9 (6-13)  Group 2: 8 (5-13) | Initial Treatment: All patients were treated with prednisolone 2 mg/kg/day and then tapered off according to degree of proteinuria during follow-up visit. Median time of prednisolone treatment was eight months (range 3-39 months). Twelve patients also received oral cyclophosphamide 2 mg/kg/day (maximum 100 mg/day) for three months while one patient also received monthly intravenous cyclophosphamide for six cycles (average dosage was 0.5 mg/kg/day). Fifteen patients received enalapril and subsequently stopped taking in eight patients. Median dosage of enalapril was 0.15 mg/kg/day.  Relapse defined: All patients had first resolution of proteinuria at median time of 6 months (range 2 to 47 months). Thereafter, thirteen patients still had no proteinuria until the last visit (remission group) while seven patients became proteinuric relapse (relapse group) without hematuria.  Treatment of Relapse: Relapse patients were treated by re-induction of prednisolone for three to six months and increase the dosage of enalapril. | In relapse patients, reintroduction of treatment led to: “reduction of UPCR was shown in all patients although some degrees of proteinuria were still detected at the last visit (median UPCR = 0.46 g/g).”  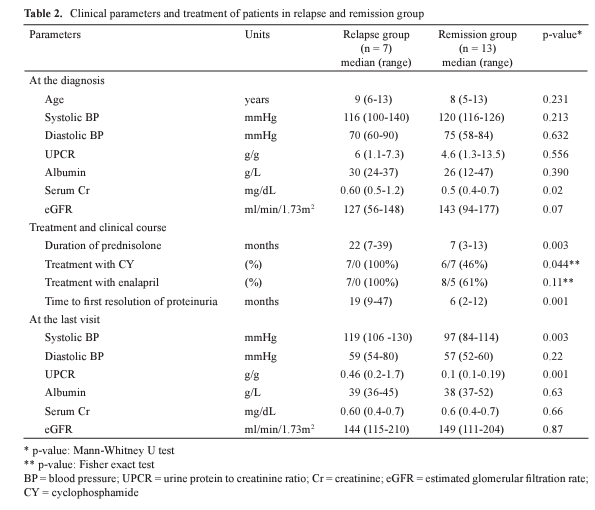 |
| Hackl A, Becker JU, 2018, Germany | Mycophenolate mofetil following glucocorticoid treatment in Henoch-Schonlein purpura nephritis: the role of early initiation and therapeutic drug monitoring  Pediatric Nephrology / 2018;33(4):619-629 | Uncontrolled trial  Included: Last 10 years (from December 2006 to December 2016) at Children’s hospital in Germany: 18 children with HSP having nephrotic-range proteinuria (defined as >40 mg/m2BSA/h or 2 g/gCrea—in most cases determined with a 24-h urine collection and in a few cases in spot urine samples) despite renin–angiotensin–aldosterone system (RAAS) blockade were biopsied in our center confirming the diagnosis of HSPN | Mycophenolic acid . Therapeutic drug monitoring . Pre-dose level . Cut-off of eMPA-AUC0-12h . Mechanism of action | N= 18  Mean age at onset: 9.6±3.4  Sex: 14M/4F  Mean follow-up time: 42.2 ±28.0 months | Treatment protocol: pulsed methylprednisolone (MP) was initiated on average 61.9 ±95.4 days after the first diagnosis (300 mg/m2BSA/ day on 3 alternate days), followed by prednisone at 60 mg/ m2BSA/day for 3 weeks, then 40 mg/m2BSA/day on alternate days for 4 weeks [29]. On average, 3 weeks (21.9 ± 17.5 days) after initiation of pulsed MP, the course of proteinuria was reevaluated, and on the basis of this evaluation (<50% decrease in proteinuria or proteinuria >40 mg/m2BSA/h or 2 g/gCrea) a decision was made to start MMF treatment. Thus, MMF was introduced as the second-line therapy on average 85.0±88. 0 days after the first diagnosis of HSP, 33.8 ± 47.6 days after the diagnosis of HSPN, and 21.9 ± 17.5 days after initiation of pulsed MP. The initial dose of MMF was 800–1200 mg/ m2BSA/day divided into two daily doses, which was later adjusted according to the result of repeated therapeutic drug monitoring (TDM) (performed within 3 months of therapy onset and yearly thereafter). TDM profiling was started 12 h after the last dose of MMF. For TDM, we used a limited sampling strategy based on measurements at 0 (MPA-C0) 30 and 120 min after drug intake.  Relapse Definition: Relapse was defined by proteinuria >40 mg/m2BSA/h or 2 g/gCrea, or deterioration of kidney function.  Relapse Treatment: Follow protocol above again | 2 of 18 children relapsed after therapy. |
| Teng MC, Wang LC, 2012, Taiwan | Kawasaki disease and Henoch-Schonlein purpura - 10 years' experience of childhood vasculitis at a university hospital in Taiwan.  Journal of microbiology, immunology and infection, 2012. 45: p. 22-30. | Retrospective cohort  vasculitis patients (onset age <18 years) who were admitted to the Pediatric Rheumatology Ward of the National Taiwan University Hospital, a tertiary referral center, from December 1997 to December 2007, and who satisfied the 1990 American College of Rheumatology (ACR) criteria. | HenocheScho¨nlein purpura; VAAD; Vasculitis-associated autoimmune disease | N= 124 with HSP vasculitis; of those, 47 have kidney involvement | Relapse defined: “the recurrence of clinical signs/symptoms or the occurrence of new symptoms after an initial remission, requiring the resumption of immunosuppressive therapy or an increased dose.” | Descriptive study of vasculitis in children, broadly. No specific outcomes related to HSP with kidney involvement |
| Rubino C, Monacelli C, 2021, Italy | Gastrointestinal involvement in IgA vasculitis: a single‑center 11‑year study on a cohort of 118 children  Clinical Rheumatology / 2021;40(12):5041-5046 | Retrospective cohort  Inclusion: Children hospitalized for IgAV at Meyer Children’s Hospital, from January 2010 to December 2020. Diagnosis of IgAV was confirmed if the 2008 EULAR/ PRINTO/PRES criteria were fulfilled. | Children · Gastrointestinal · Relapse · Immunoglobulin A vasculitis | N = 157 with 78 demonstrating Kidney involvement | Relapse Definition: A new flare of cutaneous lesions and/or other IgAV features, in a patient previously diagnosed with IgAV and asymptomatic for at least 1 month, was defined as relapse. | Descriptive study with limited IgAV data reported. |
| Liao CH, Tsai M, 2020, Taiwan | Onset age is a risk factor for refractory pediatric IgA vasculitis: a retrospective cohort study.  Pediatric rheumatology online journal / 2020;18(1):86 | Retrospective Cohort  medical records of the patients under 18years old with a diagnosis of IgAV from January 1999 to December 2018 at one tertiary medical center in Taiwan. | Pediatric IgA vasculitis, Onset age, Corticosteroid dependence, Refractory, Renal involvement | N = 484  Age (median (IQR)): 6.10 (4.72–8.58)  Sex: 252 M | Descriptive study with no comparison or treatment. Treatment of recurrence not defined.  Definition of Recurrence: “Recurrent IgAV was defined as disease flare-up after complete remission and discontinuation of all medications for at least 3 months”  “Complete remission was defined as the resolution of skin purpura, arthralgia/ arthritis, and abdominal pain, combined with normal renal function and absence of proteinuria and hematuria, as well as discontinuation of all medication.” | The overall recurrence rate of IgAV was 9.5%, and recurrence was more frequent in patients older than 6 years old (≤ 6years old, 7.3%; 6–12years old, 9.5%; 1218years old, 22.5%; P=0.0017). |
| Dawood SA, Abodiah AM, 2021, Saudi Arabia | Clinico-Epidemiological Profile and Outcome of Children with IgA Vasculitis in Aseer Region, Southwestern Saudi Arabia  Healthcare (Basel, Switzerland) / 2021;9(12): | Retrospective Cohort  Included: all hospitalized children in the past 5 years with a confirmed diagnosis of IgA vasculitis. Children enrolled in this study were ≤12 years old and were followed in the pediatric rheumatology clinic for variable periods, with a minimum of 2 years. IgA vasculitis was diagnosed in all cases according to the EULAR/PRINTO/PRES criteria | children; IgA vasculitis; abdominal pain; joints; renal involvement | N = 89  Mean Age: 5.87±2.812  Gender: 50M/39F | Descriptive study; no treatment or comparison  e not defined.  Relapse Treatment:  Two out of 15 relapsed cases were preceded by URTI and 80% of relapsed cases received steroids. | 15 of 89 children relapsed  Mean age of relapse: 7.13 ± 2.67  The duration of treatment for relapsed cases treated with steroids was 18.2±12.167 days, while cases without relapse treated with steroids had a duration of treatment of 14.28±11.685 days (p=0.622). |
| Wu, X., Zhou, D.,  2013,  China | Study of combined therapy with immunosuppressant in children with severe purpura nephritis  Wu, X., Zhou, D., Study of combined therapy with immunosuppressant in children with severe purpura nephritis. Pediatric Nephrology, 2013. 28(8): p. 1429  [data extracted from abstract] | Objective: To explore the therapeutic effect of prednisone (Pred) combined with Mycophenolate Mofetil (MMF) and Cyclosiporin A(CsA) in children with severe purpura nephritis (HSPN)  Inclusion:   1. Severe HSPN (from ISKDC IIIa to I) whose proteinuria of nephrotic syndrome did not significantly relieve after 4 weeks (when treated with oral Pred and MMF (20-30mg/(kg.d))     And (or)….   1. Gross hematuria did not disappear after 2 courses (when treated with large doses of methylprednisone)   Exclusion: n/a  Study groups:  Group 1: Oral CsA + Pred + MMF Treatment  Group 2: n/a  Outcome measures:   1. Gross hematuria 2. Remission 3. 24-hour urinary protein   Urine erythrocyte | n/a | N= 6 analyzed  Follow-up duration was 8-30 months  Group 1 (Combined treatment):  n= 6/6 pediatric patients analyzed  Mean age for pediatric patients:  Group 1: n/a (SD ± yrs)  Sex (M/F): Group 1: n/a | Group 1 (n=6):  2-4 mg/ (kg.d) of oral CsA to induce remission therap for 3-6 months, then gradually reduced to 1-3 mg/(kg.d)  Combined therapy with immunosuppressant treated a month later | 1-Month After Start of Treatment  Gross Hematuria   - Disappeared in all 5 patients   Remission   - Partial remission: 1 patient (17%) - Significant remission: 5 patients (83%)   24-Hour Urinary Protein   - 30.50 +/- 19.35 mg/kg   Urine Erythrocyte   - 15.23 +/- 11.39x104/ml   4-Months After Start of Treatment  Gross Hematuria   - n/a   Remission   - Complete remission: 1 patient (17%) - Significant remission: 5 patients (83%)   24-Hour Urinary Protein   - 18.67 +/- 10.10 mg/kg   Urine Erythrocyte   - 10.71 +/- 8.55x104/ml   10-Months After Start of Treatment  Gross Hematuria   - Disappeared in all 5 patients   Remission   - Complete remission: 1 patient (17%) - Significant remission: 5 patients (83%)   24-Hour Urinary Protein   - 7.66 +/- 6.31 mg/kg   Urine Erythrocyte   - 5.18 +/- 3.81x104/ml   Note: 4 patients were complete remission at last; median duration of complete remission was 8.5 +/- 4.93 months  Note: 2 patients had not reached complete remission, which follow-up was 8-9 months  Note: 3 patients were relapse during withdraw or reduction  Note: urea, nitrogen, and creatinine levels were normal  Adverse Outcomes   1. Hairy (4 patients) 2. Upper extremity tremor (2 patients) 3. Mild hypertension (2 patients) |
| Ren, P., Han, F.,  2012,  Switzerland | The combination of mycophenolate mofetil with corticosteroids induces remission of Henoch-Schonlein purpura nephritis.  Ren, P., Han, F., The combination of mycophenolate mofetil with corticosteroids induces remission of Henoch-Schonlein purpura nephritis. American journal of nephrology, 2012. 36(3): p. 271-277.  [data extracted from abstract] | Objective: To compare the effects of oral mycophenolate mofetil (MMF) with low-dose prednisone and the full-dose corticosteroids (CS; prednisone) for the induction therapy of HSPN with large proteinuria  Inclusion:   1. Biopsy-proven HSPN 2. Large proteinuria (>2.0 g/24 h)   Exclusion: n/a  Study groups:  Group 1: MMF Combined with Low-Dose Prednisone (MMF + Pred Group)  Group 2: Full-Dose Corticosteroids (CS Group)  Outcome measures:   1. eGFR 2. Urine protein (proteinuria) 3. Remission rate   Relapse | n/a | N= 53 analyzed  Outcomes were evaluated at 6-month follow-up and at the end of follow-up  Group 1 (MMF + Pred):  n= 27/53 pediatric patients analyzed  Group 2 (CS):  n= 26/53 pediatric patients analyzed  Mean age for pediatric patients:  n/a  Sex (M/F): n/a | Group 1 (n=27):  Oral MMF: 1.0 g/day (1.5 g/day for patients with a body weight >70 kg)  Combined with low-dose prednisone: 0.4-0.5 mg/kg/day  Group 2 (n=26)  Full-dose prednisone (0.8-1.0 mg/kg/day) | 6-Month Follow-Up  eGFR  Group 1: stable  Group 2: stable  Urine Protein  Group 1: decreased significantly  Group 2: decreased significantly  Remission Rate  Group 1: 55.5%  Group 2: 76.9%  p = 0.101  End of Follow-Up  Note: median follow-up of 28.8 months in group 2 and 28.2 months in group 1  Overall Remission Rate  Group 1: 77.8%  Group 2: 80.8%  p = 0.788  Relapse  Group 1: 0/21  Group 2: 4/21 (19.0%)  p = 0.115  Adverse Outcomes  n/a |
| Coppo, R., Mazzucco, G.,  1997,  United Kingdom | Long-term prognosis of Henoch-Schönlein nephritis in adults and children. Italian Group of Renal Immunopathology Collaborative Study on Henoch-Schönlein purpura Coppo, R., Mazzucco, G., Long-term prognosis of Henoch-Schönlein nephritis in adults and children. Italian Group of Renal Immunopathology Collaborative Study on Henoch-Schönlein purpura. Nephrology, dialysis, transplantation: official publication of the European Dialysis and Transplant Association - European Renal Association, 1997. 12(11): p. 2277–2283. | Multicentre collaborative study  Objective: to evaluate the progression of renal disease in children and adults with HSP nephritis severe enough to indi- cate renal biopsy, focusing the analysis on patients with follow-up longer than 1 year  Inclusion:   1. Adults 16 years of age and older at diagnosis 2. Children <16 years at diagnosis 3. Had a follow-up of 1 year or more 4. Reached the end-point of death or irreversible renal failure within the first year of follow-up 5. Had urinary abnormalities, IgA-dominant glomerular immune deposits, biopsy material available for review 6. Had palpable purpura and/or bowel angina   Exclusion: n/a  Study groups:  Group 1: Adults  Group 2: Children  Variables analyzed:   1. Outcome (remission, proteinuria, actuarial renal survival) 2. Risk factors for progressive nephritis 3. Treatment | n/a | N= 152 analyzed  Mean follow-up was 4.9+/-3.4 years  Mean follow-up was 4.8+/-3.9 years in children  Group 1 (Adults):  n= 95/152 patients analyzed  Group 2 (Children):  n= 57/152 pediatric patients analyzed  Mean age for patients:  27.5 (SD ± 18.3 yrs)  Sex (M/F ratio): Group 1: 1.5  Group 2: 1.7 | Group 1 (n=95):  Group 2 (n=57) | Outcome  Clinical Remission (%)  Group 1: 32.5  Group 2: 31.6  Minimal or Moderate Proteinuria, normal RF (%)  Group 1: 32.7  Group 2: 42.1  Nephrotic Proteinuria, normal RF (%)  Group 1: 3.2  Group 2: 1.7  Moderate functional impairment (%)  Group 1: 13.7  Group 2: 12.2  Severe functional impairment (%)  Group 1: 2.1  Group 2: 5.3  End-stage renal failure in dialysis (%)  Group 1: 15.8  Group 2: 7  Actuarial Renal Survival (%)  At 5 Years:  Group 1: 85%  Group 2: 95%  At 10 Years:  Group 1: 74.1%  Group 2: 73.1%  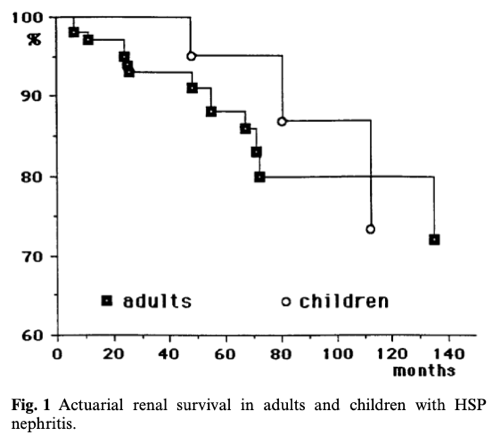  Risk Factors for Progressive Nephritis  Renal Lesions  Both cohorts included almost exclusively cases with crescents in <50% of glomeruli  Group 1:   1. Moderate or severe renal failure (%): 38.9 2. Renal failure progression to require dialysis (%): 17 3. Deterioration of renal function (%): 19.5   Group 2:   1. Moderate or severe renal failure (%): 18.5 2. Renal failure progression to require dialysis (%): 7.5 3. Deterioration of renal function (%): 23.6   Hypertension  Favourable Outcome (Remission or Moderate Proteinuria with Normal Renal Function) (%)  Group 1:   1. Normotensive adults (%): 70.6 2. Hypertensive adults (%): 45   Group 2:   1. Normotensive children (%): 73.1 2. Hypertensive children (%): 75   Required Chronic Dialysis Treatment (%)  Group 1:   1. Normotensive adults (%): 10.6 2. Hypertensive adults (%): 35   Group 2:   1. Normotensive children (%): 6.6 2. Hypertensive children (%): 8.3   Proteinuria  Favourable Outcome (Remission or Mild Proteinuria) (%)  Group 1: 89  Progression to End-Stage Renal Failure (%)  Group 1: 2.7  Note: The clinical outcome for children with absent or mild proteinuria was generally more favourable (82.1% were in remission or had mild proteinuria and 3.6% required dialysis) than those with higher levels of proteinuria, but statistical significance was not reached  Treatment  Note: Attention focused on patients with nephrotic syndrome and/or those with ≥50% glomeruli with crescents  Group 1 (n=34/95 [6 untreated])  Group 2 (n=27/57 [6 untreated])  Remission or Had Moderate Proteinuria and Normal RF at Follow-Up (%)  Treated adult patients: 42.7%  Untreated adult patients: 50%  Note: 25% of treated and 16.6% of untreated patients needed to start a chronic dialysis programme  Children with Good Outcome at Follow-Up (%)  Treated patients: 38%  Untreated patients: 33.3%  Children with Moderately or Severely Impaired Renal Function at End of Follow-Up (%)  Treated patients: 14.1%  Untreated patients: 16.6%  Note: since treatment was not given randomly, no definite conclusion can be drawn from this study  Adverse Outcomes  n/a |
| Ronkainen, J., Autio-Harmainen, H.,  2003,  Germany | Cyclosporin A for the treatment of severe Henoch-Schonlein glomerulonephritis.  Ronkainen, J., Autio-Harmainen, H., Cyclosporin A for the treatment of severe Henoch-Schonlein glomerulonephritis. Pediatr Nephrol, 2003. 18: p. 1138-1142. | Multicentre collaborative study  Objective: To describe here the efficacy of CyA treatment in seven HSP patients with nephrotic-range proteinuria  Inclusion:   1. Nephrotic-range proteinuria (>40 mg/h per m2) 2. Diagnosis of HSP   Exclusion: n/a  Study groups:  n/a  Variables analyzed:   1. Response Time to CyA Treatment 2. Remission 3. Relapse of proteinuria 4. GFR 5. Serum creatinine   Plasma cystatin C | End-stage renal disease, Immunosuppressive treatment, Nephrosis, Proteinuria, Hematuria | N= 7 analyzed  Mean HSP follow-up was 6 years (range 4.4-8.9 years)  n= 7/7 pediatric patients analyzed  Mean age for patients at diagnosis:  10.6 (range: 7.2-15.2)  Sex (M/F ratio): 5/2 | n/a | Response Time to CyA Treatment   - All patients responded at a mean of 1.4 months   Response achieved within 1-month: 4 cases (patients 2, 3, 5, and 7)  Response achieved within 2-months: 2 cases (patients 4 and 6)  Response achieved within 4-months: 1 case (patient 1)  Remission   - Achieved remission and without CyA treatment for a mean of 3.7 years: 4 patients   Proteinuria Relapse After Treatment Withdrawal  At 2-months: patient 2  At 8-months: patient 3  At 1 year and 5 months: patient 1  Note: CyA was started again and these three patients seemed to develop CyA dependence. They had received CyA treatment altogether for a mean of 4.0 years (range 2.8–5.7 years) by the end of the follow-up  Note: Two of the three CyA-dependent patients (patients 1 and 2) have been without CyA for the last 4 and 2 months, respectively.  Note: At the present time, patient 1 seems to be developing a relapse (proteinuria 3.5 g/day) and patient 2 has mild proteinuria (0.6 g/day), but neither is hypoalbuminemic (serum albumin 37.9 g/l and 42 g/l, respectively). The third patient (no. 3) is still receiving CyA and has mild proteinuria (0.6 g/day), but his serum albumin is normal (40.1 g/l).  Note: All 3patients are still considered to be CyA dependent.  Glomerular Filtration rate (GFR), Serum Creatinine, Plasma Cystatin C  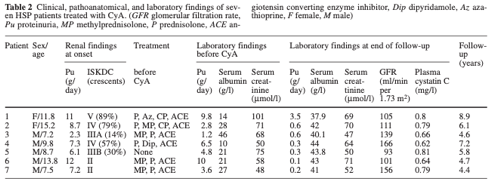  Adverse Outcomes   1. Headache (patient 2) 2. Increase in serum creatinine from 71 to 134 umol/l (patient 2) 3. Gastrointestinal symptoms (patient 5) 4. Increase in serum creatinine from 75 to 120 umol/l at the beginning of CyA treatment (patient 5) 5. Hirsutism (patients 1, 2, 5, 7) |
| Delbet, J.D., Guillaume, G.,  2019,  Netherlands | Histological prognostic factors in children with igA vasculitis with nephritis  Delbet, J.D., Guillaume, G., Histological prognostic factors in children with igA vasculitis with nephritis. Nephrology Dialysis Transplantation, 2019. 34(Supplement 1): a418.  [Data extracted from abstract] | Retrospective report  Multicenter  Objective: To report the prognosis of children with IgAVN depending on histological parameters  Inclusion:   1. IgAVN diagnosis between 2000 and 2015   Exclusion: n/a  Study groups:  n/a  Outcome measures:   1. Primary endpoint: remission 2. Acute glomerular or TI lesions 3. Chronic glomerular lesions 4. Median initial proteinuria 5. Median initial albuminemia 6. Median initial eGFR   Note: IgAVN remission defined as a proteinuria < 200mg/L and no kidney failure | n/a | N= 159 analyzed  Median follow-up of 37.4 months  No groups  Mean age for pediatric patients:  7.6  Sex (M/F): n/a | n/a | Remission at End of Follow-Up   - 112 (70%) of patients in remission   Acute Glomerular or TI Lesions  Acute glomerular lesions: 81% of patients  MP crescents: 86% of patients  EP crescents: 49% of patients  TIa lesions: 21% of patients  Chronic Glomerular Lesions  GS lesions: 6% of patients  TIc lesions: 7% of patients  Median Initial Proteinuria, Albuminemia, eGFR  Median initial proteinuria, albuminemia and eGFR were respectively of 330 mg/mmol, 32 g/l and median eGFR was 110 ml/min/1,73m2  Note: Chronic lesions were significantly associated to the absence of remission in multivariate analysis whereas EP, crescents and TIa were not associated to a poor prognosis  Adverse Outcomes  n/a |

**Table S9. Search Strategy Keywords and MESH terms for IgAN and IgAVN**

| Keywords | Mesh terms |
| --- | --- |
| IgA Nephropathy  IgA Vasculitis Nephritis  Pediatric  Diagnosis  Primary treatment/initial treatment  Relapse  Tonsillectomy  Transplant  Transition | exp immunoglobulin A nephropathy/  Glomerulonephritis.ti.  "immunoglobulin a nephropathy".ti.  IgAN.ti.  iga glomeruloneph*.ti.  IgAGN.ti.  berger* disease*.ti. iga-N.ti. exp IgA Vasculitis/  iga vasculitis.tw,kf.  igav.tw,kf.  immunoglobulin A vasculitis.tw,kf. exp anaphylactoid purpura/  anaphylactoid purpura.tw,kf. iga-v.tw,kf.  Henoch-Schonlein purpura.tw,kf.  HSPN.tw,kf. HSP-Iga-V.tw,kf.  (HSP adj3 iga-v).tw,kf. exp pediatrics/  pediatric*.tw,kf. paediatric*.tw,kf.  exp adolescent/  adolescen*.tw,kf.  exp juvenile/  juvenile*.tw,kf. youth.tw,kf.  child*.tw,kf. exp infant/  infan*.tw,kf. exp newborn/  newborn*.tw,kf. neonat*.tw,kf.  bab*.tw,kf. exp puberty/  (primary adj2 school*).tw,kf.  school age*.tw,kf.  kindergarten.tw,kf.  (elementary adj2 school).tw,kf. (nursery adj2 school).tw,kf.  prepubescen*.tw,kf. toddler.tw,kf. teen*.tw,kf.  "middle school".tw,kf.  high school.tw,kf.  highschool.tw,kf. secondary school.tw,kf.  schoolchild*.tw,kf.  diagnos*.ti.  initi*.tw.  prelim*.tw.  lab*.tw.  clinic*.tw.  follow*up.tw.  followup.tw.  followup.ti.  follow*up.ti.  exp treatment planning/  treat*.tw.  manag*.tw.  ACE.tw.  Exp Angiotensin-Converting Enzyme Inhibitors/  Angiotensin-Converting Enzyme Inhibitors.tw.  Exp Angiotensin II Type 1 Receptor Blockers/  Angiotensis II Type 1 Receptor Blockers.tw.  ARB.tw.  lifestyle.tw.  Glucocorticoids.tw.  exp Glucocorticoids/  exp Anti-inflammatory Agents, Non-Steroidal/  Non-steroidal immunosuppressive agents.tw.  non-steroid*.tw.  agent*.tw.  exp antihypertensive agents/  antihypertensive*.tw.  exp fish oils/  fish oil.tw.  exp vitamin D/  vitamin d.tw.  gastroprotect*.tw.  vaccin*.tw.  immune*.tw.  exp tonsillectomy/  tonsillectomy.tw.  atypical.tw.  exp nephrotic syndrome/  nephrotic syndrome.tw.  IgAN-ANCA.tw.  relaps*.tw.  exp recurrence/  recurr*.tw.  conclude*.tw.  discontin*.tw.  remission.tw.  transplant*.tw. |
| ti= terms in either title, abstract or keyword fields  tw=text word search in title or abstract fields  *=truncation  exp=exploded indexing term  /= indexing term  adj2= terms within two words of each other  kf= word in author provided keyword | |
